# From Bones to Bugs: Structure-Based Development of Raloxifene-Derived Pathoblockers That Inhibit Pyocyanin Production in *Pseudomonas aeruginosa*

**DOI:** 10.1021/acs.jmedchem.4c03065

**Published:** 2025-03-29

**Authors:** Marie Thiemann, Moritz Zimmermann, Christina Diederich, Huilin Zhan, Mikhail Lebedev, Jakob Pletz, Janosch Baumgarten, Maria Handke, Mathias Müsken, Rolf Breinbauer, Gabriela Krasteva-Christ, Esther Zanin, Martin Empting, Matthias Schiedel, Conrad Kunick, Wulf Blankenfeldt

**Affiliations:** † Institute of Medicinal and Pharmaceutical Chemistry, Technische Universität Braunschweig, Beethovenstraße 55, 38106 Braunschweig, Germany; ‡ Helmholtz Centre for Infection Research (HZI), Inhoffenstraße 7, 38124 Braunschweig, Germany; § Center of Pharmaceutical Engineering (PVZ), Technische Universität Braunschweig, Franz-Liszt-Straße 35a, 38106 Braunschweig, Germany; ∥ Helmholtz-Institute for Pharmaceutical Research Saarland (HIPS) - Helmholtz Centre for Infection Research (HZI), Campus E8.1, 66123 Saarbrücken, Germany; ⊥ Department of Pharmacy, Saarland University, Campus E8.1, 66123 Saarbrücken, Germany; # Faculty of Medicine, Institute for Anatomy and Cell Biology & Center for Gender-specific Biology and Medicine (CGBM), Saarland University, Kirrbergerstr. 100, 66424 Homburg, Saar, Germany; ∇ PharmaScienceHub (PSH), 66123 Saarbrücken, Germany; ○ Department Biologie, Friedrich-Alexander-Universität Erlangen-Nürnberg, 91058 Erlangen, Germany; ◆ Institute of Organic Chemistry, Graz University of Technology, Stremayrgasse 9, 8010 Graz, Austria; ¶ Partner Site Hannover-Braunschweig, German Centre for Infection Research (DZIF), 38124 Braunschweig, Germany; ⋈ Institute of Biochemistry, Biotechnology and Bioinformatics, Technische Universität Braunschweig, Spielmannstraße 7, 38106 Braunschweig, Germany

## Abstract

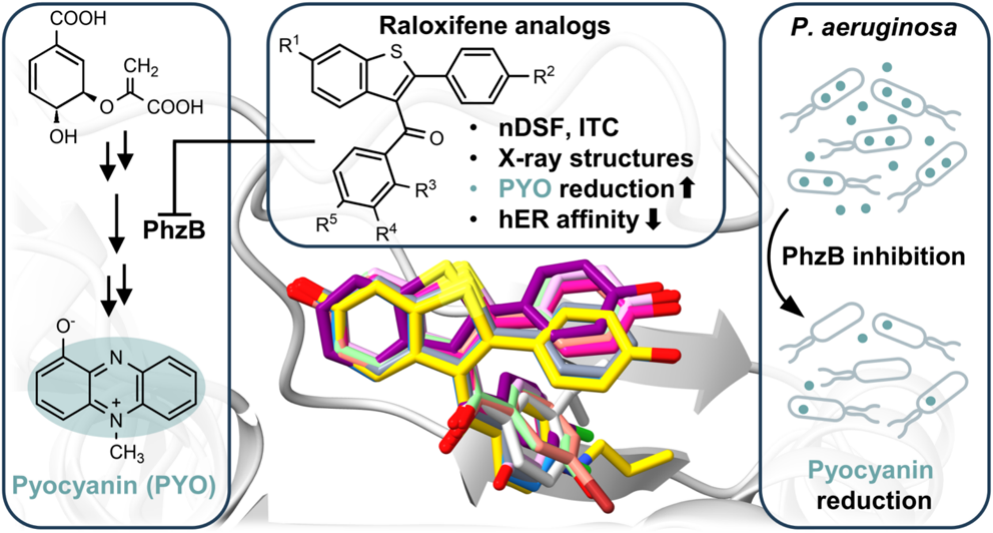

The human pathogen *Pseudomonas aeruginosa* is particularly notorious for its multiple resistance mechanisms. A new concept for anti-infectives is the “pathoblocker” approach, which targets virulence factors to disarm rather than kill pathogens and thus attenuates the development of resistance. Based on the estrogen receptor modulator raloxifene, which had previously been identified as a potential biosynthesis inhibitor of the virulence factor pyocyanin *via in silico* screening, analogues have been developed as pathoblockers against *P. aeruginosa*. These compounds reduce the production of pyocyanin by binding to the phenazine biosynthesis enzyme PhzB. Structure–activity relationships (SAR) were explored using nano differential scanning fluorimetry, isothermal titration calorimetry, and 12 X-ray cocrystal structures. Compared to raloxifene, congener **20c** shows a 60-fold lower affinity for the human estrogen receptor with a 15-fold increase in pyocyanin inhibitory activity. The comprehensive structural information gathered in this study paves the way for the development of improved pathoblockers with increased potency and selectivity.

## Introduction

Increasing resistance of bacterial pathogens to antibiotics poses a significant challenge to healthcare systems, accounting for an estimated 1.27 million deaths in 2019.^1^ Carbapenem-resistant *Pseudomonas aeruginosa* (CRPA) has been classified by the World Health Organization with high priority in terms of resistance toward classical antibiotics and the need for new anti-infectives.^2^ This Gram-negative, opportunistic pathogen can cause a wide variety of infections ranging from urinary tract and burn wound infections to pneumonia.^3,4^ It is particularly prevalent in nosocomial infections and chronic infections in cystic fibrosis patients.^5,6^ Even though this issue is well-known, there is a clear lack of innovation in the therapeutic pipeline.^2^

For instance, only one antibiotic with efficacy against CRPA was approved by the FDA between July 2017 and November 2021.^9,10^ Development of new agents against *P. aeruginosa* is challenging because of its high intrinsic resistance due to low membrane permeability, expression of efflux pumps, and antibiotic degrading enzymes.^11^

In recent years, pathoblockers (antivirulence drugs) have gained attention as a potential strategy for combating infections.^12,13^ This approach aims to interrupt bacterial virulence without killing the pathogen, thus decreasing the risk of resistance development due to reduced selection pressure. Pathoblockers are designed to work either alone (*e*.*g*., as a prophylactic treatment option) or as an add-on treatment combined with an antibiotic.

Recently, pathoblockers against various virulence factors of *P. aeruginosa* were reported and proved the concept of virulence inhibition.^12,14,15^ This is either possible by the inhibition of specific virulence factors or by targeting the quorum sensing system (QS), which controls the expression of virulence factors in a cell-density-dependent manner. Along these lines, inhibitors of the metalloprotease LasB resulted in reduced virulence in various infection models.^16^ Moreover, inhibitors of the QS receptor PqsR have been shown to reduce pyocyanin production and biofilm formation.^17−20^ Furthermore, PqsR inhibitors enhanced the efficacy of tobramycin in a murine infection model, demonstrating the beneficial effects of combining a virulence factor inhibitor with a backbone antibiotic.^18^

One of the best-studied virulence factors of *P. aeruginosa* is the phenazine pyocyanin (**4**). Pyocyanin has several effects mainly due to its redox activity.^21,22^ These include the generation of reactive oxygen species, such as hydrogen peroxide, which was causally linked to cytotoxicity in A549 respiratory cells.^23^ Furthermore, phenazines are involved in biofilm formation by acting as an electron transport shuttle in anaerobic layers of the biofilm^24,25^ and iron homeostasis *via* Fe(III)-reduction.^26,27^ Additionally, pyocyanin functions as a terminal QS signaling molecule by activating the transcription factor SoxR. Effects of SoxR activation include the expression of the efflux transporter MexGHI-OpmD and the mono-oxygenase PA2274.^28^ Importantly, pyocyanin is known to be critical for full virulence of *P. aeruginosa* in a variety of host organisms,^29,30^ especially in acute lung infection models in mice.^31^ Hence, the inhibition of pyocyanin biosynthesis is an attractive strategy for the development of new pathoblockers.

**Figure 1:**
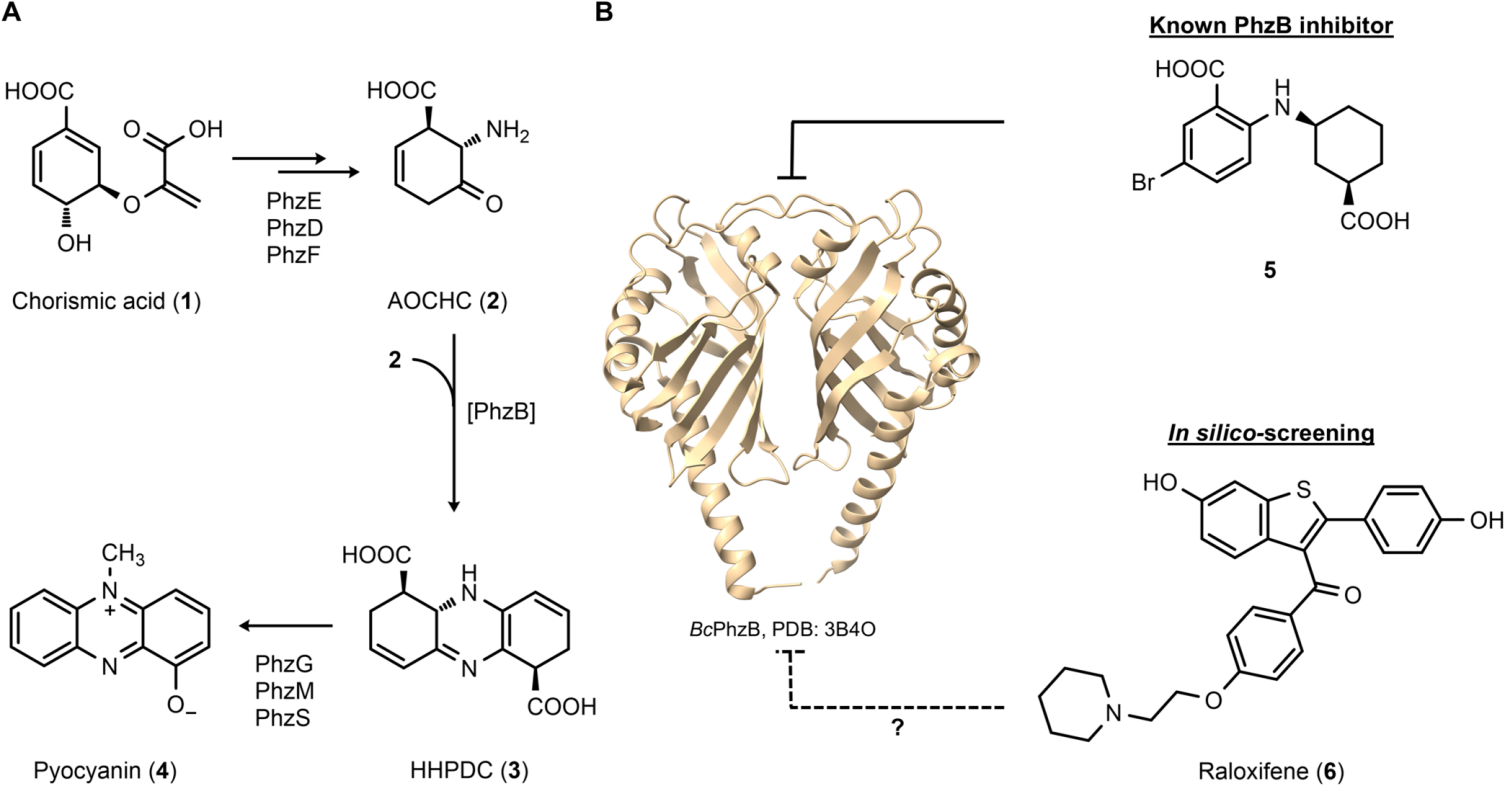
**A**. Overview of the biosynthesis pathway of pyocyanin (**4**). **B**. Crystal structure of homodimeric *Bc*PhzB (PDB: 3B4O(7)) and structure of the known PhzB ligand **5**. The SERM raloxifene (RAL, **6**) was proposed as a potential PhzB inhibitor by *in silico* studies and reduces pyocyanin production in *P. aeruginosa*.^8^

The starting point of the biosynthetic pathway of phenazines is chorismic acid (**1**) (Figure 1A).^32,33^ Modifications by the enzymes PhzE,^34^ PhzD,^35^ and PhzF^36,37^ lead to the highly reactive aminoketone intermediate AOCHC (**2**).^38^ Two AOCHC molecules then condense to the tricyclic phenazine scaffold HHPDC (**3**), either spontaneously or catalyzed by the enzyme PhzB, which is a homodimer and belongs to the nuclear transport factor 2/ketosteroid isomerase family.^7,39^**3** can be further converted into different biologically active phenazines such as pyocyanin (**4**) by the enzymes PhzG,^40,41^ PhzS,^42^ and PhzM,^43^ respectively.^38^

PhzB is not essential for the formation of HHPDC (**3**), but it greatly enhances this process *in vivo*. Pyocyanin production in a *P. aeruginosa* PA14 *phzB1*-knockout mutant, in which the *phzB* gene in the first of the two phenazine biosynthesis operons found in the genome of *P. aeruginosa* is deleted,^44^ is reduced by approximately 80%. This resulted in a 1000-fold decreased bacterial load in an acute murine lung infection model, comparable to a *pqsR*-knockout mutant. The tissue damage was reduced from entire lobes of the lungs to smaller and more scattered areas, making the infection less severe compared to the wild type. The remaining pyocyanin production in *phzB1*-knockout mutants (≈20%) can probably be attributed to *phzB2* activity or to spontaneous dimerization of AOCHC (**2**), but appears to play only a minor role for infection.^31^

PhzB in its homodimerized form displays binding sites for two substrate molecules (**2**) in each monomer. The C-terminus of one monomer functions as a lid, shielding the binding site of the neighboring monomer and thereby contributing to substrate binding and stabilization of the complex.^39^ In pseudomonads, the *phz*-operon carries the highly similar PhzB copy PhzA, which is suspected to be inactive due to mutations in the catalytic and binding amino acids (Figure S1).^39^

Several inhibitors that affect pyocyanin production indirectly by blocking the regulatory QS system of *P. aeruginosa*, such as the PqsR inhibitors described above, have been reported. However, these compounds may have the disadvantage that broad modulation of the QS system could lead to unintended consequences, since random mutations in these regulatory genes or in their promoter regions could confer substantial advantages to the bacteria, potentially allowing them to bypass the effects of inhibition. PhzB inhibitors, on the other hand, would interfere with the biosynthesis pathway of pyocyanin directly, thus offering a more targeted approach. This would reduce pyocyanin levels while minimizing interference with other vital QS-regulated cellular processes, resulting in being less prone to the development of new resistances. In addition, by acting as pathoblockers with a new mode of action, PhzB inhibitors might extend the therapeutic options available against isolates that have already acquired drug resistance.

Currently, only a limited number of direct inhibitors of pyocyanin biosynthesis have been reported, primarily targeting the enzymes PhzB and PhzS (Table S1).^39,45,46^ Among these, the few inhibitors that act on PhzS exhibit a two-digit micromolar binding affinity and reduce pyocyanin production at high concentrations of 100 μM only.^45^ In addition to this low potency, it is important to emphasize that the inhibition of PhzS only impacts the conversion of already biosynthesized phenazines into pyocyanin, while the generation of other phenazines that also play significant roles in infection and biofilm development is not affected.^47,48^ The previously reported PhzB ligands are substrate analogues mimicking the binding of the two AOCHC molecules in the active site (*e*.*g*., **5**, see Figure 1B) and display nano to low micromolar affinity to a PhzB ortholog from *Burkholderia cepacia* (*Bc*PhzB). While for **5** and its analogues binding to PhzB was demonstrated via ITC and cocrystallization in previous studies with *Bc*PhzB, no data have been reported on the effects on pyocyanin biosynthesis in *P. aeruginosa*.^39,46^

Based on a computational drug repurposing study, Ho Sui et al. proposed the FDA-approved selective estrogen receptor modulator (SERM) raloxifene (RAL, **6**) as a potential PhzB inhibitor.^8^ RAL (**6**) reduced pyocyanin production in cultures of *P. aeruginosa* when treated with high doses (0.21 mM), while not affecting bacterial growth.^8^ In general, repurposing of approved drugs such as RAL (**6**) offers the advantage of a proven safety profile over a long period of time in patients, making it a promising starting point for the development of new therapeutic agents.

In this study, we report RAL-derived PhzB inhibitors with considerably improved potency for inhibition of pyocyanin production and decreased affinity at the human estrogen receptor-α (hER-α). After confirming PhzB as the target of RAL (**6**) by cocrystallization, the obtained structural insights were used to design RAL analogues. Initially, the binding of these compounds to PhzB was evaluated using nano differential scanning fluorimetry (*n*DSF). The affinity of selected compounds was determined via isothermal titration calorimetry (ITC). Subsequently, cocrystal structures of several ligands were determined and used for the structure-based design of novel analogues. All compounds were tested for their potential to reduce the pyocyanin production in *P. aeruginosa* PA14. Furthermore, off-target affinity of representative compounds was assessed by a binding assay toward hER-α.

## Results

### Cocrystallization and Binding Mode of RAL (**6**) in PhzB

**Figure 2:**
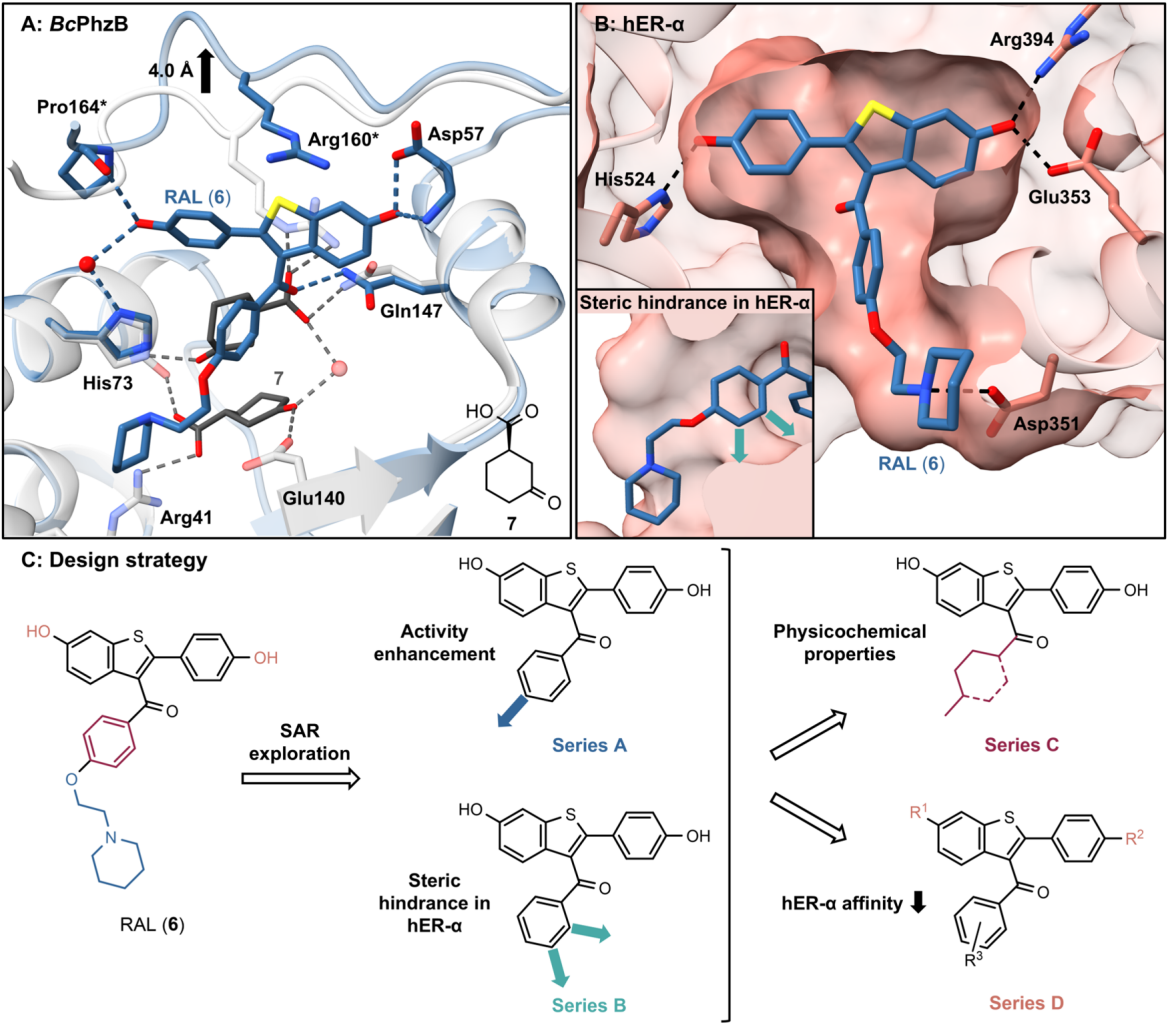
Design strategy of new PhzB inhibitors based on the binding modes of RAL (**6**) observed in cocrystal structures with *Bc*PhzB and hER-α, respectively. **A**. Superimposed cocrystal structures of *Bc*PhzB with RAL (**6**, PDB: 9F8H, blue) and substrate analogue (1*R*)-3-oxocyclohexanecarboxylic acid (**7**) (PDB: 3DZL,^49^ gray). Binding of RAL (**6**) is mediated by hydrogen bonds from the 6-hydroxy and 4′-hydroxy groups to Asp57 and Pro164* as well as from the carbonyl oxygen of the 3-acyl moiety to Gln147. With RAL (**6**) bound, the C-terminus is shifted upward (black arrow, distance between Asp161* Cα atoms). **B**. Binding mode of RAL (**6**) in hER-α (PDB: 1ERR(50)). The benzoyl moiety is located in a narrow part of the binding pocket, providing space for substitution in the 4-position, but presumably not in 2- and 3-positions (green arrows). **C**. Design strategy for PhzB inhibitors based on RAL (**6**). Series A aims at improving the on-target activity by a replacement of the 2-ethoxypiperidine moiety. Series B was designed to reduce affinity to the off-target hER-α by steric hindrance, while maintaining the on-target activity of series A. Series C aimed to improve solubility by increasing Fsp^3^. Series D combines the most promising moieties of series A and B, while alternating the substitution pattern of the hydroxy groups in 6- and 4′-positions providing a second strategy to reduce the off-target affinity toward the hER-α.

While PhzB was proposed as RAL’s (**6**) target in pyocyanin reduction based on *in silico* studies, direct interaction of RAL (**6**) and PhzB has not been validated experimentally.^8^ To this end, we initially confirmed the target by performing cocrystallization of RAL (**6**) with a PhzB ortholog from *Burkholderia cepacia* (*Bc*PhzB). The active site of *Bc*PhzB features the same amino acids as the target enzymes from *P. aeruginosa* (overall sequence identity: 58–59%, sequence alignment: Figure S1) but crystallizes more readily than the *P. aeruginosa* proteins. A cocrystal structure of *Bc*PhzB with RAL (**6**) was obtained at a resolution of 1.5 Å (PDB: 9F8H, Figure 2). RAL (**6**) binds in a different part of the active site compared to the previously reported binding mode of the substrate analogue (1*R*)-3-oxocyclohexanecarboxylic acid (**7**) and induces a shift of the C-terminus of the second monomer, which acts as a lid to the active site.^39^ This shift enlarges the binding pocket to generate space for the bulkier RAL (**6**) molecule (Figure 2A). Binding of RAL (**6**) to *Bc*PhzB is mediated by the 6-hydroxy-2-(4-hydroxyphenyl)benzo[*b*]thiophene scaffold and the 3-acyl moiety. The 6-hydroxy group forms two hydrogen bonds to Asp57 by involving the side chain and the nitrogen of the protein backbone, respectively. The 4′-hydroxy group interacts with the backbone carbonyl of Pro164* (Pro164 of the second protein monomer). This pushes the C-terminus of the second monomer upward and provides additional stabilization. The carbonyl oxygen of the 3-benzoyl moiety forms a hydrogen bond to Gln147 while the 2-ethoxypiperidine moiety is apparently not engaged in polar interactions with the host protein.

### Chemistry

#### Design Strategy

The design of PhzB inhibitors was based on the obtained cocrystal structure of RAL (**6**) bound to *Bc*PhzB (Figure 2C). In the first series, structure–activity relationship (SAR) of the benzoyl substitution pattern was investigated while preserving the parent scaffold responsible for interaction with PhzB. Initially, the influence of substitution in the benzoyl 4 position was studied (series A).

As substitutions in the benzoyl 2- and 3-positions appeared to sterically hinder binding to the hER-α, these locations were explored to decrease affinity toward this off-target while maintaining on-target activity for PhzB (series B, Figure 2B). Series A and B resulted in compounds with considerably improved activity, but also unfavorable physicochemical properties, primarily due to their high hydrophobicity and concomitant low solubility. Therefore, bioisosteric replacement of the benzoyl group by aliphatic structures was investigated, resulting in an increase in the sp^3^/sp^2^-ratio (Fsp^3^) (Series C).

The 6- and 4′-hydroxy groups of RAL (**6**) are important for binding to hER-α as they mimic the hydroxy groups of the physiological ligand estradiol.^51^ In previous studies, replacement of these substituents with different non-hydrogen-donors resulted in 1–100-fold affinity reduction toward hER-α.^52^

This finding was taken into account with a second strategy to decrease hER-α off-target affinity. Here, new PhzB inhibitors lacking one or both phenol groups (series D) were designed based on the most promising compounds derived from series A (**10d**) and B (**10i**).

#### Syntheses

**Scheme 1:**
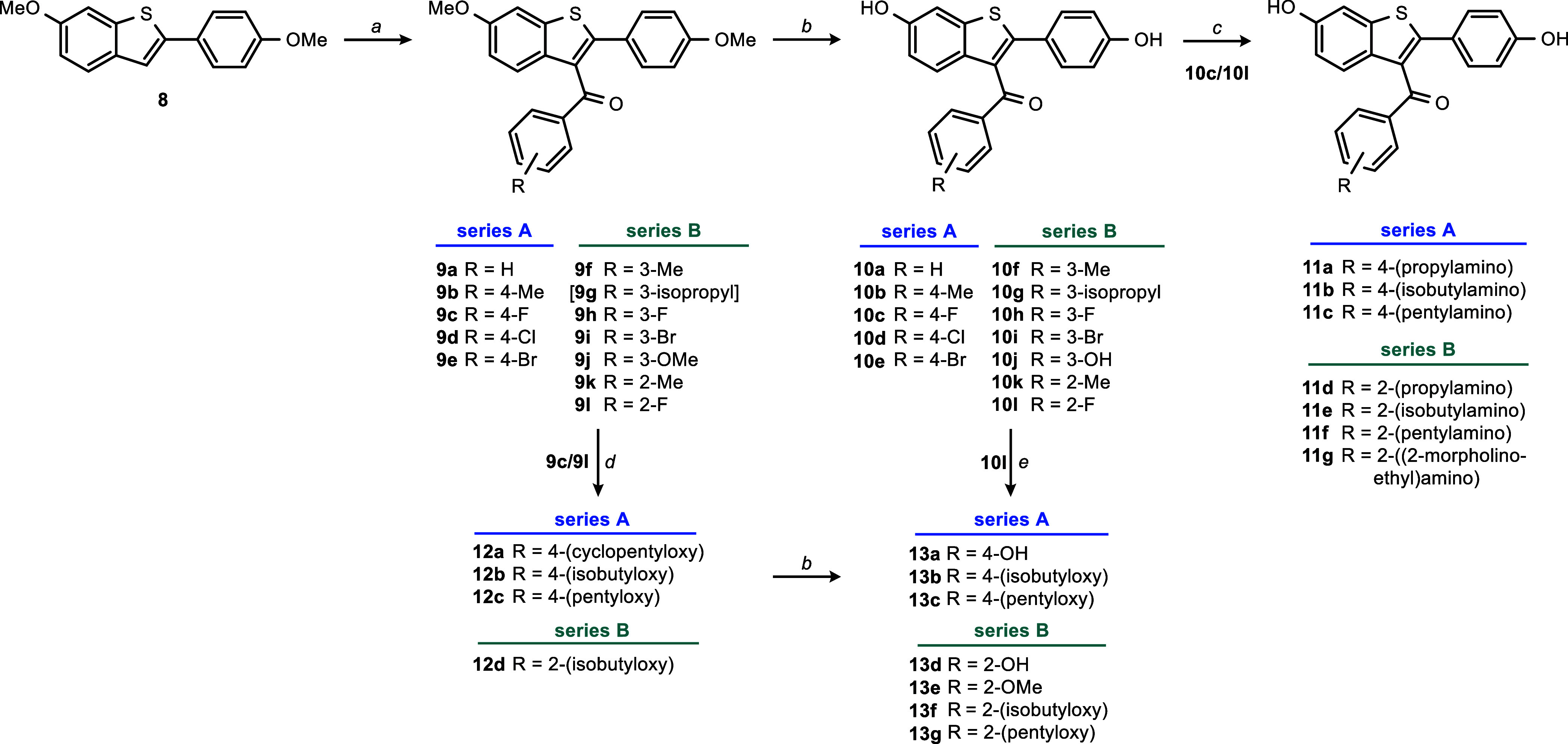
Synthesis Routes of Series A and Series B Reagents and conditions: (a) for **9a**–**f**, **9h**–**l:** appropriate carboxylic acid chloride, AlCl_3_, CH_2_Cl_2_, 0 °C → rt, 4.5–30 h, 17–91% yield; for **9g:** (i) SOCl_2_, 3-isopropylbenzoic acid, *N*,*N*-dimethylformamide (DMF), 80 °C, 2 h; (ii) AlCl_3_, CH_2_Cl_2_, 0 °C → rt, 6 h. (b) BBr_3_, CH_2_Cl_2_, rt, 2 h–2.5 days, 6–86% yield. (c) for **11a**–**11c**: **10c**, for **11d**–**11g**: **10l**, appropriate amine, DMSO, reflux, 5–6 h, 26–79% yield. (d) for **12a**–**12c**: **9c**, for **12d**: **9l**, appropriate alcohol, NaH, DMF, rt, 2.5 h, 37–92% yield. (e) for **13e**–**13g**: **10l**, appropriate alcohol, NaH, DMF, 50 °C, 1–4 h, 43–85% yield. Compounds shown in square brackets were used for the subsequent reactions directly without being isolated or characterized.

Syntheses of RAL analogues were modified based on previously published methods. Syntheses of the 3-substituted 6-hydroxy-2-(4-hydroxyphenyl)benzo[*b*]thiophenes of series A and B were performed starting from commercially available 6-methoxy-2-(4-methoxyphenyl)benzo[*b*]thiophene (**8**) (Scheme 1).^53^**8** was acylated in the 3-position via Friedel–Crafts acylation (FCA) using the corresponding carboxylic acid chlorides. Carboxylic acid chlorides that were not commercially available were prepared using the corresponding carboxylic acid and thionyl chloride. FCA yielded the dimethoxy derivatives **9a**–**l**. Subsequent *O*-demethylation of the methoxy groups in 6- and 4′-position using boron tribromide resulted in the dihydroxy derivatives **10a**–**l**.

In order to enable diverse substitution of the 3-benzoyl moiety, fluoro-substituted derivatives **9c/l** and **10c/l** were employed as substrates for nucleophilic aromatic substitution. Ether synthesis with sodium hydride was used for reaction with the corresponding alcohols, analogous to published methods.^53^ The 4′-hydroxy derivative **13a** and 4′-alkoxy derivatives **13b/c** were obtained starting from **9c** with subsequent *O-*demethylation.

Since the intended *O*-demethylation of **12d** resulted in additional *O*-dealkylation and yielded the free phenol **13d**, substitution with alcohols in 2-position was performed, starting from the already *O*-demethylated **10l**, resulting in **13e**–**g**. The amine derivatives **11a**–**g** were obtained by reaction of the aryl fluorides **10c** and **10l** with the respective amines.

**Scheme 2:**
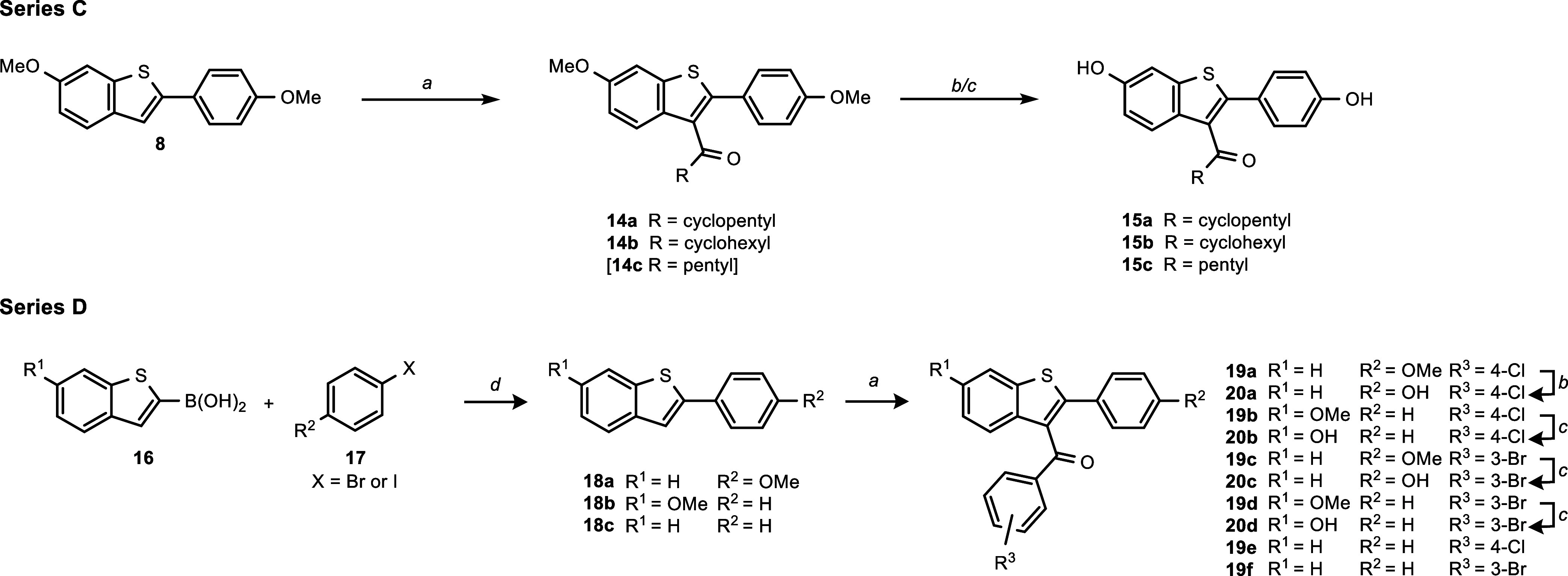
Synthesis Routes for Series C and Series D Reagents and conditions: (a) appropriate carboxylic acid chloride, AlCl_3_, CH_2_Cl_2_, 0 °C → rt, 2–27.5 h, 20–96% yield. (b) BBr_3_, CH_2_Cl_2_, rt, 2–3.5 h, 16–36% yield. (c) Pyridinium chloride, μW: stepwise heating to 180 °C, ramp to temperature, max. 20 W, 300 psi, 10 mL vessel, 2–3.5 h, 45–96% yield. (d) For **18a**: 4-bromoanisole, for **18b/c**: iodobenzene, Pd(PPh_3_)_4_ (5 mol %), K_2_CO_3_, 1,4-dioxane/H_2_O (4:1 v/v), μW: 120 °C, 300 W, 70 psi, 30 min, 42–90% yield. Compounds shown in square brackets were used for the subsequent reactions directly without being isolated or characterized.

Preparation of series C was carried out by acylation of **8** with aliphatic acid chlorides similar to series A and B but resulted in lower yields due to a reduced selectivity of the FCA. Subsequent *O*-demethylation with boron tribromide was not suitable for the cyclic analogues **14a**/**b**, thus **15a**/**b** were obtained by *O*-demethylation using pyridinium chloride (Scheme 2, top).

For series D, Suzuki coupling was used to obtain substituted 2-phenylbenzo[*b*]thiophenes **18a**–**c**, which were subsequently acylated by FCA yielding the methoxy derivatives **19a**–**f**. Finally, *O*-demethylation using boron tribromide or pyridinium hydrochloride yielded the free phenols **20a**–**d** (Scheme 2, bottom).

### Characterization of RAL Derivatives by *n*DSF

**Figure 3:**
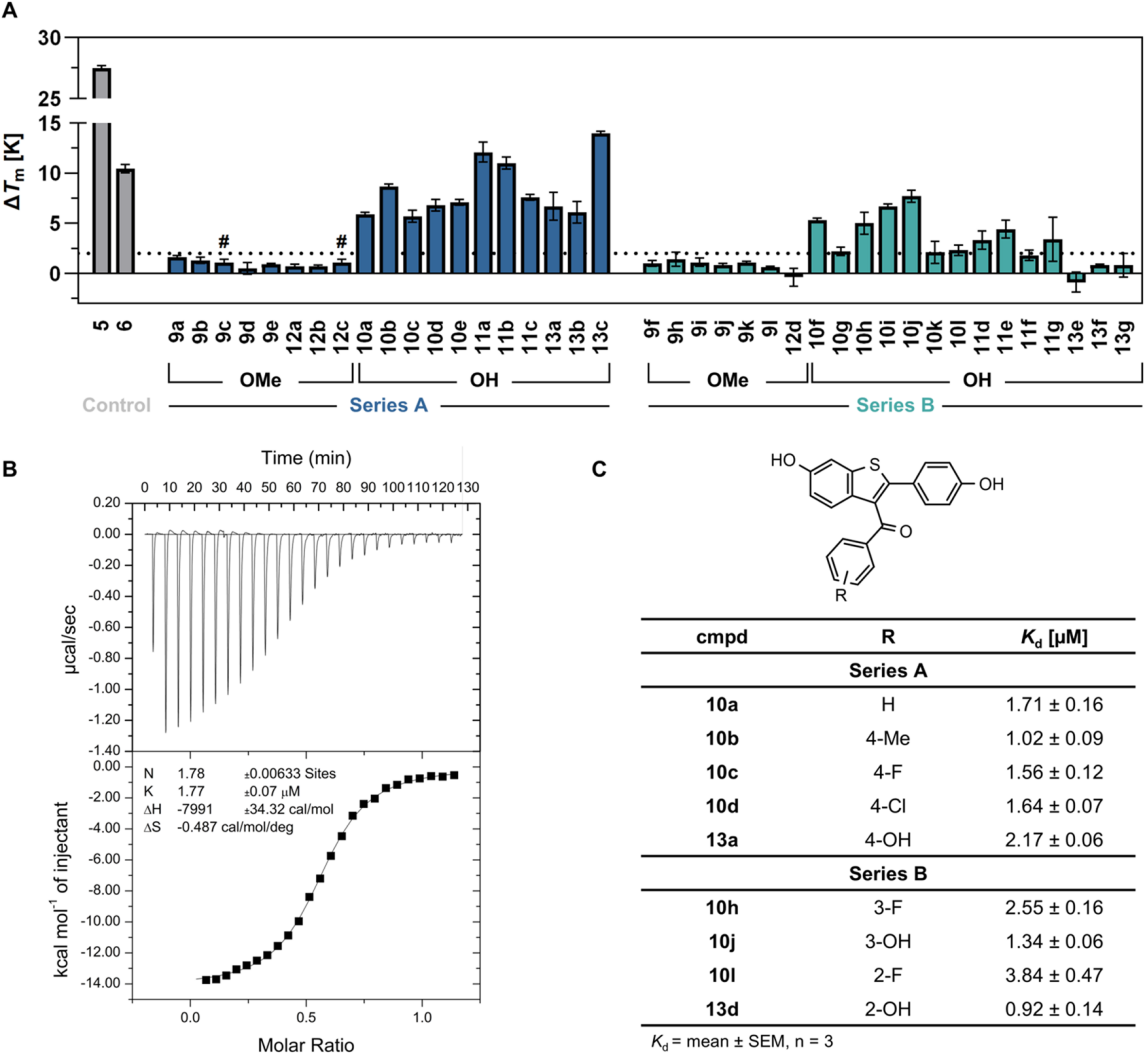
Results of nano differential scanning fluorimetry (*n*DSF) and isothermal titration calorimetry (ITC). **A**. Melting point changes (Δ*T*_m_, mean ± SD, triplicate measurements, ^#^: duplicate measurements) of *Bc*PhzB (ortholog from *Burkholderia cepacia*) incubated with compounds (*O*Me: 6,4′-dimethoxy, OH: 6,4′-dihydroxy) in comparison to DMSO (Table S2). The dotted line indicates a positive Δ*T*_m_ of 2 K. **B**. Representative ITC titration curve of **10d** (40 μM) titrated with *Bc*PhzB (300 μM). **C**. *K*_d_ values (mean ± SEM, *n* = 3) for **10a**–**d/h/j**/**l** and **13a**/**d** determined by ITC. ITC traces for all tested compounds are shown in Figures S3–S13.

Nano differential scanning fluorimetry (*n*DSF) was performed as an initial test to detect binding of the synthesized compounds to PhzB (Figure 3A). In analogy to the cocrystallization studies (*vide infra*), *Bc*PhzB was used. The known PhzB inhibitor **5** and RAL (**6**) showed high *T*_m_ shifts of +27.3 and +10.1 K, respectively. Initial docking studies suggested that the dimethoxy derivatives (**9a**–**l**, **12a**–**d**) were unlikely to fit into the binding pocket of *Bc*PhzB (Figure S2). This was confirmed by small *T*_m_ shifts in this assay. In contrast, most of the dihydroxy analogues (**10a**–**13d**) displayed a robust shift in *T*_m_ (>2 K, dotted line in Figure 3A), corroborating the hypothesis of binding to *Bc*PhzB.

Melting temperature shifts of *Bc*PhzB ranged from +5.7 to +14.0 K for series A. A similar trend was observed for most dihydroxy analogues of series B with smaller *T*_m_ shifts for 2′-substituted compounds (**10k/l** and **11d–g**) of +1.8 to +4.4 K and the 2′-alkoxy derivatives **13e**–**g** being the only dihydroxy compounds not showing a robust shift in *T*_m_ (−0.9 to +0.8 K). The results obtained from *n*DSF indicate the binding of the majority of dihydroxy compounds of series A and B to *Bc*PhzB.

### Isothermal Titration Calorimetry (ITC)

To further characterize on-target binding, the dissociation constants (*K*_d_) of selected ligands toward *Bc*PhzB were determined using ITC (Figure 3B/C). A reverse ITC setup, with the ligand in the cell and the protein in the syringe, was used due to the low solubility of most ligands. Based on their weak effects in the *n*DSF assay, the dimethoxy precursors were not further evaluated. Affinities determined for ligands of series A and B showed binding to *Bc*PhzB in the low micromolar concentration range, with the best compounds displaying *K*_d_ values of 1.02 μM (**10b**) and 0.92 μM (**13d**), respectively (Figures S3–S12). Binding curves for ligands with larger substituents such as ethers (**13b**/**c**, **13e**–**g**) or amines (**11a**–**g**) could not be retrieved from ITC experiments, due to low *c*-values of the titration curves (Figure S13). Enhancement of *c*-values by increasing the ligand concentration was limited due to low compound solubility. Accordingly, it was not possible to determine the *Bc*PhzB affinity of RAL (**6**) using ITC.

### Cocrystal Structures

**Figure 4:**
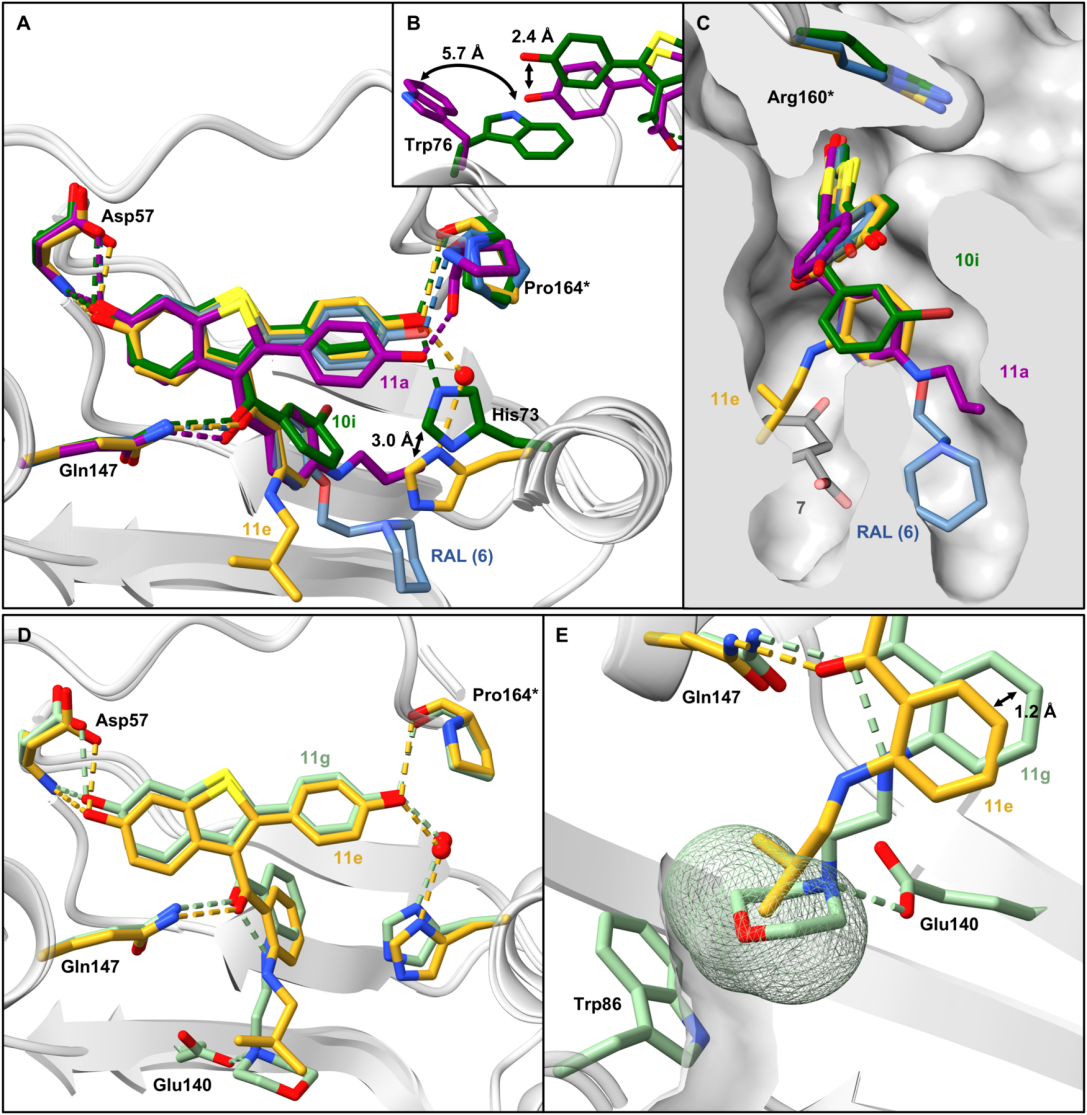
X-ray cocrystal structures of RAL (**6**, blue, PDB: 9F8H), **10i** (dark green, PDB: 9F8J), **11a** (violet, PDB: 9F8M), **11e** (gold, PDB: 9F8N), and **11g** (mint, PDB: 9F8O) bound to *Bc*PhzB (gray). Hydrogen bonds and side chains of the protein are colored in the same color as the respective ligand. **A**. Binding mode of RAL derivatives, exemplified by **10i**, **11a**, and **11e**, in comparison to RAL (**6**). **B**. A deeper position of the 2-phenyl moiety in **11a** leads to a shift of Trp76 into a different rotamer compared to **10i**. **C**. Surface representation of the *Bc*PhzB binding pocket (gray). The 2-, 3-, and 4-substituents at the benzoyl moiety (**11e**, **10i**, **11a**) occupy different binding sites in *Bc*PhzB. **D**. Comparison of the binding modes of **11e** and **11g**. **E**. Van der Waals surface of the morpholine ring of **11g** and Trp86. In comparison to **11e**, the 3-benzoyl moiety of **11g** is shifted upward.

The binding mode of several ligands (**10g/i/j**, **11a/e/g**, **13a/d**) was investigated in cocrystallization experiments with *Bc*PhzB. Most ligands interacted similarly to the parent compound RAL (**6**). Representative examples of **10i**, **11a**, and **11e** are shown in Figure 4A: the 6-hydroxy group of the parent scaffold forms two hydrogen bonds with Asp57, one with the side chain and one with the backbone, respectively. The carbonyl oxygen of the 3-benzoyl moiety interacts with Gln147.

Most ligands form a hydrogen bond from the 4′-hydroxy group to Pro164* and thus lock the C-terminus of the second monomer in the same way as the starting compound does. Like many amino acid residues in the binding site, both His73 and Trp76 appear to be highly flexible and adapt to the ligand’s position (Figure 4A/B). Therefore, the 4′-hydroxy group additionally forms a direct (**10i**) or water-mediated hydrogen bond (**11e**) to His73 depending on the position of the bound ligand (Figure 4A).

While substituents in the 4-position of the benzoyl moiety (series A) occupy the same subpocket as the 2-ethoxypiperi-dine moiety of RAL (**6**), substituents in the 2-position point toward a different region of the active site, which is also the binding site of the substrate analogue **7** (Figure 4C).

**Figure 5:**
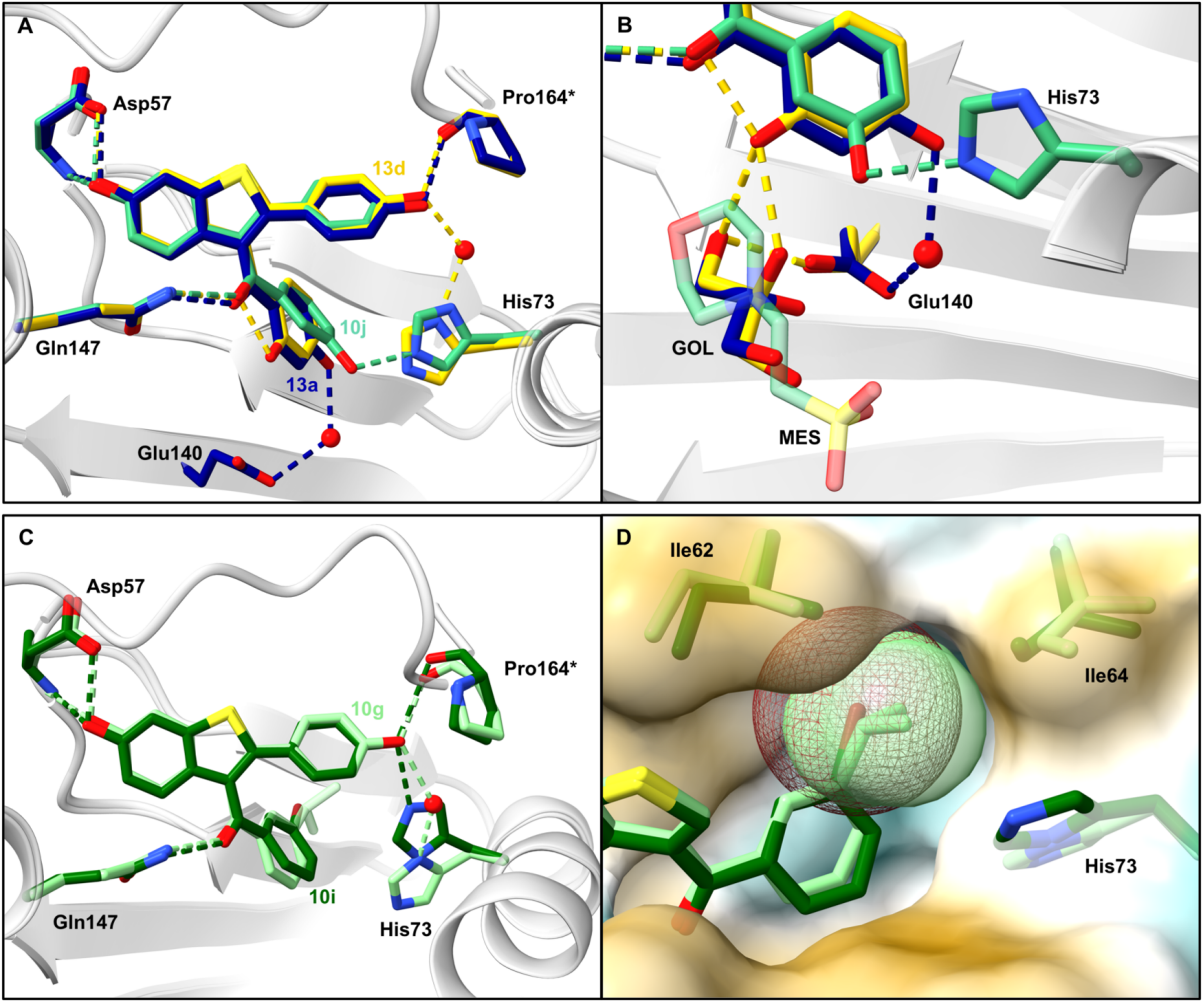
X-ray cocrystal structures of **10g** (pale green, PDB: 9F8K), **10i** (dark green, PDB: 9F8J), **10j** (cyan, PDB: 9F8L), **13a** (dark blue, PDB: 9F8P), and **13d** (yellow, PDB: 9F8Q) bound to *Bc*PhzB (gray). Hydrogen bonds and side chains of the protein are colored in the same color as the respective ligand. **A**. Comparison of the binding modes of **10j**, **13a**, and **13d**. **10j** and **13a** form new interactions with His73 and Glu140, respectively. The 2′-hydroxy group of **13d** forms an intramolecular hydrogen bond to the carbonyl oxygen. **B**. Close up on the interactions of **10j**, **13a**, and **13d** with the protein, the buffer molecule MES, and glycerol. **C**. Comparison of the binding mode of **10g** and **10i**. **D**. Van der Waals surface of the isopropyl moiety of **10g** (pale green) and the bromine atom of **10i** (brown) occupying a hydrophobic binding pocket of *Bc*PhzB (surface, hydrophilic: blue, hydrophobic: yellow).

In addition to the ligands, the buffer molecules glycerol (GOL) and 2-morpholinoethanesulfonic acid (MES) crystallized inside the active site occupying the remaining free space and forming a complex hydrogen bond network (Figure 5B, Figure S14). The binding pose of the ligand’s core scaffold was observed in two positions, in one of which the 2-phenyl moiety is shifted upward and the 3-benzoyl moiety is rotated compared to RAL (**6**) and **11a** (Figure 4A,B). Binding of MES was associated with this rotation as seen in **10j** and **10i** (Figures 4A, 5A/B). To prevent a steric clash with the downward-shifted 2-phenyl moiety, the Trp76 in **11a** flips, constituting an alternative rotamer without affecting the hydrogen bonds with Asp57, His73, Gln147, and Pro164* (Figure 4B).

MES binds via salt bridges from the sulfonic acid to Arg38 and Arg41 and forms a hydrogen bond to Ser77 (Figure S14). Based on these interactions and supported by docking studies (Figure S15), an analogue with a 2′-substituent extended with a morpholine ring (**11g**) targeting Arg38 and Ser77 was designed. However, in the cocrystal structure, the morpholine nitrogen of **11g** forms a hydrogen bond to Glu140 and thereby shifts the core scaffold upward (Figure 4D,E). The hydroxy groups in the 3- and 4-positions of the 3-benzoyl moiety (**10j**, **13a**) form a new direct hydrogen bond to His73 and a water-mediated hydrogen bond to Glu140, respectively (Figure 5A/B).

**10g** and **10i** revealed binding of their lipophilic substituents (isopropyl and bromine) in the 3-position of the 3-benzoyl moiety in a hydrophobic binding pocket formed by Ile62 and Ile64 (Figure 5C/D).

Although initial results suggested that the cross-monomer binding of Pro164* by the 4′-hydroxy group was an important factor for ligand affinity, the large distance between the donor–acceptor-pair in some monomers (*e*.*g*., **10d/i**) exceeded the range for hydrogen bonds and suggested a weaker contribution to binding. This indicates that the 4′-hydroxy group at the 2-phenylbenzo[*b*]thiophene scaffold may not be essential for *Bc*PhzB binding. This hypothesis was pursued further with series D, as it additionally provided a rationale for reducing off-target binding to hER-α.

### Pyocyanin Assay

The compounds’ potential to reduce pyocyanin production in *P. aeruginosa* was evaluated using a whole-cell assay with the highly virulent strain *P. aeruginosa* PA14. Pyocyanin production was measured using a previously described protocol with slight modifications.^54^

**Figure 6:**
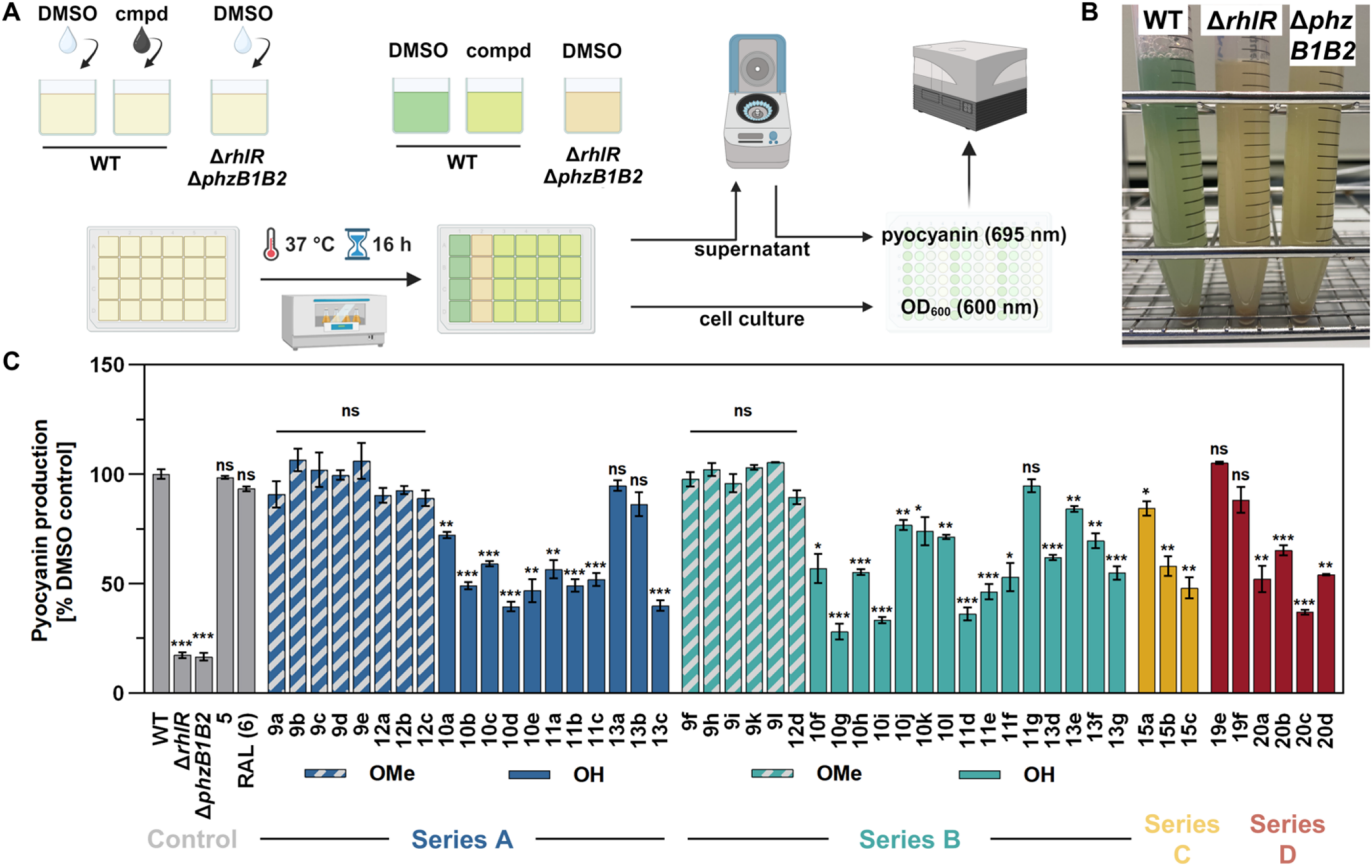
Photometric quantification of pyocyanin in planctonic *P. aeruginosa* PA14 cell cultures. **A**. Experimental setup of photometric pyocyanin determination after incubation of *P. aeruginosa* PA14 wild type (WT) with test compounds. Pyocyanin-deficient mutants lacking *rhlR* (Δ*rhlR*) or *phzB1* and *phzB2* (Δ*phzB1B2*) were used as the controls. **B**. Cell cultures of different *P. aeruginosa* PA14 strains. The characteristic blue-greenish pyocyanin coloring in the WT supernatant is absent in the mutants. **C**. Remaining pyocyanin production of mutants or WT incubated with test compounds at the 10 μM concentration level (*O*Me: 6,4′-dimethoxy, OH: 6,4′-dihydroxy) relative to the WT DMSO control. Reported is the mean ± SEM (*n* = 3). Statistically significant differences in pyocyanin production between WT treated with DMSO versus WT treated with test compounds are indicated with asterisks (ns: not significant, *p < 0.05, **p < 0.01, ***p < 0.001).

In brief, *P. aeruginosa* PA14 was incubated with the respective ligands for 16 h under aerobic conditions. Pyocyanin production was photometrically determined in the cultures’ supernatant at 695 nm and normalized for cell density at 600 nm. Pyocyanin-deficient mutants lacking the QS receptor RhlR (Δ*rhlR*) or the target (Δ*phzB1B2*) were used as the control (Figure 6A,B). Initially, all compounds were screened at a concentration of 10 μM (Figure 6C). Furthermore, IC_50_ values were determined for compounds inhibiting pyocyanin production at this concentration (Table 1, Figure S16).

**Table 1:**
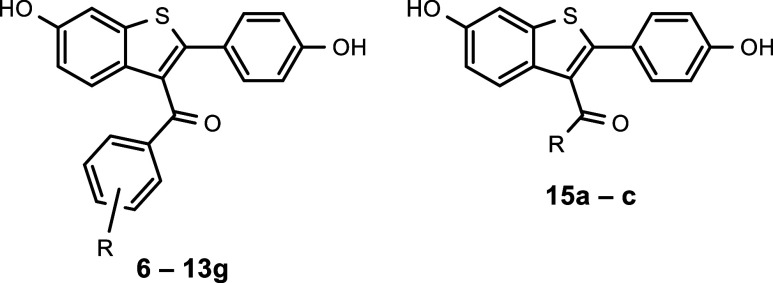
IC_50_ Values from a Photometric Quantification of Pyocyanin in Planctonic *P. aeruginosa* PA14 Cell Cultures and Kinetic Solubility

cmpd	R	IC_50_ [μM] PYO^a^	*S*_kin_ [μM]^b^
RAL (**6**)	4-(2-piperidin-1-ylethoxy)-	55.6 ± 6.0^c^	7.5 ± 0.4
**10a**	-H	15.6 ± 4.0	15.0 ± 1.6
**10b**	4-methyl-	5.4 ± 1.4	n.d.
**10c**	4-fluoro-	10.0 ± 1.6	n.d.
**10d**	4-chloro-	1.7 ± 0.4	13.0 ± 1.1
**10e**	4-bromo-	2.6 ± 0.4	n.d.
**10f**	3-methyl-	7.2 ± 1.3	n.d.
**10g**	3-isopropyl-	0.9 ± 0.1	7.7 ± 0.9
**10h**	3-fluoro-	28.3 ± 3.5^c^	14.6 ± 0.9
**10i**	3-bromo-	2.6 ± 0.7	7.3 ± 0.2
**10j**	3-hydroxy-	127 ± 13^c^	70.7 ± 4.2
**10k**	2-methyl-	29.9 ± 5.9	n.d.
**10l**	2-fluoro-	30.7 ± 6.1	18.7 ± 1.4
**11a**	4-(propylamino)-	3.1 ± 0.7	7.0 ± 0.8
**11b**	4-(isobutylamino)-	3.9 ± 0.6	8.5 ± 2.5
**11c**	4-(pentylamino)-	3.0 ± 0.5	5.5 ± 2.1
**11d**	2-(propylamino)-	6.1 ± 0.6^c^	5.8 ± 1.0
**11e**	2-(isobutylamino)-	14.1 ± 2.9^c^	3.9 ± 0.8
**11f**	2-(pentylamino)-	6.9 ± 1.2	3.6 ± 0.8
**11g**	2-[(2-morpholino-ethyl)amino]-	122 ± 10^c^	4.5 ± 0.5
**13a**	4-hydroxy-	21.7 ± 3.7^c^	105 ± 7.7
**13c**	4-pentoxy-	2.8 ± 0.7	8.3 ± 1.8
**13d**	2-hydroxy-	30.6 ± 5.1^c^	12.9 ± 0.2
**13e**	2-methoxy-	54.6 ± 4.5^c^	22.1 ± 1.1
**13f**	2-isobutoxy-	20.7 ± 3.5	8.0 ± 0.7
**13g**	2-pentoxy-	19.1 ± 2.0^c^	5.9 ± 1.1
**15a**	cyclopentyl-	18.1 ± 2.1	18.1 ± 3.7
**15b**	cyclohexyl-	7.6 ± 0.9	18.4 ± 0.8
**15c**	pentyl-	5.3 ± 0.9	18.6 ± 0.5

a Reported is the mean ± SEM (*n* ≥ 3) (Figure S16).

b Reported is the mean ± SD (triplicate measurements).

c Since the maximum inhibitory effect could not be determined, the regression was performed using the pyocyanin level of the Δ*phzB1B2* mutant as a constraint and an approximate IC_50_ value ± SEM is reported. n.d. = not determined.

Since PhzB inhibitors are developed as pathoblockers, they should not affect cell growth or density. In line with this, our PhzB inhibitors did not affect the cell density (OD_600_) at 100 μM concentration (Figure S17).

**Figure 7:**
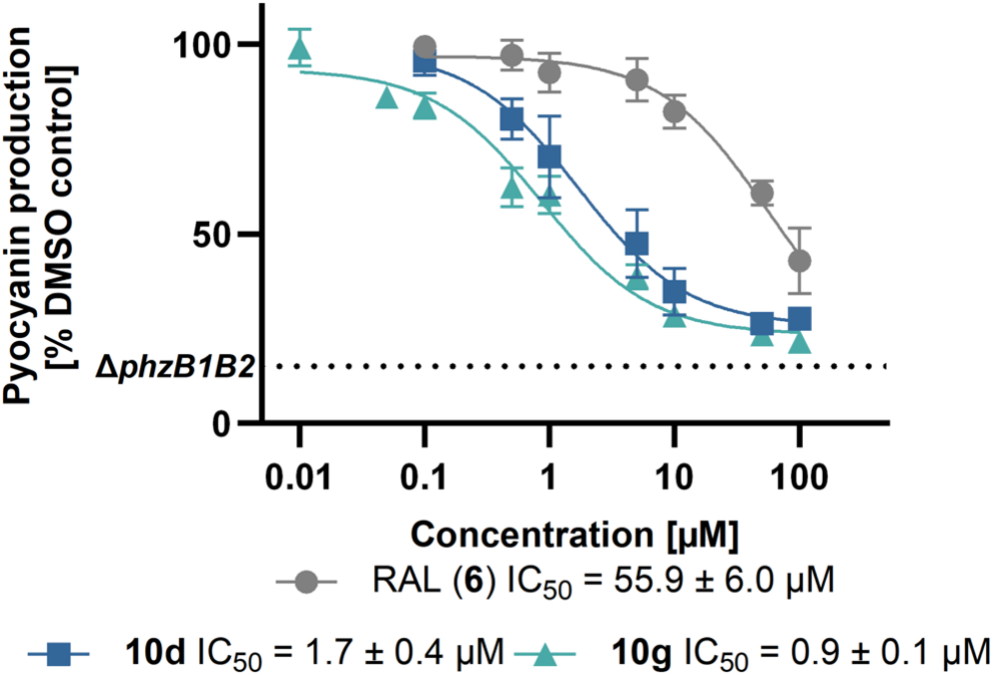
Dose–response curves from a photometric quantification of pyocyanin in planctonic *P. aeruginosa* PA14 cell cultures for RAL (**6**) and its analogues **10d** and **10g**.

Despite its high affinity (≈100 nM, Figure S3), the known PhzB inhibitor **5** did not reduce pyocyanin levels at a concentration of 10 μM. The same holds true for RAL (**6**), which is consistent with earlier results of Ho Sui et al., who employed concentrations higher than 100 μM to evoke significant effects on pyocyanin production.^8^ In our hands, a dose-dependent reduction of pyocyanin production was also observed at high concentrations, but was limited by the solubility of RAL (**6**), such that the maximum inhibitory effect could not be determined. However, the obtained dose–response curve indicates an IC_50_ of approximately 50 μM (Figure 7, Table 1). In good agreement with the docking results, *n*DSF measurements, and cocrystallization studies, none of the dimethoxy precursors reduced pyocyanin production (Figure 6C). In contrast, most of the dihydroxy compounds were active at 10 μM.

Replacing the 4′-(2-piperidinoethoxy) moiety of RAL (**6**) with a hydrogen atom (**10a**) led to an increased effect on pyocyanin levels with an IC_50_ of 15.6 μM. Potency was further enhanced by modifying the 4-position of the benzoyl moiety with lipophilic substituents such as halogens or a pentoxy moiety, resulting in the most active derivatives of series A, *i*. *e*., **10d** (IC_50_ = 1.7 μM, Figure 7) and **13c** (IC_50_ = 2.8 μM). In contrast to **13c**, the 4-hydroxy (**13a**) and 4-isobutyloxy (**13b**) derivatives did not reduce pyocyanin production. All 4′-alkyl amines **11a**–**c** were active with IC_50_ values between 3–4 μM (Table 1).

Derivatization in the 2- and 3-positions of the 3-benzoyl moiety (series B) maintained activity at 10 μM. Substituents such as fluorine (**10h/l**) or methyl (**10f/k**) in these positions barely affected the potency compared to **10a**. Only **10l** displayed a slight decrease in potency, which is in good agreement with the higher *K*_d_ obtained by ITC. Substitution with a hydroxy group was tolerated in the 2′-position (**13d**) but not in the 3′-position (**10j**), although affinities of these compounds are comparable. Converting the hydroxy group of **13d** to a methoxy substituent (**13e**) reduced the potency. This effect declines with increasing alkyl chain length (**13f**–**g**). In contrast, potency is improved by attaching the alkyl chains via a nitrogen atom to the 2-position of the benzoyl moiety (**11d**–**f**). Bulky lipophilic substituents (isopropyl and bromine, **10g** and **10i**) in 3′-position were favorable and led to the best potencies within series B (**10g:** IC_50_ = 0.9 μM, **10i:** IC_50_ = 2.6 μM, Table 1).

To corroborate that the observed reduction in pyocyanin levels is a consequence of PhzB inhibition and not due to interference with the production of alkylquinolones as part of the phenazine biosynthesis-regulating quorum sensing system PQS, we investigated the effects of our most promising PhzB inhibitors (**10d**, **10g**, **10i**, **20c**) on alkylquinolone production in *P. aeruginosa*. By liquid chromatography and tandem mass spectrometry (LC-MS/MS) quantification, we showed that our PhzB inhibitors do not decrease alkylquinolone levels, within the concentration range of their whole-cell effects on pyocyanin biosynthesis (Figure S18), thereby providing further evidence that our RAL analogues reduce pyocyanin biosynthesis by PhzB inhibition.

In summary, most compounds of series A and B inhibit pyocyanin production in the low micromolar concentration range with the best compounds exhibiting IC_50_ values of 1.6 μM (**10d**) and 0.9 μM (**10g**), respectively (Figure 7, Table 1).

### Kinetic Solubility

The solubility displays an important parameter both in early (*e*.*g*., binding assays) and later stages of development such as *in vivo* studies. Therefore, the kinetic solubility of a representative set of compounds was determined in saline phosphate buffer (pH = 7.4). The unsubstituted 3-benzoyl derivative **10a** has a slightly higher solubility of 15.0 μM compared to RAL (**6**, 7.5 μM). For most of the benzoyl substituted compounds, the determined kinetic solubility is either comparable to **10a** (**10d**/**h**/**l**, **13d**/**e**) or RAL (**6**) (**10g**/**i**, **11a**–**e**, **13c**/**f**/**g**) (Table 1). Derivatization of the 3-benzoyl group with hydrophilic moieties (**10j**, **13a**) improved the solubility up to 100 μM. However, this enhanced solubility was associated with decreased potency in pyocyanin reduction in *P. aeruginosa* (Table 1). The hydrophilic moieties in **11g** and **13d** did not improve the solubility. The observed increase in solubility, due to the exchange of the hydroxy group in **13d** with a methoxy group (**13e**) can be explained by the loss of an intramolecular hydrogen bond to the carbonyl oxygen in **13d**. Similar to **10j** and **13a**, these derivatives displayed a decreased potency in pyocyanin reduction.

In order to improve the kinetic solubility, we aimed to increase the Fsp^3^ by replacing the 3-benzoyl moiety with 3-alkanoyl groups (series C). All aliphatic derivatives **15a**–**c** exhibited pyocyanin reduction when tested at a concentration of 10 μM (Figure 6C). The cyclopentyl derivative **15a** displayed similar potency to **10a**, consistent with the bioisosteric exchange of the phenyl ring. The potency of the compounds was further increased by extending the ring system by one carbon atom to form the cyclohexyl derivative **15b** or by opening the ring system (**15c**) (Table 1). However, the aliphatic replacement of the phenyl ring of the 3-benzoyl substituent only slightly increased the kinetic solubility (Table 1).

### Affinity toward hER

Due to the nanomolar affinity of RAL (**6**) to hER-α and the high structural similarity of the synthesized PhzB inhibitors to their parent compound, it is most likely that especially compounds of series A show off-target binding to hER-α. The binding of RAL (**6**) to hER-α is mainly mediated by the 6- and 4′-hydroxy groups of the 2-phenylbenzo[*b*]thiophene scaffold (Figure 2B).^51^ Therefore, ligands lacking one or both hydroxy groups on the core heterocycle (**20a–d**) were designed based on congeners of series A and B (**10d** and **10i**, Figure 2C).

**Figure 8:**
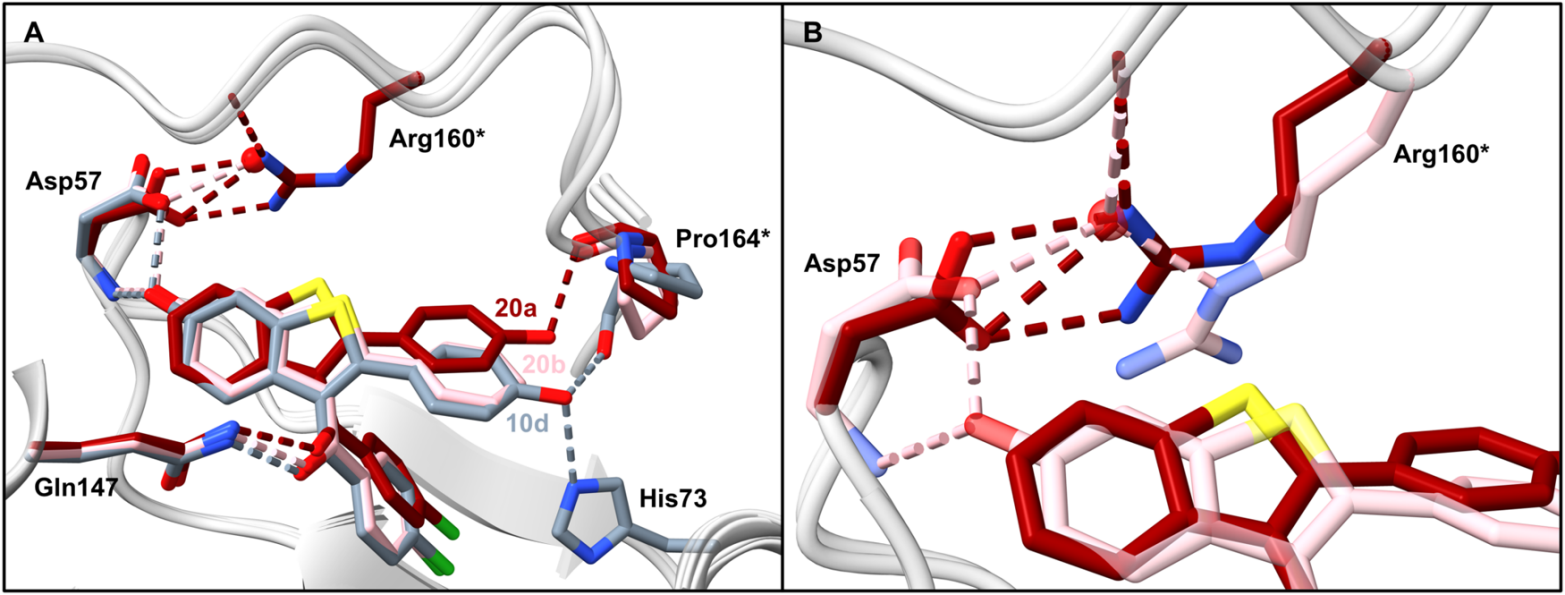
X-ray cocrystal structures of **10d** (gray, PDB: 9F8I), **20a** (dark red, PDB: 9F8R), and **20b** (pink, PDB: 9F8S) bound to *Bc*PhzB (light gray). Hydrogen bonds and side chains of the protein are colored in the same color as the respective ligand. **A**. Comparison of the binding modes of **10d**, **20a**, and **20b**. **B**. Salt bridge formation of Asp57 and Arg160* in the cocrystal structure of **20a** in comparison to the cocrystal structure of **20b** bound to *Bc*PhzB.

Subsequently, cocrystal structures of *Bc*PhzB in complex with **10d**, **20a**, and **20b** were obtained. The binding mode of **20a/b** differs slightly from their dihydroxy analogue **10d**. As expected, the lacking 4′-hydroxy group in **20b** does not allow interactions with Pro164* and His73. **20a** interacts with Pro164* via its 4′-hydroxy group but not with Asp57 due to its missing 6-hydroxy group (Figure 8A). Thus, the Asp57 side chain rotates to form a salt bridge with Arg160* (Figure 8B). Formation of this salt bridge may additionally stabilize the C-terminus but was not observed in cocrystal structures with dihydroxy ligands due to the interaction of the 6-hydroxy group with Asp57.

Simultaneous substitution of the two 6- and 4′-hydroxy groups (**19e**–**f**) with hydrogen resulted in a complete loss of pyocyanin inhibition, indicating that at least one hydroxy group is essential for binding to PhzB (Figure 6C). Comparing the pyocyanin reduction potency of the dihydroxy compounds **10d** and **10i** with their respective monohydroxy analogues supports a greater contribution of the 4′-hydroxy group to potency than the 6-hydroxy group (Table 2). Thus, substitution of the 4′-hydroxy group reduces potency by a factor of 1.3 to 2.5, whereas substitution of the 6-hydroxy group results in a loss in potency of approximately 3- to 6-fold.

**Table 2:**
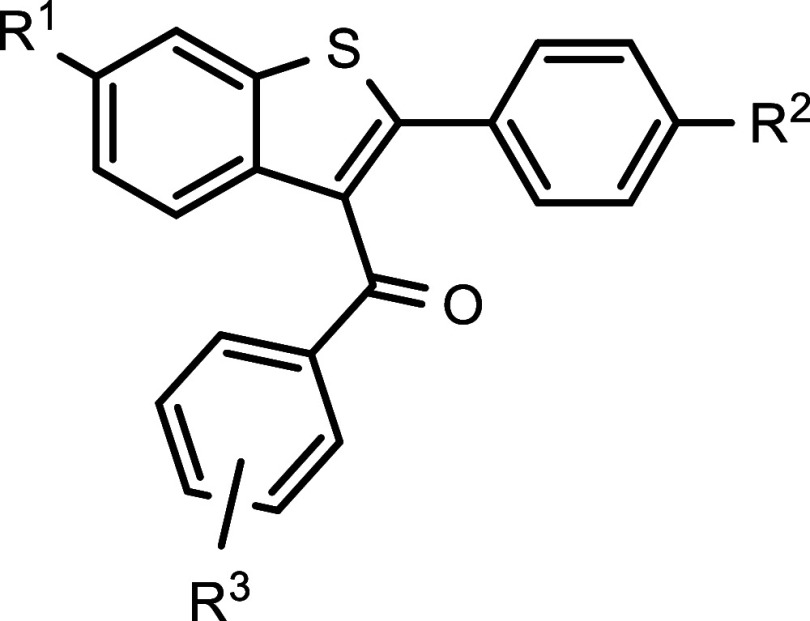
Effects on Pyocyanin Production in *P. aeruginosa* PA14 Cell Cultures and Affinity to hER-α of Series D

cmpd	R^1^	R^2^	R^3^	IC_50_ [nM] PYO^a^	IC_50_ [nM] hER-α^d^
RAL (**6**)	OH	OH	4-(2-piperidin-1-ylethoxy)-	55.6 × 10^3^ ± 6.0 × 10^3^^b^	1.44 ± 0.10
**10d**	OH	OH	4-chloro-	1.7 × 10^3^ ± 0.4 × 10^3^	0.18 ± 0.02
**10i**	OH	OH	3-bromo-	2.6 × 10^3^ ± 0.7 × 10^3^	0.25 ± 0.03
**19e**	H	H	4-chloro-	inactive^c^	n.d.
**19f**	H	H	3-bromo-	inactive^c^	n.d.
**20a**	H	OH	4-chloro-	4.2 × 10^3^ ± 0.7 × 10^3^	39.6 ± 6.3
**20b**	OH	H	4-chloro-	13.4 × 10^3^ ± 1.7 × 10^3^	1.9 ± 0.27
**20c**	H	OH	3-bromo-	3.2 × 10^3^ ± 0.3 × 10^3^	82.3 ± 9.9
**20d**	OH	H	3-bromo-	7.8 × 10^3^ ± 1.1 × 10^3^	13.3 ± 1.7

a Reported is the mean ± SEM (*n* ≥ 3).

b Since the maximum inhibitory effect could not be determined, the regression was performed using the pyocyanin level of the Δ*phzB1B2* mutant as a constraint, and an approximately IC_50_ value ± SEM is reported.

c No pyocyanin reduction observed at 200 μM concentration.

d Reported is the mean ± SEM (*n* = 4, except **20a:***n* = 2). Binding curves are shown in Figure S19.

This observation contrasts with the importance of the two hydroxyl groups for binding to hER-α, as reported by Grese et al.^52^ To test whether this strategy could be used for reducing off-target binding to hER-α, the relative hER-α binding affinity was determined using a competitive time-resolved Förster resonance energy transfer (TR-FRET) assay. Hence, selected members of series A, B, and C were initially screened for binding to hER-α at 1 μM. All tested compounds substituted in 2′-, 3′-, and 4′-position of the 3-benzoyl moiety were found to be hER-α binders. This contradicts the initial hypothesis that the introduction of steric bulk in these positions (series B) may prevent binding to the receptor. We therefore focused on series D to reduce off-target affinity to hER-α.

**10d** and **10i** show 3- to 10-fold higher affinity to hER-α than their parent compound RAL (**6**). Starting with these high-affinity ligands, we characterized the importance of the hydroxy groups in the 6- and 4′-positions of the 2-phenylbenzo[*b*]thiophene scaffold for binding to hER-α and potency in pyocyanin assay, respectively (Table 2, Figure S19). A decrease in affinity toward hER-α was observed by replacing one hydroxy group, either in 6- or in 4′-positions of the parent scaffold.

While all monohydroxy derivatives displayed reduced binding to the hER-α compared to RAL (**6**) and the dihydroxy analogues, the 6-hydroxy group (**20b**/**d**) proved to be more important for binding to hER-α than the 4′-hydroxy group (**20a**/**c**). Replacement of the 4′-hydroxy group reduced hER-α affinity by approximately 10–20-fold, whereas replacement of the 6-hydroxy group resulted in 200–300-fold reduced hER-α binding, with **20c** exhibiting the lowest off-target affinity (Table 2).

### Evaluation of Cytotoxicity

RAL (**6**), the parent compound of this study, is known to exhibit cytotoxicity across various cell lines including HepG2 cells, most likely through an aryl hydrocarbon receptor (AhR)-mediated process.^55^ Despite these cytotoxic side effects, RAL (**6**) possesses an acceptable benefit-risk profile that enabled its approval for long-term medication against postmenopausal osteoporosis. To assess the cytotoxicity of our most promising RAL analogues (**10d**, **10g**, **20c**), we performed an MTT assay with HepG2 cells and observed that our compounds display cytotoxic effects (**10d**: IC_50_ = 42.3 μM, **10g**: IC_50_ = 26.0 μM, **20c**: IC_50_ = 29.4 μM, see Figure S20) in a similar range as compared to the reported data for RAL (**6**, IC_50_ ≈ 50 μM).^56^ In this context, it is important to mention that our one-digit micromolar to the submicromolar inhibitors of pyocyanin biosynthesis (*e*.*g*., **10d**, **10g**, **20c**) did not affect the cell density of *P. aeruginosa* at a concentration of 100 μM (Figure S17), thus highlighting our PhzB inhibitors as pathoblockers.

## Discussion

As predicted *in silico* by Ho Sui et al.,^8^ we confirmed binding of RAL (**6**) to the pyocyanin biosynthesis enzyme PhzB by *n*DSF and cocrystallization. This links the observed pyocyanin reduction by high RAL (**6**) concentrations to its binding to PhzB. Using the *Bc*PhzB/RAL (**6**) cocrystal structure as a starting point, we developed the first PhzB inhibitors that evoked low micromolar to submicromolar inhibition of pyocyanin biosynthesis in *P. aeruginosa* cell culture. Evidence for direct reduction of pyocyanin biosynthesis via inhibition of PhzB was obtained by (i) confirmation of the compounds’ interaction with PhzB by *n*DSF and ITC as well as by elucidation of the binding modes with cocrystal structures; (ii) reduction in pyocyanin production to the level of the PhzB deletion mutant (Δ*phzB1B2*); (iii) the observation that the nonbinding methoxy derivatives also showed no effect on pyocyanin formation and (iv) demonstrating that the most promising PhzB inhibitors do not inhibit the PQS quorum sensing alkylquinolones. Importantly, our pyocyanin biosynthesis inhibitors did not affect the cell density of *P. aeruginosa*, thus highlighting them as pathoblockers that directly interfere with phenazine biosynthesis.

The obtained structural data for the binding mode of 12 ligands provided deep insights into key interactions to Asp57, Gln147, and Pro164* and possible vectors for ligand design: modifications of the benzoyl moiety in the 2-, 3-, and 4-positions were tolerated with a wide variety of substituents such as halogens, aliphatic amines, ethers, and hydroxy groups. These substituents occupied three different subpockets: a small lipophilic pocket in the 3-position, the substrate binding pocket in the 2-position and a large pocket in the 4-position of the 3-benzoyl moiety. A complete occupation of the lipophilic pocket in the 3-position was achieved by **10g** resulting in the highest potency of 0.9 μM. Regarding modifications of the 2-phenylbenzo[*b*]thiophene scaffold, we demonstrated that the 6,4′-dimethoxy derivatives **9a**–**l** neither bind to *Bc*PhzB *in vitro* nor reduce pyocyanin biosynthesis in *P. aeruginosa* cell culture. This is supported by docking studies and the obtained cocrystal structures, which indicate limited space in the 6-position of this scaffold. Taking into account the inactivity of compounds **19e/f**, lacking both aromatic hydroxy groups at the 2-phenylbenzo[*b*]thiophene scaffold, this indicates the importance of at least one hydrogen bond donor to interact with the side chain of Asp57 or the carbonyl backbone of Pro164*.

Interestingly, most of our compounds demonstrated whole-cell activity in *P. aeruginosa* despite violating the rules for porin-dependent uptake in Gram-negative bacteria (eNTRy rules)^57^ and, more importantly, the recently postulated *P. aeruginosa*-specific self-promoted entry (PASsagE) rules,^58,59^ due to their low polarity and missing formal positive charge (see Table S3). On the contrary, although **6** and **11g** display a positive formal charge, they displayed lower potency in pyocyanin reduction in *P. aeruginosa* in comparison to derivatives without aliphatic amines. Since the substitution of RAL’s 4′-(2-piperidinoethoxy) residue with a hydrogen atom (**10a**) was sufficient to significantly increase the activity, it can also be assumed that membrane permeation is restricted in the case of RAL (**6**) and **11g**. This could also be the case for the high-affinity PhzB binder **5**, which displayed no pyocyanin reduction probably due to its dicarboxylic acid–based scaffold, which can be deprotonated under physiological conditions, leading to negatively charged species with poor pharmacokinetics. In line with these findings, the hydroxybenzoyl derivatives **10j**/**13a**/**13d** exhibited declined potency depending on the position of the hydroxy group. We suggest that **10j** (*m*-hydroxy) and **13a** (*p*-hydroxy) also display considerable acidity and that ionization might be sufficient to lower membrane permeability and render the ligands less active. In contrast, **13d** (*o*-hydroxy) probably shows significantly less acidity due to an intramolecular hydrogen bond which keeps the phenol proton in place, enabling the neutral form of **13d** to enter the bacterial cell. This intramolecular hydrogen bond of **13d** was also verified by the cocrystal structure of the ligand with *Bc*PhzB. These findings suggest that the cellular uptake of our PhzB inhibitors may expand the known rules for small molecule uptake in *P. aeruginosa*. Further investigation of the underlaying uptake mechanisms could help to refine the uptake rules for *P. aeruginosa* and deepen our understanding of compound accumulation in this pathogen.

To optimize RAL-derived compounds as effective pathoblockers, we aimed to improve their physicochemical parameters. While RAL (**6**) exhibits adequate solubility and pharmacokinetic characteristics for its current application in osteoporosis treatment, these properties are likely insufficient for its application as a pathoblocker. Due to the low oral bioavailability and high plasma protein binding of RAL (**6**), only nanomolar *C*_max_ levels are obtained,^60−62^ which are 100- to 1000-fold lower than its IC_50_ value of 55.6 μM in our whole-cell pyocyanin quantification assay. Consequently, achieving therapeutic efficacy as a pathoblocker will most likely require improved physicochemical and pharmacokinetic properties. While the structure-based approach to improve the on-target affinity and potency of our RAL-derived PhzB inhibitors was successful (*e*.*g*., **10d**, **10g**, **20c**), attempts to increase solubility have so far led to less satisfactory results, since increased solubility, achieved by incorporating hydrophilic moieties, was found to result in decreased activity in whole-cell assays (*e*.*g*., **10j**). An alternative strategy for improving the physicochemical properties by increasing Fsp^3^ yielded compounds with one-digit micromolar IC_50_ values in whole-cell assays, however, did not result in improved solubility (*e*.*g*., **15b**, **15c**). Encouragingly, the new structural insights gained by various cocrystal structures will be highly valuable for future optimization of both pharmacodynamic and pharmacokinetic parameters and, together with the recently reported Hergenrother’s PASsagE rules for small molecule uptake in *P. aeruginosa*,^58^ can serve as a basis for the development of next-generation PhzB inhibitors.

Since RAL (**6**) is optimized for binding to hER-α, affinity to this target should be minimized to repurpose RAL analogues as PhzB inhibitors. To this end, we investigated different approaches to reduce hER-α affinity. The introduction of steric bulk in 2- and 3-positions of the 3-benzoyl moiety did not lead to reduced binding at the off-target, possibly because the helices around the entrance of the hER-α’s binding pocket display a certain amount of flexibility. Removal of the 6-hydroxy group provided a useful strategy for reducing affinity to hER-α, as **20a** and **20c** showed up to 200-fold reduced hER-α binding with only marginally reduced pyocyanin production (1.3–2.5-fold), compared to their dihydroxy analogues **10d** and **10i**. We acknowledge that hER-α affinity of our most promising PhzB inhibitor **20c** remains at the nanomolar level, which is considerably higher than its activity in the pyocyanin quantification assay. Hence, both on- and off-target affinity should be further improved in future studies. Together with the insights from the cocrystal structures presented here, the monohydroxy compound **20c** offers a promising starting point for future investigations.

Another limitation of our presented series of PhzB inhibitors is their cytotoxicity in HepG2 cells, which occurs at approximately 9–25-fold higher concentrations than the inhibition of pyocyanin synthesis in *P. aeruginosa*. Given the fact that our compounds are at an early stage as new therapeutics for short-term treatment of *P. aeruginosa* infections, we are convinced that the observed cytotoxicity, which is similar to the approved drug RAL (**6**), is a point that should definitively be addressed in future studies but represents no ‘red flag’ at the current stage.

## Conclusions and Outlook

In this study, we present a new class of raloxifene (RAL, **6**)-derived PhzB inhibitors as the first phenazine biosynthesis inhibitors that show promising whole-cell activity in *P. aeruginosa*, which is often a limiting factor for compounds that target this pathogen. Compared to the parent compound RAL (**6**), our PhzB inhibitors possess significantly (up to 50-fold) improved inhibition of the biosynthesis of pyocyanin—a well-known virulence factor of *P. aeruginosa*. Importantly, these inhibitors did not affect the cell density of *P. aeruginosa*, thus highlighting them as potential pathoblockers. Interestingly, our compounds displayed activity in *P. aeruginosa* cell culture despite violating the recently postulated PASsagE (*Pseudomonas aeruginosa* Self-promoted Entry) rules, thus suggesting that they may expand the known rules for small molecule uptake in *P. aeruginosa*. Given the current limitations regarding their remaining off-target affinity for hER-α, future studies will be directed toward gaining on-target selectivity for PhzB. A basis for such investigations will be the structure-guided approaches established in this study. Future work may also assess the impact of RAL-derived inhibitors on transcription in *P. aeruginosa*, not only because they may interfere directly with transcription but also because pyocyanin is known to regulate the activity of transcription factor SoxR.^28^

In summary, our work underscores the suitability of PhzB as a drug target to block the biosynthesis of the *P. aeruginosa* virulence factor pyocyanin. The presented PhzB inhibitors, featuring activity in cell culture in the one-digit micromolar to the submicromolar range, together with the comprehensive structural insights into PhzB–ligand interactions obtained by 12 cocrystal structures, will be of great value for the future optimization of the affinity, potency, selectivity and pharmaco-kinetic profile of next-generation PhzB inhibitors as pathoblockers for the treatment of *P. aeruginosa* infections.

## Experimental Section

### General Remarks

Reagents and solvents used were of commercially available reagent grade quality from abcr GmbH (Karlsruhe, Germany), Acros Organics (Geel, Belgum), Alfa Aesar (Kandel, Germany), BLDpharm (Kaiserslautern, Germany), ChemSpace (Riga, Latvia), Sigma-Aldrich (Steinheim, Germany), TCI Germany (Eschborn, Germany) and VWR International (Darmstadt, Germany) and used without purification unless otherwise stated. All nonaqueous reactions requiring anhydrous conditions were carried out in dried flasks and covered with a drying tube filled with calcium chloride. Solvents were dried according to common procedures, if necessary, and stored over 3 Å molecular sieves.^63^ Concentration *in vacuo* refers to the removal of solvent on a rotary evaporator under reduced pressure in a water bath at 40 °C. Celite refers to a Celite 545 filter aid. Brine refers to a saturated aqueous solution of sodium chloride. Petroleum ether refers to the fraction in the boiling point range 40–60 °C.

Reaction monitoring was performed using TLC (Polygram SIL G/UV254, 0.2 mm silica gel 60, 40 × 80 mm, Macherey-Nagel, Düren, Germany), visualization by UV light (254 nm, 366 nm). Silica gel (40–63 μm) was used for purification by column chromatography. Yields were not optimized.

Preparative HPLC was carried out with a LaPrep system (Merck, Darmstadt, Germany): LaPrep P110 pump, sample loop (Knauer), LaPrep P216 fraction collector, LaPrep P311 UV/vis detector, and a LiChrospher 100 RP-18 column (12 μm, 100 Å, 25 × 125 mm, self-fill level NW25). The compound was dissolved in DMSO and the stated eluent (MeCN/H_2_O, 2–5 mL) and injected into the sample loop [isocratic elution with MeCN/H_2_O, 40 mL min^–1^, detection at 254 nm].

For NMR spectra, deuterated solvents, dimethyl sulfoxide (C_2_D_6_OS, Deutero GmbH, 99.8%), or chloroform (CDCl_3_), were used. ^1^H NMR spectra were measured on a Bruker Avance III 400 (400 MHz), Bruker Avance IIIHD 500 (500 MHz), or Bruker Avance II 600 (600 MHz) spectrometer (Bruker BioSpin GmbH, Rheinstetten, Germany) in the stated solvents as a reference for the internal deuterium lock. The chemical shift data for each signal are given as δ in units of parts per million (ppm) relative to tetramethylsilane (TMS) where δ(TMS) = 0.00. The spectra are calibrated using the solvent.^64^ The multiplicity of each signal is indicated by s (singlet); d (doublet); t (triplet); q (quartet); quint (quintet); h (sextet); hept (septet); n (nonet), m (multiplet); or combinations thereof. The number of protons (*n*) for a given resonance signal is indicated by *n*H. Where appropriate, coupling constants (*J*) are quoted in Hz, and recorded to the nearest 0.1 Hz. Spectra were assigned using COSY, HSQC, and HMBC experiments as necessary.

^13^C NMR spectra were measured on a Bruker Avance III 400 (101 MHz), Bruker Avance IIIHD 500 (126 MHz), or Bruker Avance II 600 (150 MHz) spectrometer (Bruker BioSpin GmbH, Rheinstetten, Germany) in the stated solvents as a reference for the internal deuterium lock. The chemical shift data for each signal are given as δ in units of parts per million (ppm) relative TMS where δ(TMS) = 0.00. The spectra are calibrated using the solvent peak.^64^ The chemical shift is quoted to 2 decimal places. Magnetically equivalent C atoms, which gave a single signal, were signed as “*n*C”. Spectra were assigned using DEPT-135, HSQC, and HMBC as necessary.

Mass spectra were acquired on either an Expression^L^ CMS spectrometer (low resolution, MS, Advion, Ithaca, NY, USA) using ESI or atomic pressure chemical ionization (APCI) or LTQ-Orbitrap Velos mass spectrometer (high-resolution mass spectrometry (HRMS), Thermo Fisher Scientific, Bremen, Germany) using ESI from solutions of methanol. *m*/*z* values are reported in Daltons and followed by their percentage abundance in parentheses.

Melting points were determined using an Electrothermal IA9200 tube melting point apparatus (Cole-Parmer Instrument Company Ltd., St. Neots, UK) and are uncorrected. The solvent(s) from which the sample was crystallized is given in parentheses. Formation of gas and/or change of color was interpreted as decomposition and signed with “dec.”.

Elemental analysis was performed on a CE Instruments Flash EA 1112 Elemental Analyzer (Thermo Quest, San Jose, CA, USA). The percentage mass fraction was determined from at least two independent measurements; the mean value is reported.

Infrared spectra were recorded on a Thermo Nicolet FT-IR 200 spectrometer (Thermo Nicolet, Madison, WI, USA) using KBr pellets.

Analytical HPLC: Compounds were dissolved in DMSO (approximately 500 μg in 300–400 μL, ≥99.7%) and 1–10 μL injected to the stated HPLC system. The percentage abundance is calculated after integration using the AUC with the 100%-method. The dead time was determined using the signal of DMSO and the time 0.0–1.6 min was excluded from the integration. Absorption maxima (λ_max_) were extracted from the UV spectra recorded by the diode array detector in the peak maxima during HPLC runs of method 2 and reported for wavelengths above 240 nm. The purity of all biologically tested compounds was ≥95% as determined using the HPLC methods above.

Method 1 was carried out on a Merck Hitachi LaChrom Elite system (Hitachi High Technologies Corporation, Tokyo, Japan) with a L-2130 Pump and a L-2450 diode array detector, with detection at 254 and 280 nm, using a LiChrospher 100, RP-18 (5 μm) LiChroCART 125–4, reverse phase column [isocratic elution with MeCN/H_2_O as stated, 1 mL min^–1^, 40 °C, 10–15 min]. Threshold: 1000.

Method 2 was carried out on an VWR Hitachi Chromaster system (Hitachi High Technologies Corporation, Tokyo, Japan) with a 5110 pump and a 5430 diode array detector, with detection at 254 nm, using a LiChrospher 100, RP-18 (5 μm) LiChroCART 125–4, reverse phase column; [0–2 min: MeCN/H_2_O 10:90, 2–12 min: MeCN/H_2_O 10:90 → MeCN/H_2_O 90:10 (linear), 12–20 min: MeCN/H_2_O 90:10, 1 mL min^–1^, 40 °C]. Sensitivity: 50.

Method 3 was carried out on a Merck Hitachi LaChrom Elite system with a L-2130 Pump and a L-2450 diode array detector, with detection at 254 and 280 nm, using a LiChrospher 100, RP-18 (5 μm) LiChroCART 125–4, reverse phase column; [isocratic elution with MeCN/buffer as stated, 1 mL min^–1^, 40 °C, 10–15 min]. Buffer: triethyl amine (20 mL) was dissolved in water (980 mL) and NaOH (242 mg) was added. The pH was adjusted to 2.7 by the addition of concentrated H_2_SO_4_. Threshold: 1000.

### Syntheses and Compound Characterization

Detailed information on compound syntheses and characterization are given below. NMR spectra for new compounds and HPLC traces for biologically tested compounds are given in the Supporting Information.

#### General Procedure 1 for the Preparation of (6-Methoxy-2-(4-methoxyphenyl)benzo[*b*]thiophen-3-yl)(phenyl)-methanones—*Friedel–Crafts* Acylation (Modified from Ervin et al.)^53^

The reaction is carried out in the absence of moisture and cooled in an ice-bath to 0 °C. The respective benzo[*b*]thiophene (**8/18a**–**c**, 1.0 equiv) is dissolved in CH_2_Cl_2_ and the appropriate carboxylic acid chloride (1–1.7 equiv) is added. Aluminum chloride (1.1–2.2 equiv) is added in portions, turning the reaction mixture deep red. The reaction is stirred while reaching ambient temperature. Progress of the reaction is monitored by TLC and further aluminum chloride and appropriate carboxylic acid chloride were added, if necessary. The reaction is stopped by the addition of 2 M HCl_aq_. The layers are separated, the aqueous layer is extracted with CH_2_Cl_2_ and the combined organic layers are washed as stated, dried with Na_2_SO_4_, filtered, and concentrated *in vacuo*. The crude product is purified by recrystallization (ethanol) and/or silica gel column chromatography using the indicated eluent.

#### General Procedure 2 for the Preparation of (6-Hydroxy-2-(4-hydroxyphenyl)benzo[*b*]thiophen-3-yl)(phenyl)-methanones—*O*-Demethylation with Boron Tribromide (Modified from Campos et al.)^65^

The reaction is carried out in the absence of moisture. The respective methoxy component (**9a**–**l/12a**–**d**, 1.0 equiv) is dissolved in dried CH_2_Cl_2_. Boron tribromide (1 M in CH_2_Cl_2_, 2.0–5.2 equiv) is added turning the solution brown-red. The reaction is stirred at ambient temperature and monitored by TLC. H_2_O is added to terminate the reaction and stirred briefly. The precipitate at the phase boundary is dissolved by the addition of CH_2_Cl_2_ and methanol or EtOAc. The layers are separated and the aqueous layer is extracted with CH_2_Cl_2_ and methanol or EtOAc. The combined organic layers are washed as stated and dried with Na_2_SO_4_, filtered, and concentrated *in vacuo*. The crude product is purified by recrystallization and/or silica gel column chromatography using the indicated eluent.

#### General Procedure 3 for the Preparation of (6-Hydroxy-2-(4-hydroxyphenyl)benzo[*b*]thiophen-3-yl)(phenyl)-methanones—*O*-Demethylation with Pyridinium Hydrochloride (Modified from Ervin et al.)^53^

The respective methoxy compound (**14a–b**/**19b–d**, 1.0 equiv) and pyridinium hydrochloride (20 equiv) are melted under vigorous stirring in a microwave reactor (stepwise heating to 180 °C, ramp to temperature, max. Twenty W, 300 psi, 10 mL vessel). The resulting solid is suspended in 1.3 M HCl_aq_ and extracted with EtOAc. The combined organic layers are washed as stated, dried with Na_2_SO_4_, filtered, and concentrated *in vacuo*. The crude product is purified by silica gel column chromatography using the indicated eluent.

#### General Procedure 4 for the Preparation of (2-Phenylbenzo[*b*]thiophene-3-yl)(alkoxyphenyl)-methanones (Modified from Ervin et al.)^53^

The reaction is carried out under a nitrogen atmosphere. The respective alcohol (5.0–6.0 equiv) is slowly added to a mixture of sodium hydride (60% in mineral oil, 10 equiv) in dried DMF and stirred at ambient temperature for 15 min. The respective fluorophenyl(2-phenylbenzo[*b*]thiophen-3-yl)methanone (**9c/9l/10l**, 1.0 equiv) is dissolved in dried DMF and added to the reaction. The reaction is stirred at ambient temperature or at 50 °C. The reaction is stopped by slow addition of H_2_O. The layers are separated and the aqueous layer is extracted with EtOAc, the combined organic layers are washed as stated, dried with Na_2_SO_4_, filtered, and concentrated *in vacuo*. The crude product is purified by column chromatography with the indicated eluent and/or recrystallized from the indicated solvent.

#### General Procedure 5 for the Preparation of (2-Phenylbenzo[*b*]thiophen-3-yl)(alkylaminophenyl)-methanones (Modified from Ervin et al.)^53^

The respective fluorophenyl[6-hydroxy-2-(4-hydroxyphenyl)benzo[*b*]thiophen-3-yl]methanone (**10c/10l**, 1.0 equiv) and the respective amine (20 equiv) are dissolved in DMSO and stirred at 100 °C. After completion, HCl_aq_ is added, the layers are separated and the aqueous layer is extracted with EtOAc. The combined organic layers are washed with brine, dried with Na_2_SO_4_, filtered, and concentrated *in vacuo*. The product is purified by silica gel column chromatography with the indicated eluent.

#### General Procedure 6 for Suzuki–Miyaura Coupling (Modified from Chirgadze et al.)^66^

The respective benzo[*b*]thiophene-2-boronic acid (1.0–1.1 equiv), potassium carbonate (1.8–2.0 equiv), tetrakis(triphenylphosphine)palladium(0) (0.05 equiv), and the respective aryl halide (1.0–1.1 equiv) are suspended in 1,4-dioxan/H_2_O (1:4 v/v, 20 mL) and heated in a microwave reactor (120 °C, 300 W, 70 psi, 30 min, 35 mL vessel). The precipitated product is obtained by filtration and dissolved in CHCl_3_ or extracted with CHCl_3_, filtered through Celite, washed as stated, dried with Na_2_SO_4_, filtered, and concentrated *in vacuo*. The crude product is purified by silica gel column chromatography using the indicated eluent.

##### [6-Methoxy-2-(4-methoxyphenyl)benzo[*b*]thiophen-3-yl](phenyl)methanone (**9a**)

Following general procedure 1 using 6-methoxy-2-(4-methoxyphenyl)benzo[*b*]thiophene (**8**, 350 mg, 1.30 mmol, 1.0 equiv), benzoyl chloride (180 μL, 1.55 mmol, 1.2 equiv), and aluminum chloride (247 mg, 1.85 mmol, 1.4 equiv) in CH_2_Cl_2_ (20 mL). The reaction was stopped by the addition of 2 M HCl_aq_ (20 mL) after 19 h. Extracted with CH_2_Cl_2_ (3 × 20 mL) and washed with brine, brine/2 M HCl_aq_, and brine (50 mL each). Purification by recrystallization (ethanol, 5 mL) yielded yellow crystals (364 mg, 0.972 mmol, 75%): C_23_H_18_O_3_S (374.45); mp 123–124 °C [lit. 110–111 °C/100–102 °C (methanol)];^67^ IR (KBr) *ṽ* 1635 (C=O), 1253 (Ar–O-Me) cm^–1^; ^1^H NMR (400 MHz, C_2_D_6_OS) δ_H_ 7.72–7.66 (m, 3H), 7.54 (dddd, *J* = 7.4, 7.4, 1.8, 1.8 Hz, 1H), 7.43 (d, *J* = 8.9 Hz, 1H), 7.40–7.34 (m, 2H), 7.32–7.25 (m, 2H), 7.02 (dd, *J* = 8.9, 2.4 Hz, 1H), 6.90–6.81 (m, 2H), 3.85 (s, 3H), 3.69 (s, 3H) ppm; ^13^C NMR (101 MHz, C_2_D_6_OS) δ_C_ 193.75, 159.59, 157.44, 142.69, 139.45, 136.85, 133.67, 133.06, 129.97 (2C), 129.66, 129.36 (2C), 128.71 (2C), 125.10, 123.44, 115.17, 114.33 (2C), 105.18, 55.58, 55.21 ppm; MS (APCI-DI^+^) *m*/*z* [M + H]^+^ 375 (100%); HPLC (Method 1) retention time = 5.8 min, 97.6% (254 nm), 97.4% (280 nm), *t*_M_ (DMSO) = 1.1 min (MeCN/H_2_O 70:30); HPLC (Method 2) retention time = 13.8 min, 99.5%, *t*_M_ (DMSO) = 1.2 min, λ_max_ = 282 nm.

The compound is described in the literature.^67^

##### [6-Methoxy-2-(4-methoxyphenyl)benzo[*b*]thiophen-3-yl](*p*-tolyl)methanone (**9b**)

Following general procedure 1 using 6-methoxy-2-(4-methoxyphenyl)benzo[*b*]thiophene (**8**, 543 mg, 2.01 mmol, 1.0 equiv), 4-methylbenzoyl chloride (320 μL, 2.42 mmol, 1.2 equiv), and aluminum chloride (400 mg, 3.00 mmol, 1.5 equiv) in CH_2_Cl_2_ (20 mL). The reaction was stopped by the addition of 2 M HCl_aq_ (20 mL) after 4.75 h. Extracted with CH_2_Cl_2_ (3 × 20 mL), and washed with brine/2 M HCl_aq_, water, and brine (100 mL each). Purification by silica gel chromatography (*n*-hexane/EtOAc 8:1) yielded pale-yellow crystals (547 mg, 1.41 mmol, 70%): C_24_H_20_O_3_S (388.48); mp 110–113 °C; IR (KBr) ṽ 1638 (C=O), 1251 (Ar–O-Me) cm^–1^; ^1^H NMR (400 MHz, C_2_D_6_OS) δ_H_ 7.67 (d, *J* = 2.4 Hz, 1H), 7.64–7.59 (m, 2H), 7.34 (d, *J* = 8.9 Hz, 1H), 7.32–7.28 (m, 2H), 7.23–7.19 (m, 2H), 7.00 (dd, *J* = 8.9, 2.5 Hz, 1H), 6.91–6.86 (m, 2H), 3.84 (s, 3H), 3.71 (s, 3H), 2.30 (s, 3H) ppm; ^13^C NMR (101 MHz, C_2_D_6_OS) δ_C_ 193.44, 159.52, 157.37, 144.39, 141.47, 139.37, 134.26, 133.09, 129.95, 129.73 (2C), 129.50 (2C), 129.36 (2C), 125.12, 123.28, 115.06, 114.38 (2C), 105.16, 55.54, 55.19, 21.14 ppm; elemental analysis [%] calcd: C 74.20, H 5.19, found: C 74.18, H 5.38; MS (APCI-DI^+^) *m*/*z* [M + H]^+^ 389 (100%); MS (APCI-DI^–^) *m*/*z* [M – H]^−^ 387 (100%); HPLC (Method 1) retention time = 7.1 min, 99.5% (254 nm), 99.9% (280 nm), *t*_M_ (DMSO) = 1.1 min (MeCN/H_2_O 70:30); HPLC (Method 2) retention time = 14.2 min, 99.6%, *t*_M_ (DMSO) = 1.1 min, λ_max_ = 269 nm.

The compound is described in the literature.^68^

##### (4-Fluorophenyl)[6-methoxy-2-(4-methoxyphenyl)benzo[*b*]thiophen-3-yl]methanone (**9c**)

Following general procedure 1 using 6-methoxy-2-(4-methoxyphenyl)benzo[*b*]thiophene (**8**, 266 mg, 0.983 mmol, 1.0 equiv), 4-fluorobenzoyl chloride (130 μL, 1.08 mmol, 1.1 equiv), and aluminum chloride (141 mg, 1.06 mmol, 1.1 equiv) in CH_2_Cl_2_ (20 mL). The reaction was stopped by the addition of 2 M HCl_aq_ (20 mL) after 23 h. Extracted with CH_2_Cl_2_ (3 × 20 mL) and washed with brine/2 M HCl_aq_, water and brine (50 mL each). Purification by silica gel chromatography (petroleum ether/EtOAc 8:1) yielded a pale-yellow solid (349 mg, 0.890 mmol, 91%): C_23_H_17_FO_3_S (392.44); mp 106–108 °C [lit. 108.1–109 °C];^69^ IR (KBr) *ṽ* 1640 (C=O), 1250 (Ar–O-Me) cm^–1^; ^1^H NMR (500 MHz, C_2_D_6_OS) δ_H_ 7.78–7.73 (m, 2H), 7.68 (d, *J* = 2.4 Hz, 1H), 7.47 (dd, *J* = 8.9, 0.5 Hz, 1H), 7.30–7.26 (m, 2H), 7.22–7.17 (m, 2H), 7.04 (dd, *J* = 8.9, 2.4 Hz, 1H), 6.89–6.85 (m, 2H), 3.85 (s, 3H), 3.71 (s, 3H) ppm; ^13^C NMR (126 MHz, C_2_D_6_OS) δ_C_ 192.01, 164.96 (d, ^1^*J*_*CF*_ = 252.9 Hz), 159.55, 157.38, 142.98, 139.39, 133.54 (d, ^4^*J*_*CF*_ = 2.7 Hz), 132.84, 132.29 (d, ^3^*J*_*CF*_ = 9.8 Hz, 2C), 129.96 (2C), 129.25, 124.89, 123.37, 115.70 (d, ^2^*J*_*CF*_ = 22.2 Hz, 2C), 115.12, 114.24 (2C), 105.08, 55.49, 55.14 ppm; MS (APCI-DI^+^) *m*/*z* [M + H]^+^ 393 (100%); MS (APCI-ASAP^–^) *m*/*z* [M-CH_3_]^−^ 377 (100%); HRMS (ESI^+^) *m*/*z* [M + H]^+^ calcd: 393.09552 found: 393.09554, [M + Na]^+^ calcd: 415.07746 found: 415.07758, [M + K]^+^ calcd: 431.05140 found: 431.05137, [2M + Na]^+^ calcd: 807.16570 found: 807.16610; HPLC (Method 1) retention time = 5.9 min, 98.3% (254 nm), 98.0% (280 nm), *t*_M_ (DMSO) = 1.1 min (MeCN/H_2_O 70:30); HPLC (Method 2) retention time = 13.4 min, 99.6%, *t*_M_ (DMSO) = 1.4 min, λ_max_ = 257 nm.

The compound is described in the literature.^53,69^

##### (4-Chlorophenyl)[6-methoxy-2-(4-methoxyphenyl)benzo[*b*]thiophen-3-yl]methanone (**9d**)

Following general procedure 1 using 6-methoxy-2-(4-methoxyphenyl)benzo[*b*]thiophene (**8**, 541 mg, 2.00 mmol, 1 equiv), 4-chlorobenzoyl chloride (310 μL, 2.42 mmol, 1.2 equiv), and aluminum chloride (411 mg, 3.08 mmol, 1.5 equiv) in CH_2_Cl_2_ (25 mL). The reaction was stopped by the addition of 2 M HCl_aq_ (20 mL) after 24 h. Extracted with CH_2_Cl_2_ (3 × 20 mL) and washed with brine/2 M HCl_aq_, water, and brine (100 mL each). Purification by silica gel chromatography (*n*-hexane/EtOAc 8:1) yielded a pale crystalline solid (409 mg, 1.78 mmol, 89%): C_23_H_17_ClO_3_S (408.9); mp 114–115 °C (EtOH) [lit. 53 °C];^70^ IR (KBr) *ṽ* 1639 (C=O), 1251 (Ar–O-Me) cm^–1^; ^1^H NMR (500 MHz, C_2_D_6_OS) δ_H_ 7.70–7.65 (m, 3H), 7.48 (d, *J* = 8.9 Hz, 1H), 7.45–7.41 (m, 2H), 7.30–7.25 (m, 2H), 7.04 (dd, *J* = 8.9, 2.4 Hz, 1H), 6.90–6.84 (m, 2H), 3.85 (s, 3H), 3.71 (s, 3H) ppm; ^13^C NMR (126 MHz, C_2_D_6_OS) δ_C_ 192.30, 159.63, 157.42, 143.46, 139.41, 138.32, 135.55, 132.79, 131.11 (2C), 130.04 (2C), 129.07, 128.74 (2C), 124.85, 123.41, 115.17, 114.28 (2C), 105.10, 55.51, 55.18 ppm; elemental analysis [%] calcd: C 67.56, H 4.19. Found: C 67.87, H 4.18; MS (APCI-DI^+^) *m*/*z* [M + H]^+^ 409 (100%); HPLC (Method 1) retention time = 4.3 min, 98.3% (254 nm), 98.4% (280 nm), *t*_M_ (DMSO) = 1.1 min (MeCN/H_2_O 80:20); HPLC (Method 2) retention time = 14.5 min, 99.1%, *t*_M_ (DMSO) = 1.2 min, λ_max_ = 265 nm.

The compound is described in the literature.^70^

##### (4-Bromophenyl)[6-methoxy-2-(4-methoxyphenyl)benzo[*b*]thiophen-3-yl]methanone (**9e**)

Following general procedure 1 using 6-methoxy-2-(4-methoxyphenyl)benzo[*b*]thiophene (**8**, 272 mg, 1.00 mmol, 1.0 equiv), 4-bromobenzoyl chloride (274 mg, 1.25 mmol, 1.3 equiv), and aluminum chloride (141 mg, 1.06 mmol, 1.1 equiv) in CH_2_Cl_2_ (20 mL). Further addition of 4-bromobenzoyl chloride (219 mg, 1.00 mmol, 1.0 equiv) and aluminum chloride (114 mg, 0.855 mmol, 0.9 equiv) after 21 h. The reaction was stopped by the addition of 2 M HCl_aq_ (10 mL) after 24 h. Extracted with EtOAc (50 mL) and washed with 10% NaHCO_3_ and brine (50 mL each). Purification by silica gel chromatography (*n*-hexane/EtOAc 9:1) and following recrystallization from EtOH (2 mL) yielded a pale-yellow solid (263 mg, 0.580 mmol, 58%): C_23_H_17_BrO_3_S (453.35); mp 129–131 °C [lit. 127–129 °C];^71^ IR (KBr) *ṽ* 1637 (C=O), 1250 (Ar–O-Me) cm^–1^; ^1^H NMR (500 MHz, C_2_D_6_OS) δ_H_ 7.68 (d, *J* = 2.4 Hz, 1H), 7.62–7.55 (m, 4H), 7.48 (dd, *J* = 8.9, 0.5 Hz, 1H), 7.29–7.25 (m, 2H), 7.04 (dd, *J* = 8.9, 2.5 Hz, 1H), 6.89–6.85 (m, 2H), 3.85 (s, 3H), 3.71 (s, 3H) ppm; ^13^C NMR (126 MHz, C_2_D_6_OS) δ_C_ 192.51, 159.62, 157.40, 143.45, 139.39, 135.85, 132.77, 131.68 (2C), 131.17 (2C), 130.02 (2C), 129.03, 127.60, 124.83, 123.40, 115.16, 114.28 (2C), 105.09, 55.49, 55.16 ppm; elemental analysis [%] calcd: C 60.94, H 3.78, found: 60.84, H 3.71; MS (APCI-DI^+^) *m*/*z* [M-269]^+^ 183 (61%), [M + H]^+^ 453 (100%); HPLC (Method 1) retention time = 4.2 min, 98.2% (254 nm), 98.4% (280 nm), *t*_M_ (DMSO) = 1.2 min (MeCN/H_2_O 80:20); HPLC (Method 2) retention time = 14.5 min, 99.1%, *t*_M_ (DMSO) = 1.2 min, λ_max_ = 269 nm.

The compound is described in the literature.^69,71^

##### [6-Methoxy-2-(4-methoxyphenyl)benzo[*b*]thiophen-3-yl](*m*-tolyl)methanone (**9f**)

Following general procedure 1 using 6-methoxy-2-(4-methoxyphenyl)benzo[*b*]thiophene (**8**, 602 mg, 2.23 mmol, 1.0 equiv), 3-methylbenzoyl chloride (354 μL, 2.67 mmol 1.2 equiv), and aluminum chloride (446 mg, 3.35 mmol 1.5 equiv) in CH_2_Cl_2_ (20 mL). The reaction was stopped by the addition of 2 M HCl_aq_ (20 mL) after 24 h. Extracted with CH_2_Cl_2_ (3 × 20 mL) and washed with brine, followed by brine/2 M HCl_aq_ (1:1) and brine (50 mL each). Purification by silica gel chromatography (*n*-hexane/EtOAc 6:1) yielded a pale-yellow solid (465 mg, 1.20 mmol, 54%): C_24_H_20_O_3_S (388.48); mp 117–118 °C; IR (KBr) *ṽ* 1639 (C=O), 1248 (Ar–O-Me) cm^–1^; ^1^H NMR (500 MHz, C_2_D_6_OS) δ_H_ 7.67 (d, *J* = 2.4 Hz, 1H), 7.55–7.52 (m, 1H), 7.48–7.44 (m, 1H), 7.41 (d, *J* = 8.9 Hz, 1H), 7.37–7.34 (m, 1H), 7.31–7.27 (m, 2H), 7.26 (dd, *J* = 7.6, 7.6 Hz, 1H), 7.02 (dd, *J* = 8.9, 2.4 Hz, 1H), 6.90–6.83 (m, 2H), 3.85 (s, 3H), 3.70 (s, 3H), 2.24 (s, 3H) ppm; ^13^C NMR (126 MHz, C_2_D_6_OS) δ_C_ 193.75, 159.56, 157.41, 142.52, 139.42, 138.05, 136.80, 134.30, 133.07, 129.90 (2C), 129.84, 129.62, 128.61, 126.82, 125.20, 123.42, 115.14, 114.30 (2C), 105.16, 55.57, 55.21, 20.68 ppm; elemental analysis [%] calcd: C 71.27, H 4.98, found: C 71.17, H 4.98.; MS (APCI-DI^+^) *m*/*z* [M + H]^+^ 389 (100%); HPLC (Method 1) retention time = 7.2 min, 99.8% (254 nm), 99.8% (280 nm), *t*_M_ (DMSO) = 1.1 min (MeCN/H_2_O 70:30); HPLC (Method 2) retention time = 14.2 min, 99.9%, *t*_M_ (DMSO) = 1.2 min, λ_max_ = 259, 302 nm.

The compound is described in the literature.^67^

##### (3-Fluorophenyl)[6-methoxy-2-(4-methoxyphenyl)benzo[*b*]thiophen-3-yl]methanone (**9h**)

Following general procedure 1 using 6-methoxy-2-(4-methoxyphenyl)benzo[*b*]thiophene (**8**, 518 mg, 1.92 mmol, 1.0 equiv), 3-fluorobenzoyl chloride (280 μL, 2.31 mmol, 1.2 equiv), and aluminum chloride (337 mg, 2.53 mmol, 1.3 equiv) in CH_2_Cl_2_ (20 mL). The reaction was stopped by the addition of 2 M HCl_aq_ (20 mL) after 23 h. Extracted with CH_2_Cl_2_ (3 × 20 mL) and washed with brine, a mixture of brine and 2 M HCl_aq_ (1:1), H_2_O, and brine (50 mL each). Purification by silica gel chromatography (*n*-hexane/EtOAc 6:1) yielded a pale-yellow solid (568 mg, 1.45 mmol, 76%): C_23_H_17_FO_3_S (392.44); mp 129–130 °C; IR (KBr) *ṽ* 1644 (C=O), 1253 (Ar–O–CH_3_) cm^–1^; ^1^H NMR (400 MHz, C_2_D_6_OS) δ_H_ 7.69 (d, *J* = 2.4 Hz, 1H), 7.54 (d, *J* = 8.9 Hz, 1H), 7.47–7.41 (m, 2H), 7.39–7.35 (m, 2H), 7.32–7.23 (m, 2H), 7.05 (dd, *J* = 8.9, 2.4 Hz, 1H), 6.90–6.81 (m, 2H), 3.85 (s, 3H), 3.70 (s, 3H) ppm; ^13^C NMR (101 MHz, C_2_D_6_OS) δ_C_ 192.10 (d, ^4^*J*_*CF*_ = 2.5 Hz), 161.89 (d, ^1^*J*_*CF*_ = 245.7 Hz), 159.71, 157.50, 144.36, 139.51, 139.25 (d, ^3^*J*_*CF*_ = 6.4 Hz), 132.85, 130.82 (d, ^3^*J*_*CF*_ = 7.9 Hz), 130.20 (2C), 128.99, 125.78 (d, ^4^*J*_*CF*_ = 2.6 Hz), 124.98, 123.56, 120.34 (d, ^2^*J*_*CF*_ = 21.5 Hz), 115.36 (d, ^2^*J*_*CF*_ = 22.7 Hz), 115.26, 114.28 (2C), 105.16, 55.58, 55.23 ppm; elemental analysis [%] calcd: C 70.39, H 4.37, found: C 70.34, H 4.24; MS (APCI-DI^+^) *m*/*z* [M + H]^+^ 393 (100%); HPLC (Method 1) retention time = 6.3 min, 96.7% (254 nm), 96.9% (280 nm), *t*_M_ (DMSO) = 1.1 min (MeCN/H_2_O 70:30); HPLC (Method 2) retention time = 14.0 min, 97.6%, *t*_M_ (DMSO) = 1.2 min, λ_max_ = 285 nm.

##### (3-Bromophenyl)[6-methoxy-2-(4-methoxyphenyl)benzo[*b*]thiophen-3-yl]methanone (**9i**)

Following general procedure 1 using 6-methoxy-2-(4-methoxyphenyl)benzo[*b*]thiophene (**8**, 452 mg, 1.67 mmol, 1.0 equiv), 3-bromobenzoyl chloride (264 μL, 2.00 mmol, 1.2 equiv), and aluminum chloride (341 mg, 2.56 mmol, 1.5 equiv) in CH_2_Cl_2_ (20 mL). The reaction was stopped by the addition of 2 M HCl_aq_ (20 mL) after 21 h. Extracted with CH_2_Cl_2_ (3 × 20 mL) and washed with brine, a mixture of brine and 2 M HCl_aq_ (1:1), H_2_O, and brine (50 mL each). Purification by silica gel chromatography (*n*-hexane/EtOAc 6:1) yielded a pale-yellow solid (375 mg, 0.865 mmol, 52%): C_23_H_17_BrO_3_S (453.35); mp 99–100 °C; IR (KBr) *ṽ* 1647 (C=O); 1245 (Ar–O–CH_3_) cm^–1^; ^1^H NMR (400 MHz, C_2_D_6_OS) δ_H_ 7.76 (ddd, *J* = 2.0, 1.6, 0.4 Hz, 1H), 7.72–7.67 (m, 2H), 7.61 (ddd, *J* = 7.9, 1.6, 1.0 Hz, 1H), 7.57 (dd, *J* = 8.9, 0.5 Hz, 1H), 7.31–7.24 (m, 3H), 7.06 (dd, *J* = 8.9, 2.5 Hz, 1H), 6.89–6.81 (m, 2H), 3.85 (s, 3H), 3.70 (s, 3H) ppm; ^13^C NMR (101 MHz, C_2_D_6_OS) δ_C_ 191.78, 159.72, 157.52, 144.73, 139.49, 138.95, 135.86, 132.79, 131.62, 130.77, 130.25 (2C), 128.82, 128.42, 124.94, 123.62, 121.78, 115.28, 114.28 (2C), 105.14, 55.57, 55.24 ppm; elemental analysis [%] calcd: C 60.94, H 3.78, found: C 61.39, H 3.90; MS (APCI-DI^+^) *m*/*z* [M-269]^+^ 183 (99%), [M + H]^+^ 453 (85%); HPLC (Method 1) retention time = 4.6 min, 97.7% (254 nm), 97.2% (280 nm), *t*_M_ (DMSO) = 1.1 min (MeCN/H_2_O 80:20); HPLC (Method 2) retention time = 14.7 min, 99.2%, *t*_M_ (DMSO) = 1.2 min; λ_max_ = 286 nm.

##### [6-Methoxy-2-(4-methoxyphenyl)benzo[*b*]thiophen-3-yl](3-methoxyphenyl)methanone (**9j**)

Following general procedure 1 using 6-methoxy-2-(4-methoxyphenyl)benzo[*b*]thiophene (**8**, 515 mg, 1.91 mmol, 1.0 equiv), 3-methoxybenzoyl chloride (312 μL, 2.29 mmol, 1.2 equiv), and aluminum chloride (396 mg, 2.97 mmol, 1.6 equiv) in CH_2_Cl_2_ (20 mL). The reaction was stopped by the addition of 2 M HCl_aq_ (20 mL) after 4.5 h. Extracted with CH_2_Cl_2_ (3 × 20 mL) and washed with brine, a mixture of brine and 2 M HCl_aq_ (1:1), H_2_O, and brine (50 mL each). A fraction (557 mg, 1.38 mmol) of the crude product (783 mg, 1.93 mmol) was purified by recrystallization from ethanol (1. 11 mL, 2. 9 mL) yielding pale-yellow crystals (291 mg, 0.720 mmol, 38%): C_24_H_20_O_4_S (404.48); mp 120–121 °C; IR (KBr) *ṽ* 1656 (C=O), 1248 (Ar–O-Me) cm^–1^; ^1^H NMR (400 MHz, C_2_D_6_OS) δ_H_ 7.67 (d, *J* = 2.4 Hz, 1H), 7.44 (d, *J* = 8.9 Hz, 1H), 7.32–7.26 (m, 3H), 7.25 (dd, *J* = 2.7, 1.4 Hz, 1H), 7.22 (ddd, *J* = 7.5, 1.4, 1.4 Hz, 1H), 7.12 (ddd, *J* = 8.0, 2.7, 1.4 Hz, 1H), 7.03 (dd, *J* = 8.9, 2.5 Hz, 1H), 6.91–6.83 (m, 2H), 3.85 (s, 3H), 3.72 (s, 3H), 3.71 (s, 3H) ppm; ^13^C NMR (101 MHz, C_2_D_6_OS) δ_C_ 193.35, 159.57, 159.21, 157.40, 142.65, 139.38, 138.17, 133.04, 129.90 (2C), 129.85, 129.62, 125.12, 123.41, 122.19, 119.80, 115.12, 114.31 (2C), 113.37, 105.13, 55.54, 55.21, 55.18 ppm; elemental analysis [%] calcd: C 71.27, H 4.98, found: C 71.17, H 4.98; MS (APCI-DI^+^) *m*/*z* [M + H]^+^ 405 (100%); HPLC (Method 1) retention time = 5.7 min, 98.4% (254 nm), 98.5% (280 nm), *t*_M_ (DMSO) = 1.1 min (MeCN/H_2_O 70:30); HPLC (Method 2) retention time = 13.7 min, 99.1% (no peak separation), *t*_M_ (DMSO) = 1.2 min; λ_max_ = 240, 307 nm.

The compound is described in the literature.^72^

##### [6-Methoxy-2-(4-methoxyphenyl)benzo[*b*]thiophen-3-yl](*o*-tolyl)methanone (**9k**)

Following general procedure 1 using 6-methoxy-2-(4-methoxyphenyl)benzo[*b*]thiophene (**8**, 506 mg, 1.87 mmol, 1.0 equiv), 2-methylbenzoyl chloride (292 μL, 2.25 mmol 1.2 equiv), and aluminum chloride (391 mg, 2.93 mmol 1.6 equiv) in CH_2_Cl_2_ (20 mL). Further addition of 2-methylbenzoyl chloride (122 μL, 0,935 mmol, 0.5 equiv) and aluminum chloride (145 mg, 1.09 mmol, 0.6 equiv) after 8 h. The reaction was stopped by the addition of 2 M HCl_aq_ (20 mL) after 24 h. Extracted with CH_2_Cl_2_ (3 × 20 mL) and washed with brine, a mixture of brine and 2 M HCl_aq_ (1:1), H_2_O, and brine (50 mL each). Purification by silica gel chromatography (*n*-hexane/EtOAc 6:1) yielded a pale-yellow solid (358 mg, 0.922 mmol, 49%). A fraction (156 mg, 0.402 mmol) was further purified by recrystallization from ethanol (1.2 mL) yielding yellow crystals (122 mg, 0.314 mmol, 17%): C_24_H_20_O_3_S (388.48); mp 101–102 °C (ethanol); IR (KBr) *ṽ* 1640 (C=O), 1243 (Ar–O-Me) cm^–1^; ^1^H NMR (400 MHz, C_2_D_6_OS) δ_H_ 7.66 (d, *J* = 2.4 Hz, 1H), 7.61 (d, *J* = 9.0 Hz, 1H), 7.31–7.22 (m, 3H), 7.21 (dd, *J* = 7.8, 1.4 Hz, 1H), 7.19–7.15 (m, 1H), 7.05 (dd, *J* = 9.0, 2.4 Hz, 1H), 7.04–6.99 (m, 1H), 6.83–6.78 (m, 2H), 3.85 (s, 3H), 3.69 (s, 3H), 2.43 (s, 3H) ppm; ^13^C NMR (101 MHz, C_2_D_6_OS) δ_C_ 194.83, 159.56, 157.41, 145.29, 139.29, 137.84, 132.81, 131.64, 131.25, 131.15, 130.40, 130.13 (2C), 125.54, 125.05, 123.78, 115.24, 113.93 (2C), 105.07, 55.55, 55.20, 20.25 ppm, one aromatic carbon atom could not be detected due to superposition; elemental analysis [%] calcd: C 74.2, H 5.19, found: C 74.46, H 5.09; MS (APCI-DI^+^) *m*/*z* [M-269]^+^ 119 (100%), [M-118]^+^ 297 (100%), [M + H]^+^ 389 (39%); HPLC (Method 1) retention time = 7.2 min, 96.5% (254 nm), 95.2% (280 nm), *t*_M_ (DMSO) = 1.1 min (MeCN/H_2_O 70:30); HPLC (Method 2) retention time = 14.1 min, 96.8%, *t*_M_ (DMSO) = 1.2 min; λ_max_ = 262 nm.

##### (2-Fluorophenyl)[6-methoxy-2-(4-methoxyphenyl)benzo[*b*]thiophen-3-yl]methanone (**9l**)

Following general procedure 1 using 6-methoxy-2-(4-methoxyphenyl)benzo[*b*]thiophene (**8**, 274 mg, 1.01 mmol, 1.0 equiv), 2-fluorobenzoyl chloride (130 μL, 1.08 mmol, 1.1 equiv), and aluminum chloride (173 mg, 1.30 mmol, 1.3 equiv) in CH_2_Cl_2_ (20 mL). Further addition of aluminum chloride (79 mg, 0.6 mmol, 0.6 equiv) after 23 h. The reaction was stopped by the addition of 2 M HCl_aq_ (20 mL) after 30 h. Extracted with CH_2_Cl_2_ (3 × 20 mL) and washed with brine, a mixture of brine and 2 M HCl_aq_ (1:1), H_2_O, and brine (50 mL each). Purification by silica gel chromatography (*n*-hexane/EtOAc 4:1) yielded a pale-yellow solid (295 mg, 0.75 mmol, 75%): C_23_H_17_FO_3_S (392.44); mp 132–133 °C; IR (KBr) *ṽ* 1638 (C=O) cm^–1^; ^1^H NMR (400 MHz, C_2_D_6_OS) δ_H_ 7.81 (dd, ^3,5^*J* = 8.9, 0.5 Hz, 1H), 7.66 (d, ^4^*J* = 2.4 Hz, 1H), 7.48 (ddd, ^4^*J*_*HF*_ = 7.6, ^3,4^*J*_*HH*_ = 7.6, 1.8 Hz, 1H), 7.42 (dddd, ^4^*J*_*HF*_ = 5.2, ^3,3,4^*J*_*HH*_ = 8.3, 7.6, 1.8 Hz, 1H), 7.28–7.20 (m, 2H), 7.15–7.06 (m, 2H), 7.00 (ddd, ^3^*J*_*HF*_ = 11.0, ^3,4^*J*_*HH*_ = 8.3, 1.0 Hz, 1H), 6.83–6.75 (m, 2H), 3.85 (s, 3H), 3.68 (s, 3H) ppm; ^13^C NMR (101 MHz, C_2_D_6_OS) δ_C_ 189.16, 159.81 (d, ^1^*J*_*CF*_ = 253.6 Hz), 159.72, 157.49, 147.45 (d, ^4^*J*_*CF*_ = 2.0 Hz), 139.25, 134.43 (d, ^3^*J*_*CF*_ = 9.0 Hz), 132.32, 131.08 (d, ^4^*J*_*CF*_ = 1.6 Hz), 130.55 (2C), 130.31, 127.27 (d, ^2^*J*_*CF*_ = 11.4 Hz), 124.61, 124.37 (d, ^3^*J*_*CF*_ = 3.5 Hz), 123.83, 116.06 (d, ^2^*J*_*CF*_ = 21.7 Hz), 115.41, 113.90 (2C), 105.06, 55.57, 55.24 ppm; elemental analysis [%] calcd: C 70.39, H 4.37, found: C 70.64, H 4.16; MS (APCI-DI^+^) *m*/*z* [M + H]^+^ 393 (100%), MS (APCI-DI^–^) *m*/*z* [M-99]^−^ 283 (100%); HPLC (Method 1) retention time = 5.2 min, 98.6% (254 nm), 97.7% (280 nm), *t*_M_ (DMSO) = 1.1 min (MeCN/H_2_O 70:30); HPLC (Method 2) retention time = 13.3 min, 99.0%, *t*_M_ (DMSO) = 1.2 min, λ_max_ = 279, 351 nm.

##### [6-Hydroxy-2-(4-hydroxyphenyl)benzo[*b*]thiophen-3-yl](phenyl)methanone (**10a**)

Following general procedure 2 using [6-methoxy-2-(4-methoxyphenyl)benzo[*b*]thiophen-3-yl](phenyl)methanone (**9a**, 287 mg, 0.766 mmol) and boron tribromide (1 M in CH_2_Cl_2_, 2.3 mL, 2.3 mmol, 3.0 equiv) in CH_2_Cl_2_ (15 mL). The reaction was stopped by the addition of H_2_O (10 mL) after 6.5 h. Extracted after the addition of EtOAc (10 mL) with EtOAc (3 × 10 mL) and washed with brine (3 × 40 mL). Purification by silica gel chromatography (0–50% EtOAc in *n*-hexane) yielded an orange solid (202 mg, 0.583 mmol, 76%): C_21_H_14_O_3_S (346.40); mp 220–221 °C [lit. 203–205 °C (methanol/H_2_O)];^67^ IR (KBr) *ṽ* 3415 (OH), 1625 (C=O) cm^–1^; ^1^H NMR (500 MHz, C_2_D_6_OS) δ_H_ 9.78 (s, 1H), 9.70 (s, 1H), 7.68–7.64 (m, 2H), 7.52 (dddd, *J* = 7.4, 7.4, 1.3, 1.2 Hz, 1H), 7.37 (d, *J* = 2.4 Hz, 1H), 7.36–7.33 (m, 3H), 7.17–7.11 (m, 2H), 6.88 (dd, *J* = 8.7, 2.4 Hz, 1H), 6.67–6.60 (m, 2H) ppm; ^13^C NMR (126 MHz, C_2_D_6_OS) δ_C_ 193.87, 157.90, 155.47, 142.37, 139.28, 136.98, 133.45, 132.12, 129.98 (2C), 129.32, 129.22 (2C), 128.59 (2C), 123.66, 123.44, 115.60, 115.30 (2C), 107.11 ppm; MS (APCI-DI^–^) *m*/*z* 345 [M – H]^−^ (100%); HRMS (ESI^+^) *m*/*z* [M + H]^+^ calcd: 347.07365 found: 347.07370, [M + Na]^+^ calcd: 369.05559 found: 369.05561, [2M + Na]^+^ calcd: 715.12196, found: 715.12192; HPLC (Method 1) retention time = 7.6 min, 99.9% (254 nm), 99.9% (280 nm), *t*_M_ (DMSO) = 1.1 min (MeCN/H_2_O 40:60); HPLC (Method 2) retention time = 10.3 min, 97.6%, *t*_M_ (DMSO) = 1.2 min, λ_max_ = 255, 284 nm.

The compound is described in the literature.^67^

##### [6-Hydroxy-2-(4-hydroxyphenyl)benzo[*b*]thiophen-3-yl](*p*-tolyl)methanone (**10b**)

Following general procedure 2 using [6-methoxy-2-(4-methoxyphenyl)benzo[*b*]thiophen-3-yl](*p*-tolyl)methanone (**9b**, 350 mg, 0.900 mmol, 1.0 equiv) and boron tribromide (1 M in CH_2_Cl_2_, 2.70 mL, 2.70 mmol, 3 equiv) in CH_2_Cl_2_ (10 mL). The reaction was stopped by the addition of H_2_O (10 mL) after 18 h. Extracted after the addition of methanol (15 mL) and CH_2_Cl_2_ (5 mL) with 10% methanol in CH_2_Cl_2_ (3 × 20 mL). Purification by silica gel chromatography (20–50% EtOAc in *n*-hexane) yielded an orange solid (251 mg, 0.696 mmol, 77%): C_22_H_16_O_3_S (360.43); mp 244–246 °C; IR (KBr) *ṽ* 3417 (OH), 1626 (C=O) cm^–1^; ^1^H NMR (500 MHz, C_2_D_6_OS) δ_H_ 9.77 (s, 1H), 9.71 (s, 1H), 7.62–7.56 (m, 2H), 7.34 (d, *J* = 2.2 Hz, 1H), 7.27 (d, *J* = 8.7 Hz, 1H), 7.21–7.18 (m, 2H), 7.17–7.14 (m, 2H), 6.86 (dd, *J* = 8.7, 2.4 Hz, 1H), 6.68–6.64 (m, 2H), 2.30 (s, 3H) ppm; ^13^C NMR (126 MHz, C_2_D_6_OS) δ_C_ 193.52, 157.76, 155.34, 144.06, 141.07, 139.16, 134.32, 132.10, 129.69 (2C), 129.42, 129.40 (2C), 129.20 (2C), 123.63, 123.23, 115.59 (2C), 115.14, 107.02, 21.08 ppm; MS (ESI-DI^–^) *m*/*z* [M – H]^−^ 359 (100%); HRMS (ESI^+^) *m*/*z* [M + H]^+^ calcd: 361.08930 found: 361.08944, [M + Na]^+^ calcd: 383.07124 found: 383.07132, [2M + Na]^+^ calcd: 743.15326 found: 743.15370; HPLC (Method 1) retention time = 4.2 min, 97.1% (254 nm), 97.7% (280 nm), *t*_M_ (DMSO) = 1.1 min (MeCN/H_2_O 50:50); HPLC (Method 2) retention time = 10.7 min, 96.0%, *t*_M_ (DMSO) = 1.1 min, λ_max_ = 270 nm.

The compound is described in the literature.^68^

##### (4-Fluorophenyl)[6-hydroxy-2-(4-hydroxyphenyl)benzo[*b*]thiophen-3-yl]methanone (**10c**)

Following general procedure 2 using (4-fluorophenyl)[6-methoxy-2-(4-methoxyphenyl)benzo[*b*]thiophen-3-yl]methanone (**9c**, 96 mg, 0.25 mmol, 1.0 equiv) and boron tribromide (1 M in CH_2_Cl_2_, 750 μL, 0.750 mmol, 3.0 equiv) in CH_2_Cl_2_ (10 mL). The reaction was stopped by the addition of H_2_O (10 mL) after 7 h. Extracted after the addition of methanol (10 mL) and CH_2_Cl_2_ (2.5 mL) with 10% methanol in CH_2_Cl_2_ (3 × 10 mL). Purification by silica gel chromatography (0–50% EtOAc in *n*-hexane) yielded an orange solid (77 mg, 0.21 mmol, 86%): C_21_H_13_FO_3_S (364.39); mp 229–231 °C; IR (KBr) *ṽ* 3400 (OH), 1596 (C=O) cm^–1^; ^1^H NMR (400 MHz, C_2_D_6_OS) δ_H_ 9.80 (s, 1H), 9.74 (s, 1H), 7.76–7.69 (m, 2H), 7.41 (d, *J* = 8.8 Hz, 1H), 7.36 (d, *J* = 2.2 Hz, 1H), 7.21–7.11 (m, 4H), 6.90 (dd, *J* = 8.8, 2.2 Hz, 1H), 6.68–6.63 (m, 2H) ppm; ^13^C NMR (101 MHz, C_2_D_6_OS) δ_C_ 192.19, 164.90 (d, ^1^*J*_*CF*_ = 252.9 Hz), 157.95, 155.49, 142.80, 139.29, 133.75 (d, ^4^*J*_*CF*_ = 2.7 Hz, 2C), 132.27 (d, ^3^*J*_*CF*_ = 9.6 Hz), 131.97, 130.06 (2C), 128.90, 123.53, 123.45, 115.62 (d, ^2^*J*_*CF*_ = 22.1 Hz, 2C), 115.58 (2C), 115.32, 107.08 ppm; MS (ESI-DI^–^) *m*/*z* [M – H]^−^ 363 (100%); HRMS (ESI^+^) *m*/*z* [M + H]^+^ calcd: 365.064216 found: 365.06414, [M + Na]^+^ calcd: 387.046158 found: 387.04630, [2M + Na]^+^ calcd: 751.103098 found: 751.10324; HPLC (Method 1) retention time = 3.7 min, 97.3% (254 nm), 97.5% (280 nm), *t*_M_ (DMSO) = 1.1 min (MeCN/H_2_O 50:50); HPLC (Method 2) retention time = 10.3 min, 96.7%, *t*_M_ (DMSO) = 1.2 min, λ_max_ = 257 nm.

The compound is described in the literature.^71^

##### (4-Chlorophenyl)[6-hydroxy-2-(4-hydroxyphenyl)benzo[*b*]thiophen-3-yl]methanone (**10d**)

Following general procedure 2 using (4-chlorophenyl) [6-methoxy-2-(4-methoxyphenyl)benzo[*b*]thiophen-3-yl]methanone (**9d**, 411 mg, 1.01 mmol, 1.0 equiv) and boron tribromide (1 M in CH_2_Cl_2_, 3 mL, 3 mmol, 3 equiv) in CH_2_Cl_2_ (10 mL). The reaction was stopped by the addition of H_2_O (10 mL) after 2.5 d. Extracted after the addition of methanol (10 mL) and CH_2_Cl_2_ (5 mL) with 10% methanol in CH_2_Cl_2_ (3 × 20 mL). Purification by silica gel chromatography (20–50% EtOAc in *n*-hexane) yielded an orange solid (249 mg, 0.654 mmol, 65%): C_21_H_13_ClO_3_S (380.84); mp 239–242 °C; IR (KBr) *ṽ* 3403 (OH), 1608 (C=O) cm^–1^; ^1^H NMR (400 MHz, C_2_D_6_OS) δ_H_ 9.81 (s, 1H), 9.75 (s, 1H), 7.68–7.62 (m, 2H), 7.44–7.39 (m, 3H), 7.36 (d, *J* = 2.3 Hz, 1H), 7.15–7.10 (m, 2H), 6.90 (dd, *J* = 8.8, 2.3 Hz, 1H), 6.68–6.63 (m, 2H) ppm; ^13^C NMR (101 MHz, C_2_D_6_OS) δ_C_ 192.45, 158.01, 155.52, 143.28, 139.31, 138.12, 135.77, 131.90, 131.12 (2C), 130.12 (2C), 128.69, 128.67 (2C), 123.47 (2C), 115.60 (2C), 115.36, 107.09 ppm; elemental analysis [%] calcd: C 65.79, H 3.49, found: C 65.81, H 3.54; MS (ESI-DI^–^) *m*/*z* [M – H]^−^ 379 (100%); HPLC (Method 1) retention time = 5.1 min, 99.2% (254 nm), 99.0% (280 nm), *t*_M_ (DMSO) = 1.1 min (MeCN/H_2_O 50:50); HPLC (Method 2) retention time = 10.9 min, 95.1%, *t*_M_ (DMSO) = 1.3 min, λ_max_ = 265 nm.

##### (4-Bromophenyl)[6-hydroxy-2-(4-hydroxyphenyl)benzo[*b*]thiophen-3-yl]methanone (**10e**)

Following general procedure 2 using (4-bromophenyl)[6-methoxy-2-(4-methoxyphenyl)benzo[*b*]thiophen-3-yl]methanone (**9e**, 455 mg, 1.00 mmol, 1.0 equiv) and boron tribromide (1 M in CH_2_Cl_2_, 3 mL, 3 mmol, 3 equiv) in CH_2_Cl_2_ (10 mL). The reaction was stopped by the addition of H_2_O (20 mL) after 5 h. Extracted after the addition of methanol (5 mL) and CH_2_Cl_2_ (20 mL) with 10% methanol in CH_2_Cl_2_ (3 × 20 mL) and washed with brine (3 × 100 mL). Purification by silica gel chromatography (EtOAc/*n*-hexane 1:2) yielded an orange solid (210 mg, 0.494 mmol, 49%): C_21_H_13_BrO_3_S (425.30); mp 236–238 °C [lit. 233–234 °C];^71^ IR (KBr) *ṽ* 3417 (OH), 1603 (C=O) cm^–1^; ^1^H NMR (500 MHz, C_2_D_6_OS) δ_H_ 9.80 (s, 1H), 9.75 (s, 1H), 7.60–7.53 (m, 4H), 7.41 (d, *J* = 8.8 Hz, 1H), 7.36 (d, *J* = 2.3 Hz, 1H), 7.15–7.10 (m, 2H), 6.89 (dd, *J* = 8.8, 2.3 Hz, 1H), 6.68–6.64 (m, 2H) ppm; ^13^C NMR (126 MHz, C_2_D_6_OS) δ_C_ 192.59, 157.95, 155.44, 143.17, 139.22, 136.00, 131.81, 131.55 (2C), 131.13 (2C), 130.03 (2C), 128.59, 127.33, 123.38, 123.37, 115.54 (2C), 115.29, 107.01 ppm; MS (ESI-DI^–^) *m*/*z* [M – H]^−^ 423 (100%); HRMS (ESI^+^) *m*/*z* [M + H]^+^ calcd: 424.98416 found: 424.98432, [M + Na]^+^ calcd: 446.96610 found: 446.96622; HPLC (Method 1) retention time = 4.2 min, 95.6% (254 nm), 96.6% (280 nm), *t*_M_ (DMSO) = 1.1 min (MeCN/H_2_O 50:50); HPLC (Method 2) retention time = 10.7 min, 95.2%, *t*_M_ (DMSO) = 1.2 min, λ_max_ = 271 nm.

The compound is described in the literature.^71^

##### [6-Hydroxy-2-(4-hydroxyphenyl)benzo[*b*]thiophen-3-yl](*m*-tolyl)methanone (**10f**)

Following general procedure 2 using [6-methoxy-2-(4-methoxyphenyl)benzo[*b*]thiophen-3-yl](*m*-tolyl)methanone (**9f**, 312 mg, 0.804 mmol, 1.0 equiv) and boron tribromide (1 M in CH_2_Cl_2_, 2.4 mL, 2.4 mmol, 3.0 equiv) in CH_2_Cl_2_ (10 mL). The reaction was stopped by the addition of H_2_O (15 mL) after 2 h. Extracted after the addition of EtOAc (10 mL) with EtOAc (3 × 10 mL) and washed with brine/2 M HCl_aq_ (1:1) (2 × 20 mL). Purification by silica gel chromatography (EtOAc/*n*-hexane 2:1) yielded an orange solid, which was further purified by preparative HPLC (MeCN/H_2_O 50:50) yielded an orange solid (50 mg, 0.14 mmol, 17%): C_22_H_16_O_3_S (360.43); mp 218–219 °C; IR (KBr) *ṽ* 3415 (OH), 1626 (C=O) cm^–1^; ^1^H NMR (500 MHz, C_2_D_6_OS) δ_H_ 9.78 (s, 1H), 9.70 (s, 1H), 7.51–7.50 (m, 1H), 7.46–7.43 (m, 1H), 7.37–7.30 (m, 3H), 7.24 (dd, *J* = 7.6, 7.6 Hz, 1H), 7.17–7.11 (m, 2H), 6.87 (dd, *J* = 8.8, 2.2 Hz, 1H), 6.68–6.61 (m, 2H), 2.24 (s, 3H) ppm; ^13^C NMR (126 MHz, C_2_D_6_OS) δ_C_ 193.86, 157.87, 155.44, 142.21, 139.26, 137.90, 136.91, 134.10, 132.13, 129.92 (2C), 129.64, 129.39, 128.50, 126.77, 123.77, 123.44, 115.58 (2C), 115.28, 107.09, 20.69 ppm; MS (ESI-DI^–^) *m*/*z* 359 [M – H]^−^ (100%); HRMS (ESI^+^) *m*/*z* [M + H]^+^ calcd: 361.08930, found: 361.08942; [M + Na]^+^ calcd: 383.07124, found: 383.07136; [2M + Na]^+^ calcd: 743.15326, found: 743.15353; HPLC (Method 1) retention time = 4.2 min, 98.1% (254 nm), 98.6% (280 nm), *t*_M_ (DMSO) = 1.1 min (MeCN/H_2_O 50:50); HPLC (Method 2) retention time = 10.7 min, 96.3%, *t*_M_ (DMSO) = 1.2 min, λ_max_ = 261, 284 nm.

The compound is described in the literature.^67^

##### [6-Hydroxy-2-(4-hydroxyphenyl)benzo[*b*]thiophen-3-yl](3-isopropylphenyl)methanone (**10g**)

The reaction was carried out in the absence of moisture. Isopropylbenzoic acid (503 mg, 3.06 mmol, 1.6 equiv) and 3 drops of dimethylformamide were added portionwise to thionyl chloride (1.0 mL, 14 mmol, 4.5 equiv). The reaction was heated to 50 °C and stirred for 30 min. Gas formation occurred, after which the temperature was increased to 80 °C and stirred for 2 h to reflux. The mixture was cooled and concentrated under reduced pressure. The resulting yellow oil was cooled with an ice–water mixture and CH_2_Cl_2_ (20 mL) and 6-methoxy-2-(4-methoxyphenyl)benzo[*b*]thiophene (**8**, 514 mg, 1.90 mmol, 1.0 equiv) were added. Aluminum chloride (384 mg, 2.88 mmol, 1.5 equiv) was added in portions, the mixture turned dark red. The reaction was stirred overnight until ambient temperature was reached. After 20 h, the reaction was stopped by the addition of 2 M hydrochloric acid solution (20 mL) and stirred for 20 min; the solution turned green. The aqueous layer was extracted with CH_2_Cl_2_ (3 × 20 mL) and the combined organic layers were washed with a mixture of brine and 2 M hydrochloric acid (1:1), 0.1 M NaOH and brine (50 mL each). The combined organic layers were dried over Na_2_SO_4_, filtered, and concentrated *in vacuo*. The crude product was purified by silica gel column chromatography (*n*-hexane/EtOAc 8:1) yielding a yellow gel (0.21 g, 0.50 mmol). This was dissolved in dried CH_2_Cl_2_ (10 mL) in the absence of moisture, cooled with an ice–water mixture, and boron tribromide (1.75 mL, 1.75 mmol, 3.5 equiv) was added. The reaction was stirred at ambient temperature for 6 h. H_2_O (15 mL) was added to terminate the reaction and stirred briefly. The precipitation at the phase boundary was dissolved by the addition of EtOAc (10 mL). The layers were separated and the aqueous layer was extracted with EtOAc (3 × 15 mL). The combined organic layers were washed with brine/2 M HCl_aq_ (1:1) (3 × 50 mL) and dried over Na_2_SO_4_, filtered, and concentrated *in vacuo*. The crude product was purified by column chromatography (EtOAc/*n*-hexane 1:3) yielding an orange solid, which was further purified by preparative HPLC (MeCN/H_2_O 50:50) yielding an orange solid (70 mg, 0.18 mmol, 6%): C_24_H_20_O_3_S (388.48); mp 236–237 °C; IR (KBr) *ṽ* 3401 (OH), 1633 (C=O) cm^–1^; ^1^H NMR (500 MHz, C_2_D_6_OS) δ_H_ 9.78 (s, 1H), 9.68 (s, 1H), 7.50 (ddd, *J* = 7.6, 1.8, 1.5 Hz, 1H), 7.47 (dd, *J* = 1.8, 1.8 Hz, 1H), 7.43 (d, *J* = 8.8 Hz, 1H), 7.37 (ddd, *J* = 7.6, 1.8, 1.5 Hz, 1H), 7.35 (d, *J* = 2.3 Hz, 1H), 7.27 (dd, *J* = 7.6, 7.6 Hz, 1H), 7.15–7.08 (m, 2H), 6.89 (dd, *J* = 8.8, 2.3 Hz, 1H), 6.64–6.58 (m, 2H), 2.79 (hept, *J* = 7.0 Hz, 1H), 1.07 (d, *J* = 7.0 Hz, 6H) ppm; ^13^C NMR (126 MHz, C_2_D_6_OS) δ_C_ 193.66, 157.90, 155.46, 148.52, 142.88, 139.29, 137.04, 132.19, 131.45, 130.11 (2C), 129.20, 128.58, 127.25, 126.97, 123.74, 123.60, 115.51 (2C), 115.28, 107.07, 33.08, 23.51 (2C) ppm; MS (ESI-DI^–^) *m*/*z* 387 [M – H]^−^ (100%); HRMS (ESI^+^) *m*/*z* [M + H]^+^ calcd: 389.12060 found: 389.12079, [M + Na]^+^ calcd: 411.10254 found: 411.10266, [2M + Na]^+^ calcd: 799.21586 found: 799.21623; HPLC (Method 1) retention time = 3.3 min, 97.3% (254 nm), 98.0% (280 nm), *t*_M_ (DMSO) = 1.1 min (MeCN/H_2_O 60:40); HPLC (Method 2) retention time = 11.4 min, 97.0%, *t*_M_ (DMSO) = 1.2 min, λ_max_ = 261, 282 nm.

##### (3-Fluorophenyl)[6-hydroxy-2-(4-hydroxyphenyl)benzo[*b*]thiophen-3-yl]methanone (**10h**)

Following general procedure 2 using (3-fluorophenyl)[6-methoxy-2-(4-methoxyphenyl)benzo[*b*]thiophen-3-yl]methanone (**9h**, 297 mg, 0.757 mmol, 1.0 equiv) and boron tribromide (1 M in CH_2_Cl_2_, 2.7 mL, 2.7 mmol, 3.6 equiv) in CH_2_Cl_2_ (10 mL). The reaction was stopped by the addition of H_2_O (10 mL) after 4 h. Extracted after the addition of EtOAc (10 mL) with EtOAc (3 × 15 mL) and washed with brine/2 M HCl_aq_ (1:1) (3 × 20 mL). Purification by silica gel chromatography (EtOAc/*n*-hexane 1:2) yielded an orange solid (152 mg, 0.417 mmol, 55%): C_21_H_13_FO_3_S (364.39); mp 222–224 °C; IR (KBr) *ṽ* 3411 (OH), 1634 (C=O) cm^–1^; ^1^H NMR (400 MHz, C_2_D_6_OS) δ_H_ 9.81 (s, 1H), 9.74 (s, 1H), 7.48 (d, *J* = 8.8 Hz, 1H), 7.44–7.33 (m, 5H), 7.17–7.08 (m, 2H), 6.91 (dd, *J* = 8.8, 2.3 Hz, 1H), 6.68–6.60 (m, 2H) ppm; ^13^C NMR (101 MHz, C_2_D_6_OS) δ_C_ 192.20 (d, ^4^*J*_*CF*_ = 2.2 Hz), 161.84 (d, ^1^*J*_*CF*_ = 245.7 Hz), 158.05, 155.56, 144.13, 139.42 (d, ^3^*J*_*CF*_ = 6.2 Hz), 139.34, 131.89, 130.69 (d, ^3^*J*_*CF*_ = 7.7 Hz), 130.22 (2C), 128.58, 125.72 (d, ^4^*J*_*CF*_ = 2.7 Hz), 123.57, 123.54, 120.11 (d, ^2^*J*_*CF*_ = 21.2 Hz), 115.57 (2C), 115.41, 115.35 (d, ^2^*J*_*CF*_ = 22.2 Hz), 107.09 ppm; elemental analysis [%] calcd: C 69.22, H 3.60, found: C 68.84, H 3.58; MS (ESI-DI^–^) *m*/*z* 363 [M – H]^−^ (100%); HPLC (Method 1) retention time = 3.8 min, 97.4% (254 nm), 97.9% (280 nm), *t*_M_ (DMSO) = 1.1 min (MeCN/H_2_O 50:50); HPLC (Method 2) retention time = 10.5 min, 97.5%, *t*_M_ (DMSO) = 1.2 min, λ_max_ = 288, 363 nm.

The compound is described in the literature.^67^

##### (3-Bromophenyl)[6-hydroxy-2-(4-hydroxyphenyl)benzo[*b*]thiophen-3-yl]methanone (**10i**)

Following general procedure 2 using (3-bromophenyl)[6-methoxy-2-(4-methoxyphenyl)benzo[*b*]thiophen-3-yl]methanone (**9i**, 184 mg, 0.406 mmol, 1.0 equiv) and boron tribromide (1 M in CH_2_Cl_2_, 1.4 mL, 1.4 mmol, 3.5 equiv) in CH_2_Cl_2_ (10 mL). The reaction was stopped by the addition of H_2_O (15 mL) after 4 h. Extracted after the addition of EtOAc (10 mL) with EtOAc (3 × 15 mL) and washed with brine/2 M HCl_aq_ (1:1) (4 × 20 mL). Purification by silica gel chromatography (EtOAc/*n*-hexane 1:2) yielded an orange solid (111 mg, 0.261 mmol, 64%): C_21_H_13_BrO_3_S (425.30); mp 241–243 °C; IR (KBr) *ṽ* 3399 (OH), 1620 (C=O) cm^–1^; ^1^H NMR (400 MHz, C_2_D_6_OS) δ_H_ 9.82 (s, 1H), 9.74 (s, 1H), 7.73 (dd, *J* = 2.0, 1.6 Hz, 1H), 7.67 (ddd, *J* = 7.8, 2.0, 1.1 Hz, 1H), 7.58 (ddd, *J* = 7.8, 1.6, 1.1 Hz, 1H), 7.51 (d, *J* = 8.8 Hz, 1H), 7.36 (d, *J* = 2.3 Hz, 1H), 7.27 (dd, *J* = 7.8, 7.8 Hz, 1H), 7.16–7.08 (m, 2H), 6.91 (dd, *J* = 8.8, 2.3 Hz, 1H), 6.68–6.60 (m, 2H) ppm; ^13^C NMR (101 MHz, C_2_D_6_OS) δ_C_ 191.85, 158.07, 155.57, 144.49, 139.33, 139.09, 135.66, 131.83, 131.65, 130.68, 130.26 (2C), 128.39, 128.36, 123.63, 123.48, 121.68, 115.57 (2C), 115.44, 107.08 ppm; elemental analysis [%] calcd: C 59.31, H 3.08, found: C 59.54, H 3.43; MS (ESI-DI^–^) *m*/*z* [M – H]^−^ 423 (98%); HRMS (ESI^+^) *m*/*z* [M + Na]^+^ calcd: 446.96610 found: 446.96633, [2M + Na]^+^ calcd: 872.94093 found: 872.94136; HPLC (Method 1) retention time = 6.4 min, 98.4% (254 nm), 98.1% (280 nm), *t*_M_ (DMSO) = 1.1 min (MeCN/H_2_O 50:50); HPLC (Method 2) retention time = 11.1 min, 97.8%, *t*_M_ (DMSO) = 1.2 min, λ_max_ = 289 nm.

##### [6-Hydroxy-2-(4-hydroxyphenyl)benzo[*b*]thiophen-3-yl](3-hydroxyphenyl)methanone (**10j**)

Following general procedure 2 using [6-methoxy-2-(4-methoxyphenyl)benzo[*b*]thiophen-3-yl](3-methoxyphenyl)methanone (**9j**, 219 mg, 0.541 mmol, 1.0 equiv) and boron tribromide (1 M in CH_2_Cl_2_, 2.8 mL, 2.8 mmol, 5.2 equiv) in CH_2_Cl_2_ (10 mL). The reaction was stopped by the addition of H_2_O (10 mL) after 3 h. Extracted after the addition of EtOAc (10 mL) with EtOAc (3 × 10 mL) and washed with brine/2 M HCl_aq_ (1:1) (4 × 20 mL). Purification by silica gel chromatography (EtOAc/*n*-hexane 2:3) yielded a yellow oil, which crystallized upon layering with CH_2_Cl_2_ (127 mg, 0.350 mmol, 65%): C_21_H_14_O_4_S (362.40); mp 140 °C (dec.); IR (KBr) *ṽ* 3411 (OH), 1632 (C=O) cm^–1^; ^1^H NMR (400 MHz, C_2_D_6_OS) δ_H_ 9.79 (s, 1H), 9.74 (s, 1H), 9.70 (s, 1H), 7.34 (d, *J* = 2.3 Hz, 1H), 7.29 (d, *J* = 8.8 Hz, 1H), 7.20–7.14 (m, 3H), 7.12–7.08 (m, 2H), 6.91 (ddd, *J* = 7.9, 1.8, 1.2 Hz, 1H), 6.87 (dd, *J* = 8.8, 2.3 Hz, 1H), 6.72–6.64 (m, 2H) ppm; ^13^C NMR (101 MHz, C_2_D_6_OS) δ_C_ 194.05, 157.86, 157.42, 155.42, 141.36, 139.16, 138.28, 132.16, 129.74 (3C), 129.56, 123.71, 123.32, 120.73, 120.33, 115.63 (2C), 115.46, 115.25, 107.08 ppm; MS (ESI-DI^–^) *m*/*z* [M – H]^−^ 361 (100%); HRMS (ESI^+^) *m*/*z* [M + H]^+^ calcd: 363.06856 found: 363.06870, [M + Na]^+^ calcd: 385.05050 found: 385.05065, [2M + Na]^+^ calcd: 747.11178 found: 747.11208; HPLC (Method 1) retention time = 3.6 min, 97.6% (254 nm), 97.7% (280 nm), *t*_M_ (DMSO) = 1.1 min (MeCN/H_2_O 40:60); HPLC (Method 2) retention time = 9.3 min, 99.5%, *t*_M_ (DMSO) = 1.2 min, λ_max_ = 263 nm.

The compound is described in the literature.^72^

##### [6-Hydroxy-2-(4-hydroxyphenyl)benzo[*b*]thiophen-3-yl](*o*-tolyl)methanone (**10k**)

Following general procedure 2 using [6-methoxy-2-(4-methoxyphenyl)benzo[*b*]thiophen-3-yl](*o*-tolyl)methanone (**9k**, 175 mg, 0.451 mmol, 1.0 equiv) and boron tribromide (1 M in CH_2_Cl_2_, 1.6 mL, 1.6 mmol, 3.6 equiv) in CH_2_Cl_2_ (10 mL). The reaction was stopped by the addition of H_2_O (10 mL) after 16 h. Extracted after the addition of CH_2_Cl_2_ (10 mL) and methanol (15 mL) with 10% methanol in CH_2_Cl_2_ (2 × 20 mL) and EtOAc (2 × 10 mL). Purification by silica gel chromatography (EtOAc in *n*-hexane 25–50%) and further purification by preparative HPLC (MeCN/H_2_O 40:60) yielded a yellow solid (54 mg, 0.15 mmol, 33%): C_22_H_16_O_3_S (360.43); mp 213–214 °C; IR (KBr) *ṽ* 3399 (OH), 1605 (C=O) cm^–1^; ^1^H NMR (500 MHz, C_2_D_6_OS) δ_H_ 9.78 (s, 1H), 9.65 (s, 1H), 7.56 (d, *J* = 8.8 Hz, 1H), 7.34 (d, *J* = 2.3 Hz, 1H), 7.25 (ddd, *J* = 7.5, 7.4, 1.4 Hz, 1H), 7.18 (dd, *J* = 7.8, 1.4 Hz, 1H), 7.17–7.14 (m, 1H), 7.12–7.07 (m, 2H), 7.03–6.98 (m, 1H), 6.91 (dd, *J* = 8.8, 2.3 Hz, 1H), 6.62–6.56 (m, 2H), 2.42 (s, 3H) ppm; ^13^C NMR (126 MHz, C_2_D_6_OS) δ_C_ 195.00, 157.86, 155.46, 144.98, 139.15, 138.03, 137.79, 131.88, 131.47, 131.16, 130.77, 130.33, 130.10 (2C), 125.46, 123.80, 123.62, 115.39, 115.22 (2C), 107.01, 20.23 ppm; elemental analysis [%] calcd: C 73.31, H 4.47, found: C 73.12, H 4.82; MS (ESI-DI^–^) *m*/*z* [M – H]^−^ 359 (100%); HRMS (ESI^+^) *m*/*z* [M + H]^+^ calcd: 361.08930 found: 361.08941, [M + Na]^+^ found: calcd: 383.07124, 383.07137, [2M + Na]^+^ calcd: 743.15326 found: 743.15344; HPLC (Method 1) retention time = 4.2 min, 98.1% (254 nm), 98.6% (280 nm), *t*_M_ (DMSO) = 1.1 min (MeCN/H_2_O 50:50); HPLC (Method 2) retention time = 10.6 min, 95.8%, *t*_M_ (DMSO) = 1.2 min, λ_max_ = 260 nm.

##### (2-Fluorophenyl)[6-hydroxy-2-(4-hydroxyphenyl)benzo[*b*]thiophen-3-yl]methanone (**10l**)

Following general procedure 2 using (2-fluorophenyl)[6-methoxy-2-(4-methoxyphenyl)benzo[*b*]thiophen-3-yl]methanone (**9l**, 190 mg, 0.48 mmol, 1.0 equiv) and boron tribromide (1 M in CH_2_Cl_2_, 1.5 mL, 1.5 mmol, 3.1 equiv) in CH_2_Cl_2_ (10 mL). The reaction was stopped by the addition of H_2_O (10 mL) after 5.5 h. Extracted after the addition of CH_2_Cl_2_ (10 mL) and methanol (2.5 mL) with 10% methanol in CH_2_Cl_2_ (3 × 10 mL). Purification by silica gel chromatography (0–50% EtOAc in *n*-hexane) yielded a yellow oil, which crystallized upon layering with petroleum ether (149 mg, 0.409 mmol, 85%): C_21_H_13_FO_3_S (364.39); mp 225–226 °C; IR (KBr) *ṽ* 3330 (OH), 1624 (C=O) cm^–1^; ^1^H NMR (500 MHz, C_2_D_6_OS) δ_H_ 9.80 (s, 1H), 9.69 (s, 1H), 7.74 (d, *J* = 8.8 Hz, 1H), 7.46–7.38 (m, 2H), 7.34 (d, *J* = 2.3 Hz, 1H), 7.13–7.06 (m, 3H), 7.00 (ddd, *J* = 11.0, 8.3, 1.0 Hz, 1H), 6.95 (dd, *J* = 8.8, 2.3 Hz, 1H), 6.61–6.55 (m, 2H) ppm; ^13^C NMR (126 MHz, C_2_D_6_OS) δ_C_ 189.19, 159.71 (d, ^1^*J*_*CF*_ = 253.2 Hz), 157.96, 155.44, 147.19 (d, ^4^*J*_*CF*_ = 1.8 Hz), 139.00, 134.10 (d, ^3^*J*_*CF*_ = 9.0 Hz), 131.27, 130.96 (d, ^4^*J*_*CF*_ = 1.2 Hz), 130.43 (2C), 129.83, 127.37 (d, ^2^*J*_*CF*_ = 11.2 Hz), 124.16 (d, ^3^*J*_*CF*_ = 3.5 Hz), 123.77, 123.03, 115.90 (d, ^2^*J*_*CF*_ = 21.9 Hz), 115.45, 115.08 (2C), 106.91 ppm; elemental analysis [%] calcd: C 69.22, H 3.60, found: C 68.86, H 3.67; MS (APCI-DI^–^) *m*/*z* [M-21]^−^ 343 (100%), [M – H]^−^ 363 (58%); HRMS (ESI^+^) *m*/*z* [M + H]^+^ calcd: 365.064216 found: 365.06431, [M + Na]^+^ calcd: 387.04616 found: 387.04633, [2M + Na]^+^ calcd: 751.10310 found: 751.10314; HPLC (Method 1) retention time = 7.1 min, 99.6% (254 nm), 99.9% (280 nm), *t*_M_ (DMSO) = 1.1 min (MeCN/H_2_O 40:60); HPLC (Method 2) retention time = 10.1 min, 96.4%, *t*_M_ (DMSO) = 1.2 min, λ_max_ = 274, 359 nm.

##### [6-Hydroxy-2-(4-hydroxyphenyl)benzo[*b*]thiophen-3-yl][4-(propylamino)phenyl]methanone (**11a**)

Following general procedure 5 using propylamine (821 μL, 10.0 mmol, 20 equiv) and (4-fluorophenyl)[6-hydroxy-2-(4-hydroxyphenyl)benzo[*b*]thiophen-3-yl]methanone (**10c**, 182 mg, 0.501 mmol, 1.0 equiv) in DMSO (10 mL) for 6 h. Extracted after the addition of 0.1 M HCl_aq_ (50 mL) with EtOAc (3 × 50 mL) and washed with brine (3 × 50 mL). Purification by silica gel chromatography (1–5% MeOH in CH_2_Cl_2_) yielded a yellow solid (143 mg, 0.354 mmol, 71%): C_24_H_21_NO_3_S (403.50); mp 129–132 °C; IR (KBr) *ṽ* 3366 (broad, OH, NH overlaid), 2959, 2928, 2871 (aliph. CH), 1613 (C=O) cm^–1^; ^1^H NMR (500 MHz, C_2_D_6_OS) δ_H_ 9.70 (s, 1H), 9.68 (s, 1H), 7.47 (d, *J* = 8.5 Hz, 2H), 7.30 (d, *J* = 2.3 Hz, 1H), 7.23–7.19 (m, 2H), 7.16 (d, *J* = 8.7 Hz, 1H), 6.82 (dd, *J* = 8.7, 2.3 Hz, 1H), 6.75 (t, *J* = 5.5 Hz, 1H), 6.71–6.67 (m, 2H), 6.49–6.44 (m, 2H), 2.99 (td, *J* = 7.1 Hz, 5.5 Hz, 2H), 1.52 (h, *J* = 7.4 Hz, 2H), 0.90 (t, *J* = 7.4 Hz, 3H) ppm; ^13^C NMR (126 MHz, C_2_D_6_OS) δ_C_ 191.20, 157.49, 155.13, 153.53, 138.95, 137.81, 132.59, 131.86 (2C), 130.73, 129.25 (2C), 124.05, 124.01, 123.20, 115.54 (2C), 114.84, 110.61 (2C) 106.91, 43.85, 21.56, 11.41 ppm; MS (APCI-DI^+^) *m*/*z* [M + H]^+^ 404 (100%); MS (ESI-DI^–^) *m*/*z* [M – H]^−^ 402 (100%); HRMS (ESI^+^) *m*/*z* [M + H]^+^ calcd: 404.13149 found: 404.13166, [M + Na]^+^ calcd: 426.11343 found: 426.11351, [2M + H]^+^ calcd: 807.2557 found: 807.2564, [2M + Na]^+^ calcd: 829.23764 found: 829.23801; HPLC (Method 1) retention time = 4.0 min, 97.4% (254 nm), 97.2% (280 nm), *t*_M_ (DMSO) = 1.1 min (MeCN/H_2_O 50:50); HPLC (Method 2) retention time = 10.4 min, 98.2%, *t*_M_ (DMSO) = 1.2 min, λ_max_ = 318, 350 nm.

##### [6-Hydroxy-2-(4-hydroxyphenyl)benzo[*b*]thiophen-3-yl][4-(isobutylamino)phenyl]methanone (**11b**)

Following general procedure 5 using isobutylamine (1002 μL, 10.00 mmol, 20 equiv) and (4-fluorophenyl)[6-hydroxy-2-(4-hydroxyphenyl)benzo[*b*]thiophen-3-yl]methanone (**10c**, 183 mg, 0.501 mmol, 1.0 equiv) in DMSO (10 mL) for 6 h. Extracted after the addition of 0.1 M HCl_aq_ (50 mL) with EtOAc (3 × 50 mL) and washed with brine (3 × 50 mL). Purification by silica gel chromatography (1–5% MeOH in CH_2_Cl_2_) yielded a yellow solid (165 mg, 0.395 mmol, 79%): C_25_H_23_NO_3_S (417.52); mp 118–120 °C (dec.); IR (KBr) *ṽ* 3390 (broad; OH, NH overlaid), 2955 (aliph. CH), 1613 (C=O) cm^–1^; ^1^H NMR (500 MHz, C_2_D_6_OS) δ_H_ 9.70 (s, 1H), 9.68 (s, 1H), 7.46 (d, *J* = 8.5 Hz, 2H), 7.30 (d, *J* = 2.2 Hz, 1H), 7.24–7.19 (m, 2H), 7.16 (d, *J* = 8.7 Hz, 1H), 6.84–6.78 (m, 2H), 6.72–6.66 (m, 2H), 6.48 (d, *J* = 8.9 Hz, 2H), 2.85 (dd, *J* = 6.8, 5.7 Hz, 2H), 1.78 (hept, *J* = 6.7 Hz, 1H), 0.89 (d, *J* = 6.6 Hz, 6H) ppm; ^13^C NMR (126 MHz, C_2_D_6_OS) δ_C_ 191.18, 157.49, 155.13, 153.68, 138.95, 137.77, 132.59, 131.85 (2C), 130.75, 129.24 (2C), 124.05, 123.96, 123.21, 115.55 (2C), 114.83, 110.67 (2C) 106.91, 49.83, 27.26, 20.16 (2C) ppm; MS (ESI-DI^–^) *m*/*z* [M – H]^−^ 416 (100%); HRMS (ESI^+^) *m*/*z* [M + H]^+^ calcd: 418.14714 found: 418.14743, [M + Na]^+^ calcd: 440.12908 found: 440.12924, [2M + Na]^+^ calcd: 857.26894 found: 857.26946; HPLC (Method 1) retention time = 5.2 min, 97.8% (254 nm), 98.1% (280 nm), *t*_M_ (DMSO) = 1.1 min (MeCN/H_2_O 50:50); HPLC (Method 2) retention time = 10.8 min, 98.0%, *t*_M_ (DMSO) = 1.2 min, λ_max_ = 350 nm.

##### [6-Hydroxy-2-(4-hydroxyphenyl)benzo[*b*]thiophen-3-yl][4-(pentylamino)phenyl]methanone (**11c**)

Following general procedure 5 using 1-pentylamine (1162 μL, 10.00 mmol, 20 equiv) and (4-fluorophenyl)[6-hydroxy-2-(4-hydroxyphenyl)benzo[*b*]thiophen-3-yl]methanone (**10c**, 182 mg, 0.500 mmol, 1.0 equiv) in DMSO (10 mL) for 6 h. Extracted after the addition of 0.1 M HCl_aq_ (50 mL) with EtOAc (3 × 50 mL) and washed with brine (3 × 50 mL). Purification by silica gel chromatography (2% MeOH in CH_2_Cl_2_) yielded a yellow solid (121 mg, 0.281 mmol, 56%): C_26_H_25_NO_3_S (431.55); mp 117–119 °C (dec.); IR (KBr) *ṽ* 3367 (broad, OH, NH overlaid), 2926, 2856 (aliph. CH), 1613 (C=O); ^1^H NMR (500 MHz, C_2_D_6_OS) δ_H_ 9.70 (s, 1H), 9.68 (s, 1H), 7.46 (d, *J* = 8.4 Hz, 2H), 7.30 (d, *J* = 2.2 Hz, 1H), 7.24–7.19 (m, 2H), 7.16 (d, *J* = 8.7 Hz, 1H), 6.81 (dd, *J* = 8.7, 2.3 Hz, 1H), 6.74 (t, *J* = 5.4 Hz, 1H), 6.71–6.67 (m, 2H), 6.49–6.43 (m, 2H), 3.02 (td, *J* = 7.1, 5.4 Hz, 2H), 1.55–1.46 (m, 2H), 1.32–1.26 (m, 4H), 0.89–0.84 (m, 3H) ppm; ^13^C NMR (126 MHz, C_2_D_6_OS) δ_C_ 191.20, 157.49, 155.13, 153.51, 138.95, 137.80, 132.59, 131.85 (2C), 130.73, 129.24 (2C), 124.04, 124.00, 123.20, 115.54 (2C), 114.83, 110.63 (2C), 106.91, 42.02, 28.61, 27.98, 21.77, 13.80 ppm; MS (APCI-DI^+^) *m*/*z* [M + H]^+^ 432 (100%); MS (ESI-DI^–^) *m*/*z* [M – H]^−^ 430 (100%); HRMS (ESI^+^) *m*/*z* [M + H]^+^ calcd: 432.16279 found: 432.16281, [M + Na]^+^ calcd: 454.14473 found: 454.14487, [2M + Na]^+^ calcd: 885.30024 found: 885.30044; HPLC (Method 1) retention time = 3.4 min, 96.8% (254 nm), 97.0% (280 nm), *t*_M_ (DMSO) = 1.1 min (MeCN/H_2_O 60:40); HPLC (Method 2) retention time = 11.4 min, 96.4%, *t*_M_ (DMSO) = 1.2 min, λ_max_ = 350 nm.

##### [6-Hydroxy-2-(4-hydroxyphenyl)benzo[*b*]thiophen-3-yl][2-(propylamino)phenyl]methanone (**11d**)

Following general procedure 5 using propylamine (800 μL, 9.75 mmol, 20 equiv) and (2-fluorophenyl)[6-hydroxy-2-(4-hydroxyphenyl)benzo[*b*]thiophen-3-yl]methanone (**10l**, 177 mg, 0.485 mmol, 1 equiv) in DMSO (8 mL) for 5 h. Extracted after the addition of 2 M HCl_aq_ (10 mL) with EtOAc (5 × 15 mL) and washed with brine/2 M HCl_aq_ (1:1) (6 × 50 mL). Purification by silica gel chromatography (*n*-hexane/EtOAc 4:1, 0.1% acetic acid) yielded a yellow solid (51 mg, 0.13 mmol, 26%): C_24_H_21_NO_3_S (403.50); mp 237–238 °C; IR (KBr) *ṽ* 3434 (OH), 3330 (NH), 1616 (C=O) cm^–1^; ^1^H NMR (500 MHz, C_2_D_6_OS) δ_H_ 9.73 (s, 2H), 8.97 (t, *J* = 5.5 Hz, 1H), 7.34–7.29 (m, 2H), 7.21–7.18 (m, 2H), 7.17–7.12 (m, 2H), 6.85–6.81 (m, 2H), 6.73–6.66 (m, 2H), 6.32 (ddd, *J* = 8.1, 7.0, 1.1 Hz, 1H), 3.28 (td, *J* = 7.3, 5.5 Hz, 2H), 1.68 (h, *J* = 7.3 Hz, 2H), 1.01 (t, *J* = 7.3 Hz, 3H) ppm; ^13^C NMR (126 MHz, C_2_D_6_OS) δ_C_ 196.31, 157.70, 155.34, 151.42, 138.97, 137.91, 135.88, 134.30, 132.43, 130.84, 129.21 (2C), 123.93, 123.12, 117.20, 115.72 (2C), 115.14, 114.01, 111.82, 107.09, 43.56, 21.75, 11.40 ppm; MS (ESI-DI^–^) *m*/*z* [M – H]^−^ 402 (100%); HRMS (ESI^+^) *m*/*z* [M + H]^+^ calcd: 404.13149 found: 404.13159; [M + Na]^+^ calcd: 426.11343 found: 426.11356, [2M + Na]^+^ calcd: 829.23764 found: 829.23783; HPLC (Method 1) retention time = 8.0 min, 96.9% (254 nm), 97.8% (280 nm), *t*_M_ (DMSO) = 1.1 min (MeCN/H_2_O = 50:50); HPLC (Method 2) retention time = 11.6 min, 97.8%, *t*_M_ (DMSO) = 1.2 min, λ_max_ = 264, 304 nm.

##### [6-Hydroxy-2-(4-hydroxyphenyl)benzo[*b*]thiophen-3-yl][2-(isobutylamino)phenyl]methanone (**11e**)

Following general procedure 5 using isobutylamine (670 μL, 6.74 mmol, 20 equiv) and (2-fluorophenyl)[6-hydroxy-2-(4-hydroxyphenyl)benzo[*b*]thiophen-3-yl]methanone (**10l**, 123 mg, 0.338 mmol, 1.0 equiv) in DMSO (8 mL) for 5 h. Extracted after the addition of 0.5 M HCl_aq_ (5 mL) with EtOAc (3 × 20 mL) and washed with brine (3 × 20 mL). Purification by silica gel chromatography (*n*-hexane/EtOAc 3:2) yielded a yellow solid (83 mg, 0.20 mmol, 59%): C_25_H_23_NO_3_S (417.52); mp >228 °C (dec.); IR (KBr) *ṽ* 3337 (OH/NH), 1617 (C=O) cm^–1^; ^1^H NMR (500 MHz, C_2_D_6_OS) δ_H_ 9.74 (s, 1H), 9.70 (s, 1H), 9.08 (t, *J* = 5.6 Hz, 1H), 7.34–7.28 (m, 2H), 7.21–7.17 (m, 2H), 7.16–7.12 (m, 2H), 6.86–6.82 (m, 2H), 6.72–6.65 (m, 2H), 6.31 (ddd, *J* = 8.1, 7.0, 1.0 Hz, 1H), 3.16 (dd, *J* = 6.7, 5.6 Hz, 2H), 1.97 (hept, *J* = 6.7 Hz, 1H), 1.01 (d, *J* = 6.7 Hz, 6H) ppm; ^13^C NMR (126 MHz, C_2_D_6_OS) δ_C_ 196.29, 157.59, 155.25, 151.48, 138.87, 137.81, 135.75, 134.17, 132.33, 130.73, 129.09 (2C), 123.83, 122.99, 117.13, 115.59 (2C), 115.05, 113.87, 111.80, 107.00, 49.28, 27.26, 19.98 (2C) ppm; MS (ESI-DI^–^) *m*/*z* [M – H]^−^ 416 (100%); HRMS (ESI^+^) *m*/*z* [M + H]^+^ calcd: 418.14714 found: 418.14728, [M + Na]^+^ calcd: 440.12908 found: 440.12919, [2M + Na]^+^ calcd: 857.26894 found: 857.26926; HPLC (Method 1) retention time = 4.5 min, 99.0% (254 nm), 99.0% (280 nm), *t*_M_ (DMSO) = 1.1 min (MeCN/H_2_O 60:40); HPLC (Method 2) retention time = 12.0 min, 98.6%, *t*_M_ (DMSO) = 1.2 min, λ_max_ = 271, 306 nm.

##### [6-Hydroxy-2-(4-hydroxyphenyl)benzo[*b*]thiophen-3-yl][2-(pentylamino)phenyl]methanone (**11f**)

Following general procedure 5 using 1-pentylamine (580 μL, 5 mmol, 20 equiv) and (2-fluorophenyl)[6-hydroxy-2-(4-hydroxyphenyl)benzo[*b*]thiophen-3-yl]methanone (**10l**, 92 mg, 0.25 mmol, 1eq) in DMSO (8 mL) for 5 h. Extracted after the addition of 0.5 M HCl_aq_ (5 mL) with EtOAc (3 × 20 mL) and washed with brine (3 × 20 mL). Purification by silica gel chromatography (*n*-hexane/EtOAc 2:1 yielded a yellow solid (66 mg, 0.15 mmol, 61%): C_26_H_25_NO_3_S (431.55); mp >225 °C (dec.); IR (KBr) *ṽ* 3323 (NH/OH), 1617 (C=O) cm^–1^; ^1^H NMR (500 MHz, C_2_D_6_OS) δ_H_ 9.73 (s, 1H), 9.71 (s, 1H), 8.96 (t, *J* = 5.4 Hz, 1H), 7.35–7.28 (m, 2H), 7.21–7.17 (m, 2H), 7.16–7.12 (m, 2H), 6.85–6.81 (m, 2H), 6.72–6.66 (m, 2H), 6.32 (ddd, *J* = 8.1, 6.9, 1.0 Hz, 1H), 3.30 (td, *J* = 7.1, 5.4 Hz, 2H), 1.67 (p, *J* = 7.1 Hz, 2H), 1.46–1.32 (m, 4H), 0.92 (t, *J* = 7.1 Hz, 3H) ppm; ^13^C NMR (126 MHz, C_2_D_6_OS) δ_C_ 196.29, 157.70, 155.34, 151.40, 138.97, 137.91, 135.89, 134.30, 132.43, 130.83, 129.21 (2C), 123.93, 123.12, 117.18, 115.70 (2C), 115.12, 114.00, 111.80, 107.09, 41.82, 28.73, 28.14, 21.87, 13.95 ppm; elemental analysis [%] calcd: C 72.36, H 5.84, N 3.25, found: C 72.40, H 6.22, N 2.81; MS (ESI-DI^–^) *m*/*z* [M – H]^−^ 430 (100%); HRMS (ESI^+^) *m*/*z* [M + Na]^+^ calcd: 454.14473 found: 454.14488, [2M + Na]^+^ calcd: 885.30024 found: 885.30027; HPLC (Method 1) retention time = 6.3 min, 98.8% (254 nm), 99.5% (280 nm), *t*_M_ (DMSO) = 1.1 min (MeCN/H_2_O 60:40); HPLC (Method 2) retention time = 12.4 min, 97.7%, *t*_M_ (DMSO) = 1.2 min, λ_max_ = 263, 306 nm.

##### [6-Hydroxy-2-(4-hydroxyphenyl)benzo[*b*]thiophen-3-yl]{2-[(2-morpholinoethyl)amino]phenyl}methanone (**11g**)

Following general procedure 5 using 4-(2-aminoethyl)-morpholine (793 μL, 6.09 mmol, 20 equiv) and (2-fluorophenyl)[6-hydroxy-2-(4-hydroxyphenyl)benzo[*b*]thiophen-3-yl]methanone (**10l**, 111 mg, 0.304 mmol, 1.0 equiv) in DMSO (8 mL) for 5 h. H_2_O (20 mL) was added and the mixture was acidified with HCl_aq_ (pH = 1), followed by washing with CH_2_Cl_2_ (30 mL) and methanol (2 mL). Extracted with EtOAc (6 × 20 mL) after alkalization to pH = 9 with NH_3_ and washed with brine (3 × 20 mL). Purification by silica gel chromatography (EtOAc) yielded a yellow solid (64 mg, 0.14 mmol, 46%): C_27_H_26_N_2_O_4_S (474.58); mp >104 °C (dec.); IR (KBr) *ṽ* 3391 (OH/NH), 1615 (C=O) cm^–1^; ^1^H NMR (500 MHz, C_2_D_6_OS) δ_H_ 9.73 (s, 1H), 9.70 (s, 1H), 9.09 (t, *J* = 5.0 Hz, 1H), 7.36–7.28 (m, 2H), 7.22–7.18 (m, 2H), 7.15 (dd, *J* = 8.1, 1.6 Hz, 1H), 7.12 (d, *J* = 8.7 Hz, 1H), 6.83 (m, 2H), 6.72–6.65 (m, 2H), 6.33 (ddd, *J* = 8.1, 6.9, 1.0 Hz, 1H), 3.61 (t, *J* = 4.6 Hz, 4H), 3.39 (q, *J* = 6.3, 5.0 Hz, 2H), 2.65 (t, *J* = 6.3 Hz, 2H), 2.47 (t, *J* = 4.6 Hz, 4H) ppm; ^13^C NMR (126 MHz, C_2_D_6_OS) δ_C_ 196.02, 157.68, 155.32, 151.17, 138.96, 137.81, 135.76, 134.19, 132.44, 130.96, 129.18 (2C), 123.94, 123.09, 117.57, 115.67 (2C), 115.11, 114.10, 112.06, 107.09, 66.28 (2C), 56.03, 53.00 (2C), 38.62 ppm, the signal at 38.62 ppm is overlaid by the DMSO solvent signal and only visible in ^13^C-DEPT NMR (negative); MS (ESI-DI^+^) *m*/*z* [M + H]^+^ 475 (100%), [M + Na]^+^ 497 (70%); MS (ESI-DI^–^) *m*/*z* [M – H]^−^ 473 (100%); HRMS (ESI^+^) *m*/*z* [M + H]^+^ calcd: 475.16861 found: 475.16915, [M + Na]^+^ calcd: 497.15055 found: 497.15074; [2M + Na]^+^ calcd: 971.31188 found: 971.31269; HPLC (Method 3) retention time = 4.1 min, 99.7% (254 nm), 99.8% (280 nm), *t*_M_ (DMSO) = 1.1 min (MeCN/buffer 30:70), λ_max_ = 264, 304 nm.

##### [4-(Cyclopentyloxy)phenyl][6-methoxy-2-(4-methoxyphenyl)benzo[*b*]thiophen-3-yl]methanone (**12a**)

Following general procedure 4 using cyclopentyl alcohol (680 μL, 7.50 mmol, 5 equiv), sodium hydride (60% in mineral oil, 603 mg, 15.1 mmol, 10 equiv) in DMF (5 mL), and (4-fluorophenyl)[6-methoxy-2-(4-methoxyphenyl)benzo[*b*]thiophen-3-yl]methanone (**9c**, 590 mg, 1.50 mmol, 1 equiv) in DMF (15 mL) at ambient temperature. The reaction was stopped by the addition of H_2_O (20 mL) after 2.5 h. Extracted with EtOAc (3 × 20 mL) and washed with brine (3 × 20 mL). Without further purification, a yellow powder (694 mg, 1.51 mmol, quant.) was obtained. A fraction of the crude product (183 mg, 0.40 mmol) was further purified by recrystallization from ethanol (1.0 mL) yielding yellow crystals (108 mg, 0.236 mmol): C_28_H_26_O_4_S (458.57); mp 95–97 °C (ethanol); IR (KBr) *ṽ* 2956 (aliph. CH), 1633 (C=O), 1251 (Ar–O-Me) cm^–1^; ^1^H NMR (500 MHz, C_2_D_6_OS) δ_H_ 7.67–7.62 (m, 3H), 7.35 (dd, *J* = 8.8, 0.4 Hz, 1H), 7.33–7.29 (m, 2H), 7.00 (dd, *J* = 8.9, 2.4 Hz, 1H), 6.91–6.85 (m, 4H), 4.87–4.82 (m, 1H), 3.84 (s, 3H), 3.71 (s, 3H), 1.94–1.84 (m, 2H), 1.71–1.61 (m, 4H), 1.60–1.52 (m, 2H) ppm; ^13^C NMR (126 MHz, C_2_D_6_OS) δ_C_ 192.19, 162.05, 159.41, 157.29, 140.77, 139.25, 133.10, 131.72 (2C), 130.10, 129.61 (2C), 129.10, 125.13, 123.25, 115.19 (2C), 114.95, 114.26 (2C), 105.06, 79.24, 55.47, 55.09, 32.07 (2C), 23.44 (2C) ppm; elemental analysis (%) calcd: C 73.34, H 5.72, found: C 73.23, H 5.60; MS (APCI-DI^+^) *m*/*z* [M + H]^+^ 459 (100%); MS (APCI-DI^–^) *m*/*z* [M-69]^−^ 389 (16%), [M-15]^−^ 443 (100%); HPLC (Method 1) retention time = 6.0 min, 97.2% (254 nm), 97.6% (280 nm), *t*_M_ (DMSO) = 1.2 min (MeCN/H_2_O 80:20); HPLC (Method 2) retention time = 15.3 min, 99.2%, *t*_M_ (DMSO) = 1.3 min, λ_max_ = 303 nm.

##### (4-Isobutoxyphenyl)[6-methoxy-2-(4-methoxyphenyl)benzo[*b*]thiophen-3-yl]methanone (**12b**)

Following general procedure 4 using isobutanol (830 μL, 8.96 mmol, 6 equiv), sodium hydride (60% in mineral oil, 607 mg, 15.2 mmol, 10 equiv) in DMF (5 mL), and (4-fluorophenyl)[6-methoxy-2-(4-methoxyphenyl)benzo[*b*]thiophen-3-yl]methanone (**9c**, 590 mg, 1.50 mmol, 1 equiv) in DMF (15 mL) at ambient temperature. The reaction was stopped by the addition of H_2_O (20 mL) after 2.5 h. Extracted with EtOAc (3 × 20 mL) and washed with brine (3 × 20 mL). Without further purification, a yellow powder (690 mg, 1.55 mmol, quant.) was obtained. A fraction of the crude product (243 mg, 0.54 mmol) was purified by recrystallization from ethanol (2.0 mL) yielding yellow crystals (129 mg, 0.288 mmol): C_27_H_26_O_4_S (446.56); mp 100–101 °C (ethanol); IR (KBr) *ṽ* 2958, 2927, 2834 (aliph. CH), 1631 (C=O), 1249 (Ar–O-Me) cm^–1^; ^1^H NMR (600 MHz, C_2_D_6_OS) δ_H_ 7.69–7.66 (m, 2H), 7.66 (dd, *J* = 2.5, 0.5 Hz, 1H), 7.33 (dd, *J* = 8.9, 0.5 Hz, 1H), 7.33–7.30 (m, 2H), 7.00 (dd, *J* = 8.9, 2.4 Hz, 1H), 6.94–6.91 (m, 2H), 6.91–6.88 (m, 2H), 3.84 (s, 3H), 3.77 (d, *J* = 6.5 Hz, 2H), 3.72 (s, 3H), 1.98 (hept, *J* = 6.7 Hz, 1H), 0.94 (d, *J* = 6.7 Hz, 6H) ppm; ^13^C NMR (151 MHz, C_2_D_6_OS) δ_C_ 192.25, 163.11, 159.40, 157.29, 140.60, 139.27, 133.10, 131.76 (2C), 130.09, 129.56 (2C), 129.36, 125.11, 123.20, 114.96, 114.45 (2C), 114.31 (2C), 105.08, 73.89, 55.47, 55.11, 27.45, 18.77 (2C) ppm; elemental analysis (%) calcd: C 72.62, H 5.87, found: C 72.65, H 5.75; MS (APCI-DI^+^) *m*/*z* [M + H]^+^ 448 (100%); MS (APCI-DI^–^) *m*/*z* [M-56]^−^ 389 (45%), [M-15]^−^ 431 (100%); HPLC (Method 1) retention time = 5.7 min, 97.7% (254 nm), 98.0% (280 nm), *t*_M_ (DMSO) = 1.1 min (MeCN/H_2_O 80:20); HPLC (Method 2) retention time = 14.7 min, 99.0%, *t*_M_ (DMSO) = 1.3 min, λ_max_ = 301 nm.

##### [6-Methoxy-2-(4-methoxyphenyl)benzo[*b*]thiophen-3-yl][4-(pentyloxy)phenyl]methanone (**12c**)

Following general procedure 4 using pentyl alcohol (810 μL, 7.53 mmol, 5 equiv), sodium hydride (60% in mineral oil, 604 mg, 15.1 mmol, 10 equiv) in DMF (15 mL), and (4-fluorophenyl)[6-methoxy-2-(4-methoxyphenyl)benzo[*b*]thiophen-3-yl]methanone (**9c**, 589 mg, 1.50 mmol, 1 equiv) in DMF (5 mL) at ambient temperature. The reaction was stopped by the addition of H_2_O (20 mL) after 2.5 h. Extracted with EtOAc (3 × 20 mL) and washed with brine (3 × 20 mL). Without further purification, yellow crystals (633 mg, 1.37 mmol, 92%) were obtained: C_28_H_28_O_4_S (460.59); mp 58–60 °C; IR (KBr) *ṽ* 2924, 2924 (aliph. CH), 1633 (C=O), 1246 (Ar–O-Me) cm^–1^; ^1^H NMR (500 MHz, C_2_D_6_OS) δ_H_ 7.69–7.64 (m, 3H), 7.33 (dd, *J* = 8.8, 0.5 Hz, 1H), 7.34–7.28 (m, 2H), 7.00 (dd, *J* = 8.9, 2.4 Hz, 1H), 6.94–6.87 (m, 4H), 3.98 (t, *J* = 6.5 Hz, 2H), 3.84 (s, 3H), 3.71 (s, 3H), 1.73–1.63 (m, 2H), 1.41–1.27 (m, 4H), 0.86 (t, *J* = 7.1 Hz, 3H) ppm; ^13^C NMR (126 MHz, C_2_D_6_OS) δ_C_ 192.26, 163.03, 159.41, 157.29, 140.60, 139.27, 133.10, 131.75 (2C), 130.08, 129.57 (2C), 129.34, 125.11, 123.20, 114.95, 114.42 (2C), 114.31 (2C), 105.08, 67.80, 55.47, 55.11, 28.00, 27.44, 21.69, 13.74 ppm; MS (APCI-DI^+^) *m*/*z* [M + H]^+^ 461 (100%); MS (APCI-DI^–^) *m*/*z* [M-71]^−^ 389 (100%), [M-15]^−^ 445 (91%); HRMS (ESI^+^) *m*/*z* [M + H]^+^ calcd: 461.17811 found: 461.17852, [M + Na]^+^ calcd: 483.16005 found: 483.16036, [2M + Na]^+^ calcd: 943.33088 found: 943.33213; HPLC (Method 1) retention time = 6.5 min, 96.1% (254 nm), 96.4% (280 nm), *t*_M_ (DMSO) = 1.2 min (MeCN/H_2_O 80:20); HPLC (Method 2) retention time = 15.4 min, 99.1%, *t*_M_ (DMSO) = 1.2 min, λ_max_ = 278, 302 nm.

##### (2-Isobutoxyphenyl)[6-methoxy-2-(4-methoxyphenyl)benzo[*b*]thiophen-3-yl]methanone (**12d**)

Following general procedure 4 using isobutanol (0.48 mL, 5.2 mmol, 5.0 equiv), sodium hydride (60% in mineral oil, 0.413 g, 10.3 mmol, 10 equiv) in DMF (15 mL), and (2-fluorophenyl)[6-methoxy-2-(4-methoxyphenyl)benzo[*b*]thiophen-3-yl]methanone (**9l**, 407 mg, 1.04 mmol, 1.0 equiv) in DMF (5 mL) at ambient temperature. The reaction was stopped by the addition of H_2_O (20 mL) after 2.5 h. Extracted with EtOAc (3 × 20 mL) and washed with brine (3 × 20 mL). Purification by silica gel chromatography (EtOAc) followed by recrystallization (ethanol, 2 mL) yielded yellow crystals (86 mg, 0.19 mmol, 37%): C_27_H_26_O_4_S (446.56); mp 100–101 °C (ethanol); IR (KBr) *ṽ* 1626 (C=O), 1250 (Ar–O-Me) cm^–1^; ^1^H NMR (500 MHz, C_2_D_6_OS) δ_H_ 7.60 (d, *J* = 2.4 Hz, 1H), 7.58 (d, *J* = 8.9 Hz, 1H), 7.44 (dd, *J* = 7.5, 1.8 Hz, 1H), 7.34 (ddd, *J* = 8.4, 7.4, 1.8 Hz, 1H), 7.25–7.19 (m, 2H), 7.01 (dd, *J* = 8.9, 2.4 Hz, 1H), 6.90 (ddd, *J* = 7.5, 7.4, 0.9 Hz, 1H), 6.83–6.76 (m, 3H), 3.83 (s, 3H), 3.70 (s, 3H), 3.47 (d, *J* = 6.3 Hz, 2H), 1.49 (n, *J* = 6.7 Hz, 1H), 0.57 (d, *J* = 6.7 Hz, 6H); ^13^C NMR (126 MHz, C_2_D_6_OS) δ_C_ 191.92, 159.38, 157.28, 157.12, 144.82, 139.17, 133.59, 132.58, 132.24, 130.50, 130.36 (2C), 128.73, 125.04, 123.52, 120.00, 114.91, 113.69 (2C), 112.23, 104.81, 73.94, 55.48, 55.17, 27.62, 18.52 (2C) ppm; elemental analysis [%] calcd: C 72.62, H 5.87, found: C 72.75, H 5.67; MS (APCI-DI^+^) *m*/*z* [M-55]^+^ 391 (100%), [M + H]^+^ 447 (91%); HPLC (Method 1) retention time = 8.8 min, 98.9% (254 nm), 99.1% (280 nm), *t*_M_ (DMSO) = 1.1 min (MeCN/H_2_O = 70:30); HPLC (Method 2) retention time = 14.5 min, 99.0%, *t*_M_ (DMSO) = 1.2 min, λ_max_ = 268, 304 nm.

##### [6-Hydroxy-2-(4-hydroxyphenyl)benzo[*b*]thiophen-3-yl](4-hydroxyphenyl)methanone (**13a**)

Following general procedure 2 using [4-(cyclopentyloxy)phenyl][6-methoxy-2-(4-methoxyphenyl)benzo[*b*]thiophen-3-yl]methanone (**12a**, 358 mg, 0.781 mmol, 1 equiv) and boron tribromide (1 M in CH_2_Cl_2_, 3 mL, 3 mmol, 3 equiv) in CH_2_Cl_2_ (10 mL). The reaction was stopped by the addition of H_2_O (10 mL) after 4 h. Extracted after the addition of methanol (10 mL) and CH_2_Cl_2_ (20 mL) with 10% methanol in CH_2_Cl_2_ (3 × 10 mL). Purification by silica gel chromatography (EtOAc/*n*-hexane 1:1) and following recrystallization (CH_2_Cl_2_/EtOAc 1:1, 2 mL) yielded a yellow solid (63 mg, 0.17 mmol, 22%): C_21_H_14_O_4_S (362.40); mp >167 °C (dec.) [lit. 135 °C];^73^ IR (KBr) *ṽ* 3273 (OH), 1586 (C=O) cm^–1^; ^1^H NMR (500 MHz, C_2_D_6_OS) δ_H_ 10.42 (s, 1H), 9.75 (s, 1H), 9.71 (s, 1H), 7.61–7.54 (m, 2H), 7.33 (dd, *J* = 2.3, 0.3 Hz, 1H), 7.25 (dd, *J* = 8.7, 0.3 Hz, 1H), 7.19–7.15 (m, 2H), 6.85 (dd, *J* = 8.7, 2.3 Hz, 1H), 6.73–6.70 (m, 2H), 6.69–6.66 (m, 2H) ppm; ^13^C NMR (126 MHz, C_2_D_6_OS) δ_C_ 192.28, 162.45, 157.66, 155.26, 139.87 (2C), 139.10, 132.27, 132.00, 129.84, 129.57 (2C), 128.28, 123.78, 123.25, 115.55 (2C), 115.28 (2C), 115.03, 106.97 ppm; MS (ESI-DI^–^) *m*/*z* [M – H]^−^ 361 (100%); HRMS (ESI^+^) *m*/*z* [M + H]^+^ calcd: 363.06856 found: 363.06856, [M + Na]^+^ calcd: 385.05050 found: 385.05061, [2M + Na]^+^ calcd: 747.11178 found: 747.11198; HPLC (Method 1) retention time = 3.3 min, 97.3% (254 nm), 97.2% (280 nm), *t*_M_ (DMSO) = 1.1 min (MeCN/H_2_O 40:60); HPLC (Method 2) retention time = 9.1 min, 97.0%, *t*_M_ (DMSO) = 1.2 min, λ_max_ = 298 nm.

The compound is described in the literature.^73^

##### [6-Hydroxy-2-(4-hydroxyphenyl)benzo[*b*]thiophen-3-yl](4-isobutoxyphenyl)methanone (**13b**)

Following general procedure 2 using (4-isobutoxyphenyl)[6-methoxy-2-(4-methoxyphenyl)benzo[*b*]thiophen-3-yl]methanone (**12b**, 447 mg, 1.00 mmol, 1.0 equiv) and boron tribromide (1 M in CH_2_Cl_2_, 3 mL, 3 mmol, 3 equiv) in CH_2_Cl_2_ (10 mL). The reaction was stopped by the addition of H_2_O (10 mL) after 4 h. Extracted after the addition of methanol (2.5 mL) and CH_2_Cl_2_ (10 mL) with 10% methanol in CH_2_Cl_2_ (3 × 10 mL). Purification by silica gel chromatography (EtOAc/*n*-hexane 1:2) and following recrystallization (toluene/ethanol 10:1, 5.5 mL) yielded a pale-yellow solid (124 mg, 0.297 mmol, 30%): C_25_H_22_O_4_S (418.51); mp 144–145 °C; IR (KBr) *ṽ* 3340 (OH), 2959 (aliph. CH), 1594 (C=O) cm^–1^; ^1^H NMR (500 MHz, C_2_D_6_OS) δ_H_ 9.76 (s, 1H), 9.72 (s, 1H), 7.67–7.63 (m, 2H), 7.34 (dd, *J* = 2.3, 0.5 Hz, 1H), 7.24 (d, *J* = 8.8 Hz, 1H), 7.20–7.15 (m, 2H), 6.93–6.89 (m, 2H), 6.85 (dd, *J* = 8.7, 2.3 Hz, 1H), 6.70–6.66 (m, 2H), 3.76 (d, *J* = 6.5 Hz, 2H), 1.98 (hept, *J* = 6.6 Hz, 1H), 0.94 (d, *J* = 6.7 Hz, 6H) ppm; ^13^C NMR (126 MHz, C_2_D_6_OS) δ_C_ 192.44, 162.98, 157.71, 155.30, 140.17, 139.11, 132.17, 131.70 (2C), 129.62, 129.57 (2C), 129.46, 123.67, 123.19, 115.58 (2C), 115.08, 114.34 (2C), 107.00, 73.87, 27.46, 18.79 (2C) ppm; elemental analysis [%] calcd: C 71.75, H 5.30, found: C 71.47, H 5.14; MS (ESI-DI^–^) *m*/*z* [M – H]^−^ 417 (100%); HPLC (Method 1) retention time = 4.1 min, 99.1% (254 nm), 99.5% (280 nm), *t*_M_ (DMSO) = 1.1 min (MeCN/H_2_O 60:40); HPLC (Method 2) retention time = 11.7 min, 97.5%, *t*_M_ (DMSO) = 1.3 min, λ_max_ = 297 nm.

##### [6-Hydroxy-2-(4-hydroxyphenyl)benzo[*b*]thiophen-3-yl][4-(pentyloxy)phenyl]methanone (**13c**)

Following general procedure 2 using [6-methoxy-2-(4-methoxyphenyl)benzo[*b*]thiophen-3-yl][4-(pentyloxy)phenyl]methanone (**12c**, 461 mg, 1.00 mmol, 1 equiv) and boron tribromide (1 M in CH_2_Cl_2_, 3 mL, 3 mmol, 3 equiv) in CH_2_Cl_2_ (10 mL). The reaction was stopped by the addition of H_2_O (10 mL) after 4 h. Extracted after the addition of methanol (10 mL) and CH_2_Cl_2_ (2.5 mL) with 10% methanol in CH_2_Cl_2_ (3 × 10 mL). Purification by silica gel chromatography (EtOAc/*n*-hexane 1:2) followed by crystallization from *n*-hexane and CH_2_Cl_2_ (3 mL) under sonication yielded yellow crystals (168 mg, 0.388 mmol, 39%): C_26_H_24_O_4_S (432.53); mp 157–159 °C; IR (KBr) *ṽ* 3317 (OH), 2953, 2933, 2866 (aliph. CH), 1597 (C=O) cm^–1^; ^1^H NMR (500 MHz, C_2_D_6_OS) δ_H_ 9.76 (s, 1H), 9.72 (s, 1H), 7.67–7.63 (m, 2H), 7.34 (dd, *J* = 2.3, 0.5 Hz, 1H), 7.25 (dd, *J* = 8.7, 0.5 Hz, 1H), 7.19–7.16 (m, 2H), 6.91–6.88 (m, 2H), 6.85 (dd, *J* = 8.7, 2.3 Hz, 1H), 6.69–6.66 (m, 2H), 3.98 (t, *J* = 6.5 Hz, 2H), 1.71–1.65 (m, 2H), 1.39–1.27 (m, 4H), 0.87 (t, *J* = 7.1 Hz, 3H) ppm; ^13^C NMR (151 MHz, C_2_D_6_OS) δ_C_ 192.45, 162.90, 157.71, 155.30, 140.17, 139.11, 132.18, 131.70 (2C), 129.62, 129.57 (2C), 129.44, 123.67, 123.20, 115.58 (2C), 115.08, 114.30 (2C), 107.00, 67.78, 28.02, 27.45, 21.71, 13.75 ppm; HRMS (ESI^+^) *m*/*z* [M + H]^+^ calcd: 433.14681 found: 433.14728, [M + Na]^+^ calcd: 455.12875 found: 455.12924, [M + K]^+^ calcd: 471.10269 found: 471.10277, [2M + Na]^+^ calcd: 887.26828 found: 887.26964; MS (ESI-DI^–^) *m*/*z* [M – H]^−^ 431 (100%); HPLC (Method 1) retention time = 5.6 min, 96.0% (254 nm), 96.5% (280 nm), *t*_M_ (DMSO) = 1.2 min (MeCN/H_2_O 60:40); HPLC (Method 2) retention time = 12.2 min, 97.1%, *t*_M_ (DMSO) = 1.1 min, λ_max_ = 299 nm.

##### [6-Hydroxy-2-(4-hydroxyphenyl)benzo[*b*]thiophen-3-yl](2-hydroxyphenyl)methanone (**13d**)

Following general procedure 2 using (2-isobutoxyphenyl)[6-methoxy-2-(4-methoxyphenyl)benzo[*b*]thiophen-3-yl]methanone (**12d**, 187 mg, 0.419 mmol, 1.0 equiv) and boron tribromide (1 M in CH_2_Cl_2_, 1.5 mL, 1.5 mmol, 3.6 equiv) in CH_2_Cl_2_ (10 mL). The reaction was stopped by the addition of H_2_O (10 mL) after 1 h. Extracted after the addition of CH_2_Cl_2_ (20 mL) and methanol (5 mL) with 10% methanol in CH_2_Cl_2_ (3 × 20 mL) and washed with brine/2 M HCl_aq_ (1:1, 2 × 20 mL). Purification by silica gel chromatography (EtOAc/*n*-hexane 2:3) yielded a yellow oil, which crystallized upon layering with CH_2_Cl_2_ 131 mg, 0.362 mmol, 86%): C_21_H_14_O_4_S (362.40); mp 217–218 °C; IR (KBr) *ṽ* 3361 (OH), 1612 (C=O) cm^–1^; ^1^H NMR (500 MHz, C_2_D_6_OS) δ_H_ 11.51 (s, 1H), 9.79 (s, 1H), 9.74 (s, 1H), 7.42 (ddd, *J* = 8.4, 7.2, 1.7 Hz, 1H), 7.37 (d, *J* = 8.7 Hz, 1H), 7.35 (d, *J* = 2.3 Hz, 1H), 7.25 (dd, *J* = 8.0, 1.7 Hz, 1H), 7.21–7.16 (m, 2H), 6.93 (dd, *J* = 8.4, 1.0 Hz, 1H), 6.88 (dd, *J* = 8.7, 2.3 Hz, 1H), 6.73–6.66 (m, 3H) ppm; ^13^C NMR (126 MHz, C_2_D_6_OS) δ_C_ 198.18, 160.91, 157.99, 155.48, 141.87, 139.22, 136.47, 132.42, 131.80, 129.82 (2C), 128.77, 123.52, 123.25, 121.18, 119.17, 117.52, 115.69 (2C), 115.35, 107.11 ppm; MS (ESI-DI^–^) *m*/*z* [M-121]^−^ 241 (92%), [M – H]^−^ 361 (100%); HRMS (ESI^+^) *m*/*z* [M + H]^+^ calcd: 363.06856 found: 363.06875 (26%), [M + Na]^+^ calcd: 385.05050 found: 385.05069 (100%), [2M + Na]^+^ calcd: 747.11178 found: 747.11200 (86%); HPLC (Method 1) retention time = 8.8 min, 98.9% (254 nm), 99.1% (280 nm), *t*_M_ (DMSO) = 1.1 min (MeCN/H_2_O 70:30); HPLC (Method 2) retention time = 14.5 min, 99.0%, *t*_M_ (DMSO) = 1.2 min, λ_max_ = 262, 304 nm.

##### [6-Hydroxy-2-(4-hydroxyphenyl)benzo[*b*]thiophen-3-yl](2-methoxyphenyl)methanone (**13e**)

Following general procedure 4 using methanol (0.06 mL, 1 mmol, 5 equiv), sodium hydride (104 mg, 2.60 mmol, 10 equiv, 60% in mineral oil) in DMF (5 mL) and (2-fluorophenyl)[6-hydroxy-2-(4-hydroxyphenyl)benzo[*b*]thiophen-3-yl]methanone (**10l**, 99 mg, 0.27 mmol, 1.0 equiv) in DMF (2 mL) at ambient temperature. The reaction was stopped by the addition of H_2_O (15 mL) after 0.5 h and acidified with 2 M HCl_aq_ (2 mL). Extracted with EtOAc (3 × 20 mL) and washed with brine/2 M HCl_aq_ (7 × 15 mL). Purification by silica gel chromatography (EtOAc/*n*-hexane 1:1) yielded a yellow oil, which crystallized upon layering with *n*-hexane (85 mg, 0.23 mmol, 83%): C_22_H_16_O_4_S (376.43); mp 196–197 °C; IR (KBr) *ṽ* 3390 (OH), 1627 (C=O) cm^–1^; ^1^H NMR (500 MHz, C_2_D_6_OS) δ_H_ 9.72 (s, 1H), 9.60 (s, 1H), 7.67 (d, *J* = 8.8 Hz, 1H), 7.38–7.24 (m, 3H), 7.10–7.03 (m, 2H), 6.90 (dd, *J* = 8.8, 2.3 Hz, 1H), 6.86–6.79 (m, 2H), 6.59–6.53 (m, 2H), 3.54 (s, 3H) ppm; ^13^C NMR (126 MHz, C_2_D_6_OS) δ_C_ 191.67, 157.73, 157.63, 155.24, 145.79, 138.90, 133.16, 131.69, 130.90, 130.36, 130.34 (2C), 128.94, 123.98, 123.51, 119.93, 115.22, 114.85 (2C), 111.77, 106.86, 55.62 ppm; elemental analysis [%] calcd: C 70.20, H 4.28, found: C 70.54, H 4.40; MS (ESI-DI^–^) *m*/*z* [M – H]^−^ 375 (100%); HRMS (ESI^+^) *m*/*z* [M + H]^+^ calcd: 377.08421 found: 377.08434, [M + Na]^+^ calcd: 399.06615 found: 399.06624, [2M + Na]^+^ calcd: 775.14308 found: 775.14329; HPLC (Method 1) retention time = 5.6 min, 99.5% (254 nm), 99.7% (280 nm), *t*_M_ (DMSO) = 1.1 min (MeCN/H_2_O 40:60); HPLC (Method 2) retention time = 9.9 min, 95.8%, *t*_M_ (DMSO) = 1.2 min, λ_max_ = 267, 308 nm.

##### [6-Hydroxy-2-(4-hydroxyphenyl)benzo[*b*]thiophen-3-yl](2-isobutoxyphenyl)methanone (**13f**)

Following general procedure 4 using isobutanol (0.12 mL, 1.3 mmol, 5 equiv), sodium hydride (60% in mineral oil, 104 mg, 2.60 mmol, 10 equiv) in DMF (5 mL) and (2-fluorophenyl)[6-hydroxy-2-(4-hydroxyphenyl)benzo[*b*]thiophen-3-yl]methanone (**10l**, 94 mg, 0.26 mmol, 1 equiv) in DMF (2 mL) at 50 °C. The reaction was stopped by the addition of H_2_O (10 mL) after 2 h and acidified with 2 M HCl_aq_ (5 mL). Extracted with EtOAc (3 × 20 mL) and washed with brine/2 M HCl_aq_ (7 × 20 mL). Purification by silica gel chromatography (EtOAc/*n*-hexane 1:1) yielded a yellow oil, which crystallized upon layering with *n*-hexane (92 mg, 0.22 mmol, 85%): C_25_H_22_O_4_S (418.51); mp 174–175 °C; IR (KBr) *ṽ* 3326 (OH), 1617 (C=O) cm^–1^; ^1^H NMR (500 MHz, C_2_D_6_OS) δ_H_ 9.66 (s, 1H), 9.59 (s, 1H), 7.53 (d, *J* = 8.8 Hz, 1H), 7.40 (dd, *J* = 7.6, 1.8 Hz, 1H), 7.32 (ddd, *J* = 8.4, 7.3, 1.8 Hz, 1H), 7.28 (d, *J* = 2.3 Hz, 1H), 7.11–7.04 (m, 2H), 6.89–6.84 (m, 2H), 6.79 (dd, *J* = 8.4, 0.9 Hz, 1H), 6.62–6.55 (m, 2H), 3.47 (d, *J* = 6.2 Hz, 2H), 1.52 (n, *J* = 6.7 Hz, 1H), 0.59 (d, *J* = 6.7 Hz, 6H) ppm; ^13^C NMR (126 MHz, C_2_D_6_OS) δ_C_ 192.12, 157.65, 157.26, 155.07, 144.51, 139.02, 133.36, 131.81, 131.69, 130.49, 130.32 (2C), 128.92, 123.56, 123.55, 119.86, 115.10, 114.95 (2C), 112.18, 106.80, 73.92, 27.65, 18.56 (2C) ppm; elemental analysis [%] calcd: C 71.75, H 5.30, found: C 71.55, H 5.52; MS (ESI-DI^–^) *m*/*z* [M – H]^−^ 417 (100%); HRMS (ESI^+^) *m*/*z* [M + Na]^+^ calcd: 441.11310 found: 441.11321, [2M + Na]^+^ calcd: 859.23698 found: 859.23723; HPLC (Method 1) retention time = 5.8 min, 98.3% (254 nm), 98.6% (280 nm), *t*_M_ (DMSO) = 1.1 min (MeCN/H_2_O 50:50); HPLC (Method 2) retention time = 11.1 min, 97.1%, *t*_M_ (DMSO) = 1.2 min, λ_max_ = 268, 306 nm.

##### [6-Hydroxy-2-(4-hydroxyphenyl)benzo[*b*]thiophen-3-yl][2-(pentyloxy)phenyl]methanone (**13g**)

Following general procedure 4 using pentyl alcohol (0.29 mL, 2.7 mmol, 5.7 equiv), sodium hydride (60% in mineral oil, 213 mg, 5.33 mmol, 11 equiv) in DMF (10 mL) and (2-fluorophenyl)[6-hydroxy-2-(4-hydroxyphenyl)benzo[*b*]thiophen-3-yl]methanone (**10l**, 170 mg, 0.470 mmol, 1.0 equiv) in DMF (5 mL) at ambient temperature for 2 h, followed by 4 h at 50 °C. The reaction was stopped by the addition of H_2_O (10 mL). Extracted with EtOAc (4 × 15 mL) after the addition of 2 M HCl_aq_ (10 mL) and washed with brine/2 M HCl_aq_ (7 × 15 mL). Purification by silica gel chromatography (EtOAc/*n*-hexane 1:1) yielded a yellow solid (86 mg, 0.20 mmol, 43%): C_26_H_24_O_4_S (432.53); mp 193–194 °C; IR (KBr) *ṽ* 3329 (OH), 1618 (C=O) cm^–1^; ^1^H NMR (500 MHz, C_2_D_6_OS) δ_H_ 9.65 (s, 1H), 9.59 (s, 1H), 7.50–7.43 (m, 2H), 7.33 (ddd, *J* = 8.4, 7.3, 1.8 Hz, 1H), 7.27 (dd, *J* = 2.3, 0.5 Hz, 1H), 7.11–7.04 (m, 2H), 6.89 (ddd, *J* = 7.4, 7.3, 0.9 Hz, 1H), 6.85 (dd, *J* = 8.8, 2.3 Hz, 1H), 6.78 (dd, *J* = 8.4, 0.9 Hz, 1H), 6.62–6.55 (m, 2H), 3.62 (t, *J* = 6.1 Hz, 2H), 1.25–1.14 (m, 2H), 1.05–0.94 (m, 2H), 0.89–0.79 (m, 2H), 0.66 (t, *J* = 7.3 Hz, 3H) ppm; ^13^C NMR (126 MHz, C_2_D_6_OS) δ_C_ 192.26, 157.62, 157.40, 155.12, 143.85, 138.96, 133.52, 132.01, 131.66, 130.48, 130.26 (2C), 128.74, 123.59, 123.43, 119.95, 114.98 (3C), 112.24, 106.78, 67.59, 28.29, 27.45, 21.89, 13.62 ppm; elemental analysis [%] calcd: C 72.20, H 5.59, found: C 71.78, H 5.59; MS (ESI-DI^–^) *m*/*z* 431 [M – H]^−^ (100%); HRMS (ESI^+^) *m*/*z* [M + Na]^+^ calcd: 455.12900 found: 455.12900, [2M + Na]^+^ calcd: 887.26828 found: 887.26880; HPLC (Method 1) retention time = 7.8 min, 99.2% (254 nm), 99.6% (280 nm), *t*_M_ (DMSO) = 1.1 min (MeCN/H_2_O 50:50); HPLC (Method 2) retention time = 11.6 min, 96.6%, *t*_M_ (DMSO) = 1.2 min, λ_max_ = 268 nm.

##### Cyclopentyl[6-methoxy-2-(4-methoxyphenyl)benzo[*b*]thiophen-3-yl]methanone (**14a**)

Following general procedure 1 under a nitrogen atmosphere using 6-methoxy-2-(4-methoxyphenyl)benzo[*b*]thiophene (**8**, 542 mg, 2.00 mmol, 1.0 equiv), cyclopentanecarbonyl chloride (250 μL, 2.06 mmol, 1 equiv), and aluminum chloride (323 mg, 2.42 mmol, 1.2 equiv) in CH_2_Cl_2_ (20 mL). Cyclopentanecarbonyl chloride (50 μL, 0.41 mmol, 0.2 equiv) and aluminum chloride (56 mg, 0.42 mmol, 0.21 equiv) were added after 21 h. The reaction was stopped by the addition of 2 M HCl_aq_ (20 mL) after 26 h. Extracted with CH_2_Cl_2_ (3 × 20 mL) and washed with H_2_O, brine/2 M HCl_aq_, and brine (100 mL each). Purification by silica gel chromatography (*n*-hexane/EtOAc 20:1) yielded a pale-yellow solid (249 mg, 0.678 mmol, 34%): C_22_H_22_O_3_S (366.48); mp 111–112 °C; IR (KBr) *ṽ* 2955, 2928, 2864, 2834 (aliph. CH), 1658 (C=O), 1248 (Ar–O-Me) cm^–1^; ^1^H NMR (500 MHz, C_2_D_6_OS) δ_H_ 7.66 (d, *J* = 8.9 Hz, 1H), 7.60 (d, *J* = 2.4 Hz, 1H), 7.43–7.36 (m, 2H), 7.12–7.08 (m, 2H), 7.07 (dd, *J* = 8.9, 2.4 Hz, 1H), 3.84 (s, 3H), 3.83 (s, 3H), 2.93 (tt, *J* = 8.2, 6.8 Hz, 1H), 1.72–1.62 (m, 2H), 1.61–1.45 (m, 4H), 1.45–1.33 (m, 2H) ppm; ^13^C NMR (126 MHz, C_2_D_6_OS) δ_C_ 204.40, 160.01, 157.32, 143.08, 139.23, 132.23, 132.12, 130.22 (2C), 125.12, 123.58, 115.15, 114.52 (2C), 104.81, 55.44, 55.26, 50.52, 29.25 (2C), 25.62 (2C) ppm; elemental analysis [%] calcd: C 72.10, H 6.05, found: C 71.91, H 6.24; MS (APCI-DI^+^) *m*/*z* [M-97+H]^+^ 270 (53%), [M + H]^+^ 367 (100%); HPLC (Method 1) retention time = 4.8 min, 99.3% (254 nm), 98.2% (280 nm), *t*_M_ (DMSO) = 1.2 min (MeCN/H_2_O 80:20); HPLC (Method 2) retention time = 14.8 min, 99.3%, *t*_M_ (DMSO) = 1.1 min, λ_max_ = 266, 317 nm.

##### Cyclohexyl[6-methoxy-2-(4-methoxyphenyl)benzo[*b*]thiophen-3-yl]methanone (**14b**)

Following general procedure 1 under a nitrogen atmosphere using 6-methoxy-2-(4-methoxyphenyl)benzo[*b*]thiophene (**8**, 1352 mg, 5.00 mmol, 1.0 equiv), cyclohexanecarbonyl chloride (809 μL, 6.00 mmol, 1.2 equiv), and aluminum chloride (1003 mg, 7.52 mmol, 1.5 equiv) in CH_2_Cl_2_ (60 mL). The reaction was stopped by the addition of 2 M HCl_aq_ (40 mL) after 27.5 h. Extracted with CH_2_Cl_2_ (3 × 40 mL) and washed with H_2_O, brine/2 M HCl_aq_, and brine (150 mL each). Purification by silica gel chromatography (*n*-hexane/EtOAc 20:1) yielded a pale-yellow solid (521 mg, 1.37 mmol, 27%): C_23_H_24_O_3_S (380.50); mp 135–136 °C; IR (KBr) *ṽ* 2932 (aliph. CH), 1654 (C=O), 1248 (Ar–O-Me) cm^–1^; ^1^H NMR (400 MHz, C_2_D_6_OS) δ_H_ 7.67 (d, *J* = 8.9 Hz, 1H), 7.60 (d, *J* = 2.4 Hz, 1H), 7.43–7.37 (m, 2H), 7.12–7.08 (m, 2H), 7.06 (dd, *J* = 8.9, 2.4 Hz, 1H), 3.84 (s, 3H), 3.83 (s, 3H), 2.37 (tt, *J* = 10.8, 3.2 Hz, 1H), 1.62–1.53 (m, 4H), 1.46 (d, *J* = 13.4 Hz, 1H), 1.34–1.20 (m, 2H), 1.14–1.00 (m, 1H), 0.95–0.77 (m, 2H) ppm; ^13^C NMR (101 MHz, C_2_D_6_OS) δ_C_ 204.63, 160.11, 157.38, 142.91, 139.36, 132.42, 131.50, 130.14 (2C), 125.32, 123.73, 115.19, 114.55 (2C), 104.88, 55.51, 55.38, 49.71, 28.28 (2C), 25.17, 24.99 (2C) ppm; elemental analysis [%] calcd: C 72.60 H 6.36, found: C 72.50 H 6.46; MS (APCI-DI^+^) *m*/*z* [M + H]^+^ 381 (100%); MS (APCI-DI^–^) *m*/*z* [M – H]^−^ 379 (100%); HPLC (Method 1) retention time = 5.7 min, 96.9% (254 nm), 91.2% (280 nm), *t*_M_ (DMSO) = 1.2 min (MeCN/H_2_O 80:20); HPLC (Method 2) retention time = 15.3 min, 98.3%, *t*_M_ (DMSO) = 1.2 min, λ_max_ = 265, 316 nm.

##### Cyclopentyl[6-hydroxy-2-(4-hydroxyphenyl)benzo[*b*]thiophen-3-yl]methanone (**15a**)

Following general procedure 3 using cyclopentyl[6-methoxy-2-(4-methoxyphenyl)benzo[*b*]thiophen-3-yl]methanone (**14a**, 184 mg, 0.501 mmol, 1 equiv) and pyridinium chloride (1158 mg, 10.02 mmol, 20 equiv) for 2 h. Extracted after the addition of 1.3 M HCl_aq_ (60 mL) with EtOAc (3 × 50 mL) and washed with NaHCO_3_ 10% (3 × 50 mL), brine, and water (100 mL each). Purification by silica gel chromatography (CH_2_Cl_2_/EtOAc 30:1) and following recrystallization from CH_2_Cl_2_ yielded a yellow solid (125 mg, 0.369 mmol, 74%): C_20_H_18_O_3_S (338.42); mp 130 °C (dec.); IR (KBr) *ṽ* 3379 (OH), 2951 (aliph. CH), 1649 (C=O) cm^–1^; ^1^H NMR (600 MHz, C_2_D_6_OS) δ_H_ 9.94 (s, 1H), 9.76 (s, 1H), 7.57 (dd, *J* = 8.8, 0.4 Hz, 1H), 7.28 (dd, *J* = 2.4, 0.4 Hz, 1H), 7.27–7.24 (m, 2H), 6.92 (dd, *J* = 8.8, 2.3 Hz, 1H), 6.90–6.88 (m, 2H), 2.93 (tt, *J* = 8.3, 6.8 Hz, 1H), 1.71–1.60 (m, 2H), 1.59–1.44 (m, 4H), 1.43–1.34 (m, 2H) ppm; ^13^C NMR (151 MHz, C_2_D_6_OS) δ_C_ 204.55, 158.38, 155.35, 142.68, 139.07, 131.86, 131.27, 130.21 (2C), 123.69, 123.59, 115.82 (2C), 115.30, 106.75, 50.43, 29.30 (2C), 25.64 (2C) ppm; MS (ESI-DI^–^) *m*/*z* [M – H]^−^ 337 (100%); HRMS (ESI^+^) *m*/*z* [M + H]^+^ calcd: 339.10495 found: 339.10505, [M + Na]^+^ calcd: 361.08689 found: 339.10505, [2M + Na]^+^ calcd: 699.18456 found: 699.18477; HPLC (Method 1) retention time = 4.6 min, 95.8% (254 nm), 96.4% (280 nm), *t*_M_ (DMSO) = 1.1 min (MeCN/H_2_O 50:50); HPLC (Method 2) retention time = 10.9 min, 98.4%, *t*_M_ (DMSO) = 1.3 min, λ_max_ = 268, 318 nm.

##### Cyclohexyl[6-hydroxy-2-(4-hydroxyphenyl)benzo[*b*]thiophen-3-yl]methanone (**15b**)

Following general procedure 3 using cyclohexyl[6-methoxy-2-(4-methoxyphenyl)benzo[*b*]thiophen-3-yl]methanone (**14b**, 190 mg, 0.499 mmol, 1 equiv) and pyridinium chloride (1156 mg, 10.00 mmol, 20 equiv) for 2 h. Extracted after the addition of 2 M HCl_aq_/H_2_O (2:1, 60 mL) with EtOAc (3 × 50 mL) and washed with NaHCO_3_ 10% (3 × 50 mL), brine, and water (100 mL each). Purification by silica gel chromatography (CH_2_Cl_2_/EtOAc 30:1) and following recrystallization from CH_2_Cl_2_ yielded a yellow solid (79 mg, 0.23 mmol, 45%): C_21_H_20_O_3_S (352.45); mp 130 °C (dec.); IR (KBr) *ṽ* 3411 (OH), 2925, 2851 (aliph. CH), 1650 (C=O) cm^–1^; ^1^H NMR (600 MHz, C_2_D_6_OS) δ_H_ 9.93 (s, 1H), 9.76 (s, 1H), 7.57 (d, *J* = 8.9 Hz, 1H), 7.28 (dd, *J* = 2.3, 0.5 Hz, 1H), 7.27–7.24 (m, 2H), 6.91 (dd, *J* = 8.8, 2.3 Hz, 1H), 6.92–6.87 (m, 2H), 2.36 (tt, *J* = 11.2, 3.1 Hz, 1H), 1.61–1.53 (m, 4H), 1.50–1.43 (m, 1H), 1.30–1.21 (m, 2H), 1.12–1.01 (m, 1H), 0.91–0.80 (m, 2H) ppm; ^13^C NMR (151 MHz, C_2_D_6_OS) δ_C_ 204.69, 158.37, 155.32, 142.44, 139.11, 131.37, 131.17, 130.04 (2C), 123.79, 123.65, 115.77 (2C), 115.26, 106.75, 49.58, 28.26 (2C), 25.12, 24.95 (2C) ppm; MS (ESI-DI^–^) *m*/*z* [M – H]^−^ 351 (100%); HRMS (ESI^+^) *m*/*z* [M + H]^+^ calcd: 353.12060 found: 353.12077, [M + Na]^+^ calcd: 375.10254 found: 375.10270, [2M + Na]^+^ calcd: 727.21586 found: 727.21616; HPLC (Method 1) retention time = 5.8 min, 98.3% (254 nm), 95.9% (280 nm), *t*_M_ (DMSO) = 1.1 min (MeCN/H_2_O 50:50); HPLC (Method 2) retention time = 11.2 min, 97.3%, *t*_M_ (DMSO) = 1.2 min, λ_max_ = 267, 317 nm.

##### 1-[6-Hydroxy-2-(4-hydroxyphenyl)benzo[*b*]thiophen-3-yl]hexan-1-on (**15c**)

Step 1: Following general procedure 1 under a nitrogen atmosphere using 6-methoxy-2-(4-methoxyphenyl)benzo[*b*]thiophene (**8**, 541 mg, 2.00 mmol, 1 equiv), hexanoyl chloride (300 μL, 2.02 mmol, 1 equiv) and aluminum chloride (322 mg, 2.41 mmol, 1.2 equiv) in CH_2_Cl_2_ (20 mL). In deviation from the general procedure, the reaction was stopped by the addition of H_2_O (20 mL) after 9 h, the aqueous layer was extracted with diethyl ether (3 × 50 mL), washed with brine and H_2_O (100 mL each) and concentrated *in vacuo*.

Step 2: Following general procedure 2 using the crude obtained from step 1 (573 mg, 1.56 mmol) and boron tribromide (1 M in CH_2_Cl_2_, 4.5 mL, 4.5 mmol, 3 equiv) in CH_2_Cl_2_ (20 mL). The reaction was stopped by the addition of H_2_O (20 mL) after 3.5 h. Extracted after the addition of methanol (20 mL) and CH_2_Cl_2_ (20 mL) with 10% methanol in CH_2_Cl_2_ (3 × 20 mL) and washed with brine, H_2_O and brine (100 mL each). Purification by silica gel chromatography (CH_2_Cl_2_/EtOAc 25:1) yielded a yellow solid (111 mg, 0.326 mmol, 16%): C_20_H_20_O_3_S (340.44); mp 141–143 °C; IR (KBr) *ṽ* 3354 (OH), 2953, 2924, 2855 (aliph. CH), 1636 (C=O) cm^–1^; ^1^H NMR (500 MHz, C_2_D_6_OS) δ_H_ 9.92 (s, 1H), 9.74 (s, 1H), 7.71 (d, *J* = 8.8 Hz, 1H), 7.29–7.24 (m, 3H), 6.92 (dd, *J* = 8.8, 2.3 Hz, 1H), 6.90–6.87 (m, 2H), 2.37 (t, *J* = 7.3 Hz, 2H), 1.48–1.40 (m, 2H), 1.15–1.00 (m, 4H), 0.75 (t, *J* = 7.2 Hz, 3H) ppm; ^13^C NMR (126 MHz, C_2_D_6_OS) δ_C_ 201.21, 158.46, 155.30, 144.01, 139.11, 131.66, 130.82, 130.32 (2C), 124.00, 123.75, 115.74 (2C), 115.25, 106.68, 42.23, 30.46, 23.59, 21.56, 13.58 ppm; MS (ESI-DI^–^) *m*/*z* [M – H]^−^ 339 (100%); HRMS (ESI^+^) *m*/*z* [M + H]^+^ calcd: 341.12060 found: 341.12076, [M + Na]^+^ calcd: 363.10254 found: 363.10272, [2M + Na]^+^ calcd: 703.21586 found: 703.21615; HPLC (Method 1) retention time = 6.2 min, 98.0% (254 nm), 97.8% (280 nm), *t*_M_ (DMSO) = 1.1 min (MeCN/H_2_O 50:50); HPLC (Method 2) retention time = 11.3 min, 96.9%, *t*_M_ (DMSO) = 1.1 min, λ_max_ = 265, 316 nm.

##### 2-(4-Methoxyphenyl)benzo[*b*]thiophene (**18a**)

Following general procedure 6 using benzo[*b*]thiophene-2-boronic acid (586 mg, 3.29 mmol, 1.1 equiv), tetrakis(triphenylphosphine)palladium(0) (177 mg, 0.153 mmol, 0.05 equiv), potassium carbonate (1273 mg, 5.997 mmol, 2.0 equiv), and 4-bromoanisole (376 μL, 3.00 mmol, 1 equiv). The precipitated product was filtrated yielding a white-silver solid (302 mg, 1.26 mmol, 42%): C_15_H_12_OS (240.32); mp 188–191 °C [lit. 188–191 °C];^66^ IR (KBr) *ṽ* 1604, 1499 (C=C) cm^–1^; ^1^H NMR (500 MHz, CDCl_3_) δ_H_ 7.82–7.78 (m, 1H), 7.75–7.72 (m, 1H), 7.68–7.61 (m, 2H), 7.42 (d, *J* = 0.6 Hz, 1H), 7.35–7.31 (m, 1H), 7.30–7.26 (m, 1H), 6.99–6.92 (m, 2H), 3.85 (s, 3H) ppm; ^13^C NMR (126 MHz, CDCl_3_) δ_C_ 159.82, 144.16, 140.90, 139.20, 127.77 (2C), 127.08, 124.45, 123.95, 123.26, 122.18, 118.22, 114.37 (2C), 55.41 ppm; MS (APCI-ASAP^+^) *m*/*z* [M + H]^+^ 241 (100%); HPLC (Method 1) retention time = 3.7 min, 95.4% (254 nm), 98.3% (280 nm), *t*_M_ (DMSO) = 1.2 min (MeCN/H_2_O 80:20); HPLC (Method 2) retention time = 14.2 min, 93.6%, *t*_M_ (DMSO) = 1.1 min, λ_max_ = 308 nm.

The compound is described in the literature.^66^

##### 6-Methoxy-2-phenylbenzo[*b*]thiophene (**18b**)

Following general procedure 6 using 6-methoxybenzo[*b*]thiophene-2-boronic acid (343 mg, 1.65 mmol, 1.0 equiv), tetrakis(triphenylphosphine)palladium(0) (95 mg, 0.050 mmol, 0.05 equiv), potassium carbonate (418 mg, 3.02 mmol, 1.8 equiv), and iodobenzene (167 μL, 1.50 mmol, 1.1 equiv). Extracted with CHCl_3_ (4 × 15 mL) and washed with 1 M NaOH (2 × 50 mL) and brine (2 × 50 mL). Purification by silica gel chromatography (*n*-hexane/EtOAc 40:1) yielded a silver solid (238 mg, 0.992 mmol, 60%): C_15_H_12_OS (240.32); mp 154–156 °C [lit. 157–159 °C];^74^ IR (KBr) *ṽ* 1594 (C=C) cm^–1^; ^1^H NMR (500 MHz, CDCl_3_) δ_H_ 7.69–7.66 (m, 2H), 7.64 (d, *J* = 8.7 Hz, 1H), 7.45 (d, *J* = 0.7 Hz, 1H), 7.43–7.38 (m, 2H), 7.33–7.28 (m, 2H), 6.98 (dd, *J* = 8.7, 2.3 Hz, 1H), 3.88 (s, 3H) ppm; ^13^C NMR (126 MHz, CDCl_3_) δ_C_ 157.48, 141.62, 140.99, 134.74, 134.50, 128.90 (2C), 127.82, 126.17 (2C), 124.22, 119.01, 114.54, 104.88, 55.63 ppm; MS (APCI-DI^+^) *m*/*z* [M + H]^+^ 241 (100%); HPLC (Method 1) retention time = 3.6 min, 98.6% (254 nm), 99.1% (280 nm), *t*_M_ (DMSO) = 1.2 min (MeCN/H_2_O = 80:20); HPLC (Method 2) retention time = 14.1 min, 98.5%, *t*_M_ (DMSO) = 1.1 min, λ_max_ = 261, 310 nm.

The compound is described in the literature.^74^

##### 2-Phenylbenzo[*b*]thiophene (**18c**)

Following general procedure 6 using benzo[*b*]thiophene-2-boronic acid (588 mg, 3.31 mmol, 1.0 equiv), tetrakis(triphenylphosphine)palladium(0) (173 mg, 0.150 mmol, 0.045 equiv), potassium carbonate (1275 mg, 6.01 mmol, 1.8 equiv), and iodobenzene (410 μL, 3.68 mmol, 1.1 equiv). Extracted after the addition of H_2_O (30 mL) with CHCl_3_ (3 × 25 mL) and washed with 1 M NaOH and brine (100 mL each). Purification by silica gel chromatography (*n*-hexane) yielded a white solid (630 mg, 2.99 mmol, 90%): C_14_H_10_S (210.29); mp 173–175 °C; IR (KBr) *ṽ* 1445 (C=C) cm^–1^; ^1^H NMR (400 MHz, CDCl_3_) δ_H_ 7.86–7.80 (m, 1H), 7.79–7.75 (m, 1H), 7.74–7.69 (m, 2H), 7.54 (d, *J* = 0.6 Hz, 1H), 7.46–7.39 (m, 2H), 7.38–7.28 (m, 3H) ppm; ^13^C NMR (101 MHz, CDCl_3_) δ_C_ 144.25, 140.70, 139.51, 134.32, 128.95 (2C), 128.26, 126.50 (2C), 124.51, 124.31, 123.56, 122.27, 119.45 ppm; MS (APCI-ASAP^+^) *m*/*z* [M + H]^+^ 211 (100%); HPLC (Method 1) retention time = 3.9 min, 98.0% (254 nm), 99.5% (280 nm), *t*_M_ (DMSO) = 1.2 min (MeCN/H_2_O 80:20); HPLC (Method 2) retention time = 14.3 min, 97.8%, *t*_M_ (DMSO) = 1.1 min, λ_max_ = 288, 377 nm.

The compound is described in the literature.^75^

##### (4-Chlorophenyl)[2-(4-methoxyphenyl)benzo[*b*]thiophen-3-yl]methanone (**19a**)

Following general procedure 1 using 2-(4-methoxyphenyl)benzo[*b*]thiophene (**18a**, 310 mg, 1.29 mmol, 1 equiv), 4-chlorobenzoyl chloride (200 μL, 1.57 mmol, 1.2 equiv) and aluminum chloride (258 mg, 1.94 mmol, 1.5 equiv) in CH_2_Cl_2_ (30 mL). The reaction was stopped by the addition of 2 M HCl_aq_ (20 mL) after 23 h. Extracted with CH_2_Cl_2_ (3 × 50 mL) and washed with a mixture of brine and 2 M HCl_aq_ (1:1), 10% NaHCO_3_ and brine (100 mL each). Without further purification, pale-yellow crystals (469 mg, 1.24 mmol, 96%) were obtained: C_22_H_15_ClO_2_S (378.9); mp 137–138 °C; IR (KBr) *ṽ* 1656 (C=O), 1247 (Ar–O-Me) cm^–1^; ^1^H NMR (500 MHz, C_2_D_6_OS) δ_H_ 8.11–8.09 (m, 1H), 7.71–7.68 (m, 2H), 7.62–7.58 (m, 1H), 7.47–7.40 (m, 4H), 7.34–7.30 (m, 2H), 6.91–6.87 (m, 2H), 3.72 (s, 3H) ppm; ^13^C NMR (126 MHz, C_2_D_6_OS) δ_C_ 192.31, 159.87, 146.02, 138.87, 138.41, 137.80, 135.49, 131.12 (2C), 130.19 (2C), 129.45, 128.77 (2C), 125.38, 125.13, 124.62, 122.64, 122.44, 114.34 (2C), 55.18 ppm; elemental analysis (%) calcd: C 69.74 H 3.99, found: C 69.73, H 3.60; MS (APCI-DI^+^) *m*/*z* [M + H]^+^ 379 (100%); MS (APCI-DI^–^) *m*/*z* [M-H_2_O–H]^−^ 359 (100%); HPLC (Method 1) retention time = 4.5 min, 97.9% (254 nm), 98.5% (280 nm), *t*_M_ (DMSO) = 1.1 min (MeCN/H_2_O 80:20); HPLC (Method 2) retention time = 14.8 min, 98.0%, *t*_M_ (DMSO) = 1.1 min, λ_max_ = 268 nm.

##### (4-Chlorophenyl)(6-methoxy-2-phenylbenzo[*b*]thiophen-3-yl)methanone (**19b**)

Following general procedure 1 using 6-methoxy-2-phenylbenzo[*b*]thiophene (**18b**, 180 mg, 0.75 mmol, 1 equiv), 4-chlorobenzoyl chloride (115 μL, 0.90 mmol, 1.2 equiv) and aluminum chloride (151 mg, 1.13 mmol, 1.5 equiv) in CH_2_Cl_2_ (20 mL). The reaction was stopped by the addition of 2 M HCl_aq_ (20 mL) after 5 h. Extracted with CH_2_Cl_2_ (3 × 50 mL) and washed with brine/2 M HCl_aq_, 10% NaHCO_3_ and brine (100 mL each). Purification by silica gel chromatography (*n*-hexane/EtOAc 15:1) yielded a dark yellow gum (109 mg, 0.288 mmol, 38%): C_22_H_15_ClO_2_S (378.87); IR (KBr) *ṽ* 1638 (C=O), 1254 (Ar–O-Me) cm^–1^; ^1^H NMR (400 MHz, C_2_D_6_OS) δ_H_ 7.72 (d, *J* = 2.5 Hz, 1H), 7.70–7.66 (m, 2H), 7.53 (d, *J* = 8.9 Hz, 1H), 7.44–7.39 (m, 2H), 7.37–7.27 (m, 5H), 7.07 (dd, *J* = 8.9, 2.5 Hz, 1H), 3.86 (s, 3H) ppm; ^13^C NMR (101 MHz, C_2_D_6_OS) δ_C_ 192.22, 157.66, 143.44, 139.83, 138.43, 135.59, 132.74, 132.59, 131.16 (2C), 130.09, 128.87, 128.85 (2C), 128.77 (2C), 128.75 (2C), 123.72, 115.43, 105.16, 55.59 ppm; MS (APCI-DI^+^) *m*/*z* [M + H]^+^ 379 (100%); HPLC (Method 1) retention time = 4.5 min, 98.6% (254 nm), 98.5% (280 nm), *t*_M_ (DMSO) = 1.2 min (MeCN/H_2_O 80:20); HPLC (Method 2) retention time = 14.9 min, 98.8%, *t*_M_ (DMSO) = 1.1 min, λ_max_ = 256 nm.

##### (3-Bromophenyl)[2-(4-methoxyphenyl)benzo[*b*]thiophen-3-yl]methanone (**19c**)

Following general procedure 1 using 2-(4-methoxyphenyl)benzo[*b*]thiophene (**18a**, 147 mg, 0.612 mmol, 1.0 equiv), 3-bromobenzoyl chloride (89 μL, 0.67 mmol, 1.1 equiv) and aluminum chloride (98 mg, 0.73 mmol, 1.2 equiv) in CH_2_Cl_2_ (20 mL). Further addition of 3-bromobenzoyl chloride (16 μL, 0.12 mmol, 0.2 equiv), and aluminum chloride (16 mg, 0.12 mmol, 0.2 equiv) after 21 h. The reaction was stopped by the addition of 2 M HCl_aq_ (20 mL) after 27 h. Extracted with CH_2_Cl_2_ (3 × 20 mL) and washed with brine/2 M HCl_aq_ (1:1) (4 × 50 mL). Purification by silica gel chromatography (*n*-hexane/EtOAc 30:1) yielded a pale-yellow solid (154 mg, 0.364 mmol, 60%): C_22_H_15_BrO_2_S (423.32); mp 124–126 °C (ethanol); IR (KBr) *ṽ* 1640 (C=O); ^1^H NMR (500 MHz, C_2_D_6_OS) δ_H_ 7.76 (dd, *J* = 1.8, 1.8 Hz, 1H), 7.73 (d, *J* = 2.4 Hz, 1H), 7.68 (ddd, *J* = 7.9, 1.8, 0.9 Hz, 1H), 7.63–7.60 (m, 1H), 7.61 (d, *J* = 8.9 Hz, 1H), 7.37–7.32 (m, 2H), 7.32–7.24 (m, 4H), 7.08 (dd, *J* = 8.9, 2.4 Hz, 1H), 3.87 (s, 3H) ppm; ^13^C NMR (126 MHz, C_2_D_6_OS) δ_C_ 191.64, 157.70, 144.64, 139.85, 138.90, 135.92, 132.70, 132.63, 131.67, 130.77, 129.71, 128.97, 128.90 (2C), 128.80 (2C), 128.42, 123.89, 121.78, 115.51, 105.14, 55.61 ppm; MS (APCI-DI^+^) *m*/*z* [M_79Br_+H]^+^ 423 (96%); HPLC (Method 2) retention time = 15.2 min, 99.3%, *t*_M_ (DMSO) = 1.1 min, λ_max_ = 253, 289 nm.

##### (3-Bromophenyl)(6-methoxy-2-phenylbenzo[*b*]thiophen-3-yl)methanone (**19d**)

Following general procedure 1 using 6-methoxy-2-phenylbenzo[*b*]thiophene (**18b**, 312 mg, 1.30 mmol, 1.0 equiv), 3-bromobenzoyl chloride (188 μL, 1.43 mmol, 1.1 equiv,) and aluminum chloride (225 mg, 1.69 mmol, 1.3 equiv) in CH_2_Cl_2_ (25 mL). The reaction was stopped by the addition of 2 M HCl_aq_ (25 mL) after 17 h. Extracted with CH_2_Cl_2_ (3 × 15 mL) and washed with brine and 2 M HCl_aq_ (1:1) (4 × 50 mL). Purification by recrystallization from ethanol (15 mL) yielded yellow crystals (267 mg, 0.631 mmol, 49%): C_22_H_15_BrO_2_S (423.32); mp 141–142 °C (ethanol); IR (KBr) *ṽ* 1637 (C=O), 1251 (Ar–O-Me) cm^–1^; ^1^H NMR (500 MHz, C_2_D_6_OS) δ_H_ 8.15–8.07 (m, 1H), 7.78 (dd, *J* = 1.8, 1.8 Hz, 1H), 7.73–7.67 (m, 2H), 7.62 (ddd, *J* = 7.9, 1.8, 1.1 Hz, 1H), 7.50–7.41 (m, 2H), 7.35–7.27 (m, 3H), 6.91–6.85 (m, 2H), 3.71 (s, 3H) ppm; ^13^C NMR (126 MHz, C_2_D_6_OS) δ_C_ 191.72, 159.88, 147.22, 138.82, 138.79, 137.82, 135.88, 131.53, 130.75, 130.32 (2C), 129.10, 128.39, 125.43, 125.19, 124.65, 122.78, 122.43, 121.76, 114.27 (2C), 55.18 ppm; elemental analysis [%] calcd: C 62.42 H 3.57, found: C 62.16 H 3.41; MS (APCI-DI^+^) *m*/*z* [M_79Br_+H]^+^ 423 (97%); HPLC (Method 1) retention time = 5.0 min, 97.0% (254 nm), 97.4% (280 nm), *t*_M_ (DMSO) = 1.1 min (MeCN/H_2_O 80:20); HPLC (Method 2) retention time = 15.1 min, 98.6%, *t*_M_ (DMSO) = 1.1 min, λ_max_ = 253, 289 nm.

##### (4-Chlorophenyl)(2-phenylbenzo[*b*]thiophen-3-yl)methanone (**19e**)

Following general procedure 1 using 2-phenylbenzo[*b*]thiophene (**18c**, 212 mg, 1.01 mmol, 1 equiv), 4-chlorobenzoyl chloride (154 μL, 1.21 mmol, 1.2 equiv), and aluminum chloride (202 mg, 1.51 mmol, 1.5 equiv) in CH_2_Cl_2_ (20 mL). The reaction was stopped by the addition of 2 M HCl_aq_ (20 mL) after 2 h. Extracted with CH_2_Cl_2_ (3 × 50 mL) and washed with brine/2 M HCl_aq_, 10% NaHCO_3_ and brine (100 mL each). Purification by silica gel chromatography (*n*-hexane/EtOAc 30:1) followed by preparative HPLC (MeCN/H_2_O 80:20) yielded a pale-red solid (69 mg, 0.20 mmol, 20%): C_21_H_13_ClOS (348.8); mp 66–68 °C; IR (KBr) *ṽ* 1660 (C=C) cm^–1^; ^1^H NMR (500 MHz, CDCl_3_) δ_H_ 7.92–7.86 (m, 1H), 7.77–7.73 (m, 1H), 7.71–7.67 (m, 2H), 7.43–7.36 (m, 4H), 7.25–7.20 (m, 5H) ppm; ^13^C NMR (126 MHz, CDCl_3_) δ_C_ 192.88, 146.87, 139.61, 139.49, 138.97, 135.86, 133.10, 131.21 (2C), 131.02, 129.33 (2C), 129.03, 128.69 (2C), 128.64 (2C), 125.30, 125.24, 123.56, 122.03 ppm; MS (APCI-DI^+^) *m*/*z* [M + H]^+^ 349 (100%); HRMS (ESI^+^) *m*/*z* [M + H]^+^ calcd: 349.04484 found: 349.04495, [M + Na]^+^ calcd: 371.02678 found: 371.02688, [2M + Na]^+^ calcd: 719.06434 found: 719.06462; HPLC (Method 1) retention time = 4.8 min, 99.9% (254 nm), 99.9% (280 nm), *t*_M_ (DMSO) = 1.2 min (ACN/H_2_O = 80:20); HPLC (Method 2) retention time = 15.1 min, 99.5%, *t*_M_ (DMSO) = 1.1 min, λ_max_ = 246, 268 nm.

The compound is described in the literature.^76,77^

##### (3-Bromophenyl)(2-phenylbenzo[*b*]thiophen-3-yl)methanone (**19f**)

Following general procedure 1 using (2-phenyl)benzo[*b*]thiophene (**18c**, 387 mg, 1.84 mmol, 1.0 equiv), 3-bromobenzoyl chloride (266 μL, 2.01 mmol, 1.1 equiv) and aluminum chloride (318 mg, 2.39 mmol, 1.3 equiv) in CH_2_Cl_2_ (40 mL). The reaction was stopped by the addition of 2 M HCl_aq_ (20 mL) after 18 h. Extracted with CH_2_Cl_2_ (3 × 40 mL) and washed with brine/2 M HCl_aq_ (1:1) and brine (50 mL each). Purification of a fraction (125 mg) of the crude product (680 mg) by preparative HPLC (MeCN/H_2_O 80:20) yielded a pale-yellow solid (33 mg, 0.084 mmol, 5%): C_21_H_13_BrOS (393.30); mp 66–67 °C; IR (KBr) *ṽ* 1636 (C=O) cm^–1^; ^1^H NMR (500 MHz, CDCl_3_) δ_H_ 7.91 (ddd, *J* = 5.9, 3.3, 0.7 Hz, 1H), 7.87 (dd, *J* = 1.8, 1.8 Hz, 1H), 7.85 (ddd, *J* = 6.1, 3.3, 0.9 Hz, 1H), 7.64 (ddd, *J* = 7.9, 1.8, 1.2 Hz, 1H), 7.50 (ddd, *J* = 7.9, 1.8, 1.2 Hz, 1H), 7.45–7.41 (m, 2H), 7.42–7.36 (m, 2H), 7.28–7.20 (m, 3H), 7.11 (dd, *J* = 7.9, 7.9 Hz, 1H) ppm; ^13^C NMR (126 MHz, CDCl_3_) δ_C_ 192.56, 148.10, 139.56, 139.42, 139.12, 135.92, 133.29, 132.89, 130.88, 129.94, 129.58 (2C), 129.21, 128.79 (2C), 128.55, 125.56, 125.47, 123.81, 122.62, 122.16 ppm; MS (APCI-DI^+^) *m*/*z* [M_79Br_+H]^+^ 393 (100%); HRMS (ESI^+^) *m*/*z* [M + H]^+^ calcd: 394.99228 found: 394.99223, [M + Na]^+^ calcd: 416.97422 found: 416.97420, [2M + Na]^+^ calcd: 808.963312, found: 808.96160; HPLC (Method 1) retention time = 5.2 min, 99.9% (254 nm), >99.9% (280 nm), *t*_M_ (DMSO) = 1.1 min (MeCN/H_2_O 80:20); HPLC (Method 2) retention time = 15.3 min, >99.9%, *t*_M_ (DMSO) = 1.1 min, λ_max_ = 241 nm.

##### (4-Chlorophenyl)[2-(4-hydroxyphenyl)benzo[*b*]thiophen-3-yl]methanone (**20a**)

Following general procedure 2 using (4-chlorophenyl)[2-(4-methoxyphenyl)benzo[*b*]thiophene-3-yl]methanone (**19a**, 378 mg, 1.00 mmol, 1.0 equiv) and boron tribromide (1 M in CH_2_Cl_2_, 2 mL, 2 mmol, 2.0 equiv) in CH_2_Cl_2_ (10 mL). The reaction was stopped by the addition of H_2_O (20 mL) after 2 h. Extracted after the addition of methanol (20 mL) and CH_2_Cl_2_ (10 mL) with 10% methanol in CH_2_Cl_2_ (3 × 20 mL) and washed with brine (100 mL each). Purification by silica gel chromatography (*n*-hexane/EtOAc 5:1) yielded a yellow solid (131 mg, 0.360 mmol, 36%): C_21_H_13_ClO_2_S (364.8); mp 154–155 °C; IR (KBr) *ṽ* 3418 (OH), 1625 (C=O) cm^–1^; ^1^H NMR (500 MHz, C_2_D_6_OS) δ_H_ 9.83 (s, 1H), 8.10–8.07 (m, 1H), 7.69–7.65 (m, 2H), 7.64–7.61 (m, 1H), 7.46–7.40 (m, 4H), 7.21–7.18 (m, 2H), 6.70–6.67 (m, 2H) ppm; ^13^C NMR (126 MHz, C_2_D_6_OS) δ_C_ 192.29, 158.37, 146.95, 138.96, 138.21, 137.68, 135.60, 131.10 (2C), 130.32 (2C), 128.87, 128.67 (2C), 125.32, 125.00, 123.03, 122.57, 122.39, 115.65 (2C) ppm; elemental analysis (%) calcd: C 69.13 H 3.59. Found: C 69.12, H 3.92; MS (ESI-DI^–^) *m*/*z* [M_35Cl_-H]^−^ 363 (100%); HPLC (Method 1) retention time = 4.0 min, 99.2% (254 nm), 99.5% (280 nm), *t*_M_ (DMSO) = 1.1 min (MeCN/H_2_O 70:30); HPLC (Method 2) retention time = 13.0 min, 98.5%, *t*_M_ (DMSO) = 1.1 min, λ_max_ = 268 nm.

##### (4-Chlorophenyl)[6-hydroxy-2-phenylbenzo[*b*]thiophen-3-yl]methanone (**20b**)

Following general procedure 3 using (4-chlorophenyl)[6-methoxy-2-phenylbenzo[*b*]thiophen-3-yl]methanone (**19b**, 76 mg, 0.20 mmol, 1 equiv) and pyridinium chloride (233 mg, 2.02 mmol, 10 equiv) for 1.5 h. Extracted after the addition of 1.3 M HCl_aq_ (30 mL) with EtOAc (3 × 50 mL). Deviating from the general procedure, the organic layer was concentrated in a microwave vessel (10 mL) *in vacuo*. The resulting solid and pyridinium chloride (465 mg, 4.03 mmol, 20 equiv) were heated following general procedure 3 for 2 h. Extracted after the addition of 1.3 M HCl_aq_ (60 mL) with EtOAc (3 × 50 mL) and washed with NaHCO_3_ 10% (3 × 50 mL), brine, and water (100 mL each) yielded a yellow-brownish oil (65 mg, 0.18 mmol, 90%): C_21_H_13_ClO_2_S (364.84); IR (KBr) *ṽ* 3411 (OH), 1643 (C=O) cm^–1^; ^1^H NMR (600 MHz, C_2_D_6_OS) δ_H_ 9.90 (s, 1H), 7.69–7.66 (m, 2H), 7.44 (d, *J* = 8.8 Hz, 1H), 7.43–7.40 (m, 3H), 7.34–7.25 (m, 5H), 6.93 (dd, *J* = 8.8, 2.3 Hz, 1H) ppm; ^13^C NMR (151 MHz, C_2_D_6_OS) δ_C_ 192.29, 155.77, 142.12, 139.72, 138.31, 135.54, 132.66, 131.66, 131.07 (2C), 130.12, 128.75 (2C), 128.69 (2C), 128.64, 128.58 (2C), 123.74, 115.57, 107.06 ppm; MS (ESI-DI) *m*/*z* [M_35Cl_-H]^−^ 363 (100%); HRMS (ESI^+^) *m*/*z* [M + H]^+^ calcd: 365.03976 found: 365.03992, [M + Na]^+^ calcd: 387.02170 found: 387.02188, [2M + Na]^+^ calcd: 751.05418 found: 751.05448; HPLC (Method 1) retention time = 3.6 min, 96.3% (254 nm), 96.4% (280 nm), *t*_M_ (DMSO) = 1.1 min (MeCN/H_2_O 70:30); HPLC (Method 2) retention time = 12.9 min, 96.5%, *t*_M_ (DMSO) = 1.2 min, λ_max_ = 256 nm.

##### (3-Bromophenyl)[2-(4-hydroxyphenyl)benzo[*b*]thiophen-3-yl]methanone (**20c**)

Following general procedure 3 using (3-Bromophenyl)(2-(4-methoxyphenyl)benzo[*b*]thiophen-3-yl)methanone (**19c**, 106 mg, 0.250 mmol, 1.0 equiv) and pyridinium hydrochloride (579 mg, 5.01 mmol, 20 equiv) for 2 h. Extracted after the addition of 1.3 M HCl_aq_ (10 mL) with EtOAc (3 × 10 mL) and washed with brine/2 M HCl_aq_ (4 × 15 mL). Purification by silica gel chromatography (*n*-hexane/EtOAc 4:1) yielded a yellow solid (99 mg, 0.24 mmol, 96%): C_21_H_13_BrO_2_S (409.30); mp 169–170 °C; IR (KBr) *ṽ* 3417 (OH), 1639 (C=O) cm^–1^; ^1^H NMR (600 MHz, C_2_D_6_OS) δ_H_ 9.91 (s, 1H), 7.75 (ddd, *J* = 2.1, 1.6, 0.4 Hz, 1H), 7.67 (ddd, *J* = 8.0, 2.1, 1.0 Hz, 1H), 7.61 (ddd, *J* = 7.8, 1.6, 1.0 Hz, 1H), 7.52 (dd, *J* = 8.7, 0.5 Hz, 1H), 7.41 (dd, *J* = 2.3, 0.5 Hz, 1H), 7.34–7.30 (m, 2H), 7.30–7.23 (m, 4H), 6.94 (dd, *J* = 8.8, 2.3 Hz, 1H) ppm; ^13^C NMR (151 MHz, C_2_D_6_OS) δ_C_ 191.78, 155.89, 143.42, 139.83, 138.93, 135.89, 132.77, 131.69, 131.64, 130.78, 129.82, 128.83, 128.82 (2C), 128.77 (2C), 128.42, 123.98, 121.78, 115.72, 107.14 ppm; MS (APCI-DI^–^) *m*/*z* [M_79Br_-H]^−^ 407 (100%), [M_81Br_-H]^−^ 409 (100%); HRMS (ESI^+^) *m*/*z* [M + H]^+^ calcd: 410.98720 found: 410.98714, [M + Na]^+^ calcd: 432.96914 found: 432.96902, [2M + Na]^+^ calcd: 840.951103, found: 840.95135; HPLC (Method 1) retention time = 3.8 min, 98.6% (254 nm), 98.7% (280 nm), *t*_M_ (DMSO) = 1.1 min (MeCN/H_2_O 70:30); HPLC (Method 2) retention time = 13.0 min, 99.5%, *t*_M_ (DMSO) = 1.1 min, λ_max_ = 283 nm.

##### (3-Bromophenyl)(6-hydroxy-2-phenylbenzo[*b*]thiophen-3-yl)methanone (**20d**)

Following general procedure 3 using (3-bromophenyl)(6-methoxy-2-phenylbenzo[*b*]thiophen-3-yl)methanone (**19d**, 209 mg, 0.494 mmol, 1.0 equiv) and pyridinium hydrochloride (1142 mg, 9.88 mmol, 20 equiv) for 2 h. Extracted after the addition of 1.3 M HCl_aq_ (10 mL) with EtOAc (3 × 20 mL) and washed with brine/2 M HCl_aq_ (3 × 30 mL). Purification by silica gel chromatography (*n*-hexane/EtOAc 4:1) yielded a yellow solid (107 mg, 0.261 mmol, 53%): C_21_H_13_BrO_2_S (409.30); mp 160–161 °C; IR (KBr) *ṽ* 3396 (OH), 1620 (C=O) cm^–1^; ^1^H NMR (600 MHz, CDCl_3_) δ_H_ 7.89–7.86 (m, 1H), 7.86 (dd, *J* = 2.0, 1.6 Hz, 1H), 7.83–7.80 (m, 1H), 7.62 (ddd, *J* = 7.8, 1.6, 1.1 Hz, 1H), 7.51 (ddd, *J* = 7.9, 2.0, 1.1 Hz, 1H), 7.44–7.37 (m, 2H), 7.27–7.23 (m, 2H), 7.12 (dd, *J* = 7.9, 7.8 Hz, 1H), 6.71–6.66 (m, 2H), 4.85 (s, 1H) ppm; ^13^C NMR (151 MHz, CDCl_3_) δ_C_ 192.56, 156.27, 147.98, 139.46, 139.28, 138.68, 135.77, 132.75, 130.97 (2C), 129.98, 129.82, 128.44, 125.86, 125.36, 125.14, 123.48, 122.48, 121.95, 115.63 (2C) ppm; MS (APCI-DI^–^) *m*/*z* [M_79Br_-H]^−^ 407 (100%); HRMS (ESI^+^) *m*/*z* [M + H]^+^ calcd: 408.98924 found: 408.98933, [M + Na]^+^ calcd: 430.97118 found: 430.97123, [2M + Na]^+^ calcd: 840.951103 found: 840.95130; HPLC (Method 1) retention time = 4.3 min, 99.6% (254 nm), 99.7% (280 nm), *t*_M_ (DMSO) = 1.1 min (MeCN/H_2_O 70:30); HPLC (Method 2) retention time = 13.3 min, 99.3%, *t*_M_ (DMSO) = 1.1 min, λ_max_ = 254, 291 nm.

### Molecular Docking Studies

Molecular docking was performed using an initial version of the obtained cocrystal structure of RAL (**6**) in *Bc*PhzB (PDB: 9F8H). Protein preparation was carried out in MOE2022.02^78^ using the tools QuickPrep, Protonate 3D, and Structure Preparation. Ligand structures were energy-minimized using MOE (“QuickPrep” function). Molecular docking was carried out in GOLD 5.2.2 (GUI: Hermes 1.6.2). Protons were added and water as well as ligands were extracted. The binding pocket was defined by selecting a 10 Å radius around the extracted RAL (**6**) molecule. Ten docking poses were generated for each ligand (10 genetic algorithm runs, search efficiency 200%, no early termination allowed). Docking poses were evaluated on the basis of their docking scores (ChemPLP scoring function) and by visual inspection using ChimeraX.^79^

### Protein Expression and Purification

Protein expression and purification were performed as described previously with minor modifications.^39^ Briefly, *Bc*PhzB was overexpressed from pET15b using *E. coli* BL21 CodonPlus(DE3)-RIL in terrific broth media (12 g/L tryptone, 24 g/L yeast extract, 0.4% (w/v) glycerol, 17 mM KH_2_PO_4_, 72 mM K_2_HPO_4_) at 20 °C overnight. Overexpression was induced with isopropyl β-d-1-thiogalactopyranoside (final concentration of 0.5 mM) at an OD_600_ of 0.6–0.8. Purification followed standard procedures, involving a two-step purification protocol using a Ni^2+^ affinity matrix (HisTrap HP 5 mL, GE Healthcare) and size exclusion chromatography on HiLoad Superdex 75 pg (GE Healthcare). Pure protein was concentrated in SEC buffer (50 mM TRIS-HCl pH 8.0, 150 mM NaCl), snap-frozen in liquid nitrogen, and stored at −80 °C until further usage.

### Cocrystallization

Structure determination by protein crystallography followed standard protocols. Crystallization assays were set up with the Crystal Gryphon LPC (Art Robbins Instruments, USA) using the vapor diffusion sitting drop method in 96-well Intelli Plates (Art Robbins Instruments, USA) at 20 °C. *Bc*PhzB (10 mg/mL) was incubated with 5 mM of the respective compound (100 mM stock solution in DMSO, 2–8% v/v final DMSO concentration) at 4 °C overnight prior to crystallization. To remove precipitates, the solution was centrifuged (10 min, 17 000*g*, 4 °C). Initial crystallization conditions were identified using commercially available screens and further optimized by random screening. *Bc*PhzB-inhibitor cocrystals formed after a few days in the indicated conditions (Table S4). The crystals were mounted on a nylon loop, cryoprotected with glycerol (25%), and flash-cooled in liquid nitrogen.

### Data Collection, Refinement, and Validation

Diffraction data of flash-cooled crystals were collected at 100 K on beamline P11 of the PETRA III synchrotron at DESY (Hamburg, Germany) and processed with autoProc^80^ using XDS^81^ for reducing and AIMLESS from the CCP4 software suite^82^ for scaling. One crystal was used per reported structure. Phasing was achieved by molecular replacement in phenix.phaser^83^ and refinement involved alternating rounds of manual adjustments in COOT^84^ and minimization with phenix.refine^85^ of the PHENIX software suite.^86^ Ligand restraints were generated using the grade2 web server (version 1.6.0)^87^ or phenix.elbow. Data collection and refinement statistics are listed in Table S5. Figures have been prepared with ChimeraX.^79^

### Nano Differential Scanning Fluorimetry

Protein inflection temperatures were determined via *n*DSF with a Tycho NT.6 instrument (NanoTemper Technologies GmbH, München, Germany). *Bc*PhzB (0.5 mg/mL) was incubated with 250 μM of the respective compound (5% v/v final DMSO concentration) in SEC buffer followed by centrifugation (5 min, 17 000*g*, rt). The solution was loaded into glass capillaries and the melting temperature was obtained as the inflection point of the fluorescence ratio at 350 and 330 nm with respect to temperature (35–95 °C, 30 °C min^–1^).

### Isothermal Titration Calorimetry

*Bc*PhzB was buffer-exchanged via ultrafiltration (Vivaspin ultrafiltration units, molecular weight cutoff 10 kDa) and diluted to the indicated concentration (200–350 μM) in SEC buffer and DMSO was added matching the DMSO concentration in the ligand solution. The respective ligand was diluted to the indicated concentration using SEC buffer (1–2% v/v final DMSO concentration). All solutions were degassed before measurement. ITC measurements were conducted in a MicroCal VP-ITC (Malvern Panalytical) under the specified conditions. Data were analyzed using MicroCal ITC Origin. For this purpose, the first injection was removed and the baseline and integration limits were adjusted manually. The mean value of three independent measurements (±SEM) is reported. ITC traces are reported in Figures S3–S13.

### Pyocyanin Quantification Assay

Pyocyanin was quantified using a previously published procedure with slight modifications.^17,54^ Briefly, overnight cultures of the respective *P. aeruginosa* strains were prepared (2 mL lysogeny broth (LB) medium: 10 g/L tryptone, 5 g/L yeast extract, 10 g/L NaCl, 37 °C). The next day, the cultures were centrifuged (5 min, 4 700 rpm, rt), washed with LB medium (2 × 2 mL), and adjusted to OD_600_ = 0.02 with LB medium. 1960 μL of the culture were placed in 24-well plates and a stock solution of the respective ligand (40 μL, 2% v/v final DMSO concentration) was added. The plates were incubated under aerobic conditions (37 °C, 16 h, 225 rpm). OD_600_ was determined before pelleting cells by centrifugation (5 min, 17 000*g*, rt) and measuring pyocyanin in the supernatant at 695 nm in a microplate reader (Tecan Spark, Tecan Trading AG, Switzerland) to report the ratio of *A*_695_/*A*_600_. Pyocyanin reduction at 10 μM is reported relative to the PA14 wild type DMSO control as the mean value of three independent measurements (±SEM). IC_50_ values were calculated with data from at least three independent measurements using GraphPad Prism 10.1.2 with a three-parameter nonlinear regression model and are reported ± SEM. If the maximum inhibitory effect could not be determined (**6**, **10h/j**, **11d/e/g**, **13a/d/e/g**), the regression was performed using the pyocyanin production of the phzB-knockout mutant as the bottom constraint and an approximately IC_50_ value ± SEM is reported.

### Alkylquinolone Quantification Assay

The production of the alkylquinolones DHQ (2,4-dihydroxyquinoline), 2-AA (2-aminoacetophenone), HQNO (*N*-oxo-2-heptyl-4-hydroxyquinoline), HHQ (2-heptyl-4-quinolone) and PQS (2-heptyl-3-hydroxy-4-quinolone) in *P. aeruginosa* PA14 was measured as described previously.^18^ In brief, *P. aeruginosa* PA14 was incubated with the respective compounds (1% (v/v) DMSO) for 17 h. The internal standard HHQ-*d*4 (5,6,7,8-tetradeutero-2-heptyl-4(1*H*)-quinolone in ACN) was mixed with liquid bacteria culture. After centrifugation, the alkylquinolone concentration in the supernatant was measured by LC-ESI-MS/MS (Dionex Ultimate 3000, Zorbax Eclipse XDB 80 Å C18 5 μm 4.6 × 50 mm (Agilent, Santa Clara, CA, USA)).

### Competitive hER-α Binding Assay

Binding to the human estrogen receptor α was assessed using the commercially available LanthaScreen TR-FRET ER α competitive binding assay (Thermo Fisher Scientific Inc., Waltham, USA) following the provided protocol. In brief, a 12-point 3-fold dilution series of the respective ligands was prepared in DMSO. The ligand’s dilution series (1% v/v final DMSO concentration) was placed in a 384-well plate (Corning 384-well, low volume, round-bottom, black, nontreated surface) and Fluormone ES2 Green (final concentration: 3 nM) was added. Subsequently, a premixed solution of glutathion-S-transferase (GST) tagged hER-α ligand binding domain (final assay concentration: 2.1 nM) and terbium-labeled anti-GST antibody (final assay concentration: 2 nM) was added. The 384-well plate was sealed to minimize evaporation, gently mixed on a plate shaker and incubated at ambient temperature for 1 h. Fluorescence was determined at wavelengths of 515 nm (bandwidth 20 nm) and 486 nm (bandwidth 20 nm) with a delay time of 100 μs and an integration time of 200 μs in a microplate reader (Tecan Spark, Tecan Trading AG, Switzerland) to report the ratio *F*_515_/*F*_486_. IC_50_ values were calculated using GraphPad Prism 10.1.2 with a four-parameter global nonlinear regression model (shared top and bottom values for all data sets) and are reported ± SEM (*n* = 4, except for **20a**: *n* = 2).

### Kinetic Solubility

Kinetic solubility was assessed in saline phosphate buffer pH 7.4 (2.38 g Na_2_HPO_4_·12H_2_O, 0.19 g KH_2_PO_4_, and 8.0 g NaCl in 1 L H_2_O). The pH was adjusted to 7.4 with hydrochloric acid. The buffer was filtered through a 0.45 μM membrane filter prior to use. Kinetic aqueous solubility was determined by nephelometry. Measurements were performed on a NEPHELOstar Plus (BMG LABTECH, Ortenberg, Germany). In brief, a dilution series of the compounds in DMSO (1% (v/v) final DMSO concentration) was prepared to obtain at least three concentrations below and three concentrations above the precipitation point. 198 μL of saline phosphate buffer pH 7.4 were placed in a 96-well plate (Corning UV transparent flat-bottom 96-well plate, Corning Incorporated, Kennebunk, ME, USA) and 2 μL of the compound DMSO stock solution was added and immediately mixed. After shaking (10 s, 500 rpm, double orbital), the measurement was carried out with a laser intensity of 80%, a beam focus of 2.5 mm at 25 °C. With the obtained data points, nephelometric turbidity was plotted against the tested concentrations and the intersection with the baseline (no precipitate) gave the kinetic solubility. The measurements were performed in triplicates and the mean value is reported (±SD).

### Cytotoxicity Assay

HepG2 cells were diluted (2 × 10^5^ cells/mL) in DMEM medium (+1% penicillin/streptomycin, +10% fetal bovine serum), seeded (100 μL/well) to 96-well plates (GREINER, F-bottom), and incubated at 37 °C, 5% CO_2_. The next day, the medium of the cells was aspirated and the respective compound dilutions or DMSO in DMEM medium (1% (v/v) final DMSO concentration, 100 μL) were added to the cells and incubated for 48 h at 37 °C and 5% CO_2_. Ten μL MTT solution (5 mg/mL in PBS, pH = 7.4) was added and incubated for 20 min at 37 °C and 5% CO_2_. The medium was carefully aspirated and 75 μL/well lysis solution (5% (w/v) SDS and 0.5% (v/v) acetic acid in DMSO) was added. The conversion of MTT to formazan was measured photometrically at 570 nm (Pherastar plate reader). IC_50_ values were calculated using GraphPad Prism 10.1.2 with a four-parameter nonlinear regression model and are reported ± SEM (*n* = 2).

### Calculation of Physicochemical Properties

The calculation of physicochemical properties was performed based on a protocol by Geddes et al. using MOE2022.02.^58,78^ Initial structure preparation and 3D minimization was performed using the “QuickPrep” function. Generation of ensembles of conformations was performed using “Conformational Search” and physicochemical descriptors (globularity: glob in MOE, number of rotatable bonds: RotB in MOE, HBD surface area: vsa_don in MOE, polar surface area: vsa_pol in MOE, positive polar surface area: Q_vsa_Ppos in MOE and formal charge: Fcharge in MOE) were calculated. Descriptors were averaged across all conformations for each molecule.

### PAINS Analysis

For the identification of potential pan-assay interference compounds (PAINS), all tested compounds presented in this study were reviewed using http://zinc15.docking.org/patterns/home. Of all biologically tested compounds, **11d–g** were flagged as PAINS due to their similarity to anthranilic acid derivatives.

## Supporting Information Available

Supplementary figures and tables, ITC traces, dose–response curves (pyocyanin inhibition, hER-α, MTT), OD600, alkylquinolone inhibition data, calculation of physicochemical properties, X-ray crystallography information (table, crystallization conditions), ^1^H- and ^13^C-NMR spectra and HPLC chromatograms for biologically tested compounds (PDF)
Molecular formula strings with biological data (CSV)
Coordinates of modeled structures of *Bc*PhzB and **9c** (PDB)
Coordinates of modeled structures of *Bc*PhzB and **10c** (PDB)
Coordinates of modeled structures of *Bc*PhzB and **11g** (PDB)
Coordinates of modeled structure of *Bc*PhzB for docking of **9***c***/10c** (PDB)
Coordinates of modeled structure of *Bc*PhzB for docking of **11g** (PDB)

## Supplementary Material

jm4c03065_si_002.csv

jm4c03065_si_003.pdb

jm4c03065_si_004.pdb

jm4c03065_si_005.pdb

jm4c03065_si_006.pdb

jm4c03065_si_007.pdb

## Abbreviations Used

2-AA: 2-aminoacetophenone
AhR: aryl hydrocarbon receptor
AMR: antimicrobial resistance
CRPA: carbapenem-resistant *Pseudomonas aeruginosa*
DHQ: 2,4-dihydroxyquinoline
EtOAc: ethyl acetate
FCA: Friedel crafts acylation
GST: glutathione-S-transferase
hER-α: human estrogen receptor-α
HHQ: 2-heptyl-4-quinolone
HQNO: *N*-oxo-2-heptyl-4-hydroxyquinoline
ITC: isothermal titration calorimetry
*K*_d_: dissociation constant
MES: 2-morpholinoethanesulfonic acid
QS: quorum sensing
*n*DSF: nano differential scanning fluorimetry
PQS: 2-heptyl-3-hydroxy-4-quinolone
PYO: pyocyanin
RAL: raloxifene
SD: standard deviation
SEM: standard error of the mean
*T*_m_: protein unfolding temperature
*t*_M_: migration time
TR-FRET: time-resolved Förster resonance energy transfer
