# Supplementary material for: From Bones to Bugs: Structure-Based Development of Raloxifene-Derived Pathoblockers That Inhibit Pyocyanin Production in Pseudomonas aeruginosa
Source: J Med Chem. 2025 Mar 29;68(7):7390–420. doi: 10.1021/acs.jmedchem.4c03065 (PMC11998002; doi:10.1021/acs.jmedchem.4c03065)
Supplement: Supplementary file 1 — jm4c03065_si_001.pdf [file jm4c03065_si_001.pdf]

Supporting Information

From Bones to Bugs: Structure-Based Development of Raloxifene-Derived Pathoblockers That Inhibit Pyocyanin Production in *Pseudomonas aeruginosa*

Marie Thiemann[‡,a,b,c], Moritz Zimmermann[‡,a,b,c], Christina Diederich[b], Huilin Zhan[d,e,f,g], Mikhail Lebedev[h], Jakob Pletz[i], Janosch Baumgarten[a,b,c], Maria Handke[a,b,c], Mathias Müsken[b], Rolf Breinbauer[i], Gabriela Krasteva-Christ[f,g], Esther Zanin[h], Martin Empting[d,e,g,j], Matthias Schiedel[*,a,c], Conrad Kunick[*,a,c], Wulf Blankenfeldt[*,b,k]

[a] Institute of Medicinal and Pharmaceutical Chemistry, Technische Universität Braunschweig, Beethovenstraße 55, 38106 Braunschweig, Germany

[b] Helmholtz Centre for Infection Research (HZI), Inhoffenstraße 7, 38124 Braunschweig, Germany

[c] Center of Pharmaceutical Engineering (PVZ), Technische Universität Braunschweig, Franz-Liszt-Straße 35a, 38106 Braunschweig, Germany

[d] Helmholtz-Institute for Pharmaceutical Research Saarland (HIPS) - Helmholtz Centre for Infection Research (HZI), Campus E8.1, 66123 Saarbrücken, Germany

[e] Saarland University, Department of Pharmacy, Campus E8.1, 66123 Saarbrücken, Germany

[f] Saarland University, Faculty of Medicine, Institute for Anatomy and Cell Biology & Center for Gender-specific Biology and Medicine (CGBM), Kirrbergerstr. 100, 66424 Homburg/Saar, Germany

[g] PharmaScienceHub (PSH), 66123 Saarbrücken, Germany

[h] Friedrich-Alexander-Universität Erlangen-Nürnberg, Department Biologie, 91058 Erlangen, Germany

[i] Institute of Organic Chemistry, Graz University of Technology, Stremayrgasse 9, 8010 Graz, Austria

[j] German Centre for Infection Research (DZIF), Partner Site Hannover-Braunschweig, 38124 Braunschweig, Germany

[k] Institute of Biochemistry, Biotechnology and Bioinformatics, Technische Universität Braunschweig, Spielmannstraße 7, 38106 Braunschweig, Germany

*E-mail: W.B., for structual biology/biochemistry: e-mail, wulf.blankenfeldt@helmholtz-hzi.de; C.K., for synthetic chemistry: e-mail, c.kunick@tu-braunschweig.de; M.S., for medicinal chemistry: e-mail, matthias.schiedel@tu-braunschweig.de

[‡]: These authors contributed equally.

Table of Contents

Page	Contents
S3 – S21	Supplementary Figures
S22 – S30	Supplementary Tables
S31 – S77	Supplementary NMR spectra of novel compounds
S78 – S94	Supplementary HPLC traces of biologically tested compounds
S95	Supplementary References

Supplementary Figures

```
BcPhzB        MSDVESLENTSENRAQVAARQHNRKIVEQYMHTRGEARLKRHLLFTEDGVGGLWTTDSGQ  60
Pa14PhzB1     MPDTT---NPIGFTDANELREKNRATVEKYMNTKGQDRLRRHELFVEDGCGGLWTTDTGS  57
Pa14PhzB2     MLDNA---IPQGFEDAVELRRKNRETVVKYMNTKGQDRLRRHELFVEDGCGGLWTTDTGS  57
Pa14PhzA1     MNGQR---YRETPLDIERLRRLNRATVERYMAMKGAERLQRHSLFVEDGCAGNWTTESGE  57
Pa14PhzA2     MREYQ---RLKGFTDNLELRRRNRATVEHYMRMKGAERLQRHSLFVEDGCAGNWTTESGE  57

BcPhzB        PIAIRGREKLGEHAVWSLQCFPDWVWTDIQIFETQDPNWFWVECRGEGAIVFPGYPRGQY  120
Pa14PhzB1     PIVIRGKDKLAEHAVWSLKCFPDWEWYNINIFGTDDPNHFWVECDGHGKILFPGYPEGYY  117
Pa14PhzB2     PIVIRGKDKLAEHAVWSLKCFPDWEWYNIKVFETDDPNHFWVECDGHGKILFPGYPEGYY  117
Pa14PhzA1     PLVFRGHESLRRLAEWLERCFPDWEWRNVRIFETEDPNHFWVECDGRGKALVPGYPQGYC  117
Pa14PhzA2     PLVFRGHESLRRLAEWLERCFPDWEWHNVRIFETEDPNHLWVECDGRGKALVPGYPQGYC  117

BcPhzB        RNHFLHSFRFENGLIKEQREFMNPCEQFRSLGIEVPEVRRDGLPS-  165
Pa14PhzB1     ENHFLHSFELEDGKIKRNREFMNVFQQLRALSIPVPEIKREGIPT-  162
Pa14PhzB2     ENHFLHSFELDDGKIKRNREFMNVFQQLRALSIPVPQIKREGIPT-  162
Pa14PhzA1     ENHYIHSFELENGRIKRNREFMNPMQKLRALGIAVPQIKRDGIPT-  162
Pa14PhzA2     ENHYIHSFELENGRIKRNREFMNPMQKLRALGIAVPQIKRDGIPT-  162
```

Figure S1: Sequence alignment of PhzB from *Burkholderia cepacia* (*Bc*PhzB) and $PhzA_1$, $PhzA_2$, $PhzB_1$ and $PhzB_2$ from *Pseudomonas aeruginosa* PA14. Highlighted are amino acids that are involved in substrate binding (blue) and catalysis (yellow) as well as amino acids involved in the binding of the parent compound RAL (**6**, green). Sequence alignment was performed using Clustal Omega.[1]

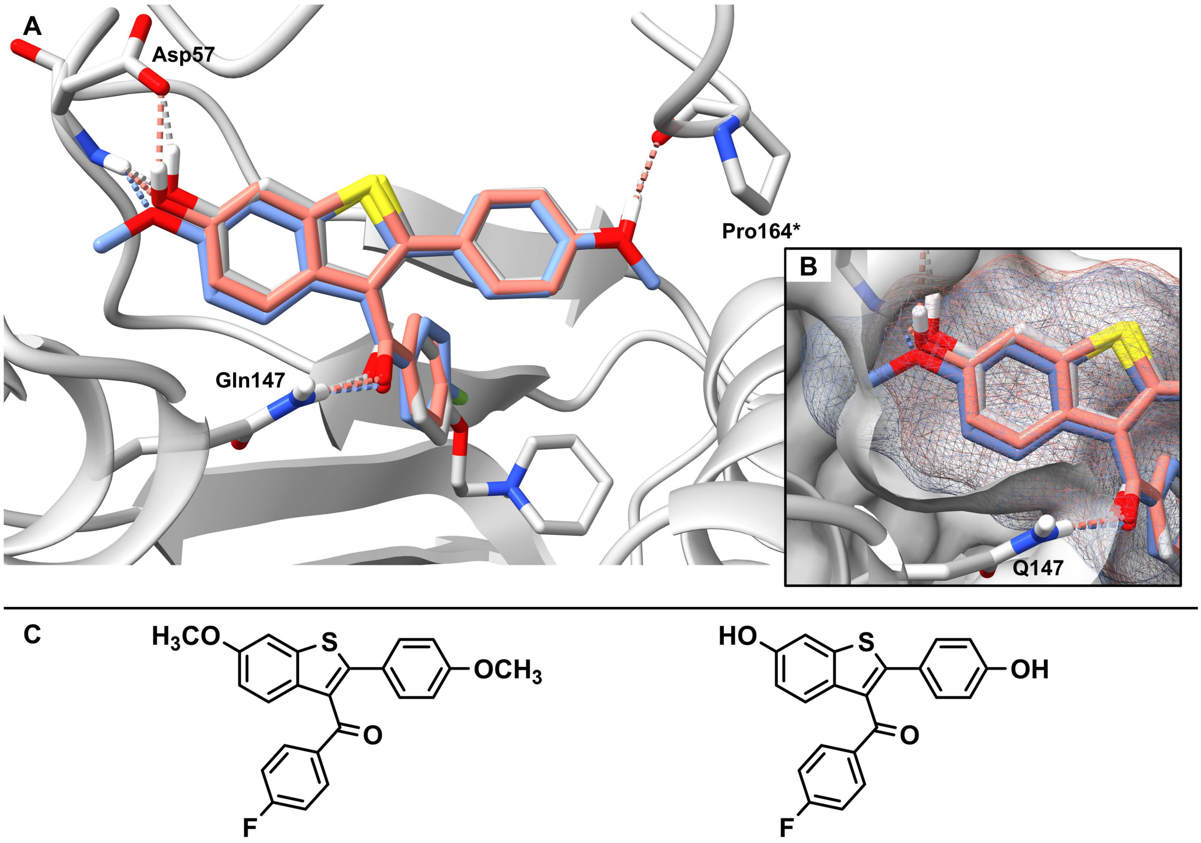

9c **10c**

Figure S2: A. Comparison of docking poses of 6,4'-dimethoxy compound **9c** (blue) and 6,4'-dihydroxy compound **10c** (red) in *Bc*PhzB. **B.** Close up: The 6-methoxy substituent does not fit into the binding pocket of *Bc*PhzB **C.** Chemical structures of **9c** and **10c**.

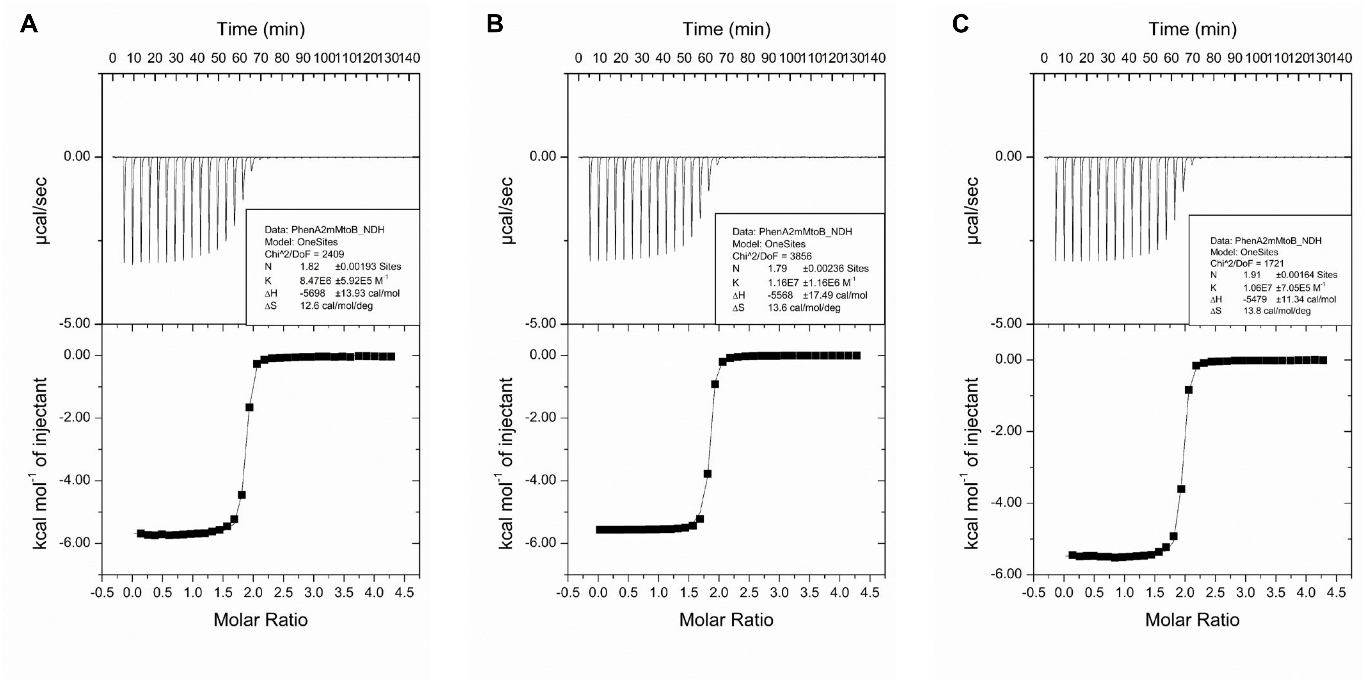

Figure S3: ITC traces for phenazistatin A (**5**). **A – C:** Phenazistatin A (**5**) (2 mM) titrated against *Bc*PhzB (100 µM).

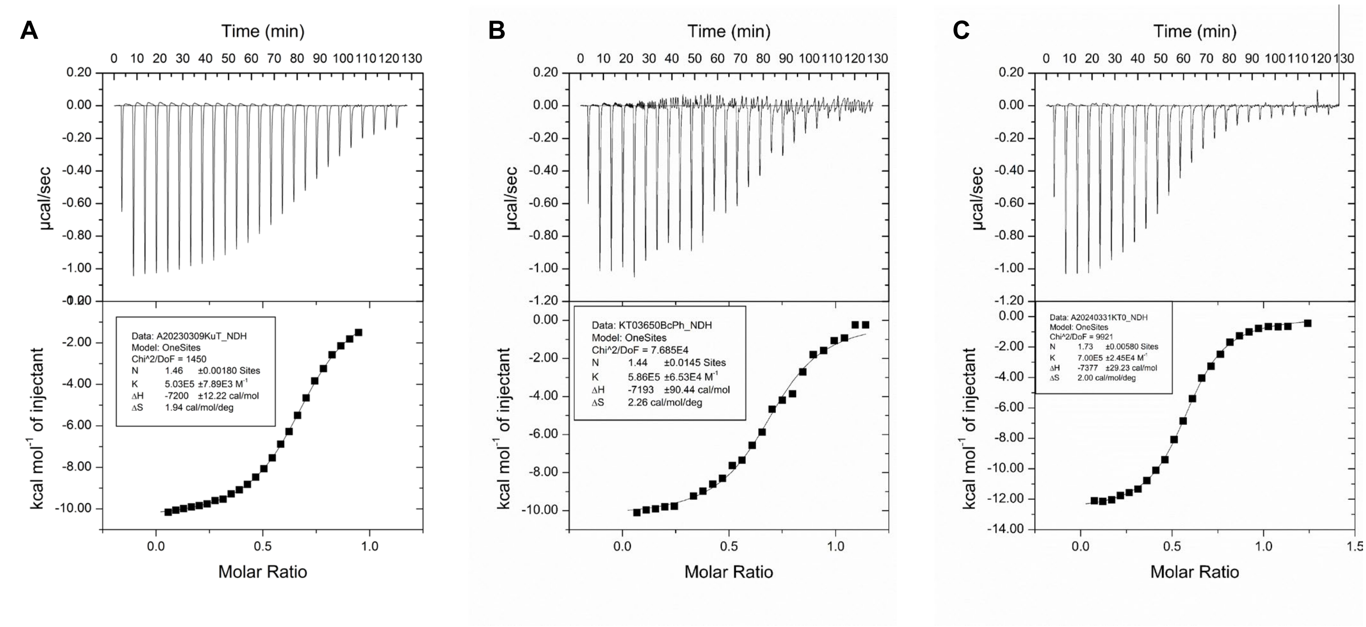

Figure S4: ITC traces for **10a**. **A:** *Bc*PhzB (300 µM) titrated against **10a** (60 µM). **B.** *Bc*PhzB (300 µM) titrated against **10a** (50 µM). **C:** *Bc*PhzB (260 µM) titrated against **10a** (40 µM).

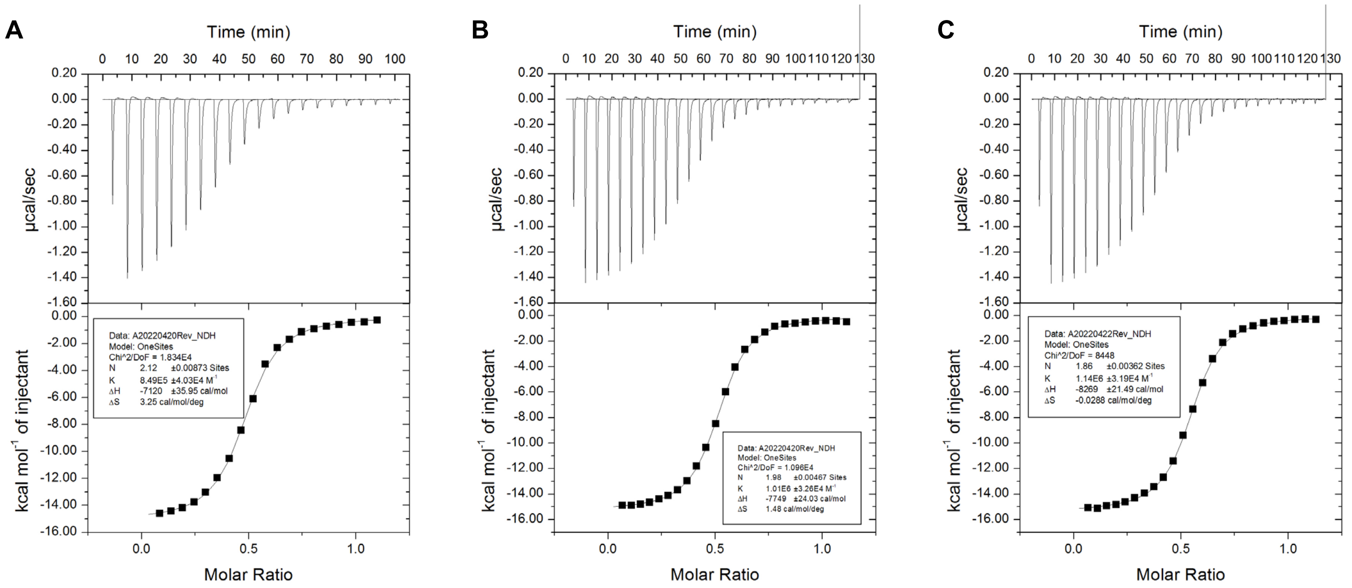

Figure S5: ITC traces for **10b**. **A:** *Bc*PhzB (300 µM) titrated against **10b** (40 µM). **B:** *Bc*PhzB (300 µM) titrated against **10b** (50 µM). **C:** *Bc*PhzB (260 µM) titrated against **10b** (50 µM).

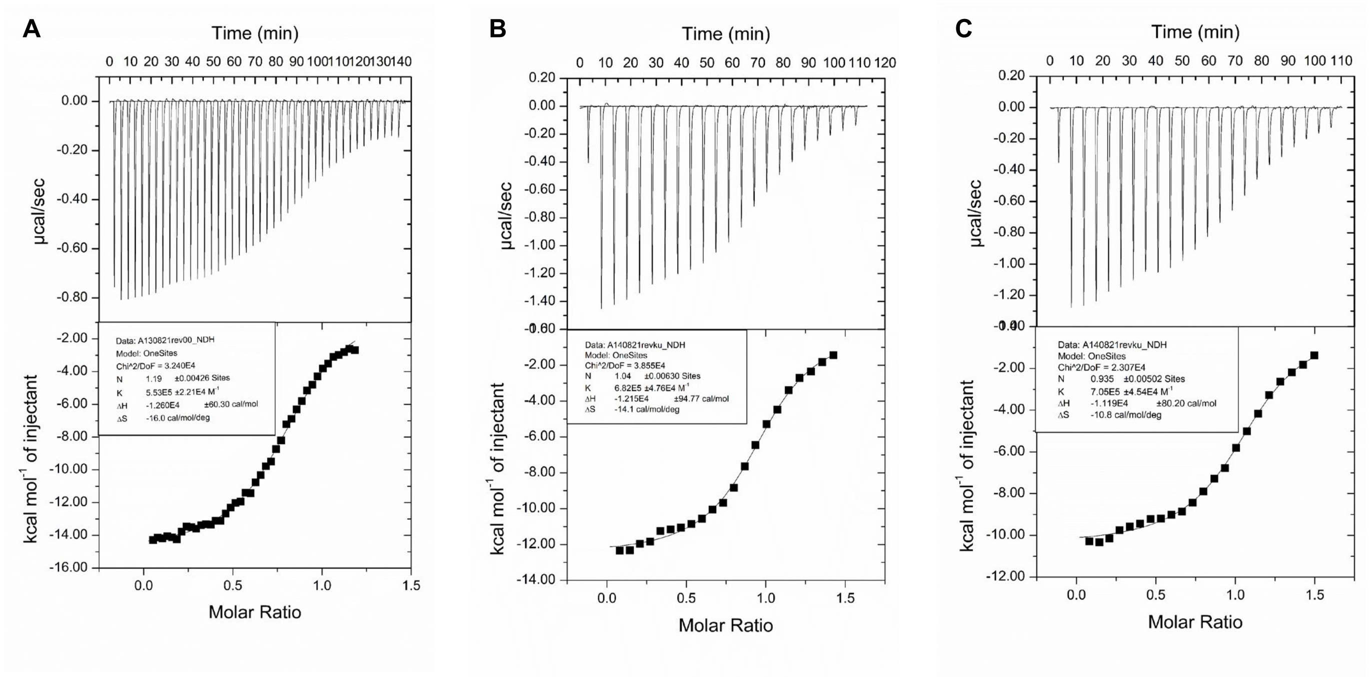

Figure S6: ITC traces for **10c**. **A:** *Bc*PhzB (280 µM) titrated against **10c** (40 µM). **B/C:** *Bc*PhzB (350 µM) titrated against **10c** (40 µM).

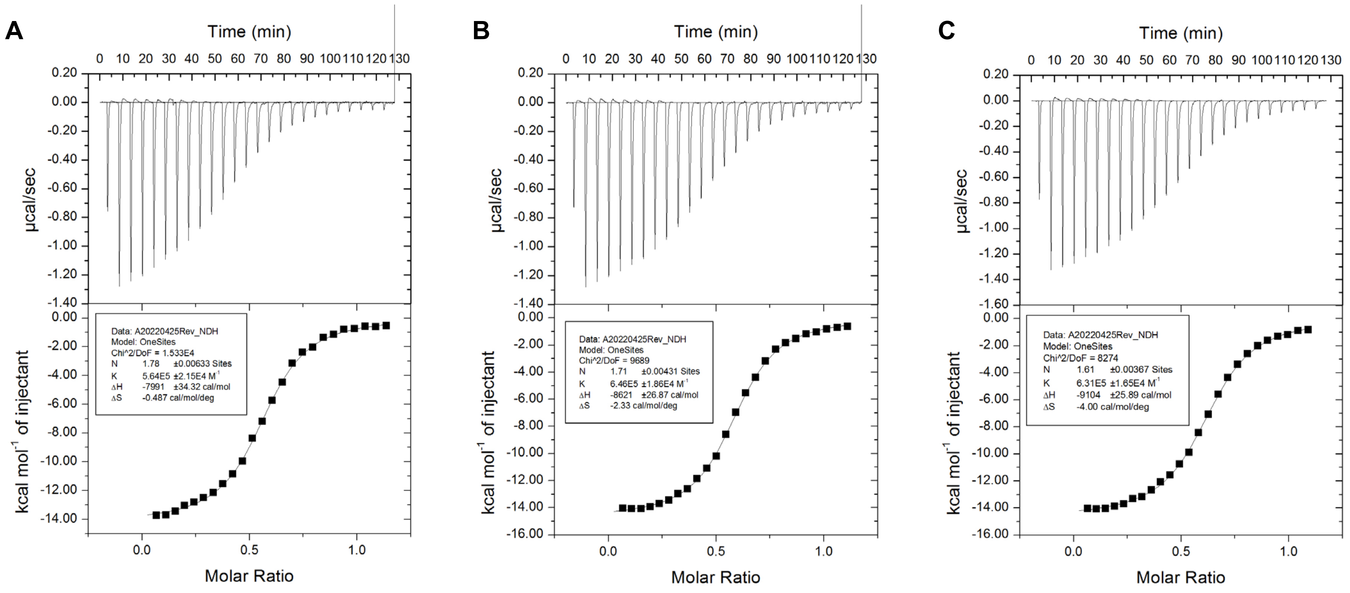

Figure S7: ITC traces for **10d**. **A – C:** *Bc*PhzB (300 µM) titrated against **10d** (50 µM).

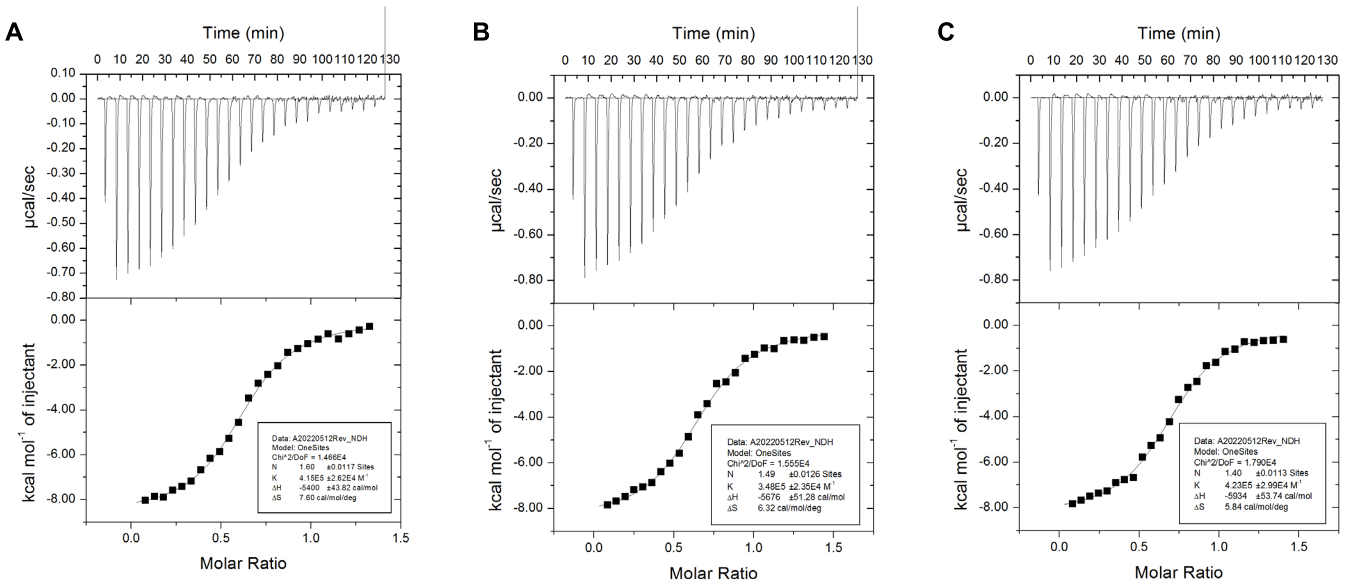

Figure S8: ITC traces for **10h**. **A – C:** *Bc*PhzB (300 µM) titrated against **10h** (40 µM).

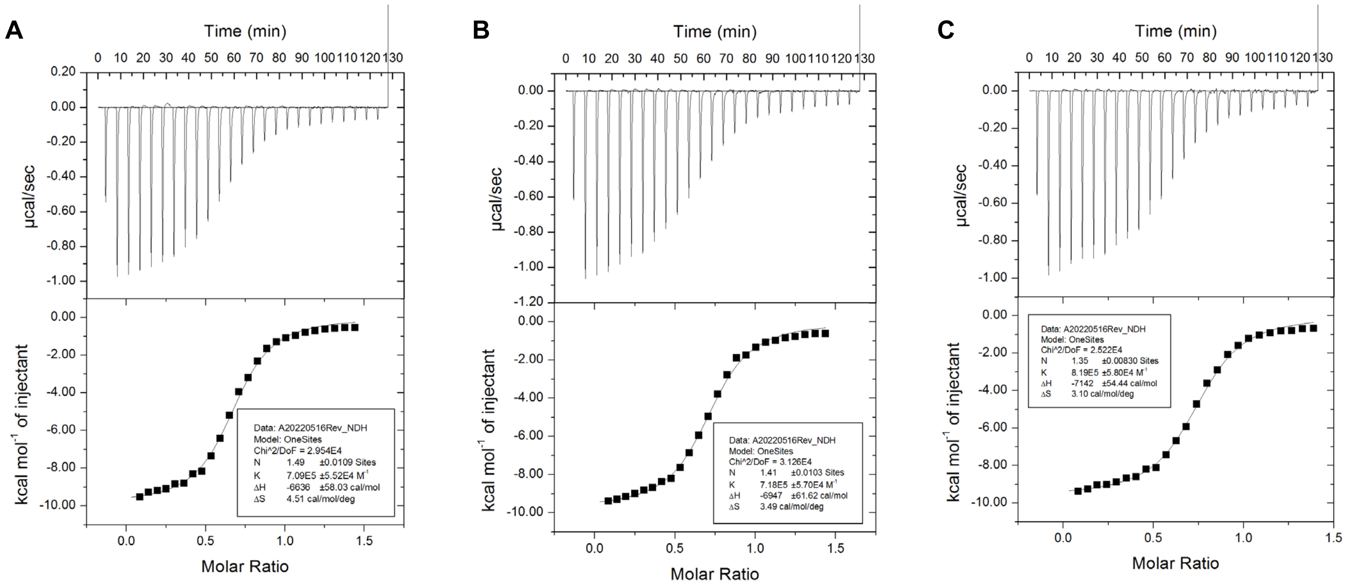

Figure S9: ITC traces for **10j**. **A – C:** *Bc*PhzB (300 µM) titrated against **10j** (40 µM).

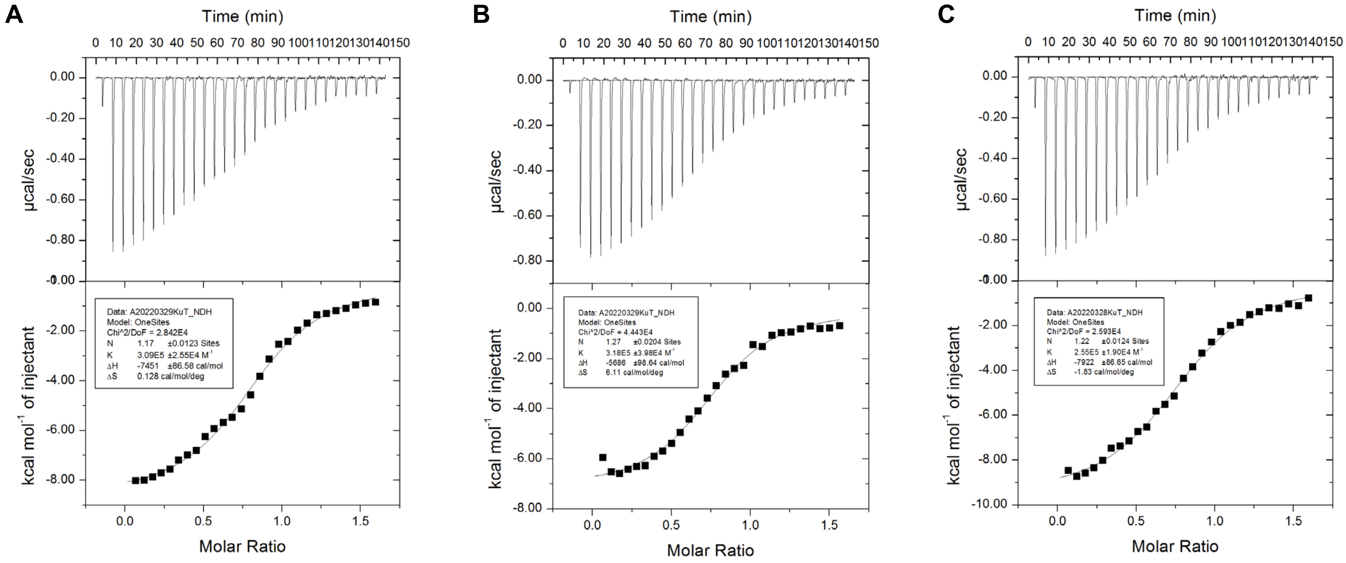

Figure S10: ITC traces for **10l**. **A – C:** *Bc*PhzB (300 µM) titrated against **10l** (40 µM).

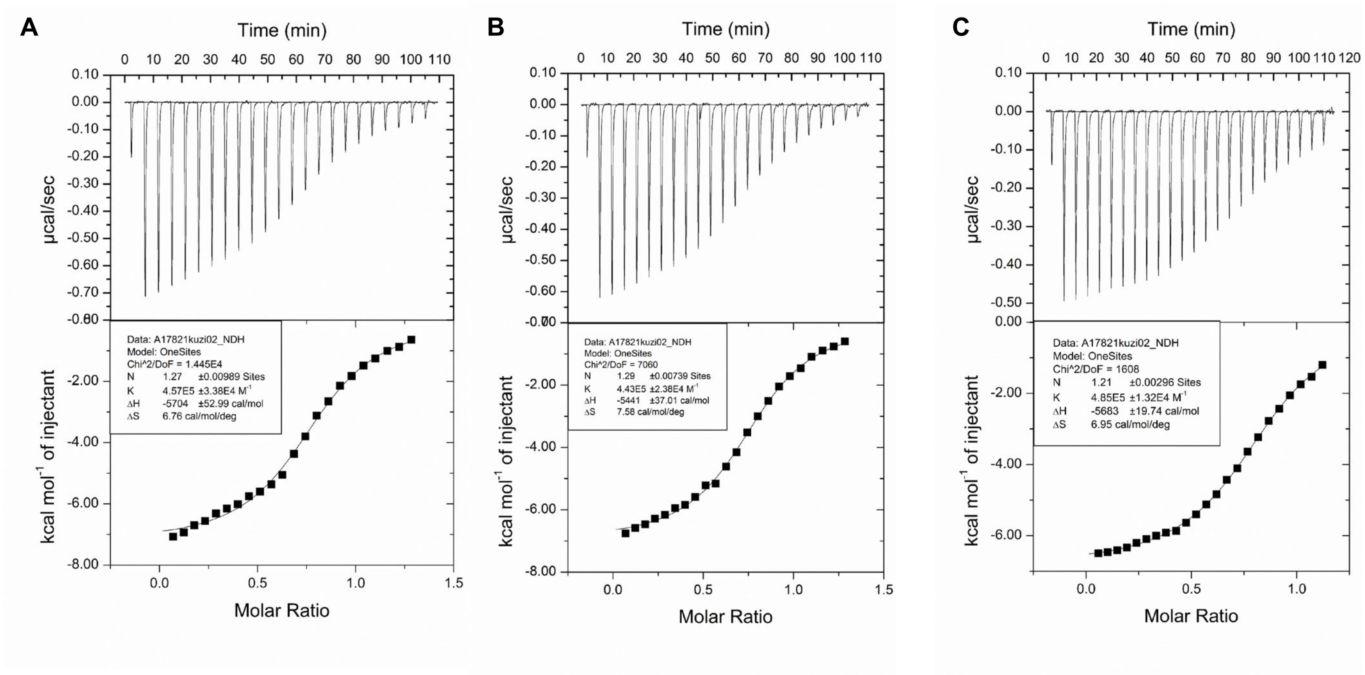

Figure S11: ITC traces for **13a**. **A/B:** *Bc*PhzB (300 µM) titrated against **13a** (40 µM). **C:** *Bc*PhzB (250 µM) titrated against **13a** (40 µM).

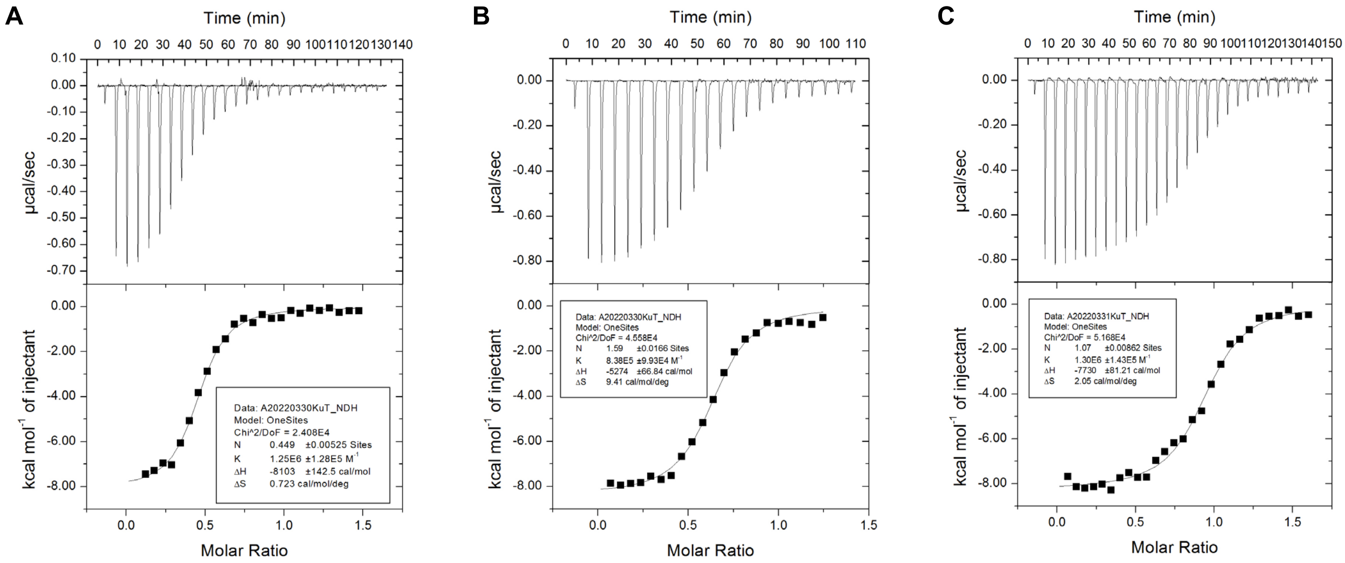

Figure S12: ITC traces for **13d**. **A:** *Bc*PhzB (300 µM) titrated against **13d** (30 µM). **B/C:** *Bc*PhzB (300 µM) titrated against **13d** (40 µM).

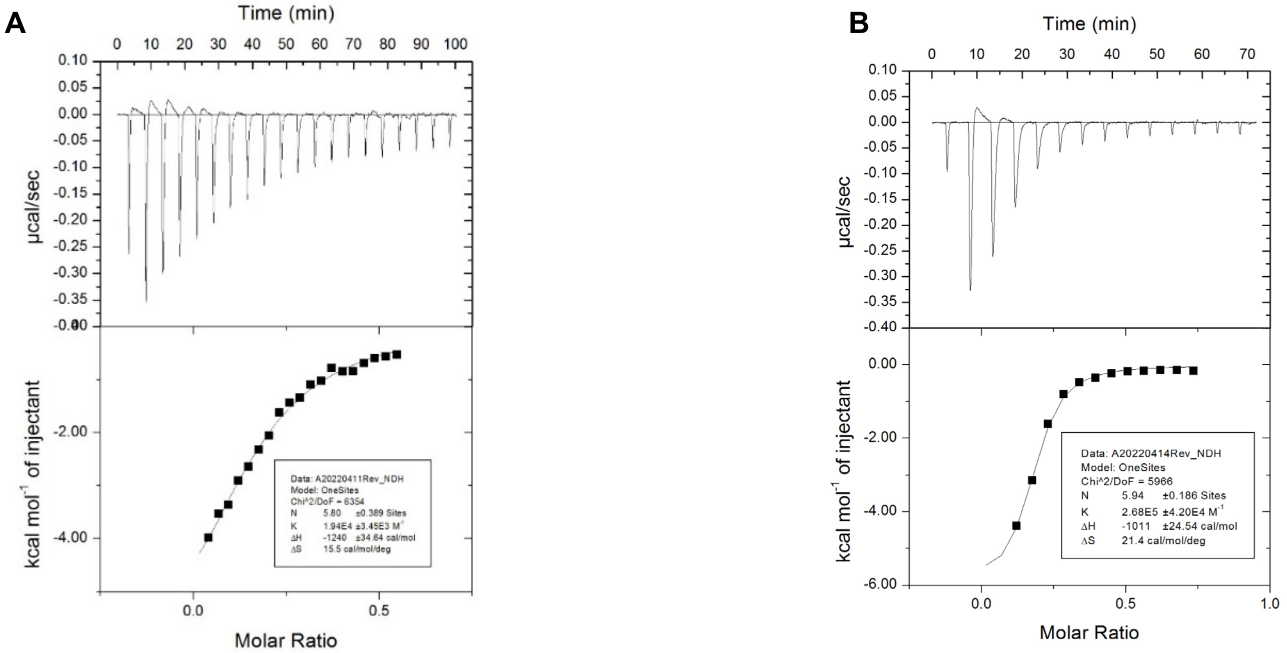

Figure S13: ITC traces for RAL (**6**) (**A**) and **11a** (**B**). Both titration curves have a low *c*-value and are therefore not analysable. Setting the stoichiometry N to 1 did not give interpretable results either. **A.** *Bc*PhzB (300 µM) titrated against RAL (**6**) (80 µM). **B.** *Bc*PhzB (300 µM) titrated against **11a** (40 µM).

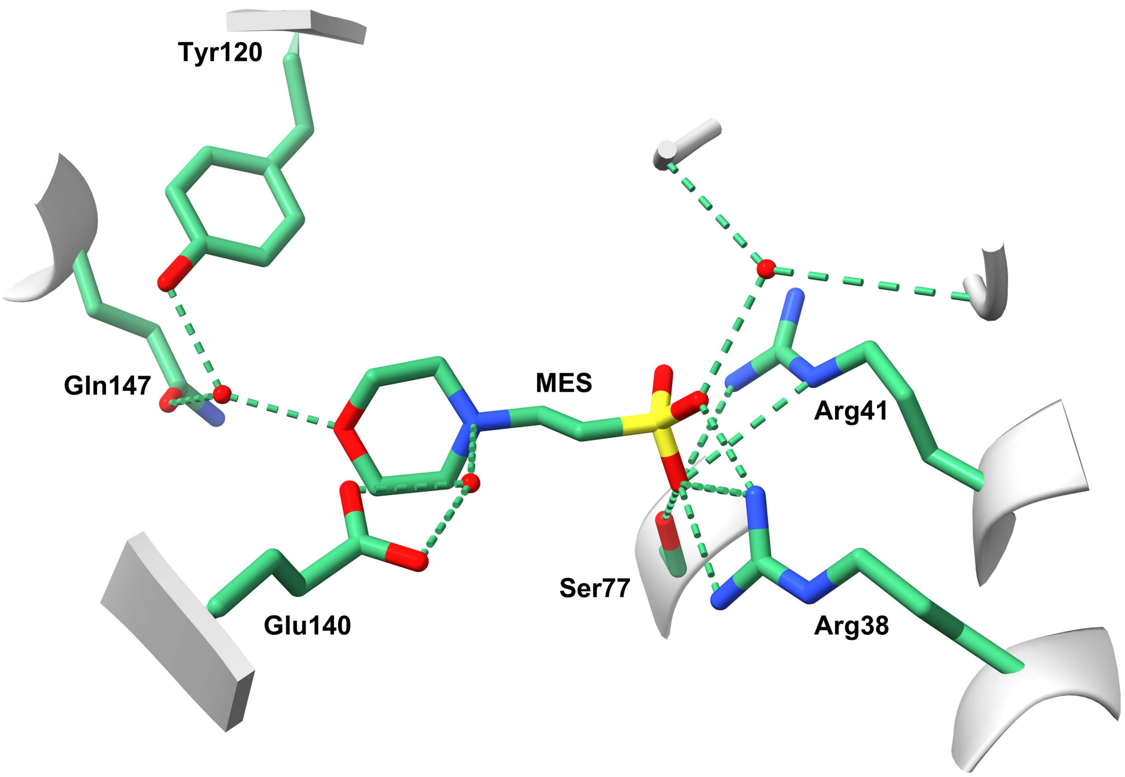

Figure S14: Binding mode of 2-morpholinoethanesulfonic acid (MES) in the cocrystal structure of **10i** with *Bc*PhzB (PDB: 9F8J). MES was part of the crystallization buffer in several conditions and was observed in the binding site of the cocrystal structures of **10i**, **10j**, **13a** and **20a** with *Bc*PhzB.

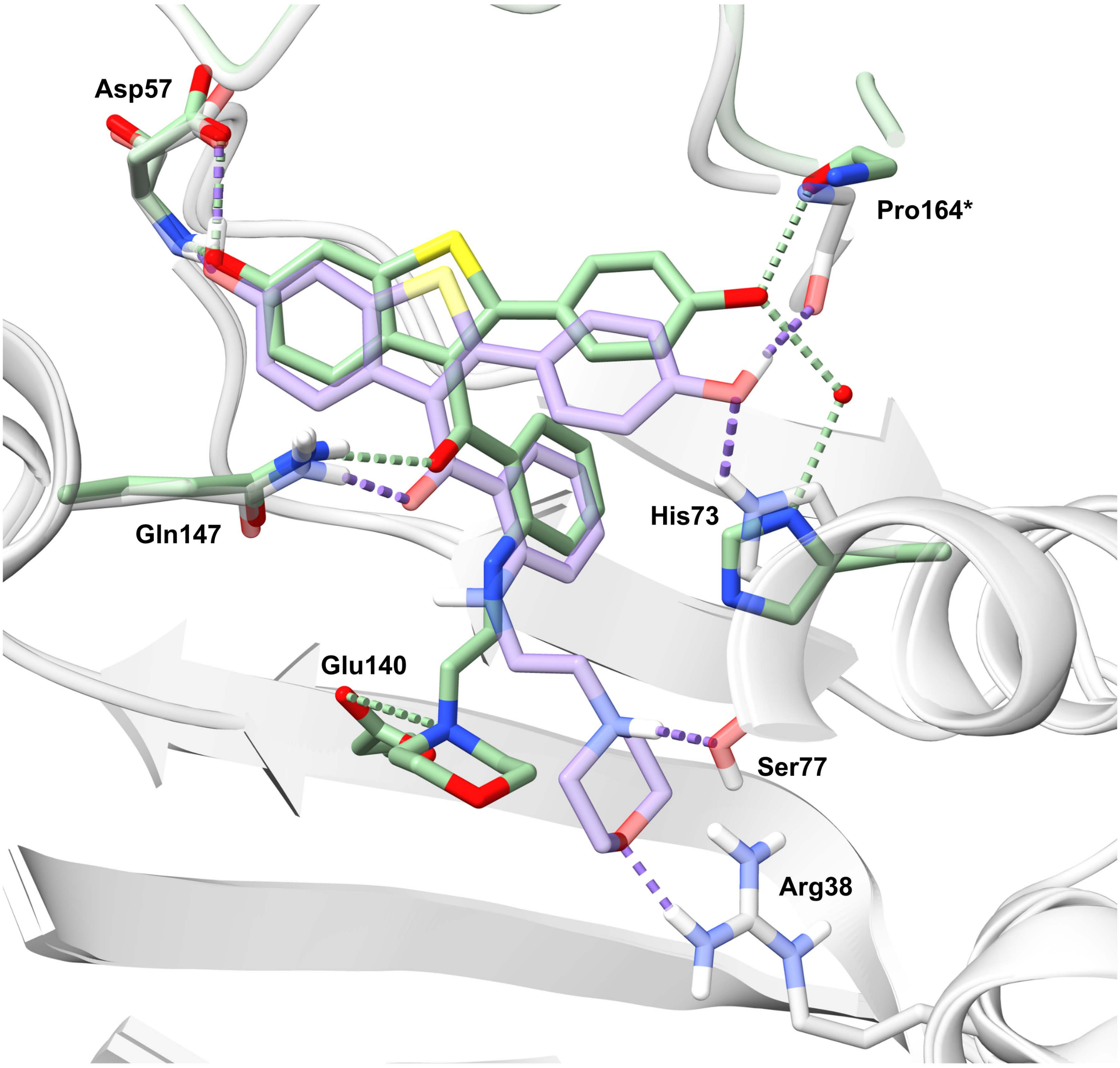

Figure S15: Docking of **11g** in the binding pocket of *Bc*PhzB (purple) and comparison to the obtained cocrystal structure with *Bc*PhzB (green, PDB: 9F8O). In contrast to the docking calculations, the 4'-hydroxy group forms no direct, but a water mediated hydrogen bond to His73. Furthermore, the predicted interactions of the morpholine ring with Arg38 and Ser77 are not formed, instead a hydrogen bond of the morpholine nitrogen to Glu140 was observed.

Figure S16: Pyocyanin reduction dose-response curves of IC_{50} determination for compounds **6**, **10a – l**, **11a – g**, **13a**, **13c – g**, **15a – c** and **20a – d**. Pyocyanin reduction in *P. aeruginosa* PA14 was determined after aerobic incubation with test compounds (16 h, 37 °C, 225 rpm) relative to DMSO control. #Regression analysis was performed using the pyocyanin production of a *P. aeruginosa* PA14 *phzB1B2*-knockout mutant (Δ*phzB1B2*) as bottom constraint.

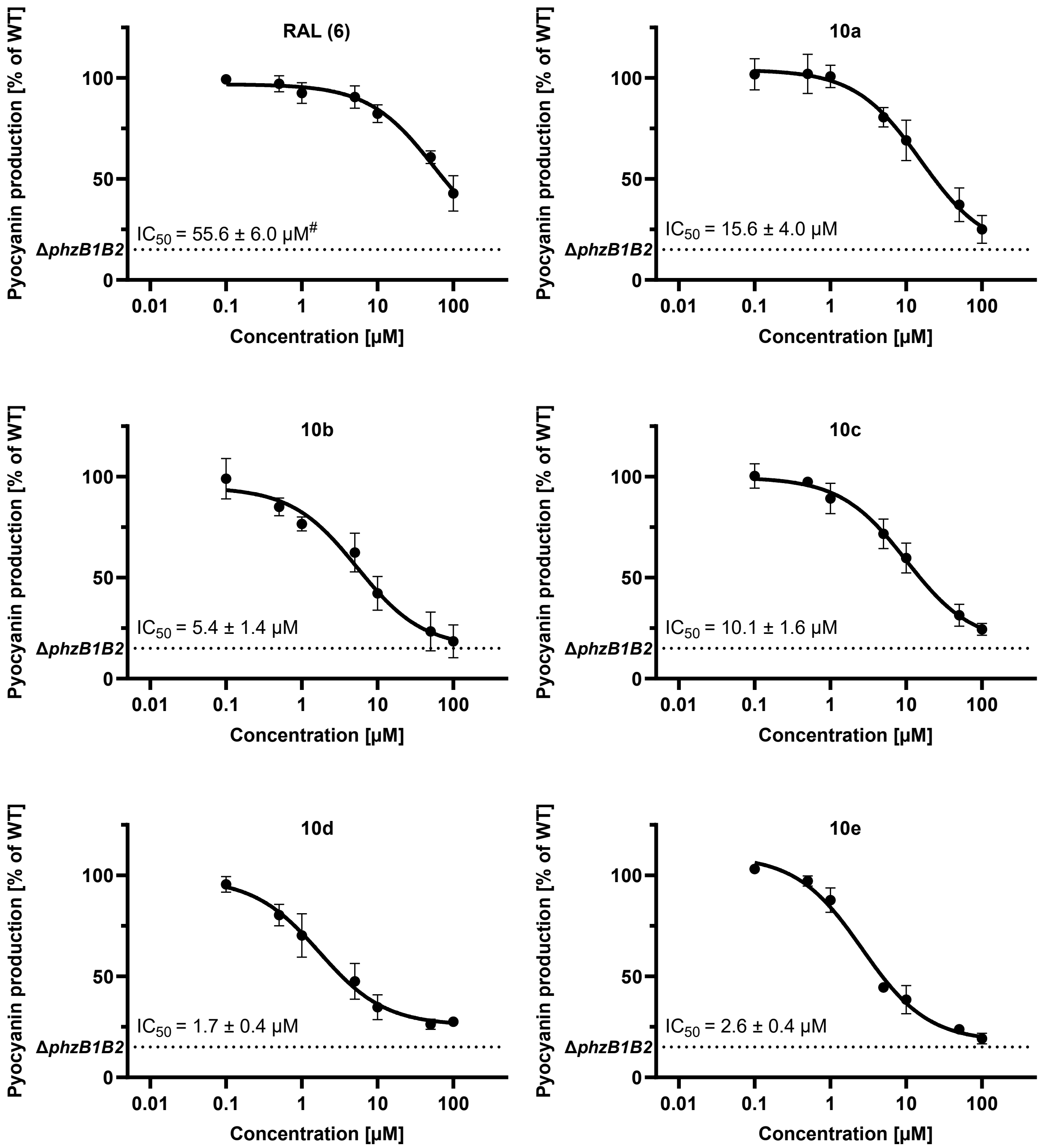

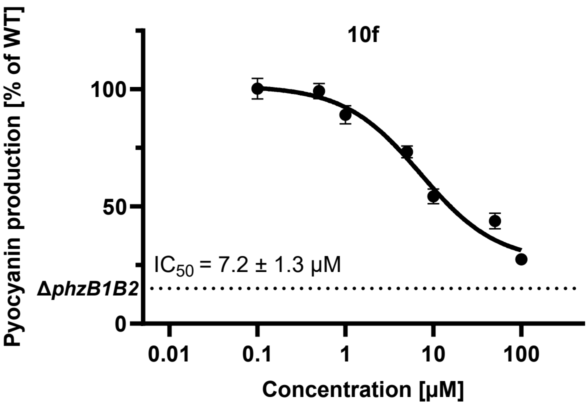
10f
IC$_{50}$ = 7.2 ± 1.3 μM
Δ*phzB1B2*
Pyocyanin production [% of WT]
0
50
100
0.01
0.1
1
10
100
Concentration [μM]

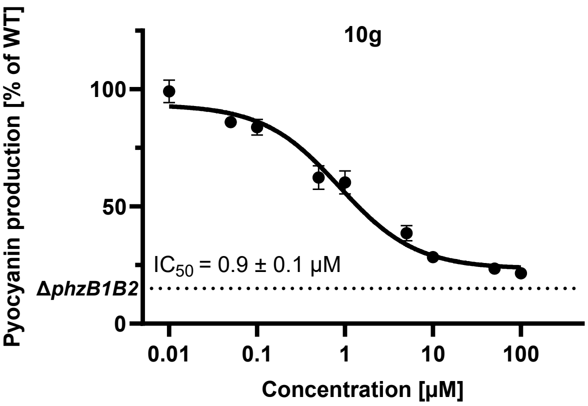
10g
IC$_{50}$ = 0.9 ± 0.1 μM
Δ*phzB1B2*
Pyocyanin production [% of WT]
0
50
100
0.01
0.1
1
10
100
Concentration [μM]

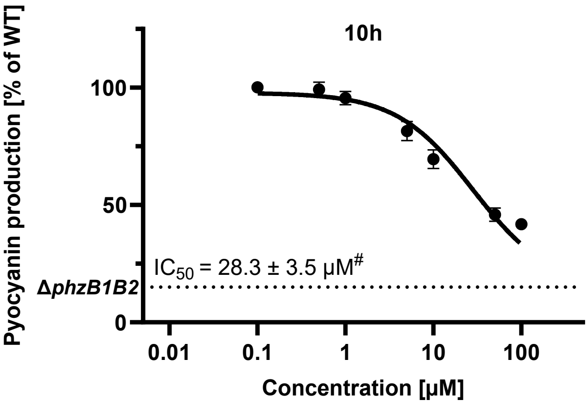
10h
IC$_{50}$ = 28.3 ± 3.5 μM$^{\#}$
Δ*phzB1B2*
Pyocyanin production [% of WT]
0
50
100
0.01
0.1
1
10
100
Concentration [μM]

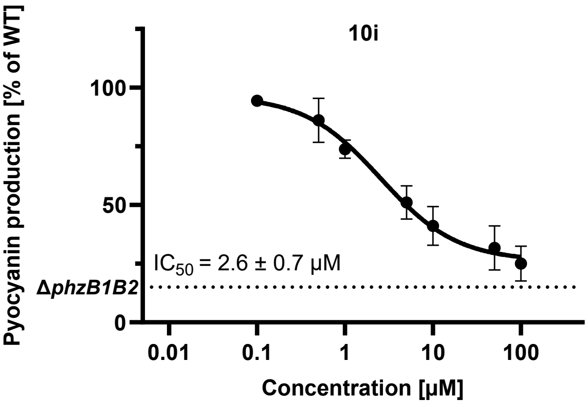
10i
IC$_{50}$ = 2.6 ± 0.7 μM
Δ*phzB1B2*
Pyocyanin production [% of WT]
0
50
100
0.01
0.1
1
10
100
Concentration [μM]

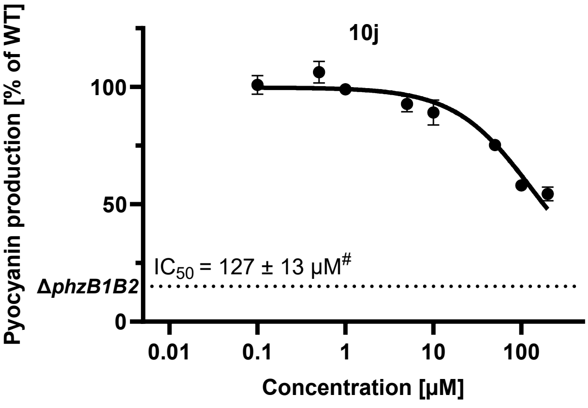
10j
IC$_{50}$ = 127 ± 13 μM$^{\#}$
Δ*phzB1B2*
Pyocyanin production [% of WT]
0
50
100
0.01
0.1
1
10
100
Concentration [μM]

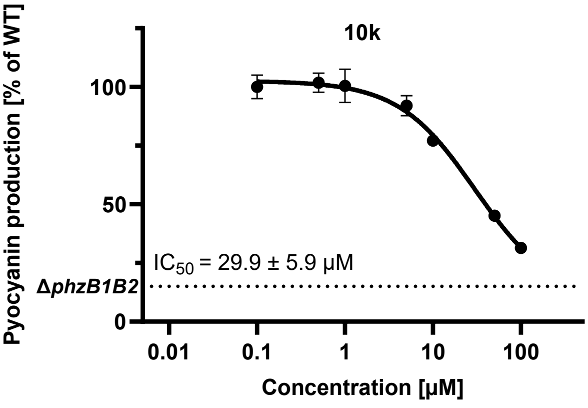
10k
IC$_{50}$ = 29.9 ± 5.9 μM
Δ*phzB1B2*
Pyocyanin production [% of WT]
0
50
100
0.01
0.1
1
10
100
Concentration [μM]

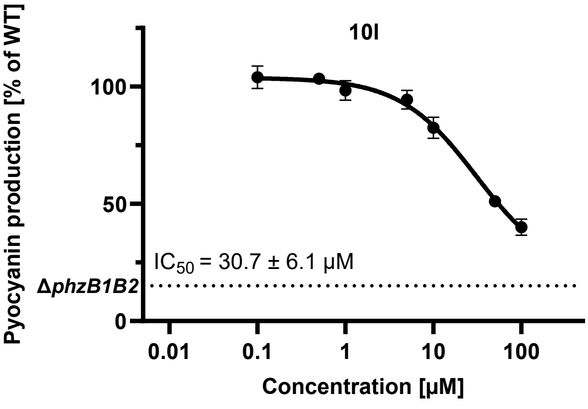
10l
IC$_{50}$ = 30.7 ± 6.1 μM
Δ*phzB1B2*
Pyocyanin production [% of WT]
0
50
100
0.01
0.1
1
10
100
Concentration [μM]

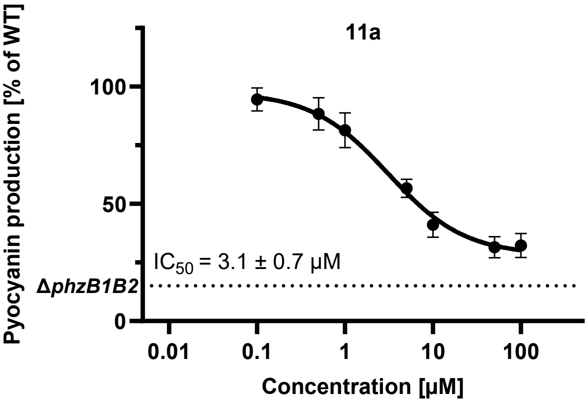
11a
IC$_{50}$ = 3.1 ± 0.7 μM
Δ*phzB1B2*
Pyocyanin production [% of WT]
0
50
100
0.01
0.1
1
10
100
Concentration [μM]

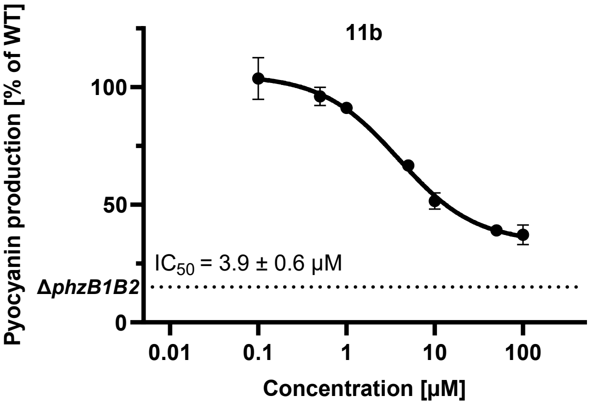
11b
Pyocyanin production [% of WT]
100
50
0
ΔphzB1B2
IC50 = 3.9 ± 0.6 µM
0.01
0.1
1
10
100
Concentration [µM]

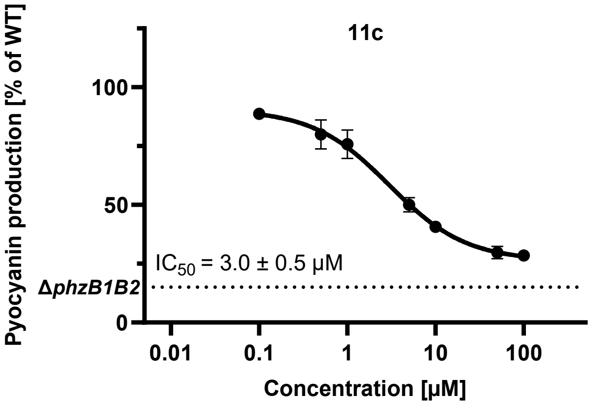
11c
Pyocyanin production [% of WT]
100
50
0
ΔphzB1B2
IC50 = 3.0 ± 0.5 µM
0.01
0.1
1
10
100
Concentration [µM]

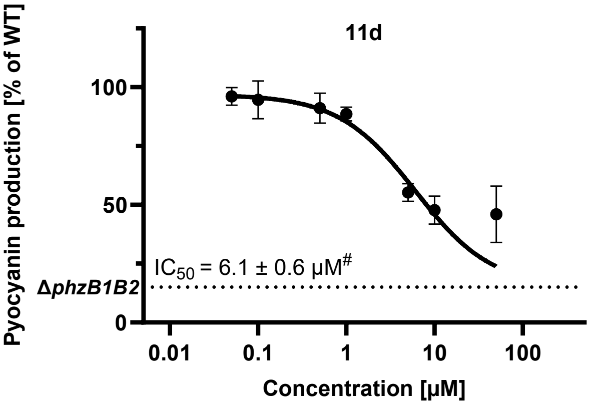
11d
Pyocyanin production [% of WT]
100
50
0
ΔphzB1B2
IC50 = 6.1 ± 0.6 µM#
0.01
0.1
1
10
100
Concentration [µM]

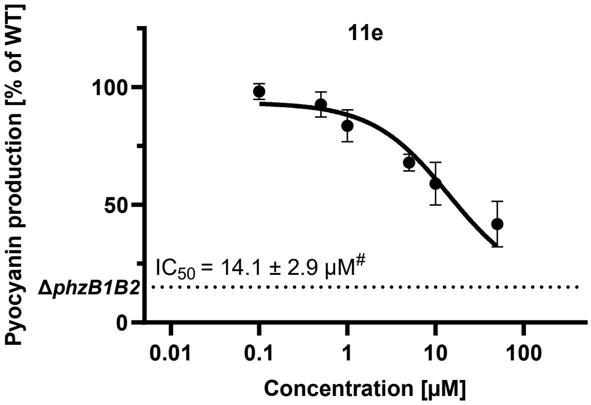
11e
Pyocyanin production [% of WT]
100
50
0
ΔphzB1B2
IC50 = 14.1 ± 2.9 µM#
0.01
0.1
1
10
100
Concentration [µM]

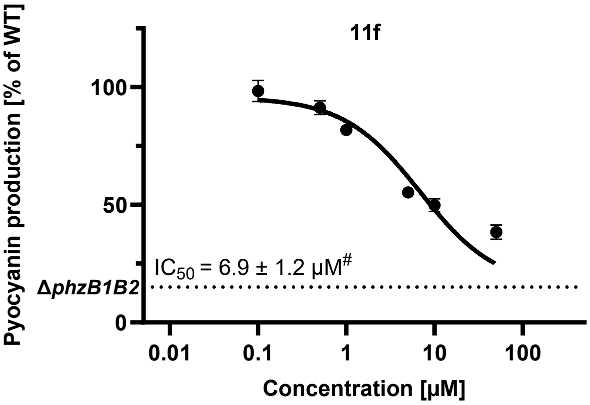
11f
Pyocyanin production [% of WT]
100
50
0
ΔphzB1B2
IC50 = 6.9 ± 1.2 µM#
0.01
0.1
1
10
100
Concentration [µM]

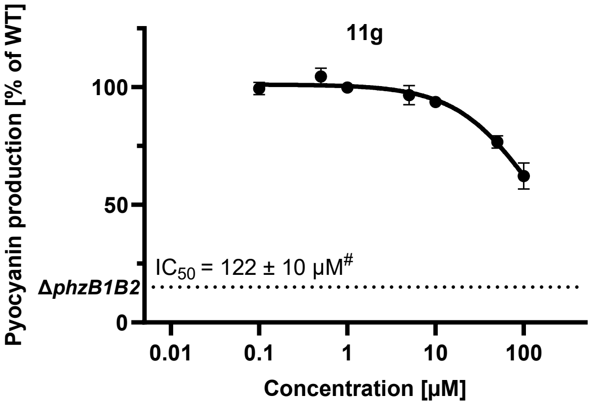
11g
Pyocyanin production [% of WT]
100
50
0
ΔphzB1B2
IC50 = 122 ± 10 µM#
0.01
0.1
1
10
100
Concentration [µM]

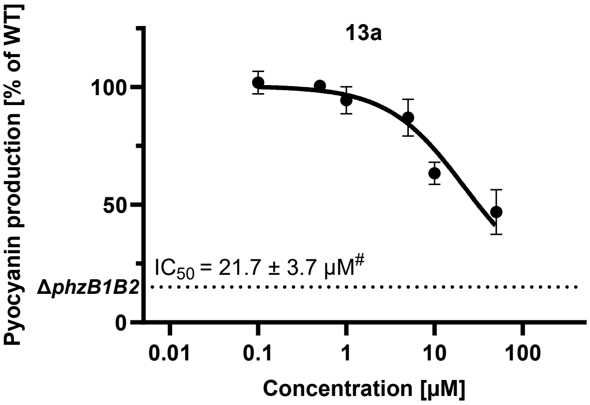
13a
Pyocyanin production [% of WT]
100
50
0
ΔphzB1B2
IC50 = 21.7 ± 3.7 µM#
0.01
0.1
1
10
100
Concentration [µM]

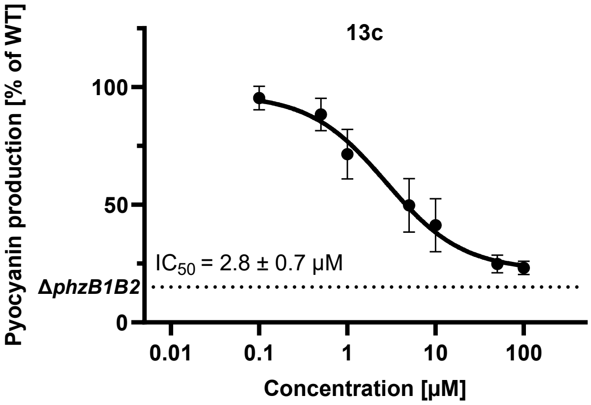
13c
Pyocyanin production [% of WT]
100
50
0
ΔphzB1B2
IC50 = 2.8 ± 0.7 µM
0.01
0.1
1
10
100
Concentration [µM]

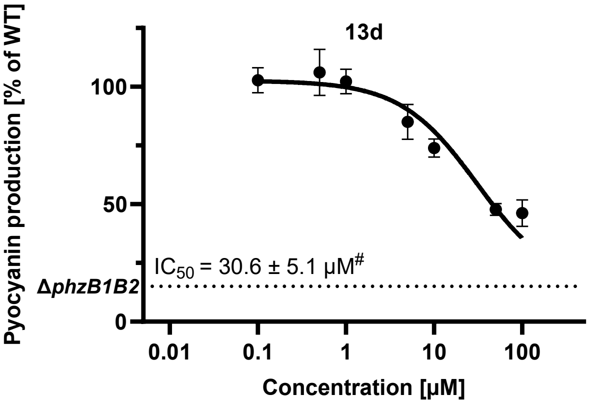
13d
Pyocyanin production [% of WT]
100
50
ΔphzB1B2
0
IC$_{50}$ = 30.6 ± 5.1 µM#
0.01
0.1
1
10
100
Concentration [µM]

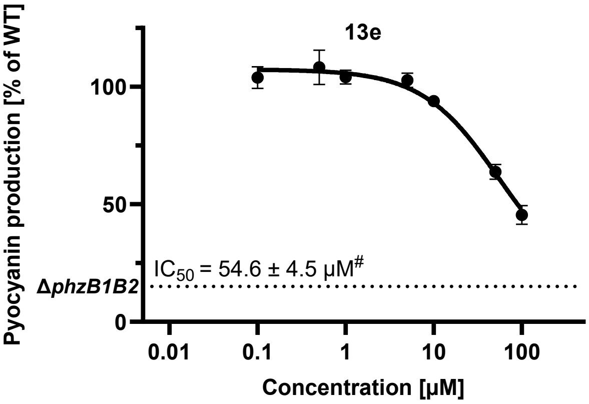
13e
Pyocyanin production [% of WT]
100
50
ΔphzB1B2
0
IC$_{50}$ = 54.6 ± 4.5 µM#
0.01
0.1
1
10
100
Concentration [µM]

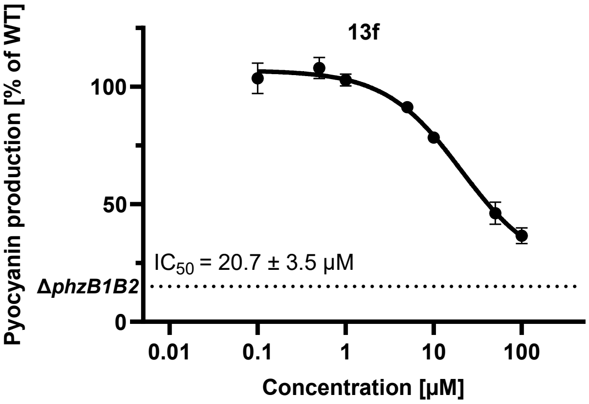
13f
Pyocyanin production [% of WT]
100
50
ΔphzB1B2
0
IC$_{50}$ = 20.7 ± 3.5 µM
0.01
0.1
1
10
100
Concentration [µM]

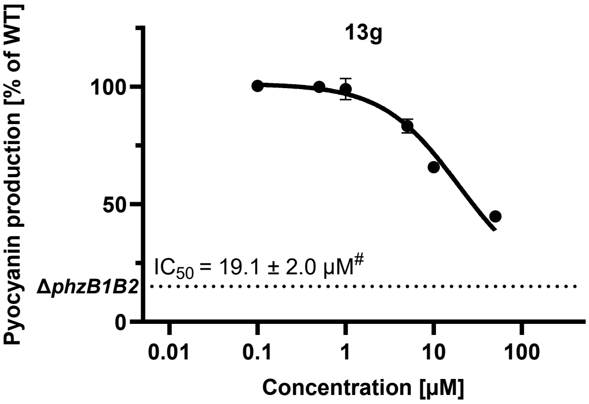
13g
Pyocyanin production [% of WT]
100
50
ΔphzB1B2
0
IC$_{50}$ = 19.1 ± 2.0 µM#
0.01
0.1
1
10
100
Concentration [µM]

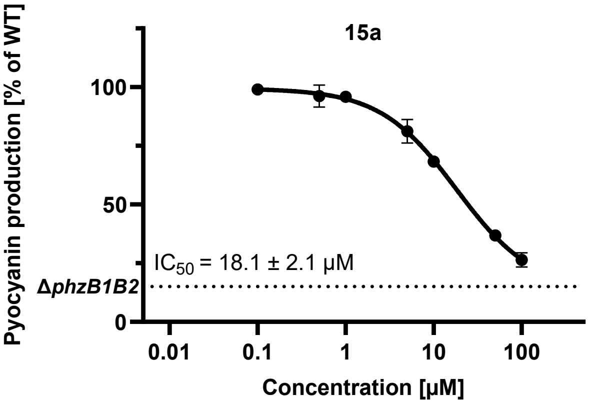
15a
Pyocyanin production [% of WT]
100
50
ΔphzB1B2
0
IC$_{50}$ = 18.1 ± 2.1 µM
0.01
0.1
1
10
100
Concentration [µM]

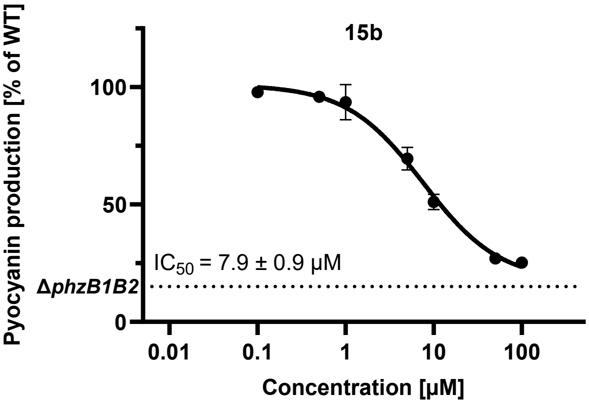
15b
Pyocyanin production [% of WT]
100
50
ΔphzB1B2
0
IC$_{50}$ = 7.9 ± 0.9 µM
0.01
0.1
1
10
100
Concentration [µM]

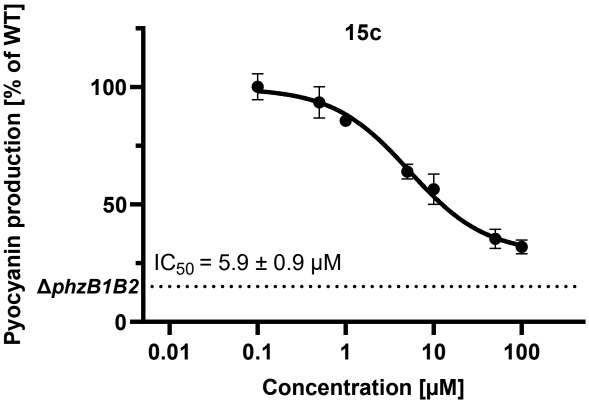
15c
Pyocyanin production [% of WT]
100
50
ΔphzB1B2
0
IC$_{50}$ = 5.9 ± 0.9 µM
0.01
0.1
1
10
100
Concentration [µM]

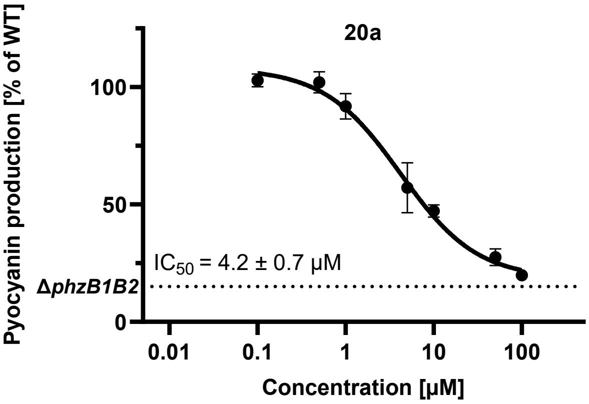
20a
Pyocyanin production [% of WT]
100
50
ΔphzB1B2
0
IC$_{50}$ = 4.2 ± 0.7 µM
0.01
0.1
1
10
100
Concentration [µM]

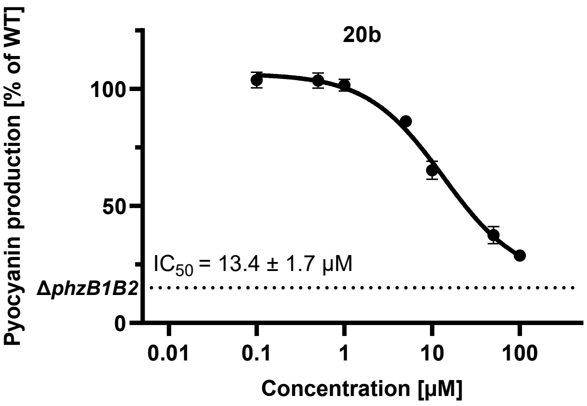
20b
Pyocyanin production [% of WT]
100
50
0
ΔphzB1B2
IC50 = 13.4 ± 1.7 µM
0.01
0.1
1
10
100
Concentration [µM]

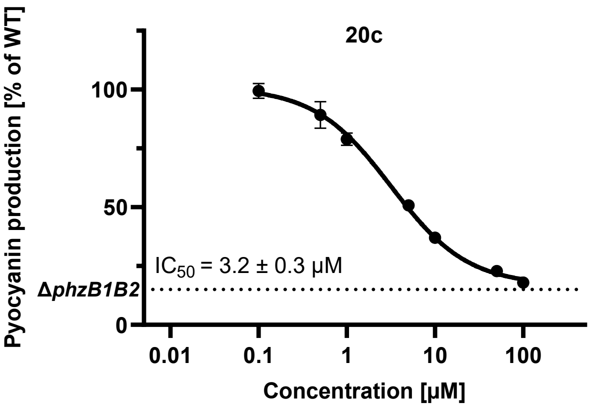
20c
Pyocyanin production [% of WT]
100
50
0
ΔphzB1B2
IC50 = 3.2 ± 0.3 µM
0.01
0.1
1
10
100
Concentration [µM]

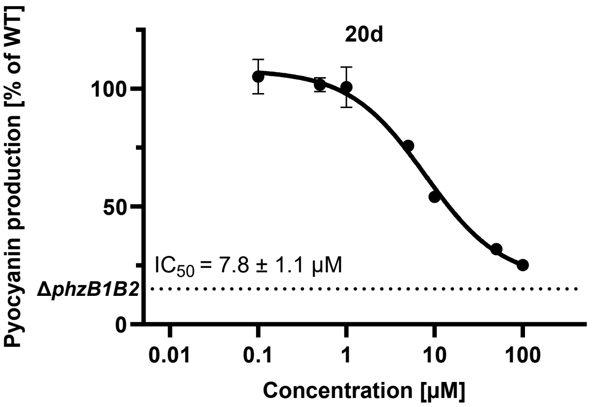
20d
Pyocyanin production [% of WT]
100
50
0
ΔphzB1B2
IC50 = 7.8 ± 1.1 µM
0.01
0.1
1
10
100
Concentration [µM]

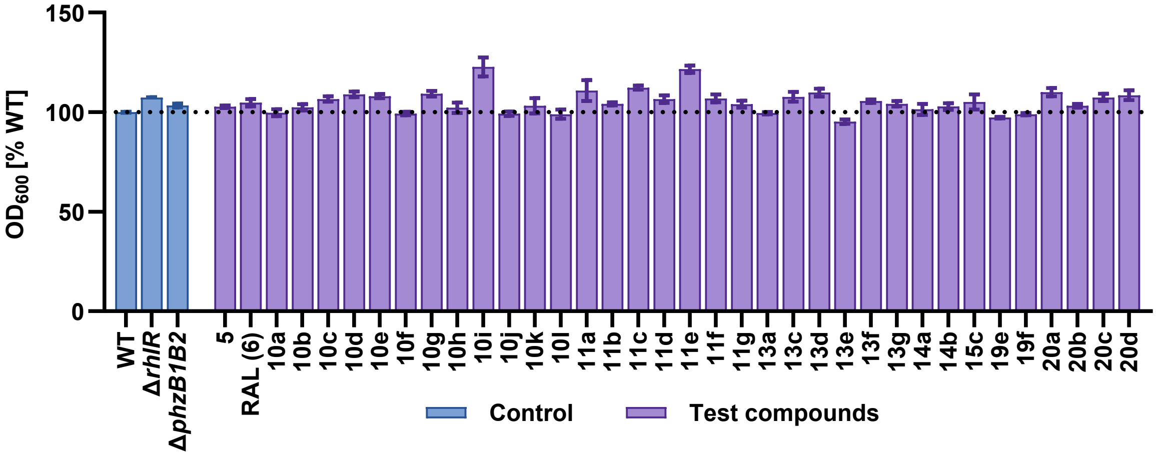

Figure S17: Cell density (OD_{600}) of *P. aeruginosa* PA14 wild type (WT) cell cultures after 16 h of aerobic incubation with test compounds (200 µM; **19f**: 100 µM, 2% v/v DMSO, 37 °C, 225 rpm) compared to OD_{600} of WT treated with DMSO and *P. aeruginosa* PA14 *rhlR*/*phzB1B2*-knockout mutants (Δ*rhlR*/Δphz*B1B2*) incubated under the same conditions. Reported is the mean ± SEM (n ≥ 3). The results show that the compounds do not exhibit direct antibacterial activity.

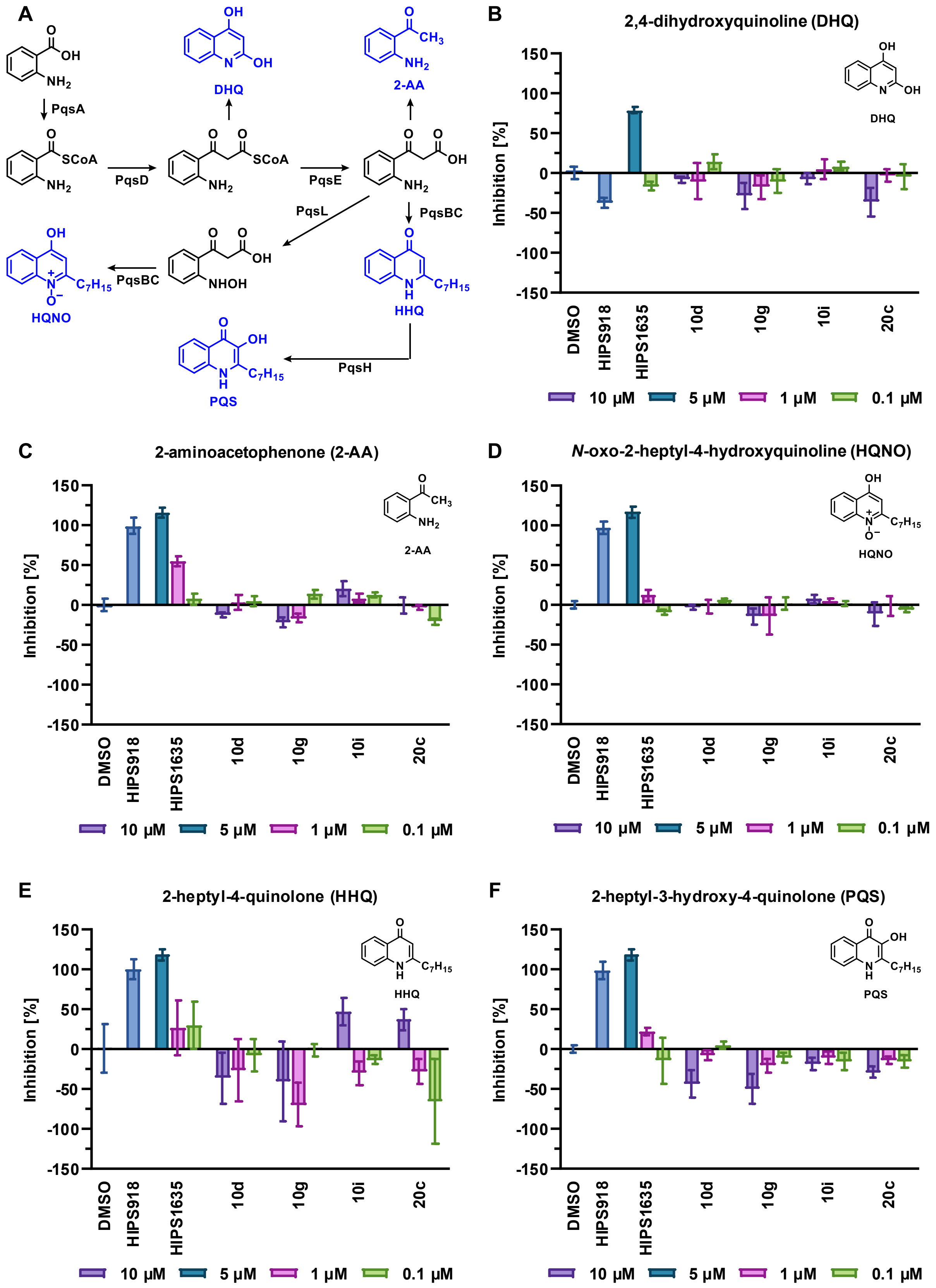

Figure S18: Results of LC-MS/MS-based alkylquinolone (AQ) quantification assay. **A.** Brief overview of AQ biosynthesis with assayed AQs highlighted in blue. Figure adapted from Schütz *et al.*[1] **B – F.** Effect of **10d**, **10g**, **10i** and **20c** on biosynthesis of AQs DHQ (**B**), 2-AA (**C**), HQNO (**D**), HHQ (**E**) and PQS (**F**). PqsR inhibitors HIPS918 (compound **M64** in Starkey *et al.*[2]) and HIPS1635 (compound **24** in Hamed *et al.*[3]) were used as controls. Reported is the mean ± SEM (n = 3).

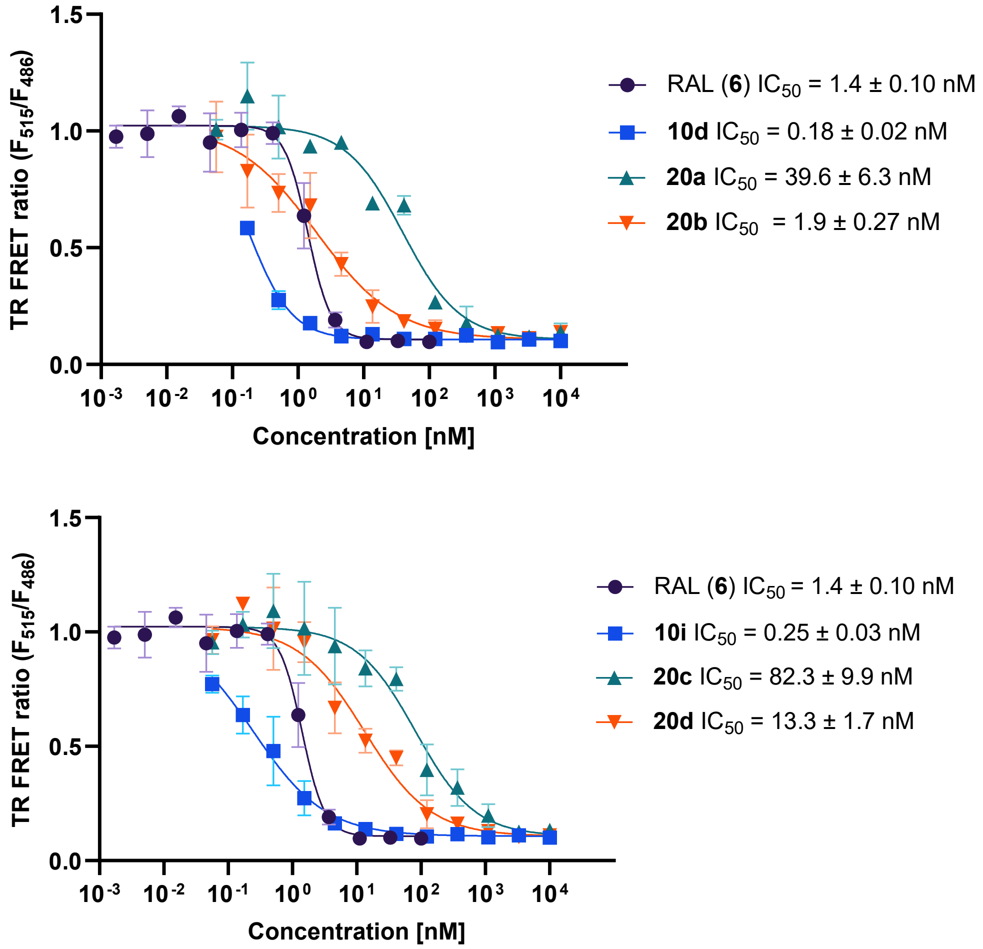

Figure S19: Human estrogen receptor-α (hER-α) binding dose-response curves of IC_{50} determination for compounds RAL (**6**), **10d/i** and **20a – d.** IC_{50} values were calculated using GraphPad Prism 10.1.2 with a four-parameter global nonlinear regression model (shared top and bottom values for all data sets) and are reported ± SEM (n = 4, except **20a**: n = 2).

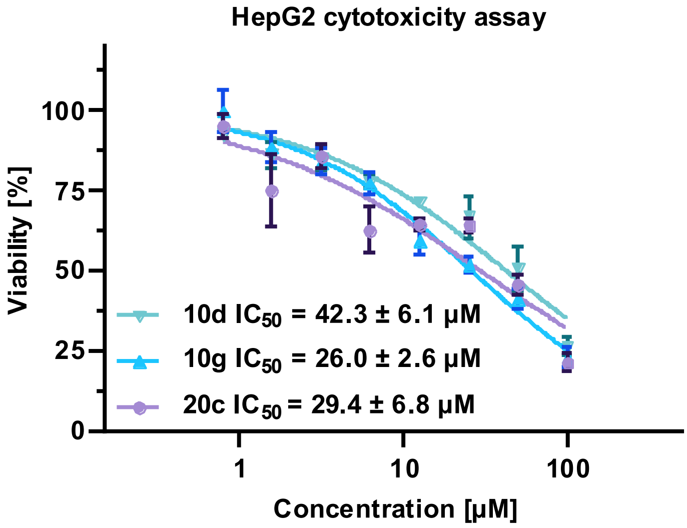

Figure S20: Dose-response curves from MTT viability assay with HepG2 cells. IC_{50} values were calculated using GraphPad Prism 10.1.2 with a four-parameter nonlinear regression model and are reported ± SEM (n = 2).

Supplementary Tables

Table S1: Previously reported inhibitors of the phenazine biosynthesis. n.d. = not determined.

Structure	Target	K_d [µM]	PYO reduction (*P. aeruginosa*)	PDB accession code	Reference
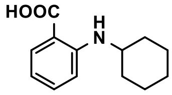	*Bc*PhzB	4.0 ± 0.6	n.d.	-	Ahuja *et al.*[4]
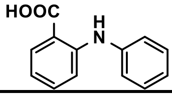	*Bc*PhzB	12 ± 1	n.d.	-	Ahuja *et al.*[4]
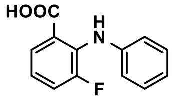	*Bc*PhzB	22 ± 3	n.d.	-	Ahuja *et al.*[4]
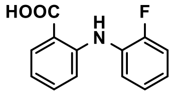	*Bc*PhzB	3.5 ± 0.4	n.d.	-	Ahuja *et al.*[4]
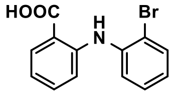	*Bc*PhzB	22 ± 2	n.d.	-	Ahuja *et al.*[4]
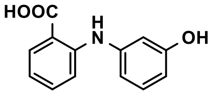	*Bc*PhzB	25 ± 3	n.d.	-	Ahuja *et al.*[4]
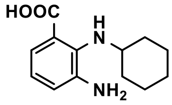	*Bc*PhzB	2.2 ± 0.2	n.d.	-	Ahuja *et al.*[4]
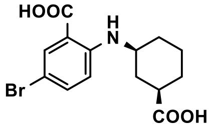<br>5	*Bc*PhzB	0.05[5]<br>0.1 ± 0.01<br>(this study)	n.d.	3JUM[6]	Mentel *et al.*[7]
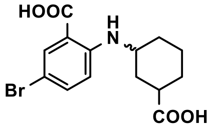	*Bc*PhzB	12.4 ± 0.82	n.d.	3JUN[8]/3JUQ[9]	Mentel *et al.*[7]
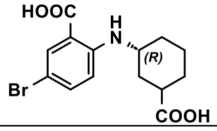	*Bc*PhzB	8.55 ± 2.6	n.d.	3JUO[10]	Mentel *et al.*[7]
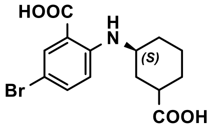	*Bc*PhzB	2.63 ± 1.1	n.d.	3JUP[11]	Mentel *et al.*[7]
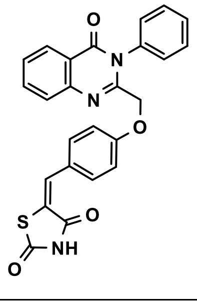	*Pa*PhzS	18 ± 0.89	>50%<br>@ 100 µM	-	Froes *et al.*[12]

Structure	Target	K_d [µM]	PYO reduction (*P. aeruginosa*)	PDB accession code	Reference
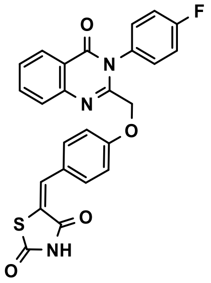	*Pa*PhzS	75 ± 0.73	>50% @ 100 µM	-	Froes *et al.*[12]

Table S2: Biological data for compounds **9a – l**, **10a – l**, **11a – g**, **12a – d**, **13a – g**, **14a/b**, **15a – c**, **19e/f** and **20a – d**. [#] Since the maximal inhibitory effect could not be determined, the regression was performed using the pyocyanin production of the *phzB1B2*-knockout mutant as bottom constraint and an approx. IC_{50} value ± SEM is reported. n.d. = not determined.

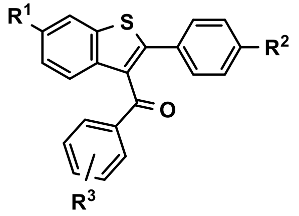

Cmpd	R[1]	R[2]	R[3]	*n*DSF ΔT_m (*Bc*PhzB) [K]	K_d (*Bc*PhzB) [µM]	Remaining PYO production @ 10 µM [%]	PYO IC_{50} [µM]	hER binding IC_{50} [nM]	S_{kin} [µM]	PDB accession code
**5**	-	-	-	27.5 ± 0.2	0.10 ± 0.01	98.4 ± 1.2	n.d.	n.d.	n.d.	3JUM[6]
**6** (RAL)	OH	OH	4-(2-(piperidin-1-yl)ethoxy)phenyl	10.1 ± 0.4	n.d.	93.3 ± 1.9	55.6 ± 6.0[#]	1.44 ± 0.10	7.5 ± 0.4	9F8H
**9a**	OMe	OMe	phenyl	1.6 ± 0.2	n.d.	90.8 ± 10.6	n.d.	n.d.	12.1 ± 2.6	n.d.
**9b**	OMe	OMe	4-methylphenyl	1.3 ± 0.3	n.d.	106.5 ± 8.8	n.d.	n.d.	n.d.	n.d.
**9c**	OMe	OMe	4-fluorophenyl	1.1 ± 0.3	n.d.	102.03 ± 13.6	n.d.	n.d.	n.d.	n.d.
**9d**	OMe	OMe	4-chlorophenyl	0.5 ± 0.6	n.d.	99.6 ± 3.8	n.d.	n.d.	n.d.	n.d.
**9e**	OMe	OMe	4-bromophenyl	0.9 ± 0.1	n.d.	106.0 ± 14.1	n.d.	n.d.	n.d.	n.d.
**9f**	OMe	OMe	3-methylphenyl	1.0 ± 0.3	n.d.	97.7 ± 5.7	n.d.	n.d.	n.d.	n.d.
**9h**	OMe	OMe	3-fluorophenyl	1.4 ± 0.7	n.d.	102.2 ± 5.2	n.d.	n.d.	n.d.	n.d.
**9i**	OMe	OMe	3-bromophenyl	1.1 ± 0.4	n.d.	95.9 ± 7.3	n.d.	n.d.	n.d.	n.d.
**9j**	OMe	OMe	3-methoxyphenyl	0.8 ± 0.2	n.d.	n.d.	n.d.	n.d.	n.d.	n.d.
**9k**	OMe	OMe	2-methylphenyl	1.1 ± 0.1	n.d.	103.1 ± 1.8	n.d.	n.d.	n.d.	n.d.
**9l**	OMe	OMe	2-fluorophenyl	0.6 ± 0.1	n.d.	105.4 ± 0.1	n.d.	n.d.	n.d.	n.d.
**10a**	OH	OH	phenyl	5.9 ± 0.2	1.71 ± 0.16	72.3 ± 2.4	15.6 ± 4.0	n.d.	15.0 ± 1.6	n.d.
**10b**	OH	OH	4-methylphenyl	8.7 ± 0.2	1.02 ± 0.09	49.1 ± 2.7	5.4 ± 1.4	n.d.	n.d.	n.d.
**10c**	OH	OH	4-fluorophenyl	5.7 ± 0.6	1.56 ± 0.12	59.2 ± 2.1	10.0 ± 1.6	n.d.	n.d.	n.d.
**10d**	OH	OH	4-chlorophenyl	6.8 ± 0.6	1.64 ± 0.07	39.6 ± 3.7	1.7 ± 0.4	0.18 ± 0.02	13.0 ± 1.1	9F8I
**10e**	OH	OH	4-bromophenyl	7.1 ± 0.3	n.d.	46.9 ± 9.1	2.6 ± 0.4	n.d.	n.d.	n.d.
**10f**	OH	OH	3-methylphenyl	5.3 ± 0.2	n.d.	57.0 ± 11.5	7.2 ± 1.3	n.d.	n.d.	n.d.
**10g**	OH	OH	3-isopropylphenyl	2.2 ± 0.4	n.d.	28.1 ± 6.3	0.9 ± 0.1	n.d.	7.7 ± 0.9	9F8K
**10h**	OH	OH	3-fluorophenyl	5.0 ±1.1	2.55 ± 0.16	55.2 ± 2.3	28.3 ± 3.5[#]	n.d.	14.6 ± 0.9	n.d.
**10i**	OH	OH	3-bromophenyl	6.7 ± 0.2	n.d.	33.3 ± 2.5	2.6 ± 0.7	0.25 ± 0.03	7.3 ± 0.2	9F8J
**10j**	OH	OH	3-hydroxyphenyl	7.7 ± 0.6	1.34 ± 0.06	76.7 ± 4.0	127 ± 13[#]	n.d.	70.7 ± 4.2	9F8L

Cmpd	R[1]	R[2]	R[3]	*n*DSF ΔT_m (*Bc*PhzB) [K]	K_d (*Bc*PhzB) [µM]	Remaining PYO production @ 10 µM [%]	PYO IC_{50} [µM]	hER binding IC_{50} [nM]	S_{kin} [µM]	PDB accession code
**10k**	OH	OH	2-methylphenyl	2.1 ± 1.1	n.d.	74.0 ± 11.3	29.9 ± 5.9	n.d.	n.d.	n.d.
**10l**	OH	OH	2-fluorophenyl	2.3 ± 0.5	3.84 ± 0.47	71.4 ± 1.6	30.7 ± 6.1	n.d.	18.7 ± 1.4	n.d.
**11a**	OH	OH	4-(propylamino)phenyl	12.1 ± 1.0	n.d.	56.6 ± 7.0	3.1 ± 0.7	n.d.	7.0 ± 0.8	9F8M
**11b**	OH	OH	4-(isobutylamino)phenyl	11.0 ± 0.6	n.d.	49.1 ± 4.9	3.9 ± 0.6	n.d.	8.5 ± 2.5	n.d.
**11c**	OH	OH	4-(pentylamino)phenyl	7.6 ± 0.3	n.d.	51.9 ± 5.2	3.0 ± 0.5	n.d.	5.5 ± 2.1	n.d.
**11d**	OH	OH	2-(propylamino)phenyl	3.3 ± 2.2	n.d.	36.1 ± 5.1	6.1 ± 0.6[#]	n.d.	5.8 ± 1.0	n.d.
**11e**	OH	OH	2-(isobutylamino)phenyl	4.4 ± 1.0	n.d.	46.4 ± 6.1	14.1 ± 2.9[#]	n.d.	3.9 ± 0.8	9F8N
**11f**	OH	OH	2-(pentylamino)phenyl	1.8 ± 0.1	n.d.	53.0 ± 11.0	6.9 ± 1.2	n.d.	3.6 ± 0.8	n.d.
**11g**	OH	OH	2-((2-morpholino ethyl)amino-phenyl	3.4 ± 1.2	n.d.	94.7 ± 5.0	122 ± 10[#]	n.d.	4.5 ± 0.5	9F8O
**12a**	OMe	OMe	4-(cyclopentoxy)phenyl	0.7 ± 0.2	n.d.	90.4 ± 5.9	n.d.	n.d.	n.d.	n.d.
**12b**	OMe	OMe	4-(isobutoxy)phenyl	0.7 ± 0.1	n.d.	92.6 ± 3.2	n.d.	n.d.	n.d.	n.d.
**12c**	OMe	OMe	4-(pentoxy)phenyl	1.1 ± 0.3	n.d.	89.0 ± 6.3	n.d.	n.d.	n.d.	n.d.
**12d**	OMe	OMe	2-(isobutoxy)phenyl	-0.4 ± 0.9	n.d.	89.5 ± 5.3	n.d.	n.d.	n.d.	n.d.
**13a**	OH	OH	4-hydroxyphenyl	6.7 ± 1.4	2.17 ± 0.06	94.8 ± 4.1	21.7 ± 3.7[#]	n.d.	104.5 ± 7.7	9F8P
**13b**	OH	OH	4-(isobutyloxy)phenyl	6.1 ±1.1	n.d.	86.3 ± 9.4	n.d.	n.d.	n.d.	n.d.
**13c**	OH	OH	4-(pentoxy)phenyl	14.0 ± 0.2	n.d.	40.0 ± 4.2	2.8 ± 0.7	n.d.	8.3 ± 1.8	n.d.
**13d**	OH	OH	2-hydroxyphenyl	n.d.	0.92 ± 0.14	62.0 ± 2.1	30.6 ± 5.1[#]	n.d.	12.9 ± 0.2	9F8Q
**13e**	OH	OH	2-methoxyphenyl	-0.9 ± 0.9	n.d.	84.1 ± 2.2	54.6 ± 4.5[#]	n.d.	22.1 ± 1.1	n.d.
**13f**	OH	OH	2-(isobutoxy)phenyl	0.8 ± 0.9	n.d.	69.6 ± 6.1	20.7 ± 3.5	n.d.	8.0 ± 0.7	n.d.
**13g**	OH	OH	2-(pentoxy)phenyl	0.8 ± 0.5	n.d.	54.9 ± 5.4	19.1 ± 2.0[#]	n.d.	5.9 ± 1.1	n.d.
**15a**	OH	OH	cyclopentyl	2.0 ± 0.2	n.d.	84.4 ± 5.7	18.1 ± 2.1	n.d.	18.1 ± 3.7	n.d.
**15b**	OH	OH	cyclohexyl	1.0 ± 0.2	n.d.	58.1 ± 7.8	7.6 ± 0.9	n.d.	18.4 ± 0.8	n.d.
**15c**	OH	OH	Pentyl	3.7 ± 0.7	n.d.	48.1 ± 8.1#	5.3 ± 0.9	n.d.	18.6 ± 0.5	n.d.
**19e**	H	H	4-chlorophenyl	0.9 ± 0.3	n.d.	105.1 ± 1.0	n.d.	n.d.	n.d.	n.d.
**19f**	H	H	3-bromophenyl	0.9 ± 0.1	n.d.	88.2 ± 10.1	n.d.	n.d.	n.d.	n.d.
**20a**	H	OH	4-chlorophenyl	5.3 ± 0.0	n.d.	52.3 ± 10.3	4.2 ± 0.7	39.6 ± 6.31	n.d.	9F8R
**20b**	OH	H	4-chlorophenyl	5.0 ± 0.2	n.d.	65.2 ± 3.9#	13.4 ± 1.7	1.9 ± 0.27	n.d.	9F8S
**20c**	H	OH	3-bromophenyl	5.5 ± 0.2	n.d.	37.0 ± 1.9#	3.2 ± 0.3	82.3 ± 9.87	5.5 ± 0.4	n.d.
**20d**	OH	H	3-bromophenyl	5.4 ± 0.5	n.d.	54.1 ± 0.6#	7.8 ± 1.1	13.3 ± 1.69	6.1 ± 1.6	n.d.

Table S3: Calculation of physicochemical properties of potential PhzB inhibitors. According to the so-called eNTRy rules for porin-dependent uptake in Gram-negative bacteria, compounds should feature a globularity of ≤ 0.25 and a number of rotatable bonds ≤ 5.[13] According to the *P. aeruginosa* permeation guidelines, also referred to as PASsagE (*Pseudomonas aeruginosa* Self-promoted Entry) rules, small molecules should have a hydrogen bond donor (HBD) surface area of ≥ 23 Å², a positive polar surface area of ≥ 80 Å² and/or a formal charge of ≥ 0.98.[14] n.a. = not applicable.

Cmpd	PYO IC_{50} [µM]	Globularity (glob)	# rotatable bonds (RotB)	HBD surface area [Å²] (vsa_don)	Polar surface area [Å²] (vsa_pol)	Positive polar surface area [Å²] (Q_vsa_Ppos)	Formal charge (Fcharge)
**5**	n.a.	0.2	4	5.7	60.0	61.8	-2
**6**	55.9	0.2	7	0.0	43.2	29.1	1
**9a**	n.a.	0.3	5	0.0	18.6	8.5	0
**9b**	n.a.	0.4	5	0.0	18.6	8.5	0
**9c**	n.a.	0.4	5	0.0	18.6	8.5	0
**9d**	n.a.	0.4	5	0.0	18.6	8.5	0
**9e**	n.a.	0.4	5	0.0	18.6	8.5	0
**9f**	n.a.	0.3	5	0.0	18.6	8.5	0
**9g**	n.a.	0.4	6	0.0	18.6	8.5	0
**9h**	n.a.	0.3	5	0.0	18.6	8.5	0
**9i**	n.a.	0.4	5	0.0	18.6	8.5	0
**9j**	n.a.	0.4	6	0.0	21.1	8.5	0
**9k**	n.a.	0.3	5	0.0	18.6	8.5	0
**9l**	n.a.	0.3	5	0.0	18.6	8.5	0
**9a**	n.a.	0.3	5	0.0	18.6	8.5	0
**9b**	n.a.	0.4	5	0.0	18.6	8.5	0
**9c**	n.a.	0.4	5	0.0	18.6	8.5	0
**9d**	n.a.	0.4	5	0.0	18.6	8.5	0
**9e**	n.a.	0.4	5	0.0	18.6	8.5	0
**9f**	n.a.	0.3	5	0.0	18.6	8.5	0
**9g**	n.a.	0.4	6	0.0	18.6	8.5	0
**9h**	n.a.	0.3	5	0.0	18.6	8.5	0
**9i**	n.a.	0.4	5	0.0	18.6	8.5	0
**9j**	n.a.	0.4	6	0.0	21.1	8.5	0
**9k**	n.a.	0.3	5	0.0	18.6	8.5	0
**9l**	n.a.	0.3	5	0.0	18.6	8.5	0
**10a**	15.6	0.4	3	0.0	40.7	29.1	0
**10b**	5.4	0.4	3	0.0	40.7	29.1	0
**10c**	10	0.4	3	0.0	40.7	29.1	0
**10d**	1.7	0.4	3	0.0	40.7	29.1	0
**10e**	2.6	0.3	3	0.0	40.7	29.1	0
**10f**	7.2	0.4	3	0.0	40.7	29.1	0
**10g**	0.9	0.4	4	0.0	40.7	29.1	0
**10h**	28.3	0.4	3	0.0	40.7	29.1	0
**10i**	2.6	0.4	3	0.0	40.7	29.1	0
**10j**	127	0.4	3	0.0	54.3	39.4	0

Cmpd	PYO IC_{50} [µM]	Globularity (glob)	# rotatable bonds (RotB)	HBD surface area [Å²] (vsa_don)	Polar surface area [Å²] (vsa_pol)	Positive polar surface area [Å²] (Q_vsa_Ppos)	Formal charge (Fcharge)
**10k**	29.9	0.3	5	0.0	18.6	8.5	0
**10l**	30.7	0.4	3	0.0	40.7	29.1	0
**11a**	3.1	0.3	6	5.7	46.4	50.9	0
**11b**	3.9	0.3	6	5.7	46.4	50.9	0
**11c**	3	0.3	8	5.7	46.4	50.9	0
**11d**	6.1	0.5	6	5.7	46.4	50.9	0
**11e**	14.1	0.5	6	5.7	46.4	50.9	0
**11f**	6.9	0.5	8	5.7	46.4	50.9	0
**11g**	122	0.4	7	5.7	48.9	50.9	1
**12a**	n.a.	0.3	7	0.0	21.1	8.5	0
**12b**	n.a.	0.3	8	0.0	21.1	8.5	0
**12c**	n.a.	0.3	10	0.0	21.1	8.5	0
**12d**	n.a.	0.4	8	0.0	21.1	8.5	0
**13a**	21.7	0.4	3	0.0	54.3	39.4	0
**13b**	2.8	0.3	6	0.0	43.2	29.1	0
**13c**	30.6	0.3	8	0.0	43.2	29.1	0
**13d**	54.6	0.4	3	0.0	54.3	39.4	0
**13e**	20.7	0.4	4	0.0	43.2	29.1	0
**13f**	19.1	0.5	6	0.0	43.2	29.1	0
**13g**	n.a.	0.5	8	0.0	43.2	29.1	0
**14a**	n.a.	0.3	5	0.0	18.6	8.5	0
**14b**	n.a.	0.3	5	0.0	18.6	8.5	0
**15a**	18.1	0.4	3	0.0	40.7	29.1	0
**15b**	7.6	0.4	3	0.0	40.7	29.1	0
**15c**	5.3	0.4	6	0.0	40.7	29.1	0
**19a**	n.a.	0.4	4	0.0	16.1	8.5	0
**19b**	n.a.	0.4	4	0.0	16.1	8.5	0
**19c**	n.a.	0.4	4	0.0	16.1	8.5	0
**19d**	n.a.	0.4	4	0.0	16.1	8.5	0
**19e**	n.a.	0.4	3	0.0	13.6	8.5	0
**19f**	n.a.	0.4	3	0.0	13.6	8.5	0
**20a**	4.2	0.4	3	0.0	27.1	18.8	0
**20b**	13.4	0.4	3	0.0	27.1	18.8	0
**20c**	3.2	0.4	3	0.0	27.1	18.8	0
**20d**	7.8	0.4	3	0.0	27.1	18.8	0

Table S4: Crystallization conditions for the obtained cocrystal structures of *Bc*PhzB in complex with the compounds listed below.

Cmpd	PDB accession code	Crystallization condition
RAL (**6**)	9F8H	17% (w/v) PEG 3350, 0.1 M Bis-TRIS pH 6.6, 0.2 M ammonium acetate
**10d**	9F8I	18.3% (w/v) PEG 3350, 0.1 M Bis-TRIS pH 6.56, 0.133 M lithium acetate
**10i**	9F8J	15% (w/v) PEG monomethyl ether, 0.1 M MES pH 6.14, 0.122 M ammonium acetate, 5% (v/v) glycerol
**10g**	9F8K	25% (w/v) PEG 3350, 0.1 M Bis-TRIS pH 6.37, 0.122 M ammonium acetate
**10j**	9F8L	15% (w/v) PEG monomethyl ether, 0.1 M MES pH 6.14, 0.122 M ammonium acetate, 5% (v/v) glycerol
**11a**	9F8M	28.3% (w/v) PEG monomethyl ether
**11e**	9F8N	16.7% (w/v) PEG 3350, 0.1 M MES pH 6.48, 6.67% (v/v) glycerol
**11g**	9F8O	18.3% (w/v) PEG 3350, 0.1 M Bis-TRIS pH 6.56, 0.133 M lithium acetate
**13a**	9F8P	18.3% (w/v) PEG 3350, 0.1 M MES pH 6.37, 0.278 M ammonium acetate, 6.67% (v/v) glycerol
**13d**	9F8Q	23.3% (w/v) PEG 4000, 0.1 M Bis-TRIS pH 6.93, 8.33% glycerol
**20a**	9F8R	15% (w/v) PEG monomethyl ether, 0.1 M MES pH 6.14, 0.122 M ammonium acetate, 5% (v/v) glycerol
**20b**	9F8S	25% (w/v) PEG 3350, 0.1M Bis-TRIS pH 5.99, 0.15 M lithium sulfate

Table S5: Protein crystallography data collection and refinement statistics. [a]Values for the highest resolution shell are shown in parentheses.

**Structure (PDB code)**	*Bc*PhzB in complex with **6** (9F8H)	*Bc*PhzB in complex with **10d** (9F8I)	*Bc*PhzB in complex with **10g** (9F8K)	*Bc*PhzB in complex with **10i** (9F8J)	*Bc*PhzB in complex with **10j** (9F8L)	*Bc*PhzB in complex with **11a** (9F8M)
**Data collection**						
Beamline	ESRF, ID23-1	DESY, Petra III, P11	DESY, Petra III, P11	DESY, Petra III, P11	DESY, Petra III, P11	DESY, Petra III, P11
Wavelength (Å)	0.972	1.033	1.033	1.033	1.033	1.033
Space group	$P12_11$	$P12_11$	C121	$P12_11$	C121	$P12_11$
Cell dimensions						
a, *b*, *c* (Å)	53.04, 69.10, 54.22	68.86, 68.18, 82.81	71.36, 79.30, 64.06	69.36, 69.09, 82.99	82.96, 69.37, 69.18	69.25, 68.39, 83.84
α, β, γ (°)	90, 101, 90	90, 90, 90	90, 98, 90	90, 90, 90	90, 94, 90	90, 90, 90
Resolution (Å)[a]	53.206 – 1.468 (1.493 – 1.468)	19.236 – 1.389 (1.413 – 1.389)	52.730 – 1.566 (1.593 – 1.566)	82.993 – 1.601 (1.629 – 1.601)	53.181 – 1.379 (1.403 – 1.379)	69.251 – 1.428 (1.453 – 1.428)
R_{merge} (%)[a]	6.5 (95.5)	6.0 (40.5)	5.3 (49.0)	8.0 (96.7)	4.7 (68.6)	4.6 (47.1)
R_{pim} (%)[a]	3.6 (53.2)	6.5 (43.7)	6.0 (28.7)	3.4 (41.9)	1.9 (27.8)	5.0 (51.6)
$I/\sigma I$[a]	11.1 (2.2)	14.3 (3.1)	12.8 (2.2)	11.2 (2.1)	16.5 (2.1)	14.2 (2.1)
Completeness (%)[a]	99.5 (99.6)	98.9 (97.8)	96.7 (78.0)	95.6 (88.5)	99.1 (99.1)	98.9 (92.4)
Redundancy[a]	4.0 (4.1)	7.0 (6.6)	4.4 (3.5)	6.4 (6.1)	6.9 (6.9)	6.7 (6.0)
$CC_{1/2}$ (%)[a]	99.8 (99.7)	99.9 (94.8)	99.8 (77.5)	99.8 (80.6)	99.9 (89.7)	99.9 (94.7)
**Refinement**						
Resolution (Å)	1.47	1.35	1.57	1.60	1.38	1.43
No. reflections	66389	152234	48009	98628	79659	142907
R_{work} / R_{free} (%)	13.4 / 16.4	12.9 / 15.5	15.3 / 17.5	16.4 / 18.7	13.7 / 16.4	15.7 / 18.7
No. atoms (non-H)	3145	6377	3231	6490	3381	6255
Protein	2723	5498	2799	5657	2933	5508
Ligand/ion	158	136	56	298	100	150
Water	264	743	376	535	348	597
B-factors ($Å^2$)	21.87	19.45	24.08	30.38	25.88	26.14
Protein	20.30	17.55	22.32	29.27	24.19	24.84
Ligand/ion	23.54	18.04	29.87	32.69	29.96	25.19
Water	37.03	33.71	36.25	40.81	38.95	38.33
R.m.s deviations						
Bond lengths (Å)	0.016	0.008	0.006	0.009	0.010	0.007
Bond angles (º)	1.323	0.919	0.945	1.066	1.172	0.901
Ramachandran statistics						
Favored	99.34	99.17	99.00	98.86	99.3	99.18
Allowed	0.66	0.83	1.00	0.98	0.70	0.92
Outliers	0.00	0.00	0.00	0.16	0.00	0.00
Clashscore (MolProbity)	1.17	0.90	2.35	1.52	1.10	0.79
MolProbity score	0.83	0.77	1.01	0.94	0.82	0.75

**Structure (PDB code)**	*Bc*PhzB in complex with **11e** (9F8N)	*Bc*PhzB in complex with **11g** (9F8O)	*Bc*PhzB in complex with **13a** (9F8P)	*Bc*PhzB in complex with **13d** (9F8Q)	*Bc*PhzB in complex with **20a** (9F8R)	*Bc*PhzB in complex with **20b** (9F8S)
**Data collection**						
Beamline	DESY, Petra III, P11	DESY, Petra III, P11	DESY, Petra III, P11	DESY, Petra III, P11	DESY, Petra III, P11	DESY, Petra III, P11
Wavelength (Å)	1.033	1.033	1.033	1.033	1.033	1.033
Space group	$P12_11$	$P12_11$	$P12_11$	$P12_11$	$P12_11$	$P12_11$
Cell dimensions						
a, b, c (Å)	54.02, 68.85, 54.17	68.83, 69.63, 83.14	54.06, 69.10, 54.14	53.97, 68.80, 54.06	69.93, 69.02, 83.35	54.02, 68.90, 54.08
α, β, γ (°)	90, 101, 90	90, 90, 90	90, 101, 90	90, 101, 90	90, 90, 90	90, 100, 90
Resolution (Å)[a]	42.034 – 1.316 (1.339 – 1.316)	68.830 – 1.567 (1.594 – 1.567)	53.122 – 1.363 (1.386 – 1.363)	53.061 – 1.336 (1.359 – 1.336)	69.933 – 1.580 (1.608 – 1.580)	53.146 – 1.686 (1.715 – 1.686)
R_{merge} (%)[a]	4.0 (52.8)	5.9 (63.4)	5.9 (70.6)	6.8 (50.8)	13.2 (127.1)	12.3 (101.8)
R_{pim} (%)[a]	1.7 (25.9)	2.4 (25.4)	2.4 (28.8)	2.7 (22.8)	14.2 (123.4)	4.9 (42.1)
$I/\sigma I$[a]	20.5 (2.2)	14.0 (2.4)	14.1 (2.0)	11.9 (2.2)	8.0 (2.3)	9.2 (2.1)
Completeness (%)[a]	98.4 (88.9)	99.0 (98.9)	98.9 (99.9)	99.0 (96.2)	98.9 (98.3)	98.2 (97.8)
Redundancy[a]	6.6 (5.0)	7.0 (7.0)	6.7 (6.7)	6.8 (5.6)	7.0 (7.1)	7.1 (6.7)
$CC_{1/2}$ (%)[a]	99.9 (89.7)	99.9 (93.8)	99.9 (88.2)	99.7 (91.0)	98.5 (87.5)	99.5 (88.5)
**Refinement**						
Resolution (Å)	1.32	1.57	1.36	1.34	1.58	1.69
No. reflections	90741	108813	82521	86925	107022	43215
R_{work} / R_{free} (%)	13.5 / 16.0	16.4 / 18.3	13.6 / 16.4	12.9 / 15.2	19.3 / 21.7	16.7 / 18.9
No. atoms (non-H)	3344	6392	3136	3435	6189	3064
Protein	2830	5678	2800	2965	5536	2739
Ligand/ion	132	188	80	88	164	68
Water	382	526	256	382	489	257
B-factors (Å^2)	27.22	27.86	25.56	22.61	22.98	22.96
Protein	25.22	27.10	24.24	20.49	21.91	21.83
Ligand/ion	31.69	24.78	28.34	25.33	25.86	22.53
Water	40.50	36.70	39.26	38.41	34.12	35.13
R.m.s deviations						
Bond lengths (Å)	0.010	0.008	0.010	0.009	0.010	0.005
Bond angles (°)	1.072	0.791	1.182	1.024	1.110	0.763
Ramachandran statistics						
Favored	99.30	99.30	99.34	99.3	99.34	99.34
Allowed	0.70	0.70	0.66	0.70	0.66	0.66
Outliers	0.00	0.00	0.00	0.00	0.00	0.00
Clashscore (MolProbity)	1.13	1.52	1.55	0.55	0.98	0.80
MolProbity score	0.87	0.89	0.90	0.72	0.85	0.75

Supplementary NMR Spectra of test compounds

^{1}H NMR (500 MHz, C_2D_6OS) for compound **9f**

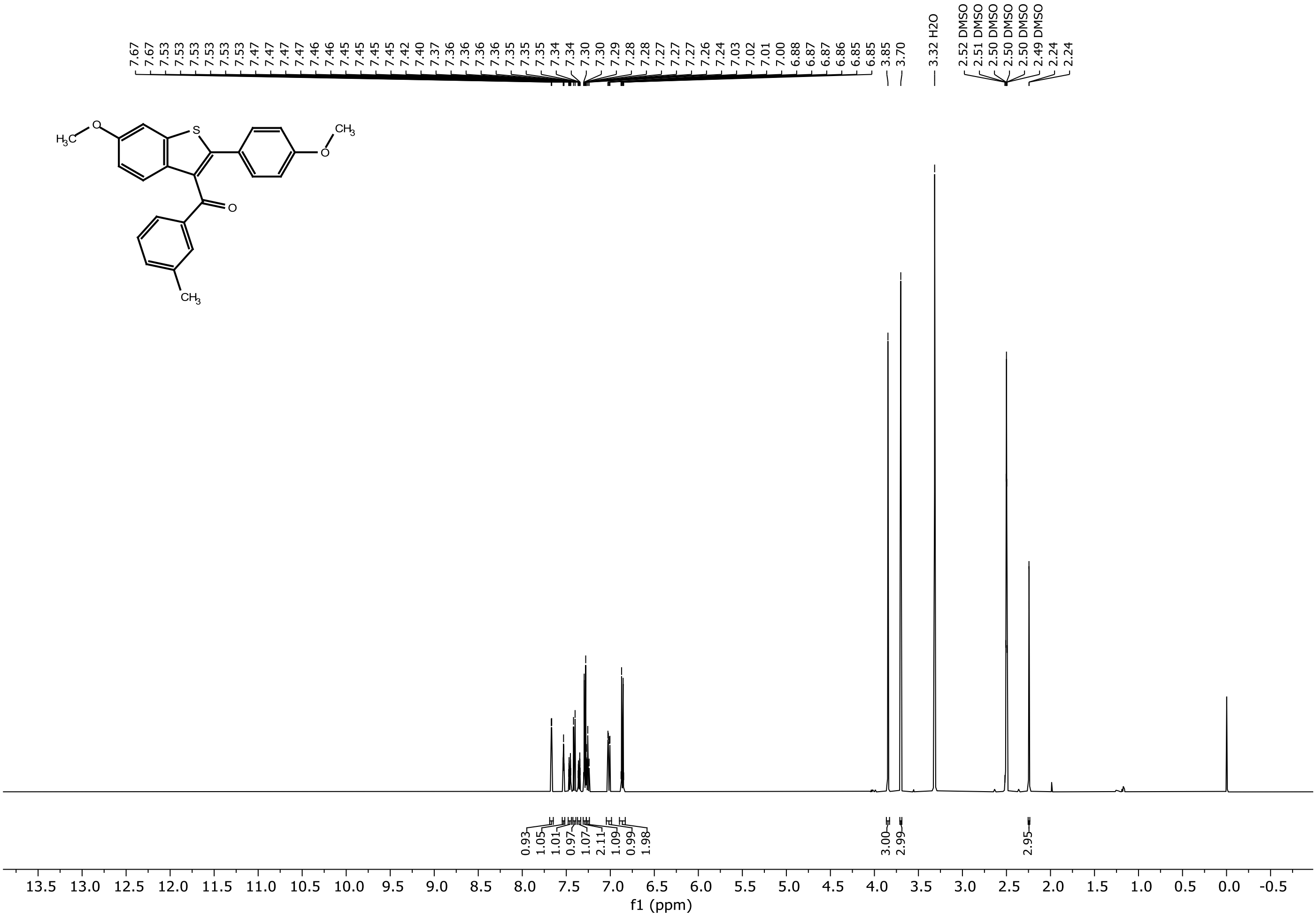

^{13}C NMR (126 MHz, C_2D_6OS) for compound **9f**

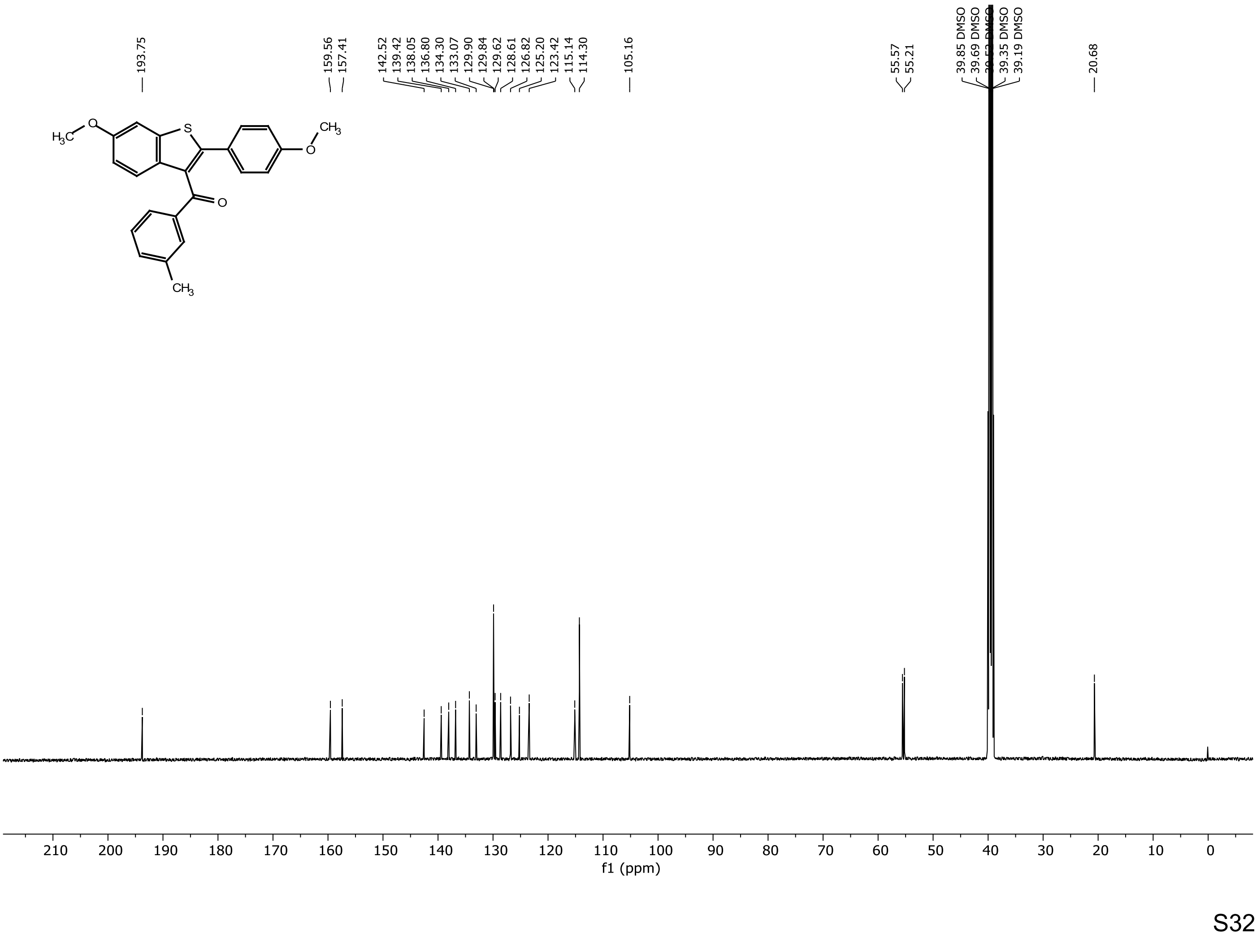

^{1}H NMR (400 MHz, C_2D_6OS) for compound **9h**

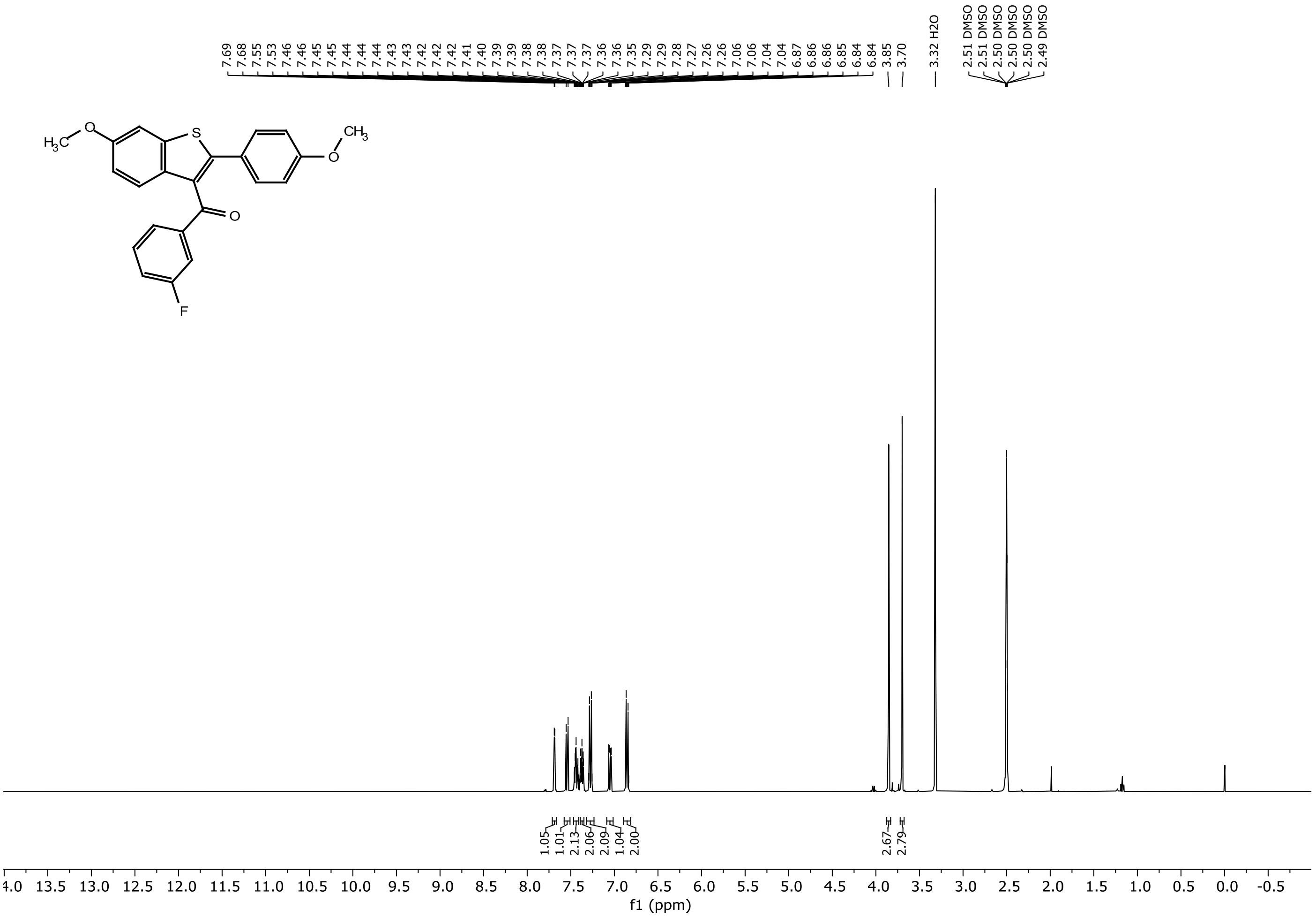

^{13}C NMR (101 MHz, C_2D_6OS) for compound **9h**

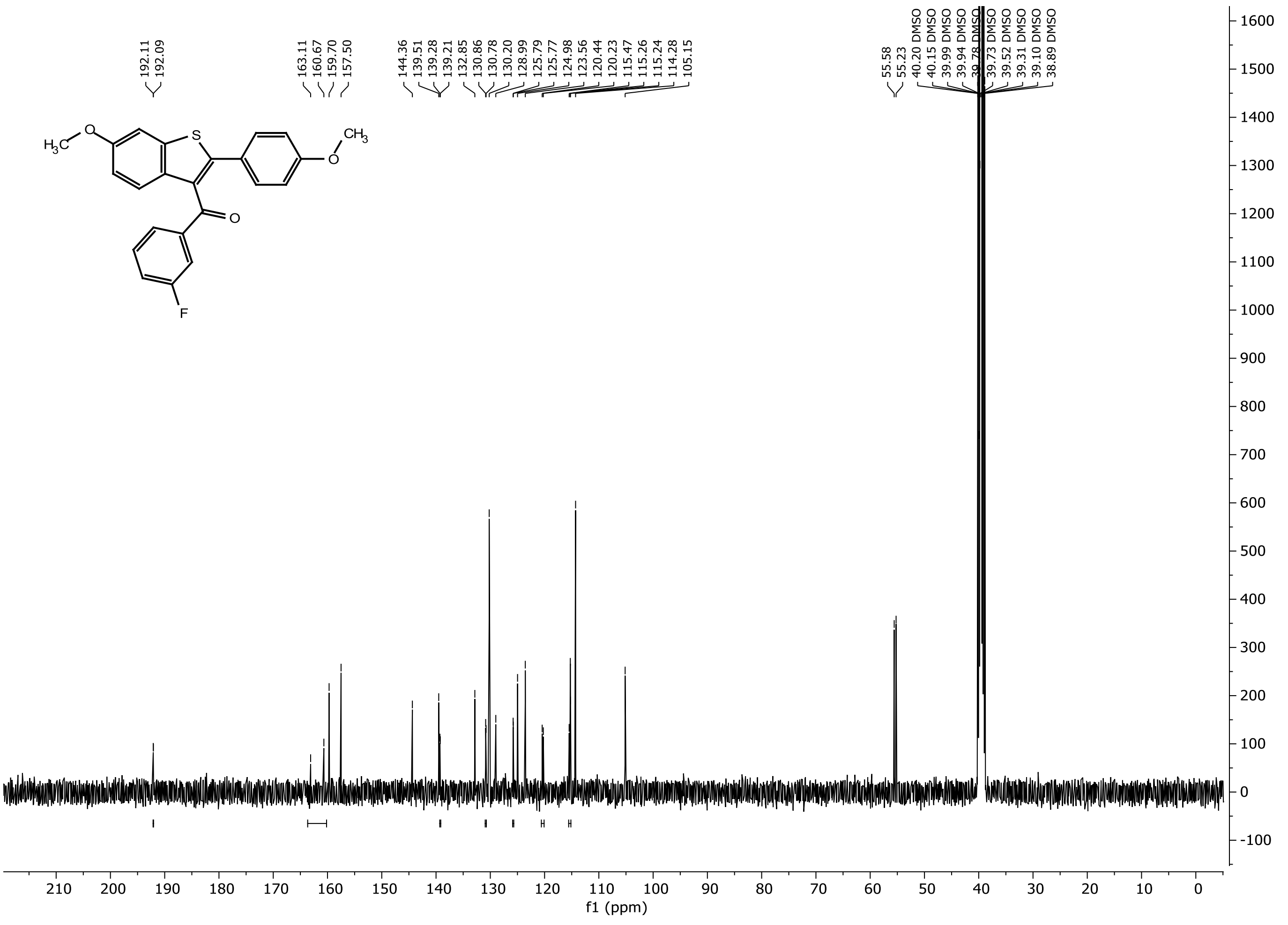

^{1}H NMR (400 MHz, C_2D_6OS) for compound **9i**

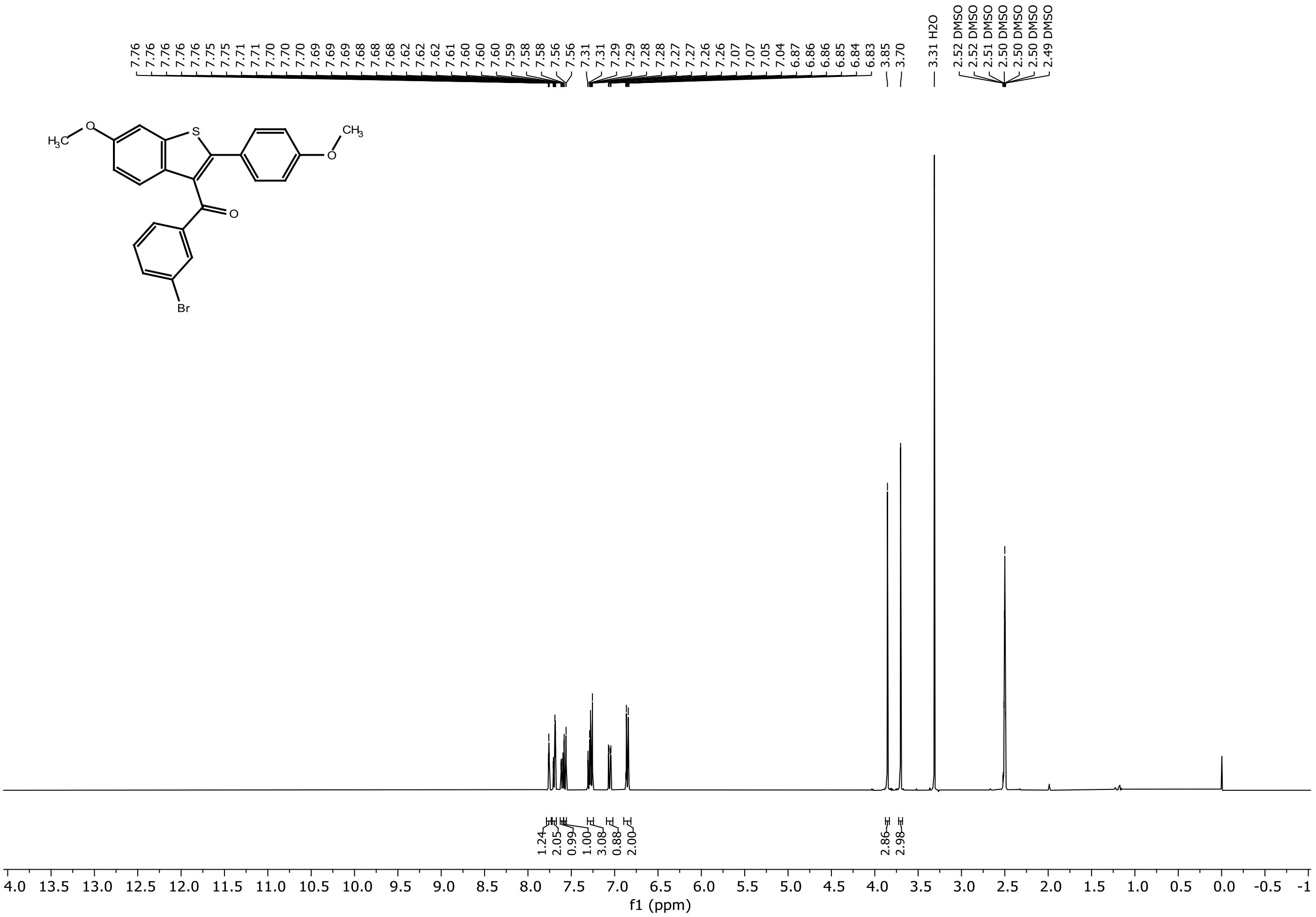

^{13}C NMR (101 MHz, C_2D_6OS) for compound **9i**

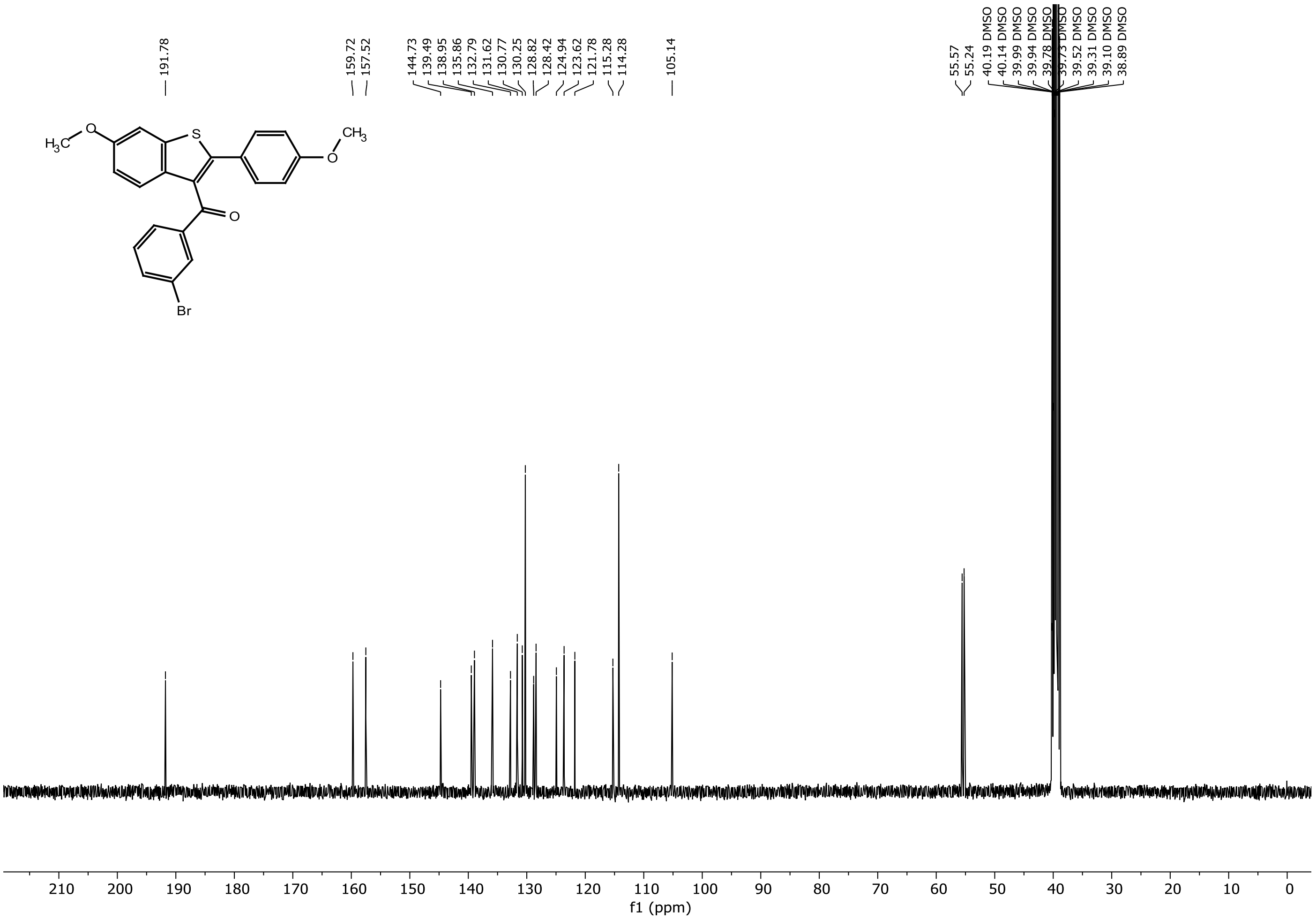

^1H NMR (400 MHz, C_2D_6OS) for compound **9j**

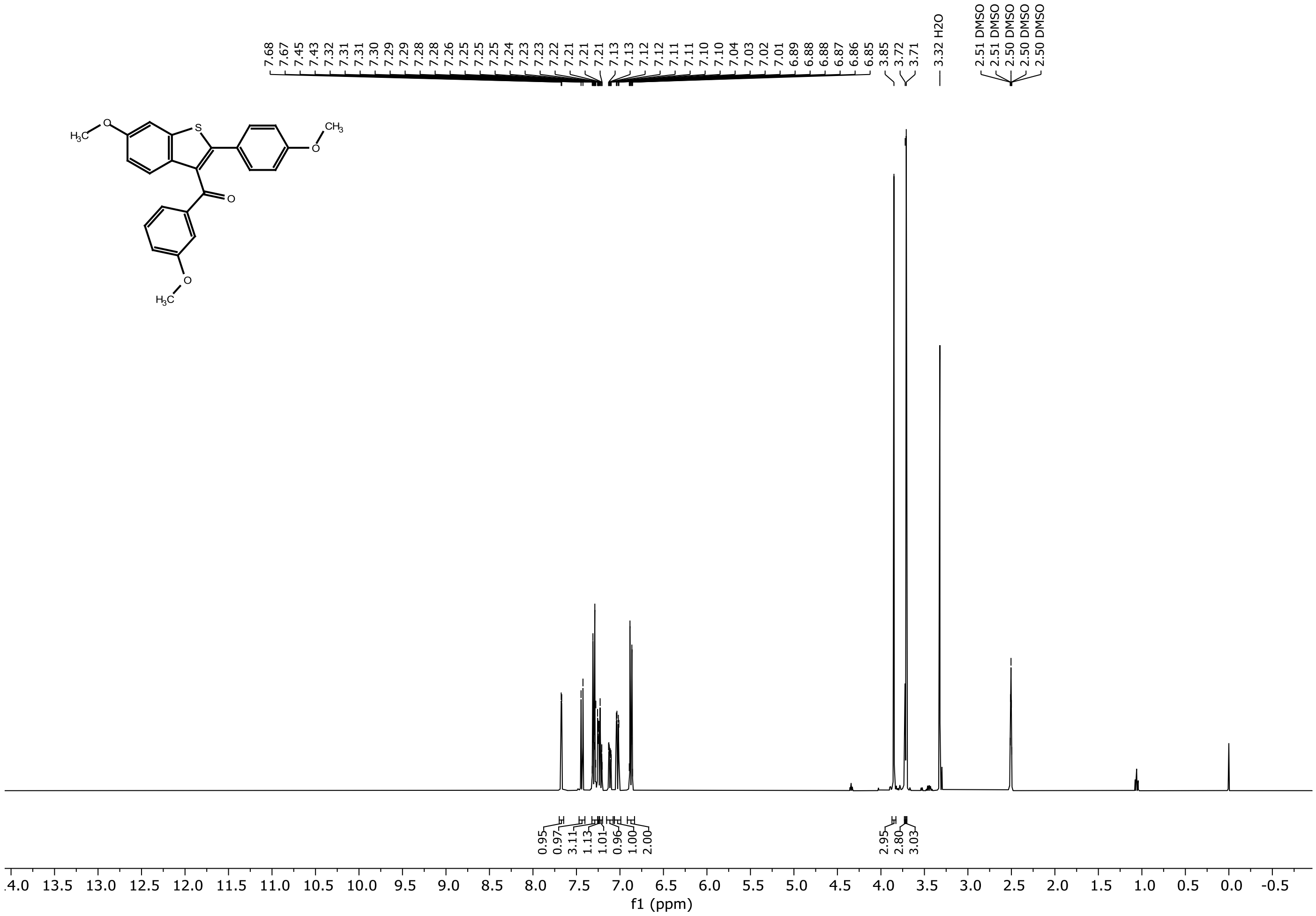

^{13}C NMR (101 MHz, C_2D_6OS) for compound **9j**

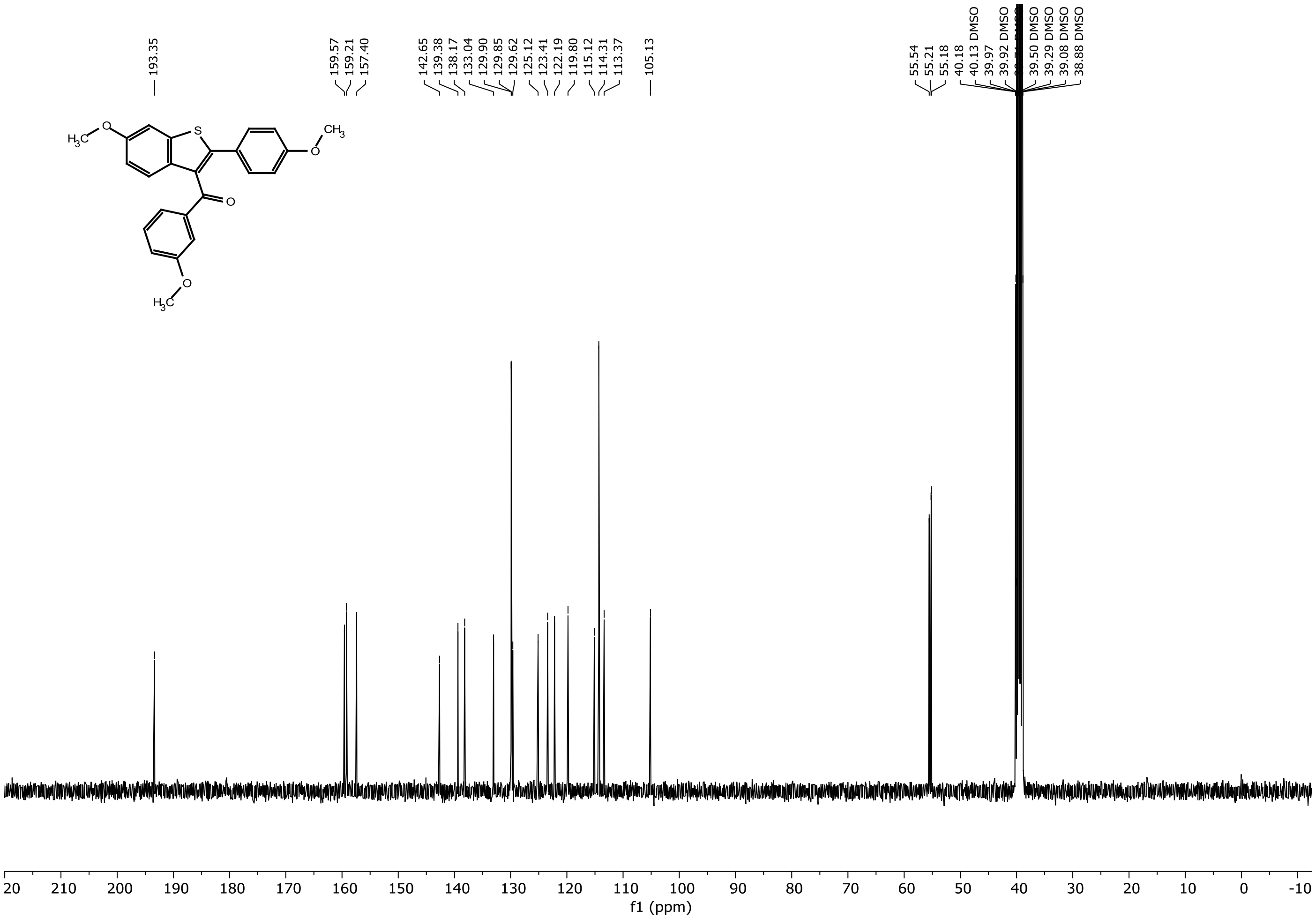

^{1}H NMR (400 MHz, C_2D_6OS) for compound **9k**

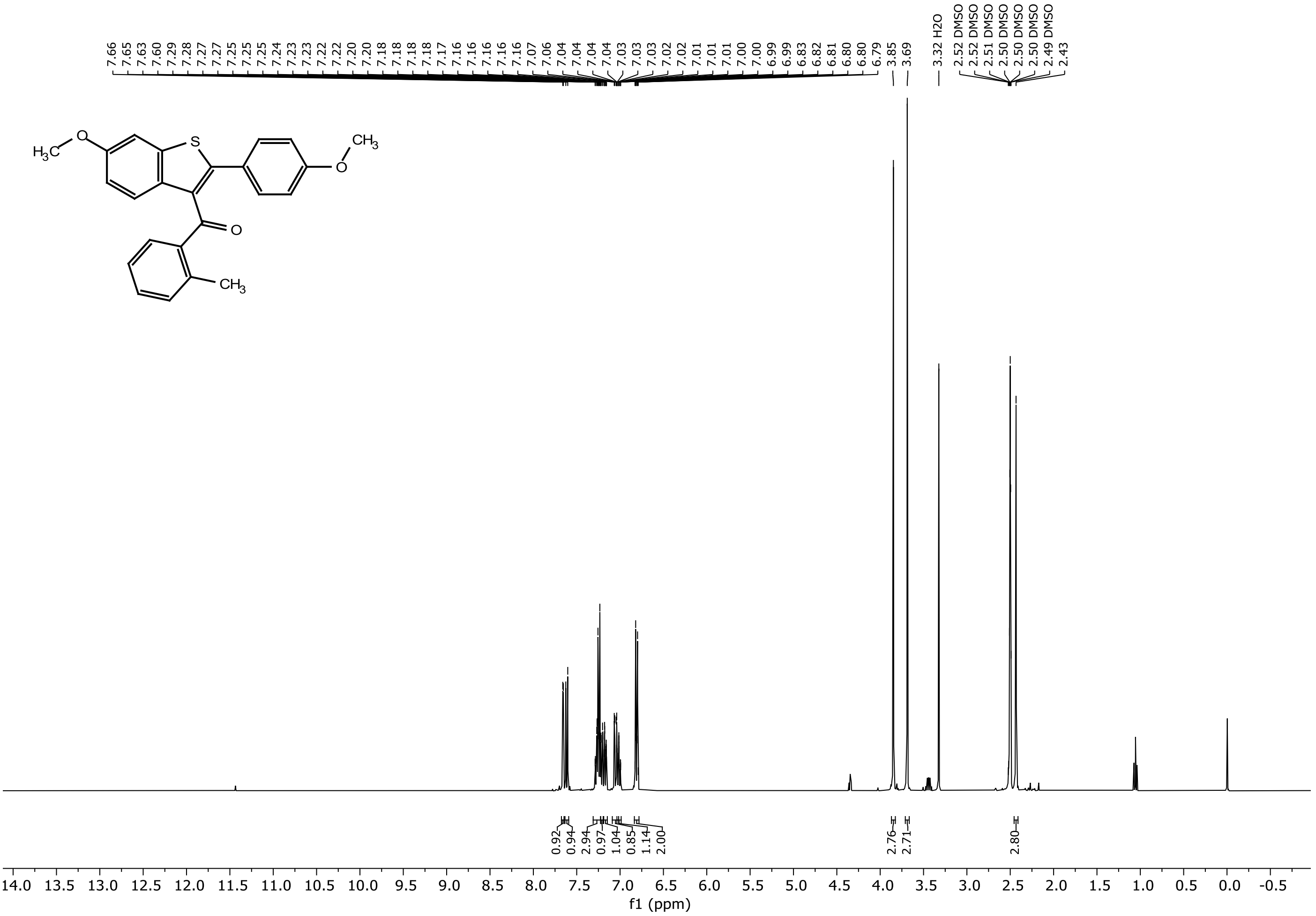

^{13}C NMR (101 MHz, C_2D_6OS) for compound **9k**

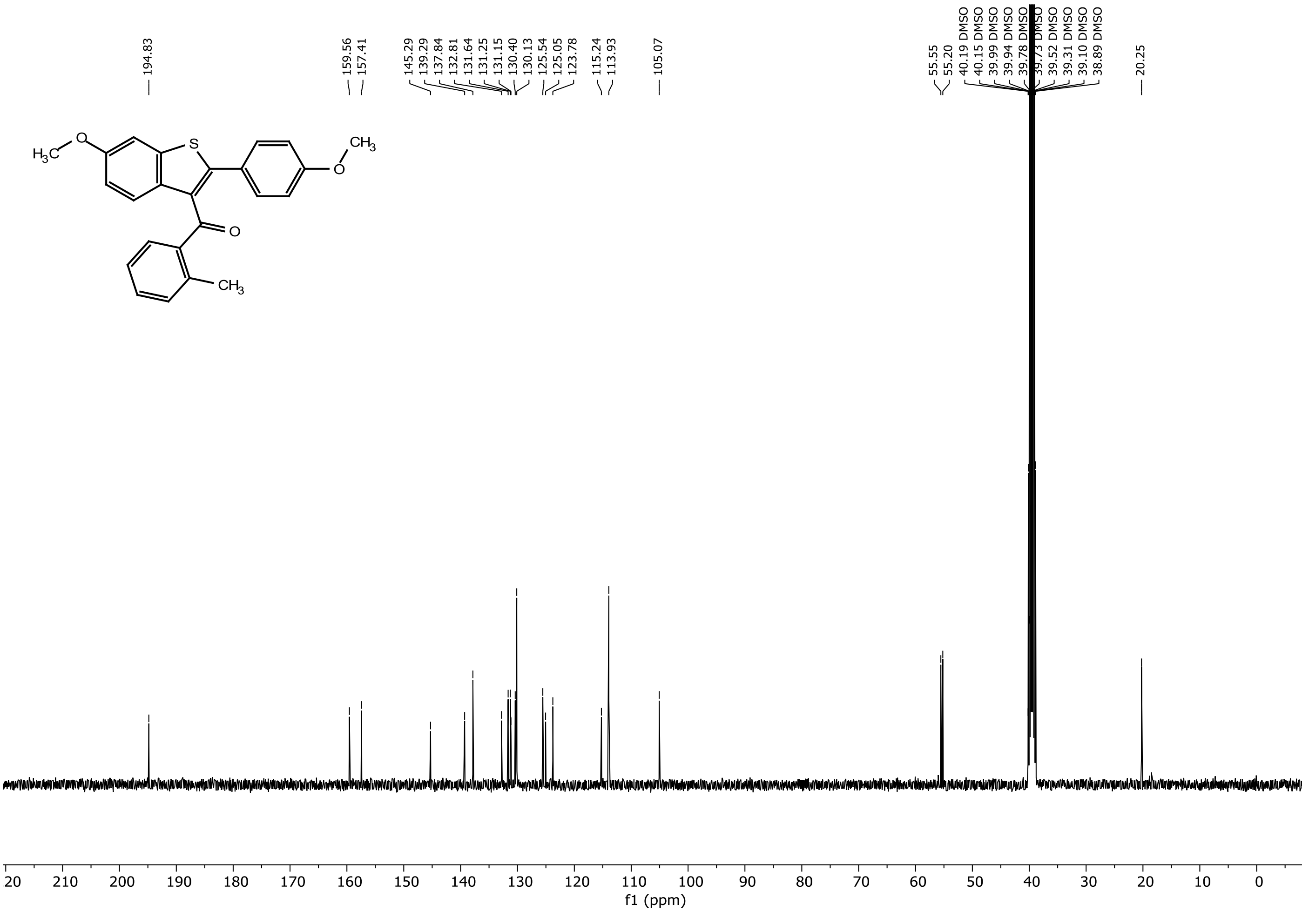

^{1}H NMR (400 MHz, C_2D_6OS) for compound **9l**

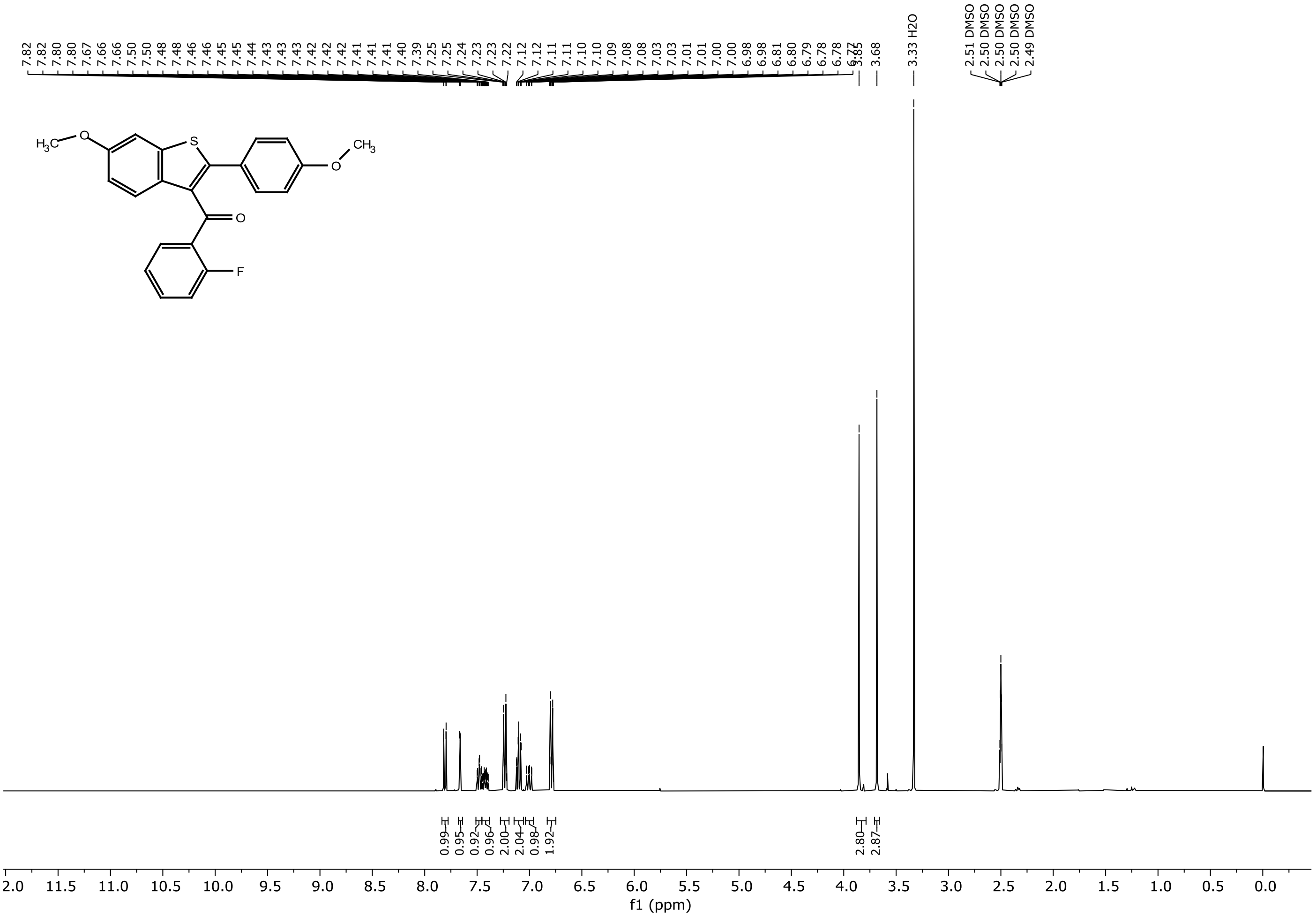

^{13}C NMR (101 MHz, C_2D_6OS) for compound **9l**

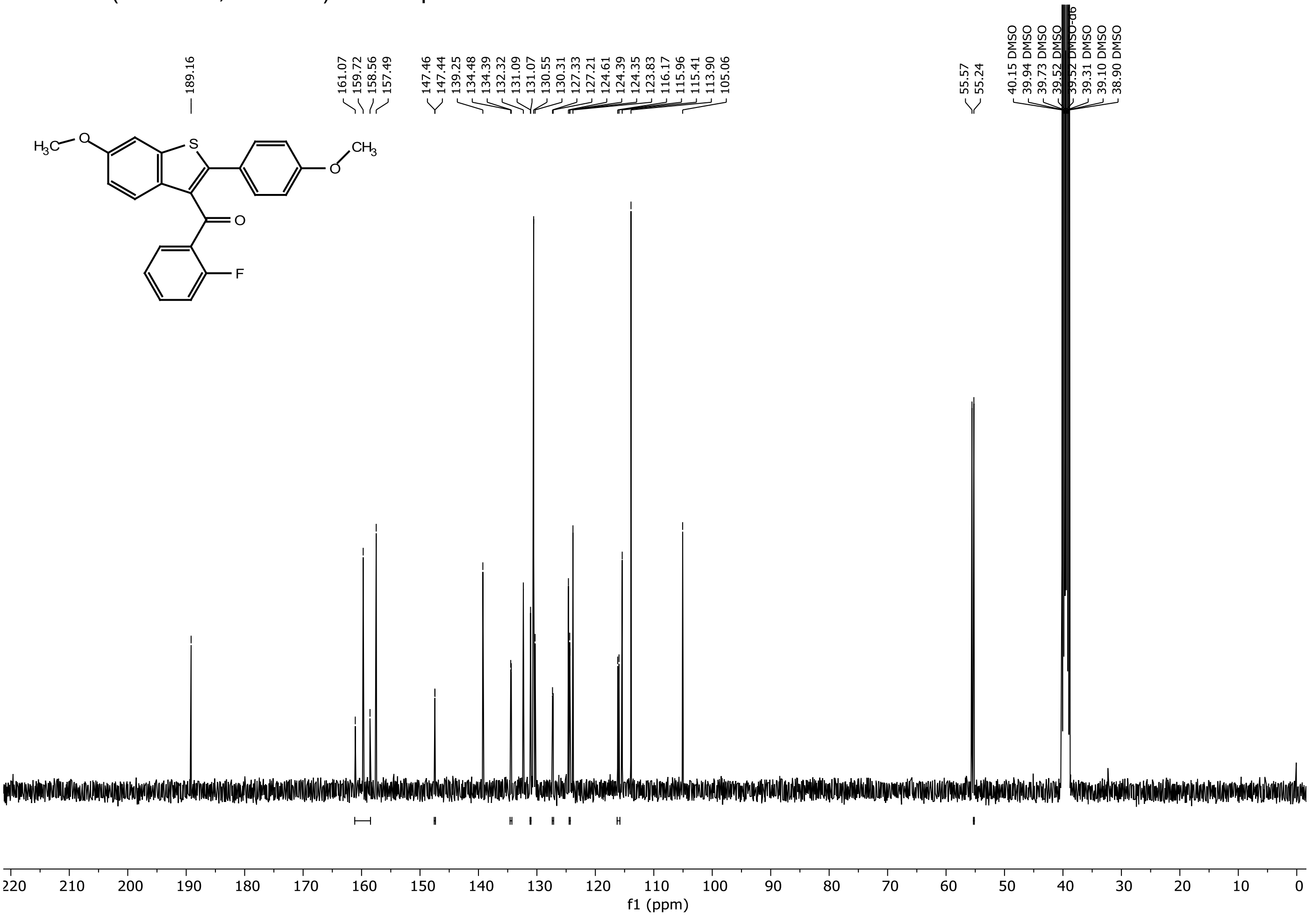

^{1}H NMR (400 MHz, C_2D_6OS) for compound **10d**

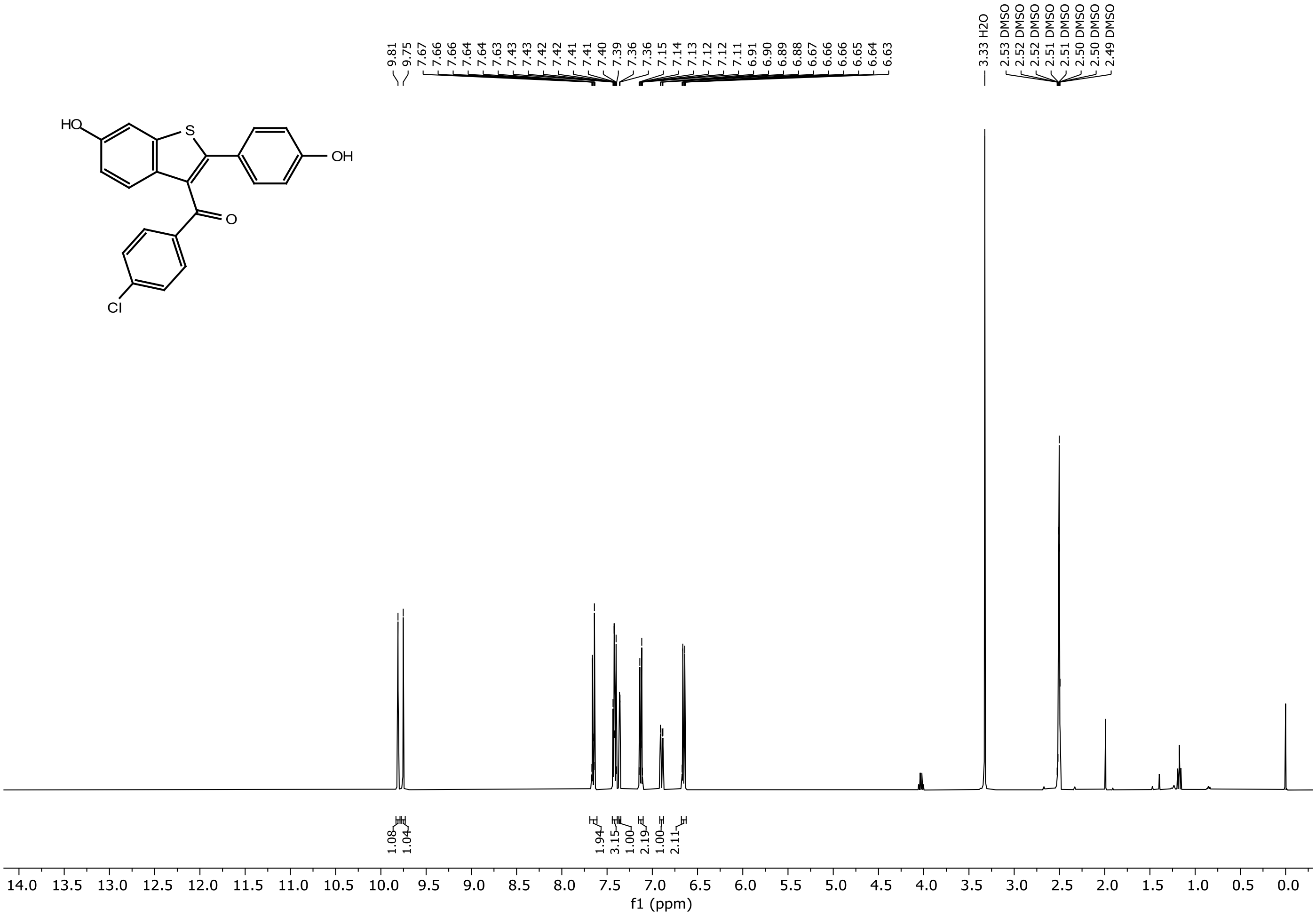

^{13}C NMR (101 MHz, C_2D_6OS) for compound **10d**

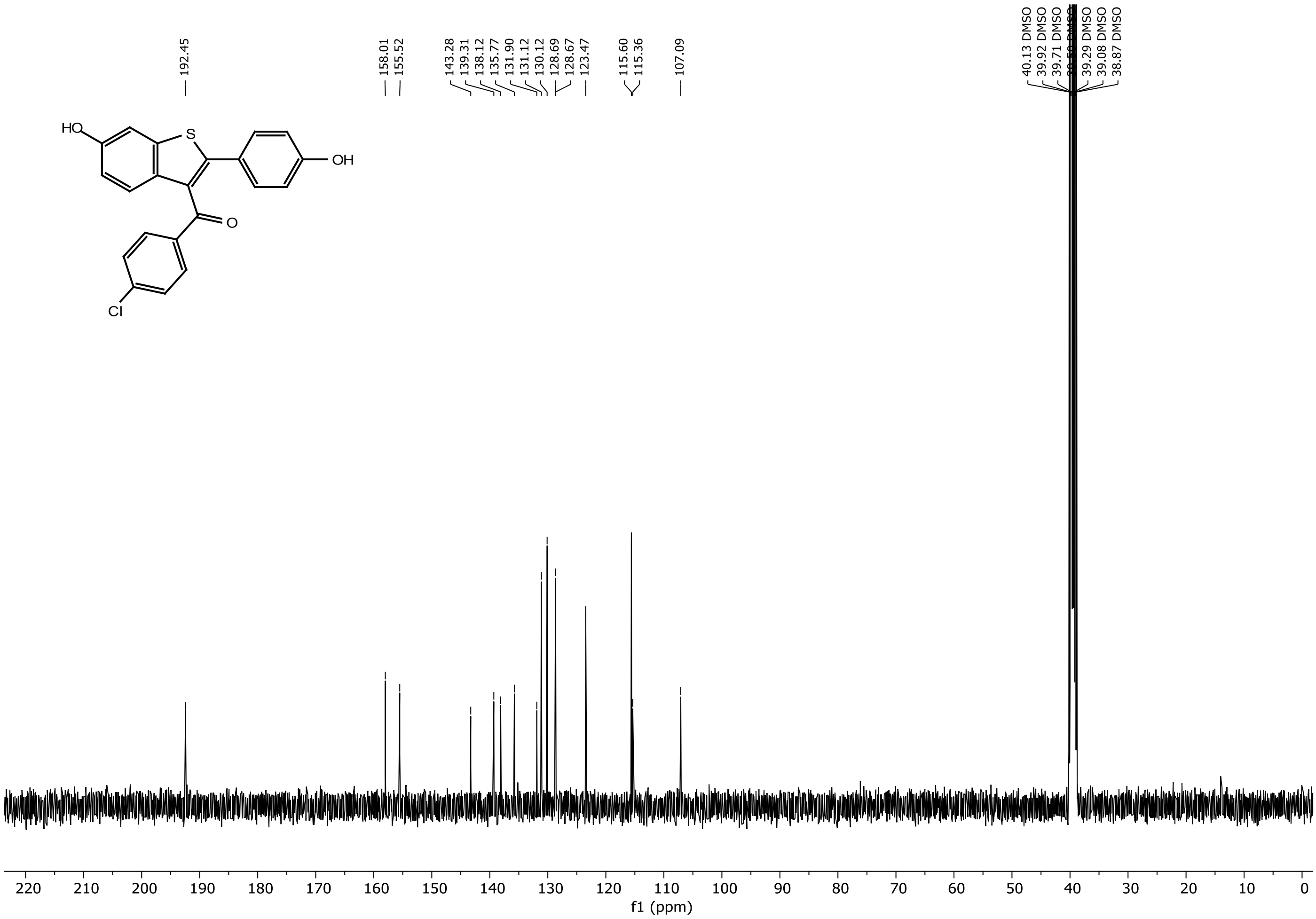

^{1}H NMR (500 MHz, C_2D_6OS) for compound **10f**

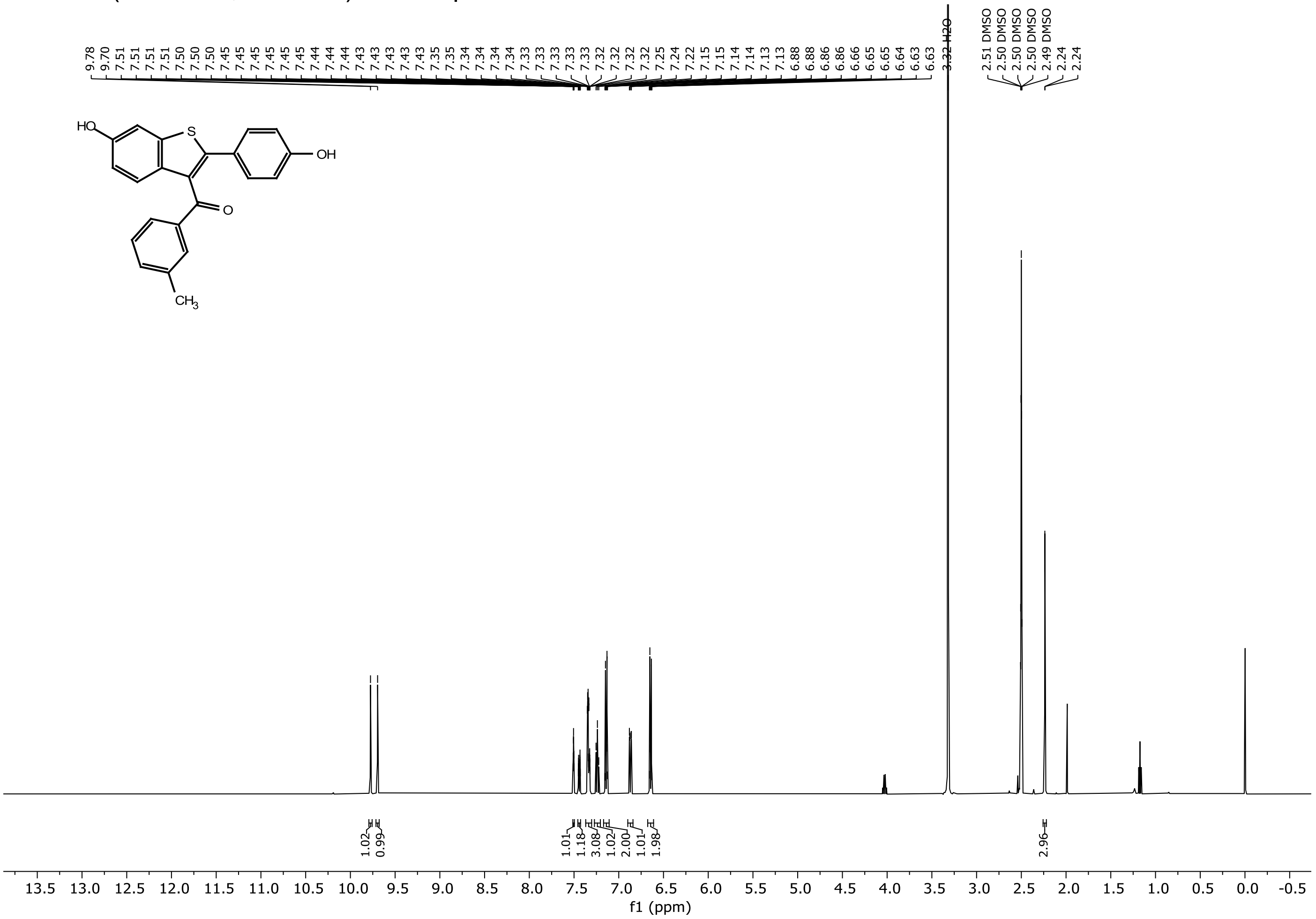

^{13}C NMR (126 MHz, C_2D_6OS) for compound **10f**

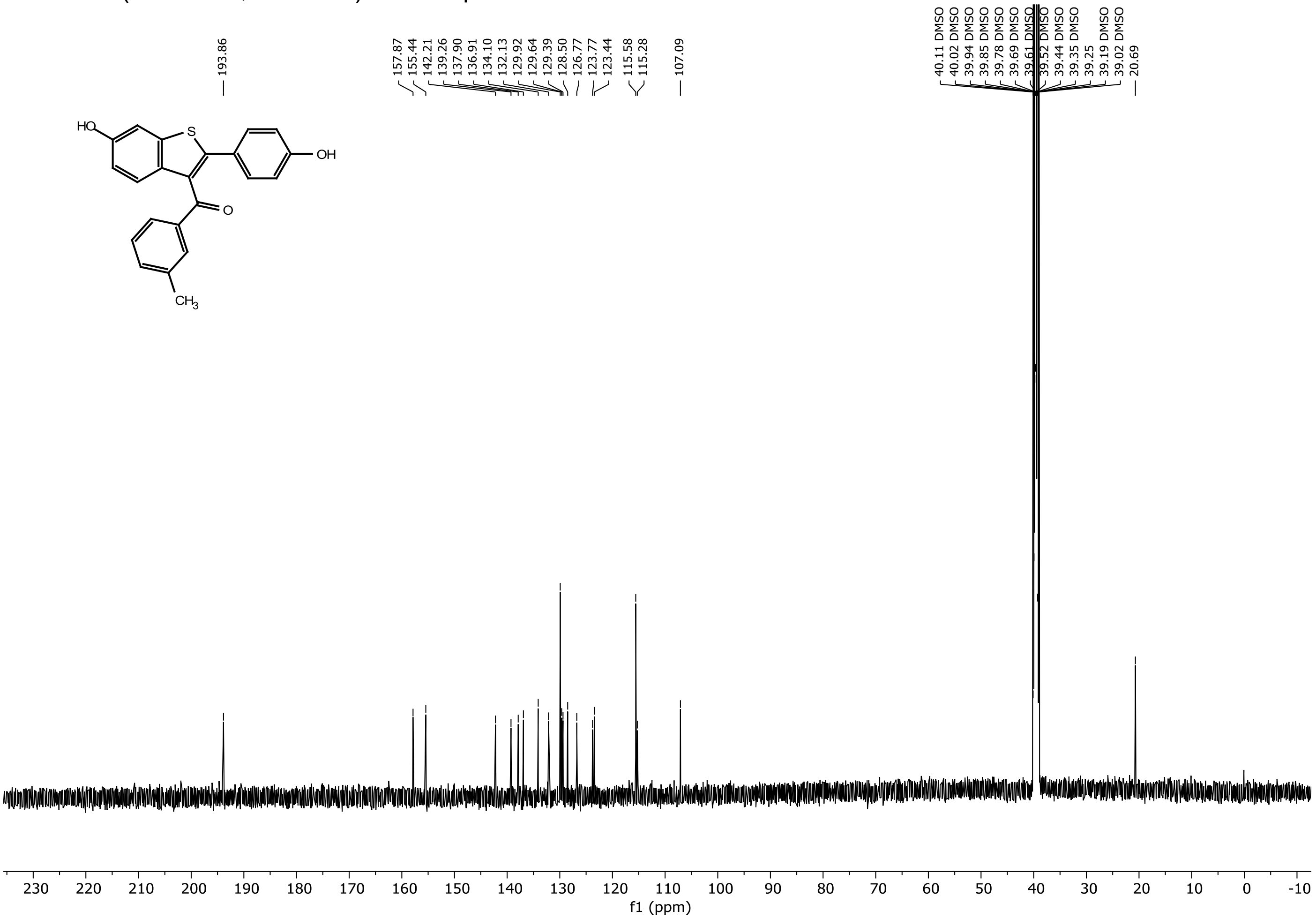

^{1}H NMR (500 MHz, C_2D_6OS) for compound **10g**

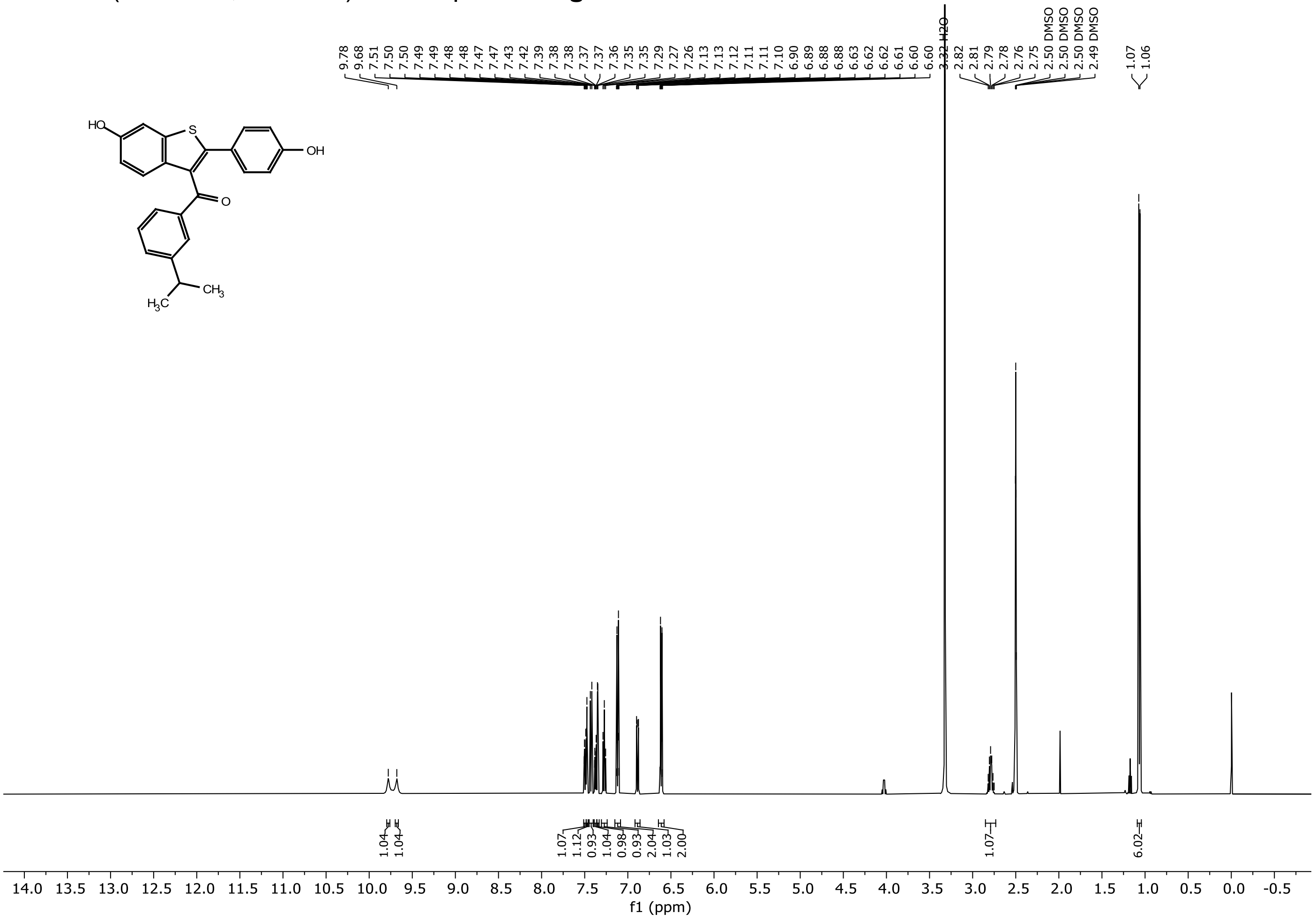

^{13}C NMR (126 MHz, C_2D_6OS) for compound **10g**

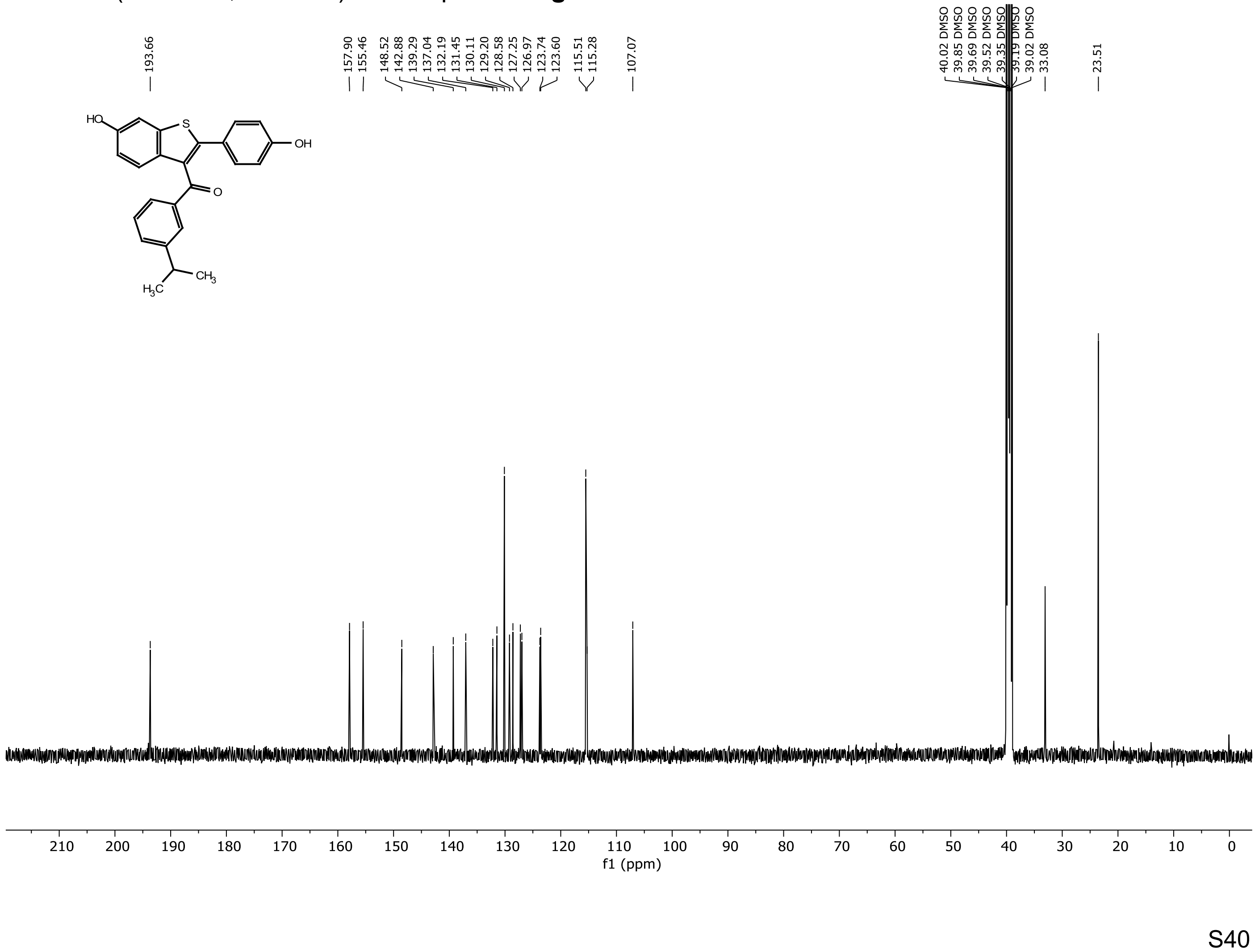

^1H NMR (400 MHz, C_2D_6OS) for compound **10h**

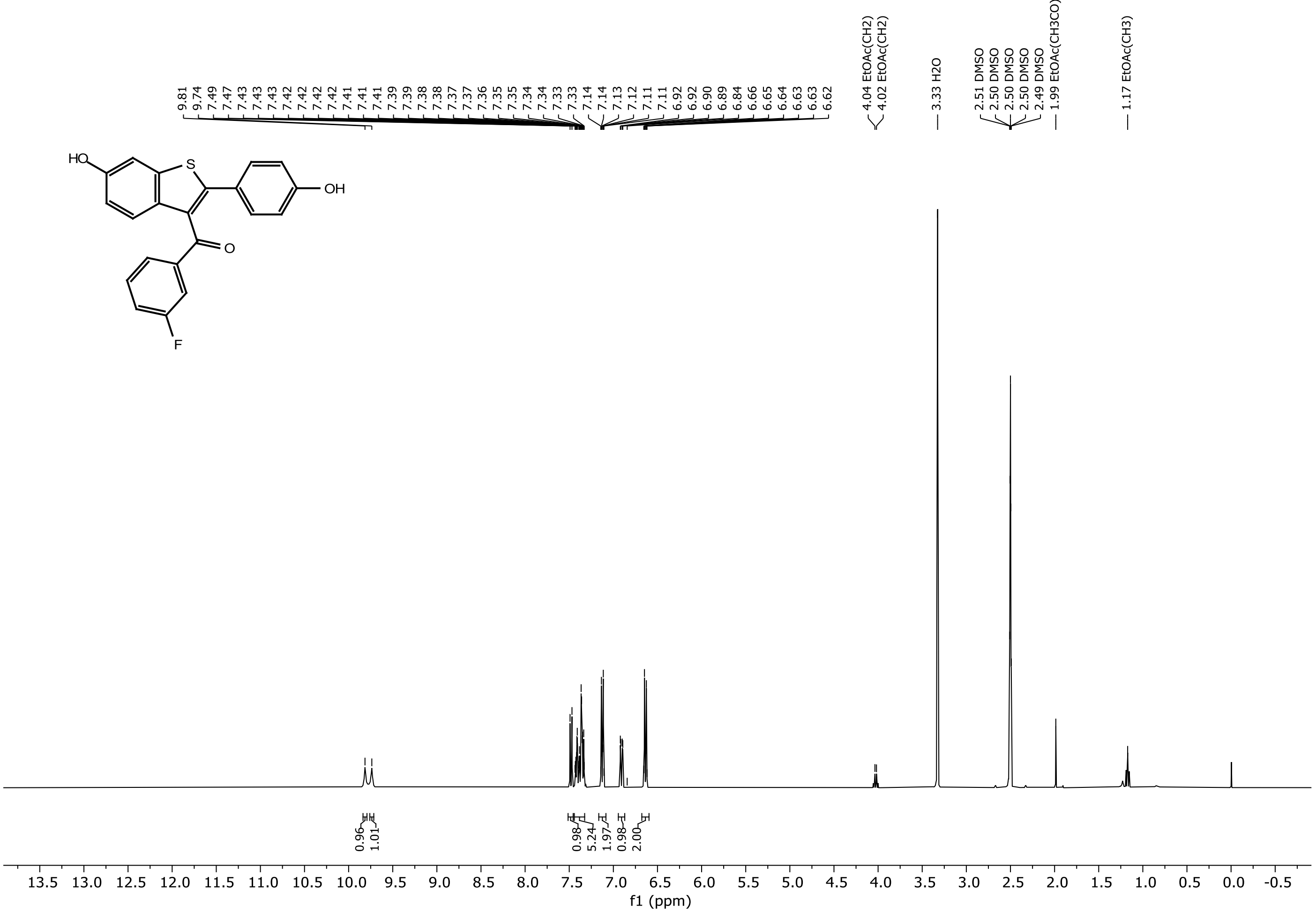

^{13}C NMR (101 MHz, C_2D_6OS) for compound **10h**

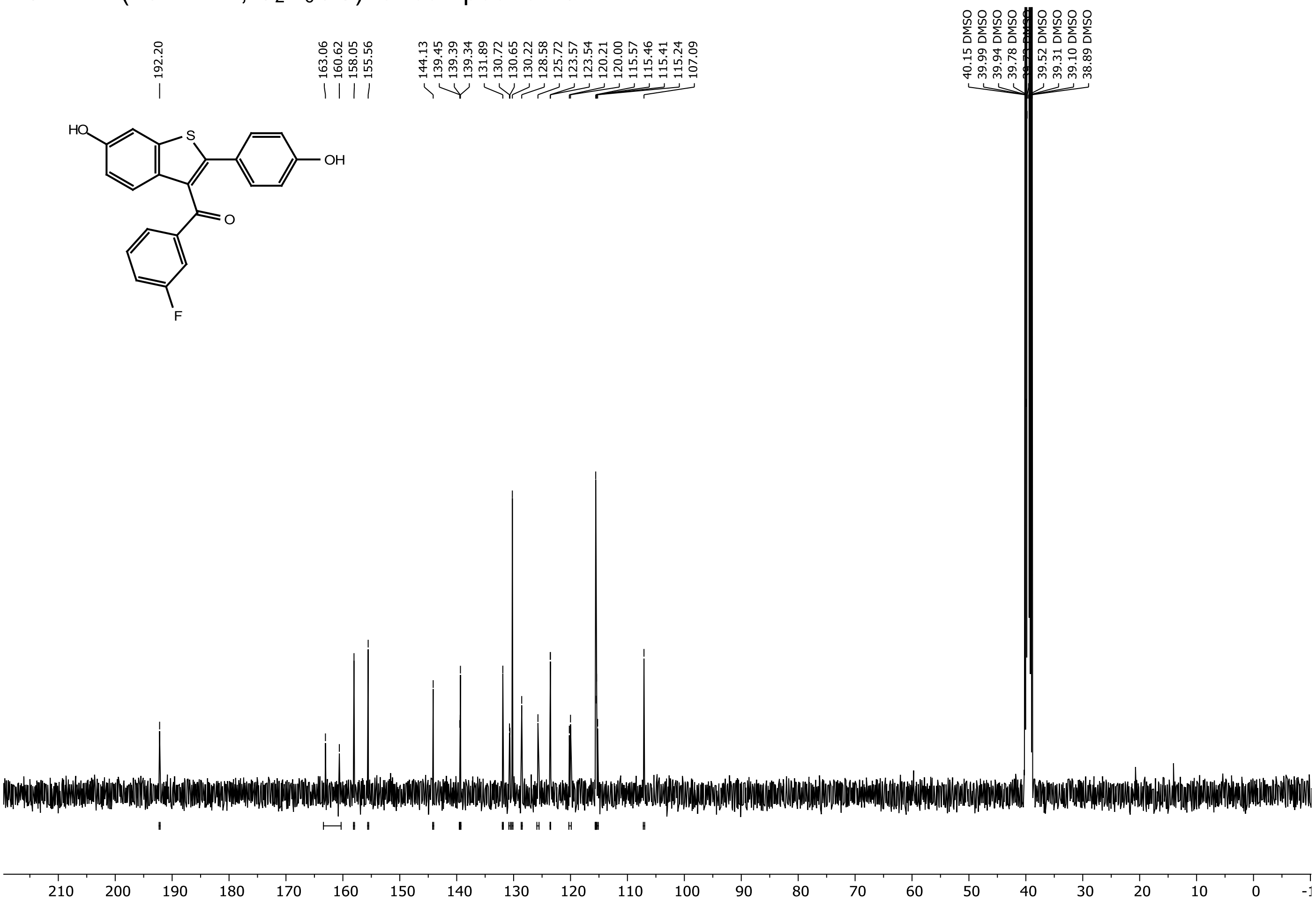

^{1}H NMR (400 MHz, C_2D_6OS) for compound **10i**

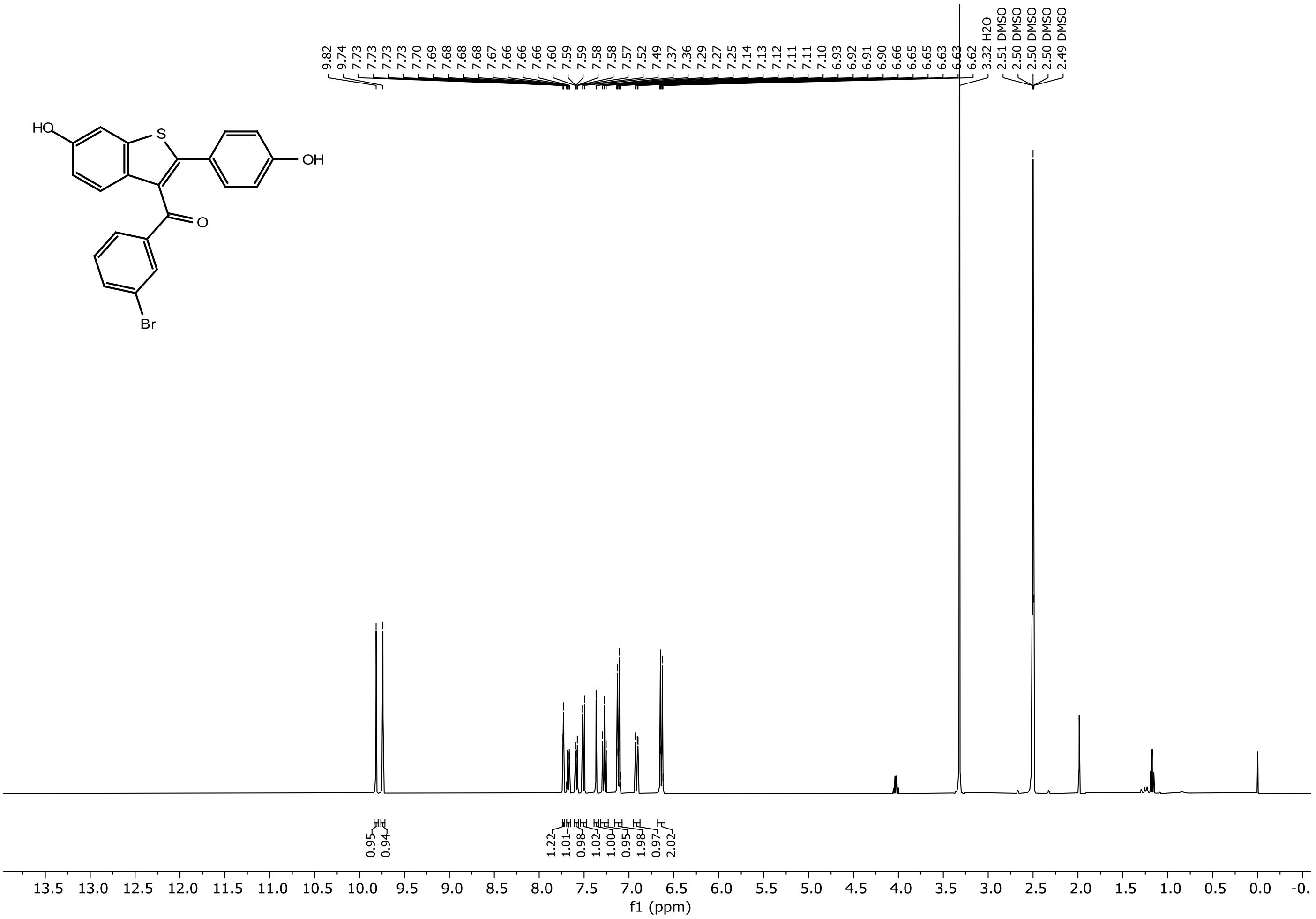

^{13}C NMR (101 MHz, C_2D_6OS) for compound **10i**

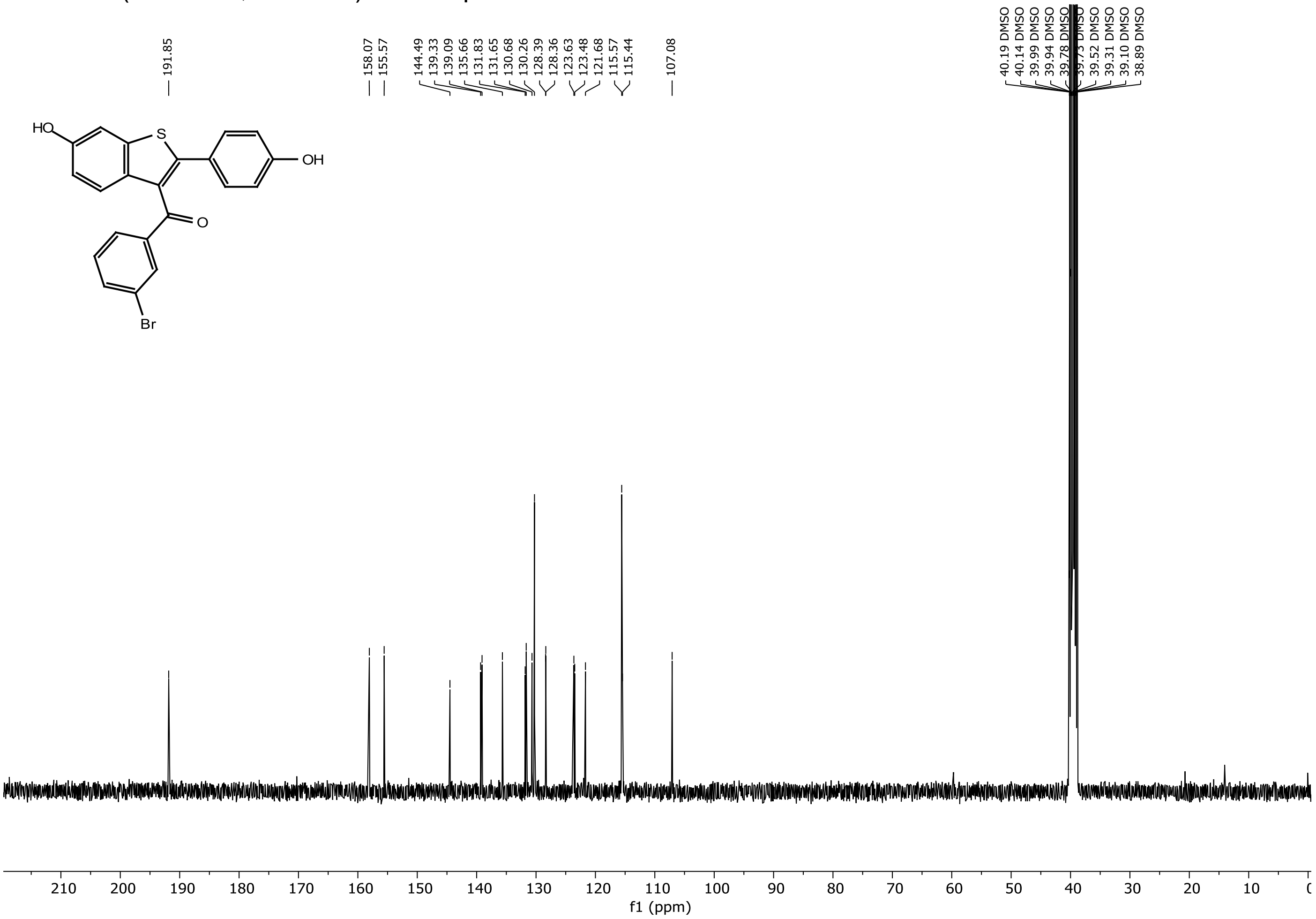

^{1}H NMR (400 MHz, C_2D_6OS) for compound **10j**

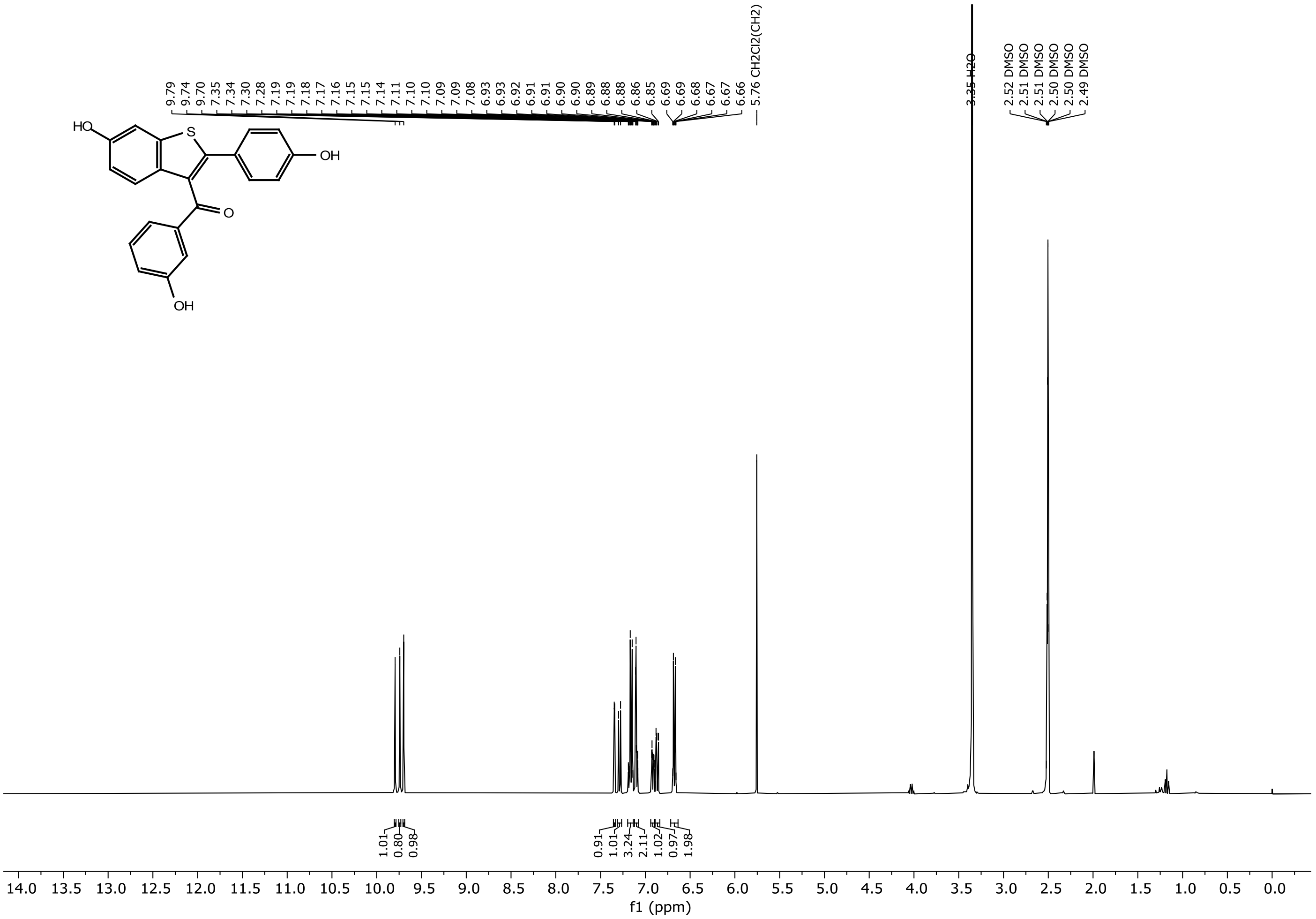

^{13}C NMR (101 MHz, C_2D_6OS) for compound **10j**

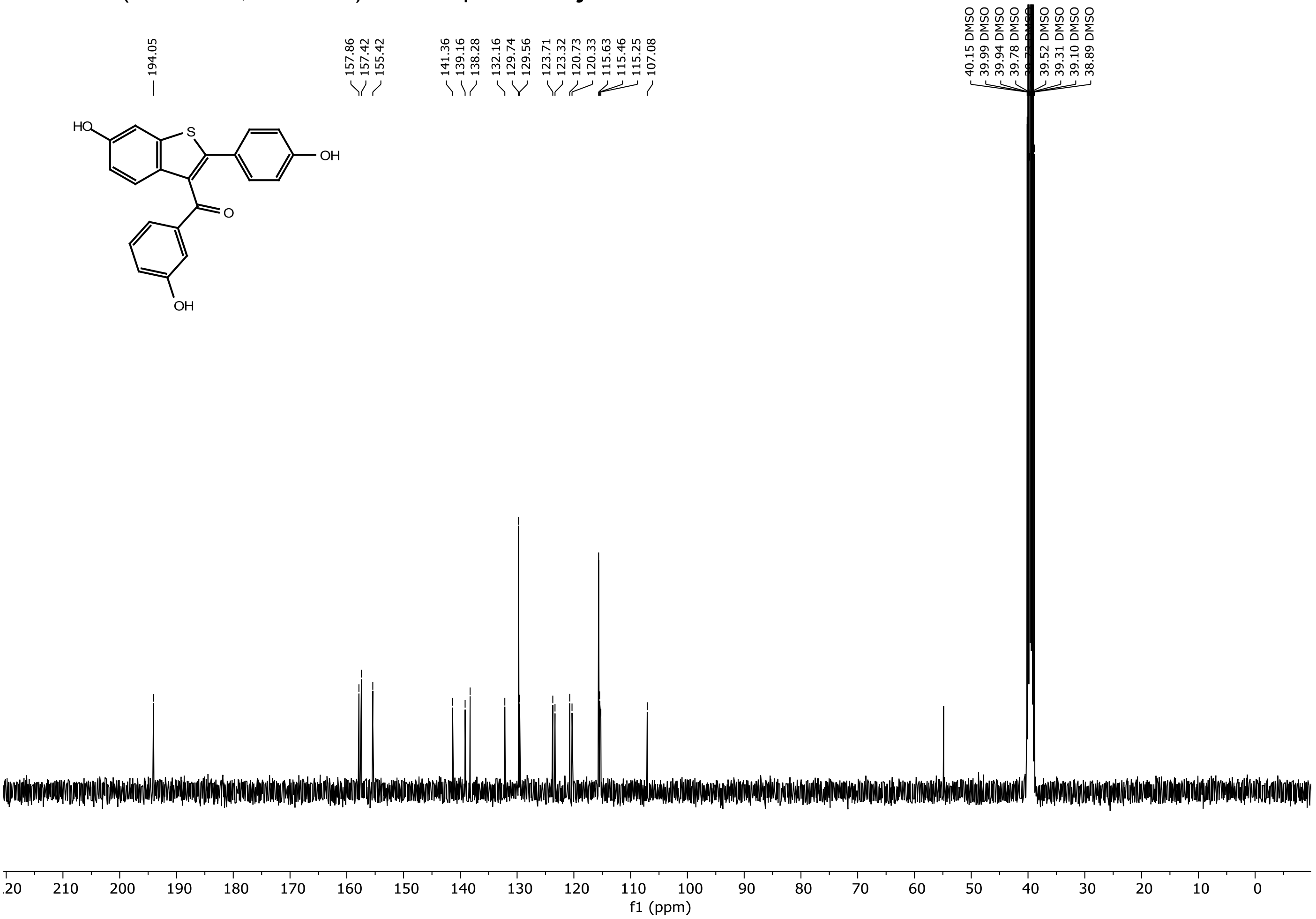

^{1}H NMR (500 MHz, C_2D_6OS) for compound **10k**

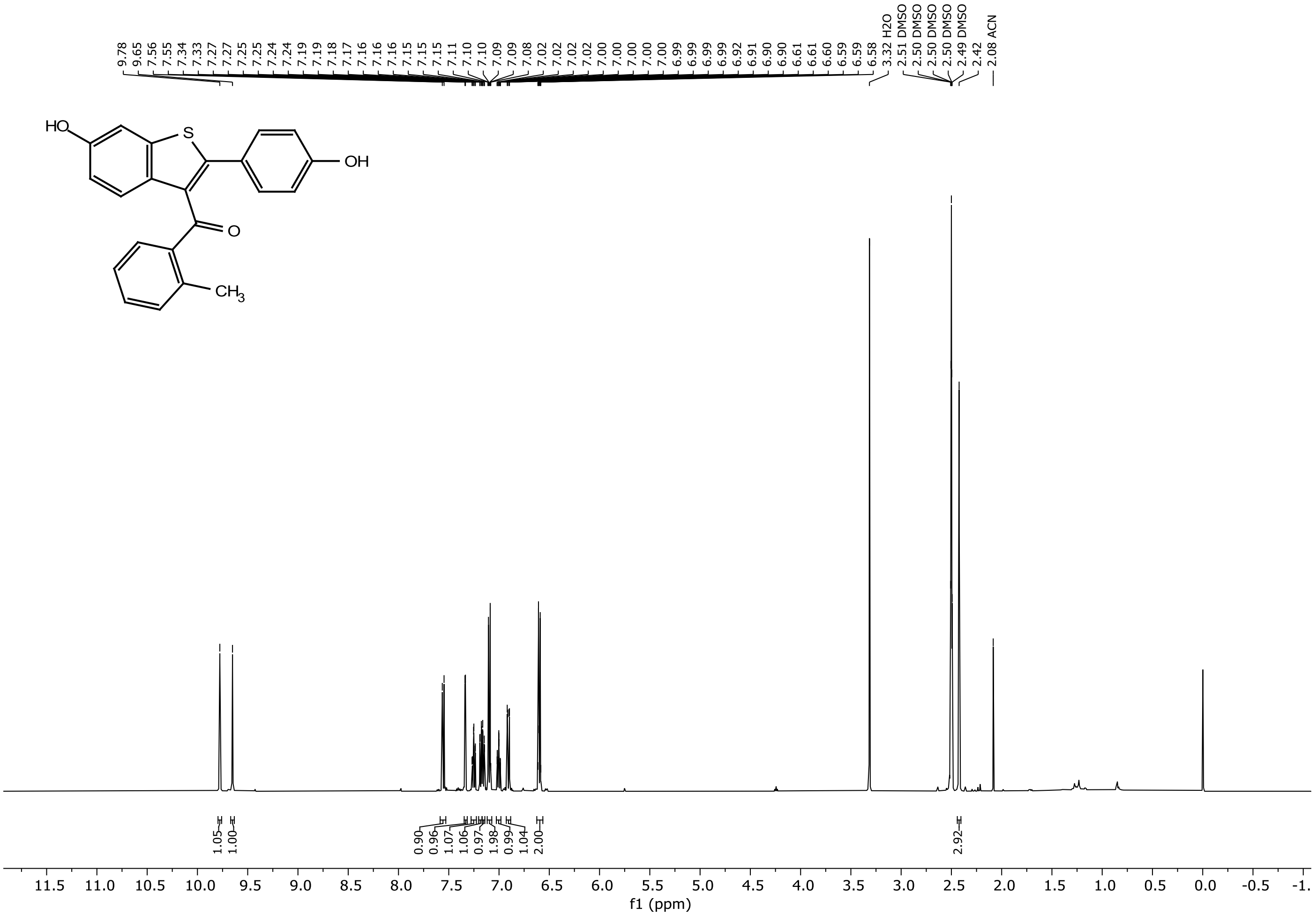

^{13}C NMR (126 MHz, C_2D_6OS) for compound **10k**

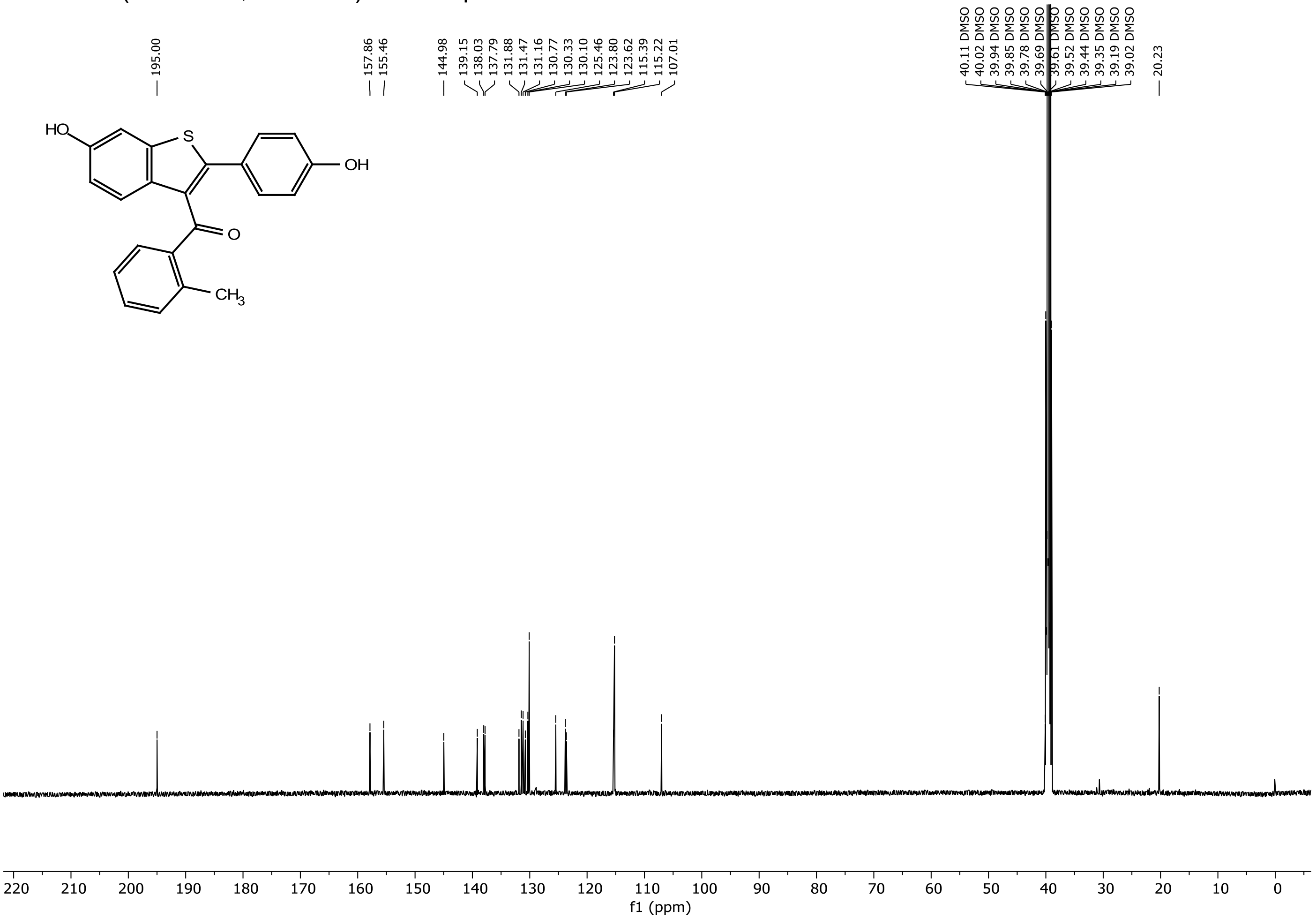

^{1}H NMR (500 MHz, C_2D_6OS) for compound **10l**

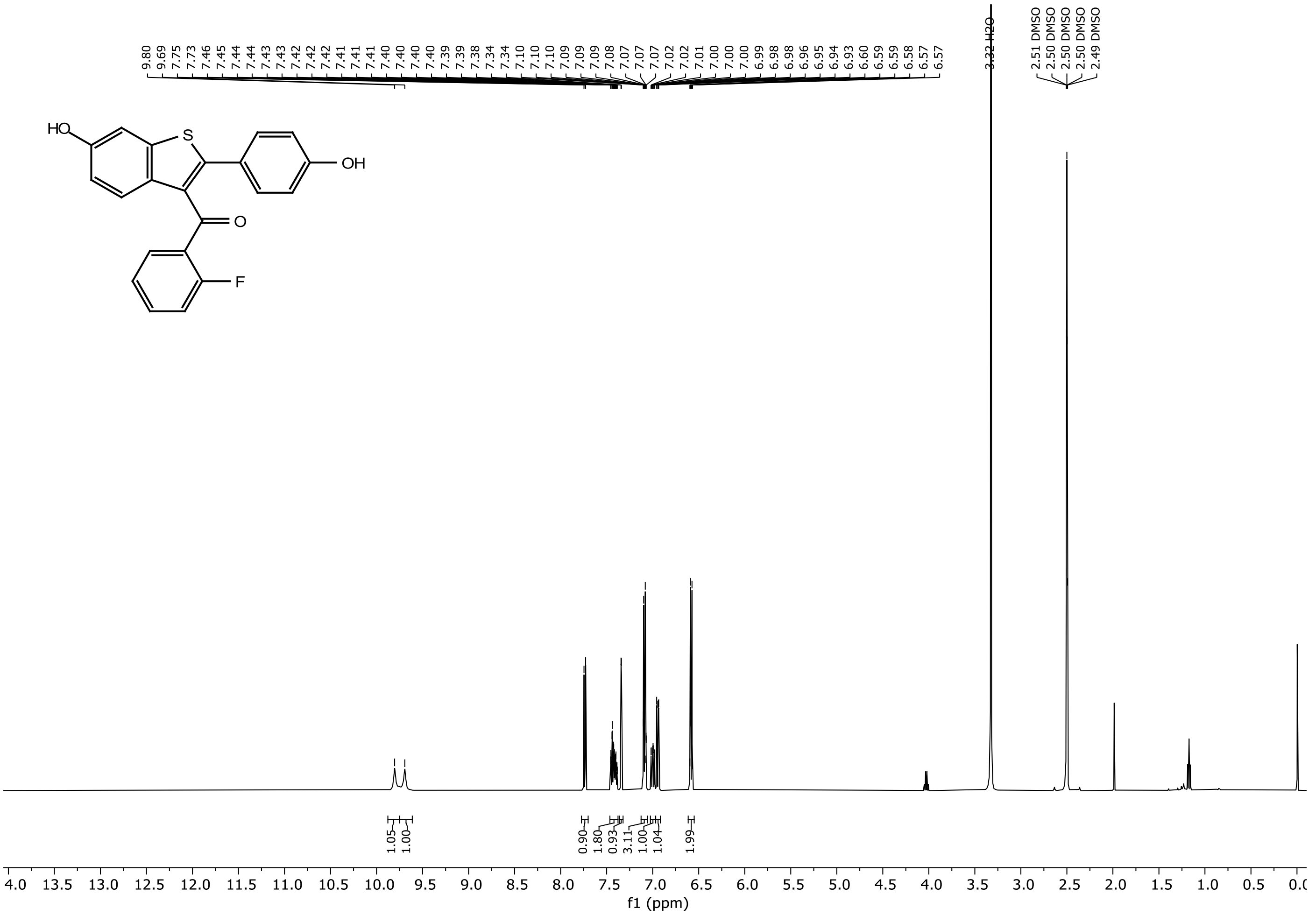

^{13}C NMR (126 MHz, C_2D_6OS) for compound **10l**

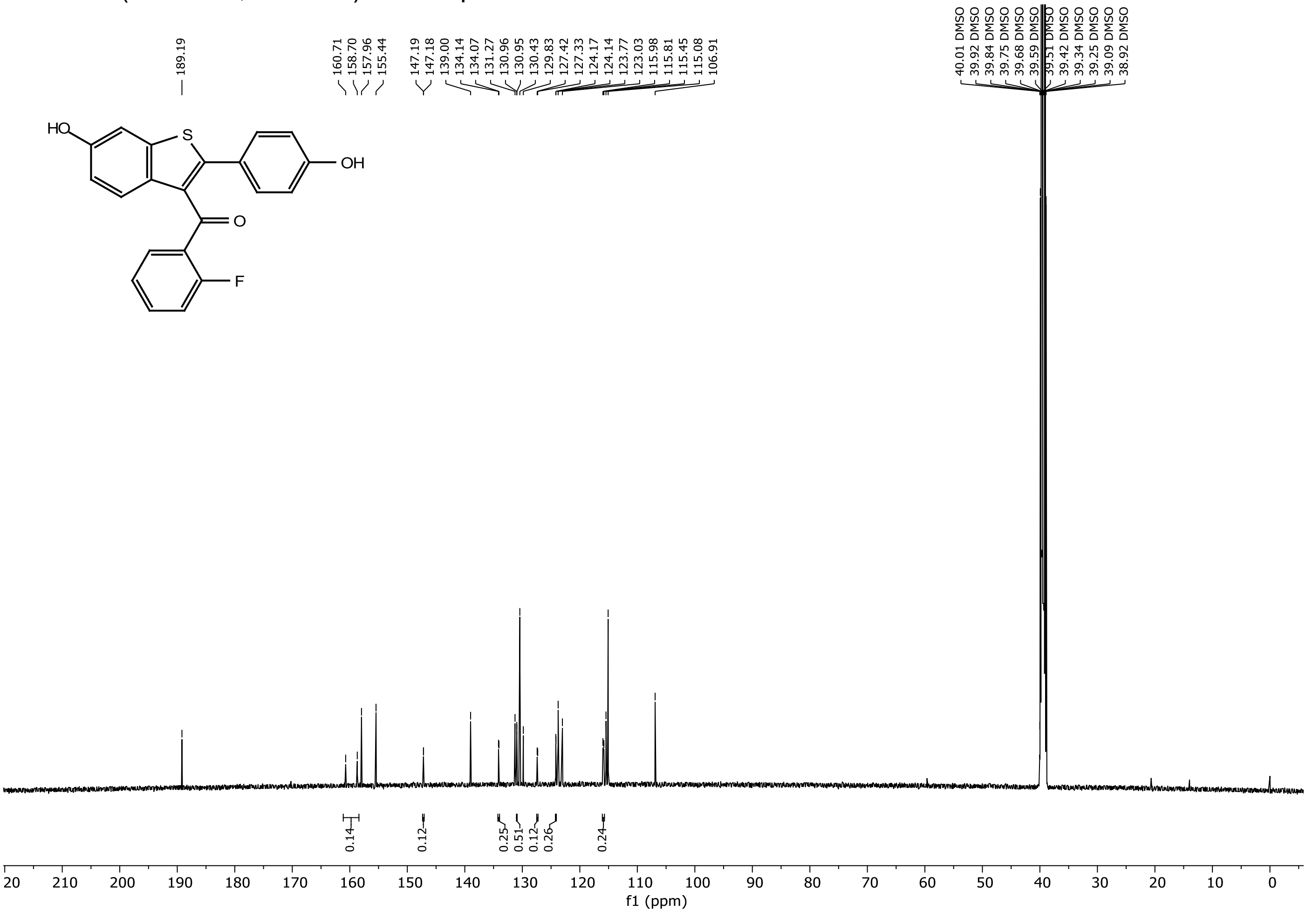

^{1}H NMR (500 MHz, C_2D_6OS) for compound **11a**

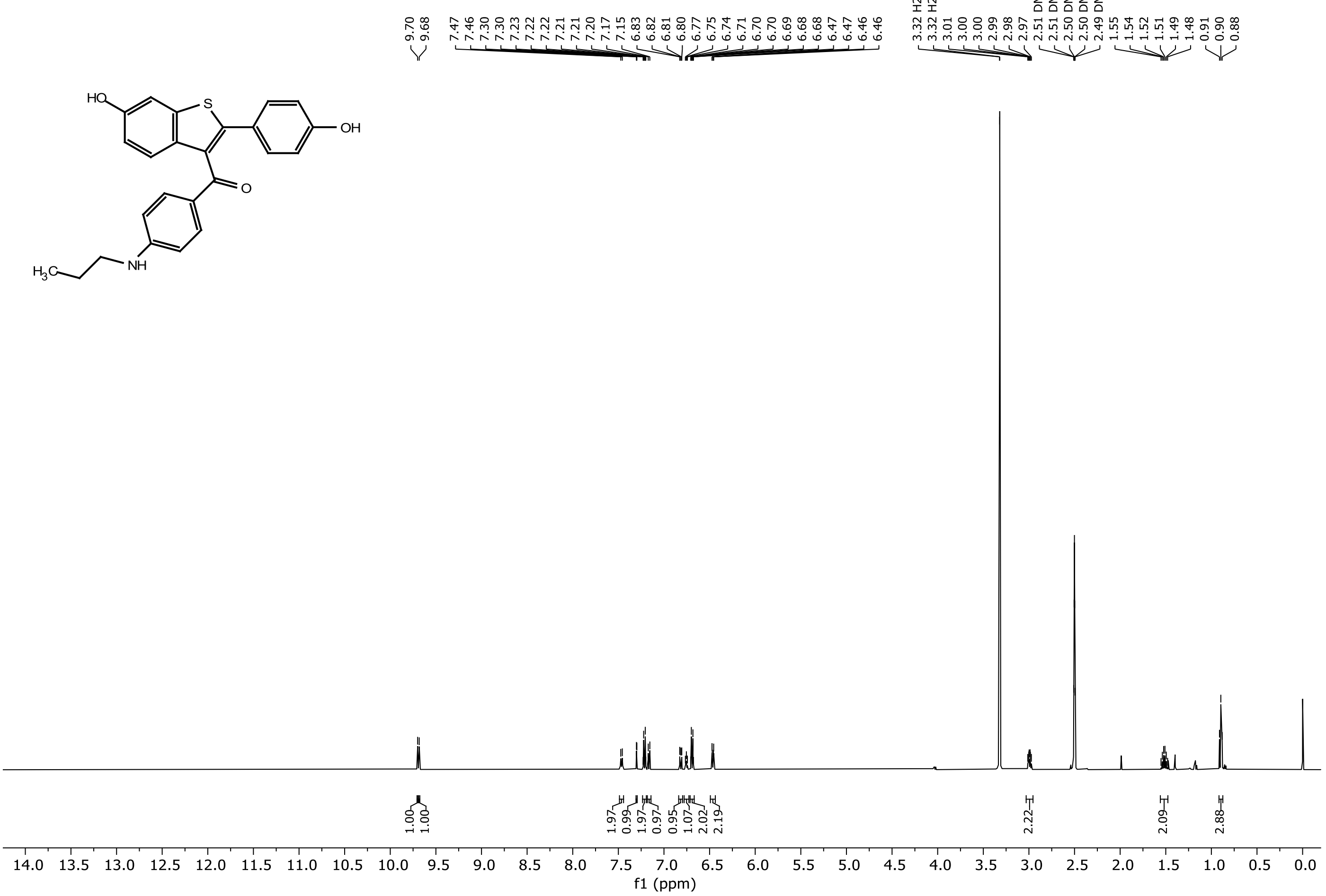

^{13}C NMR (126 MHz, C_2D_6OS) for compound **11a**

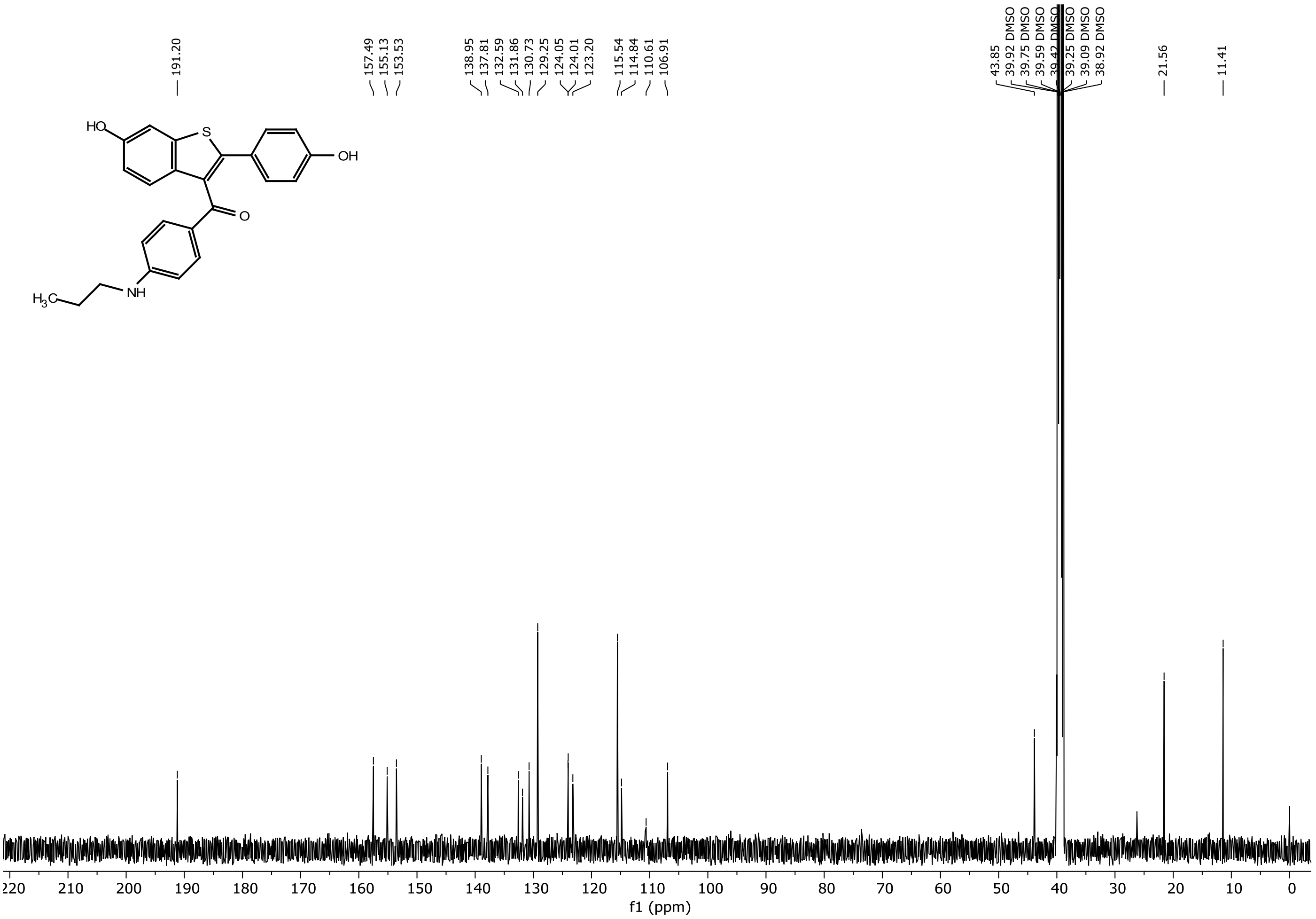

^{1}H NMR (500 MHz, C_2D_6OS) for compound **11b**

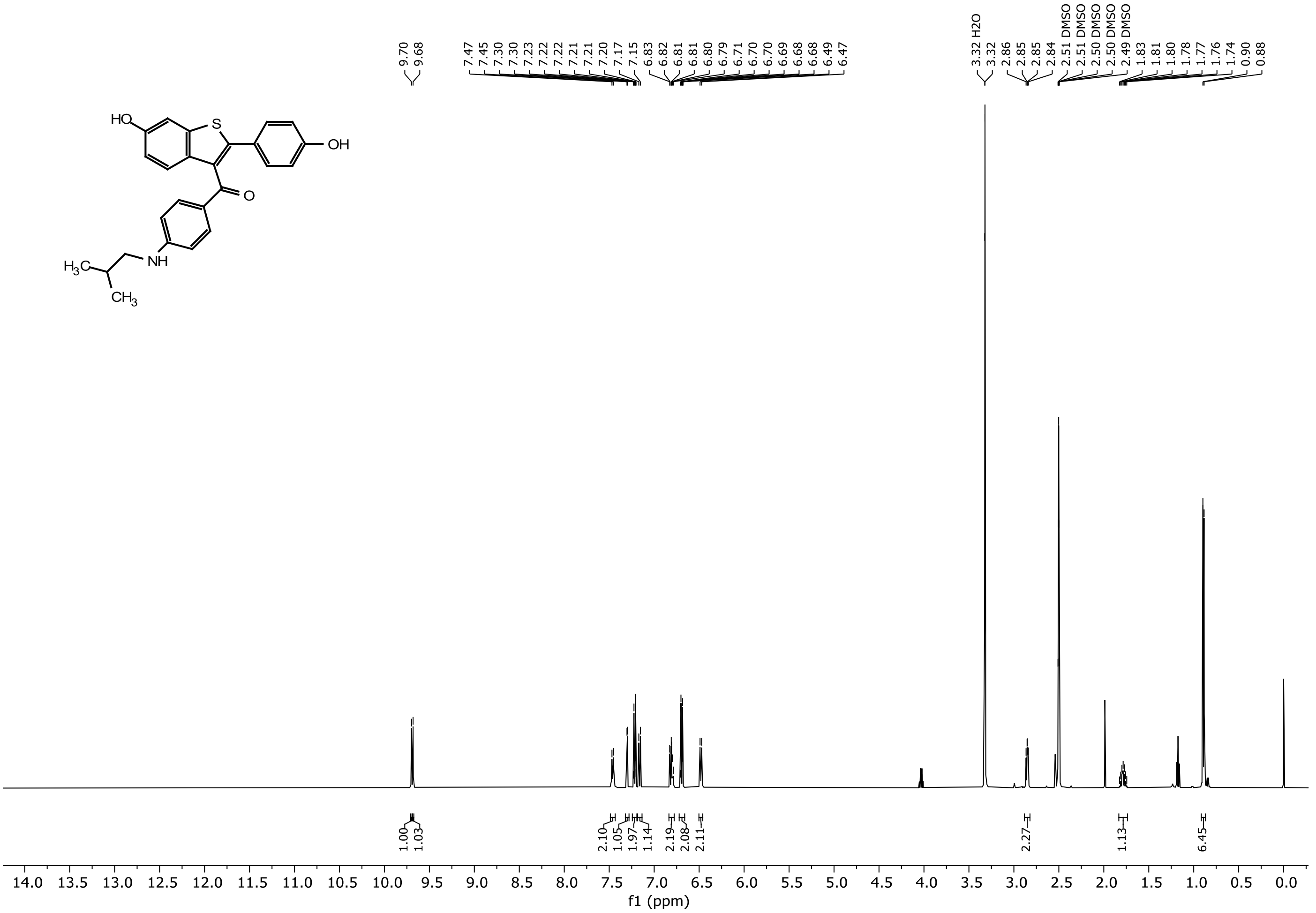

^{13}C NMR (126 MHz, C_2D_6OS) for compound **11b**

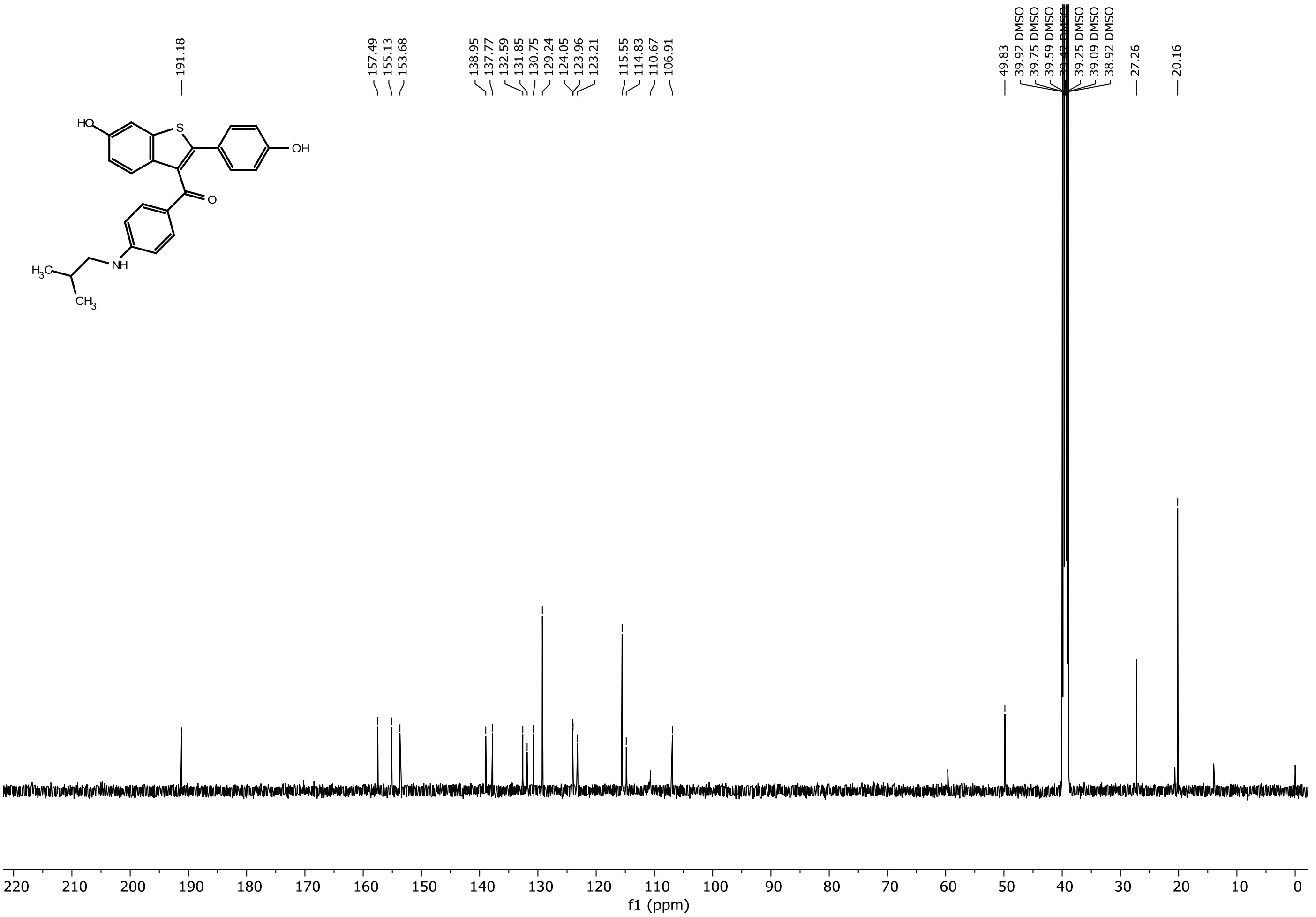

^{1}H NMR (500 MHz, C_2D_6OS) for compound **11c**

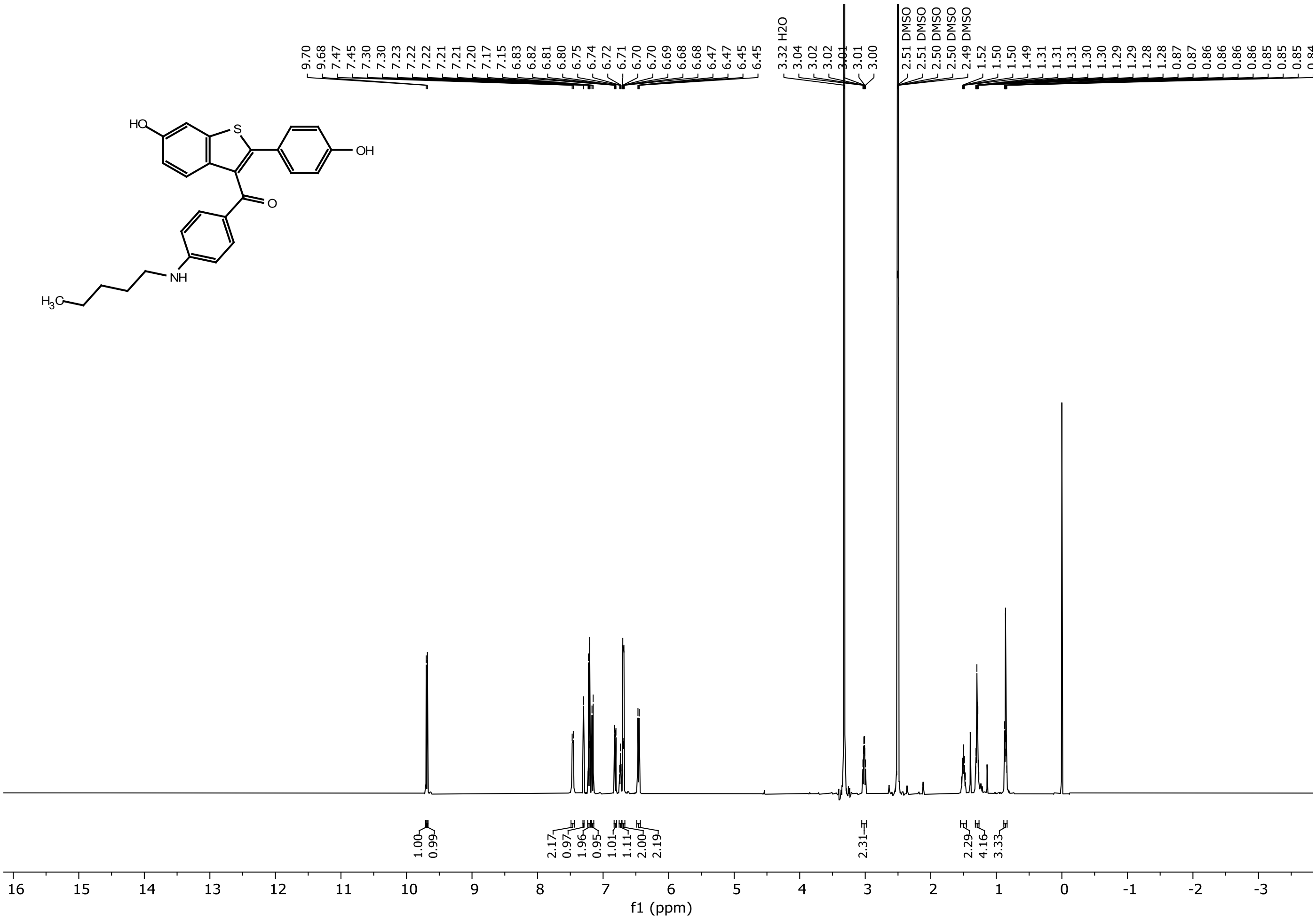

^{13}C NMR (126 MHz, C_2D_6OS) for compound **11c**

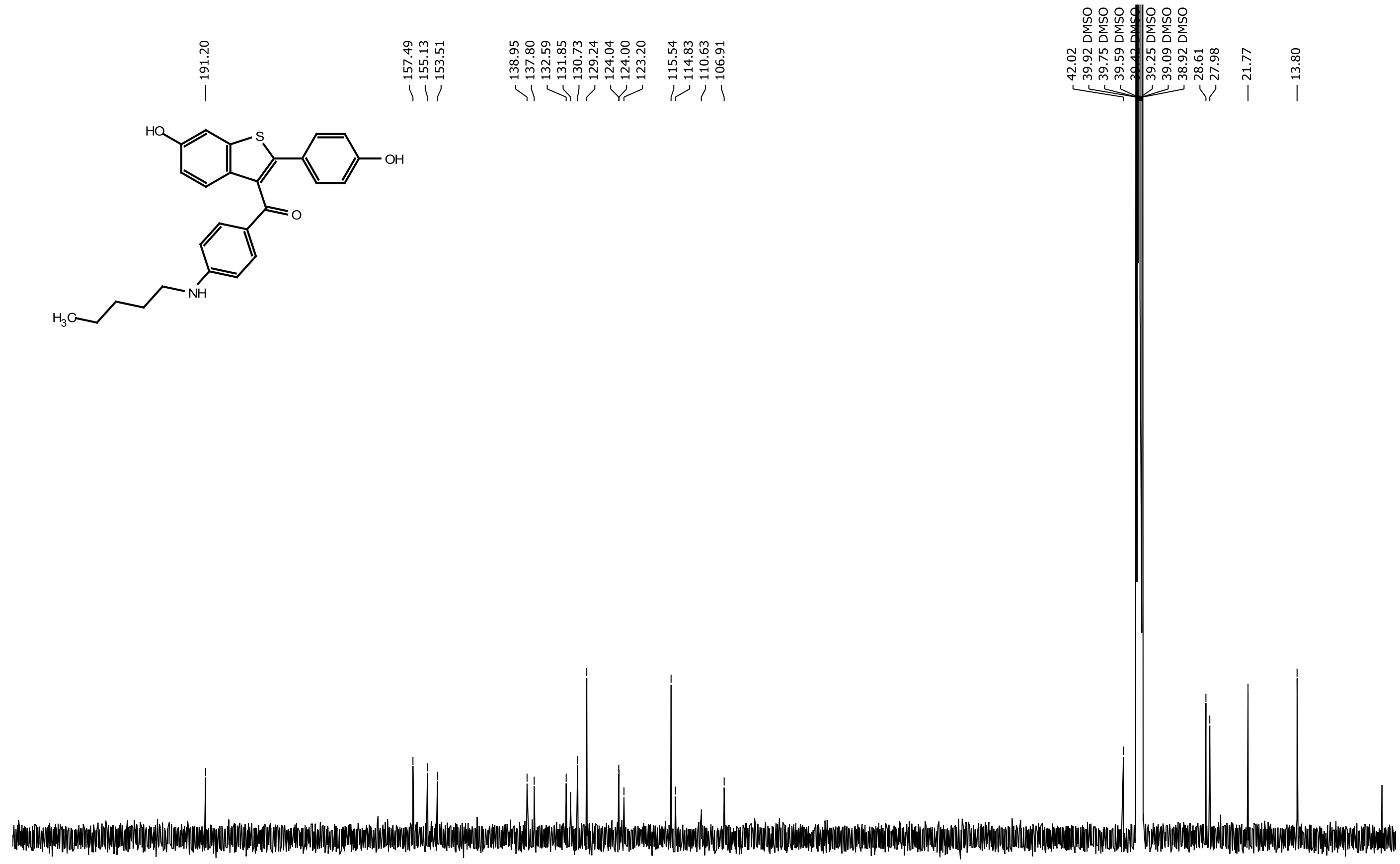

^{1}H NMR (500 MHz, C_2D_6OS) for compound **11d**

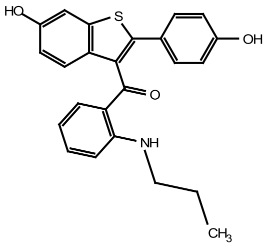

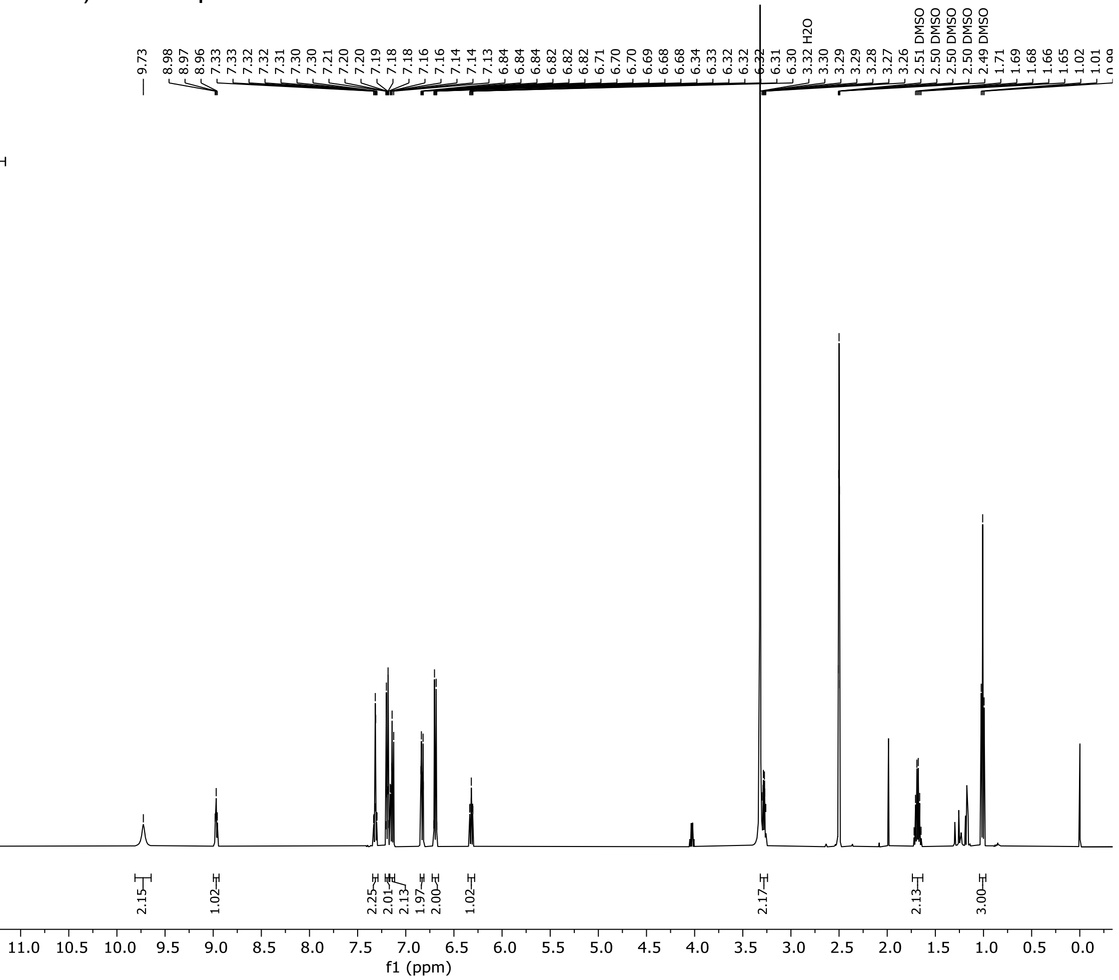

^{13}C NMR (126 MHz, C_2D_6OS) for compound **11d**

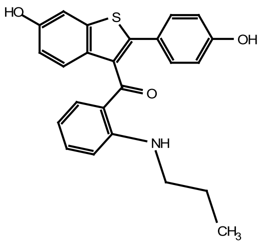

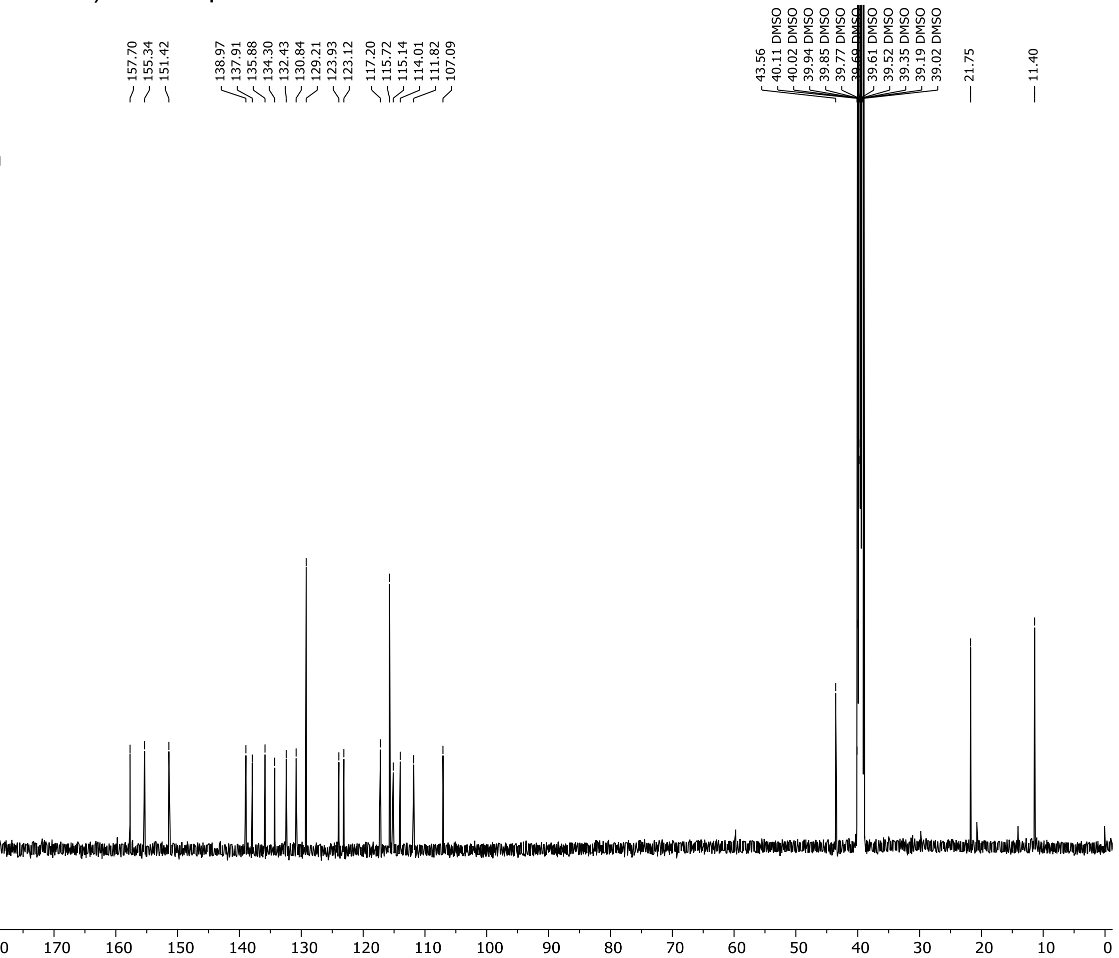

^{1}H NMR (500 MHz, C_2D_6OS) for compound **11e**

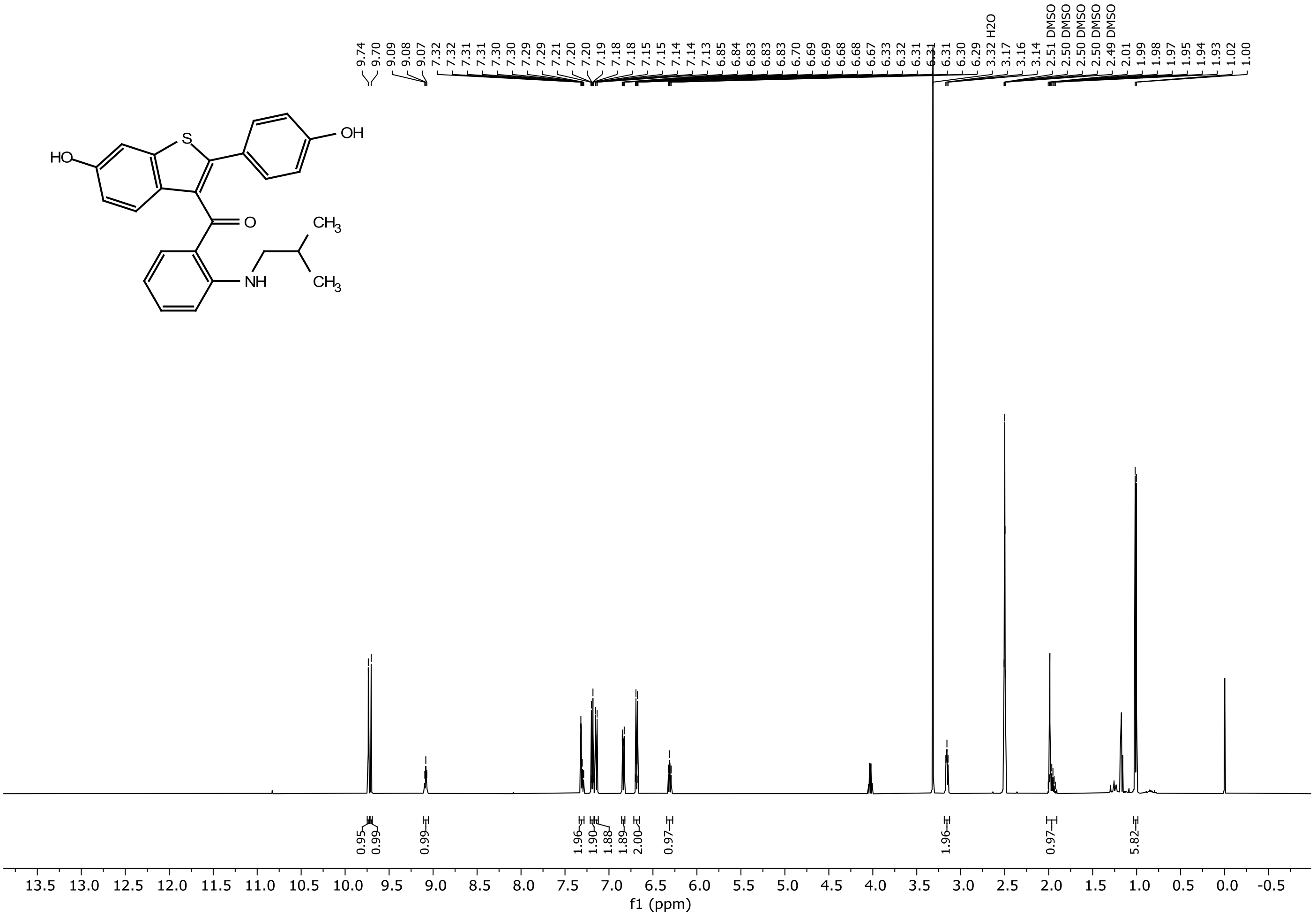

^{13}C NMR (126 MHz, C_2D_6OS) for compound **11e**

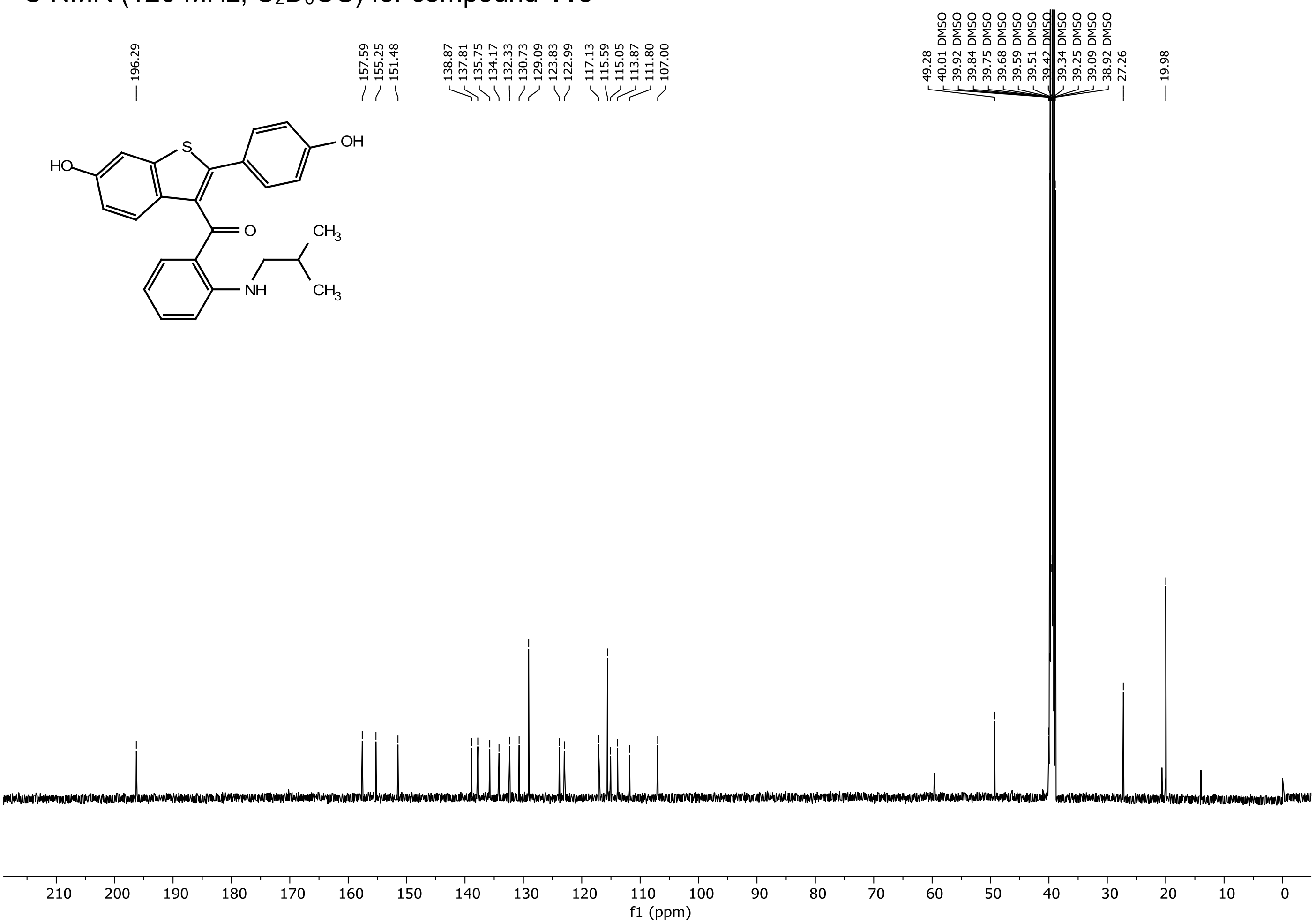

^{1}H NMR (500 MHz, C_2D_6OS) for compound **11f**

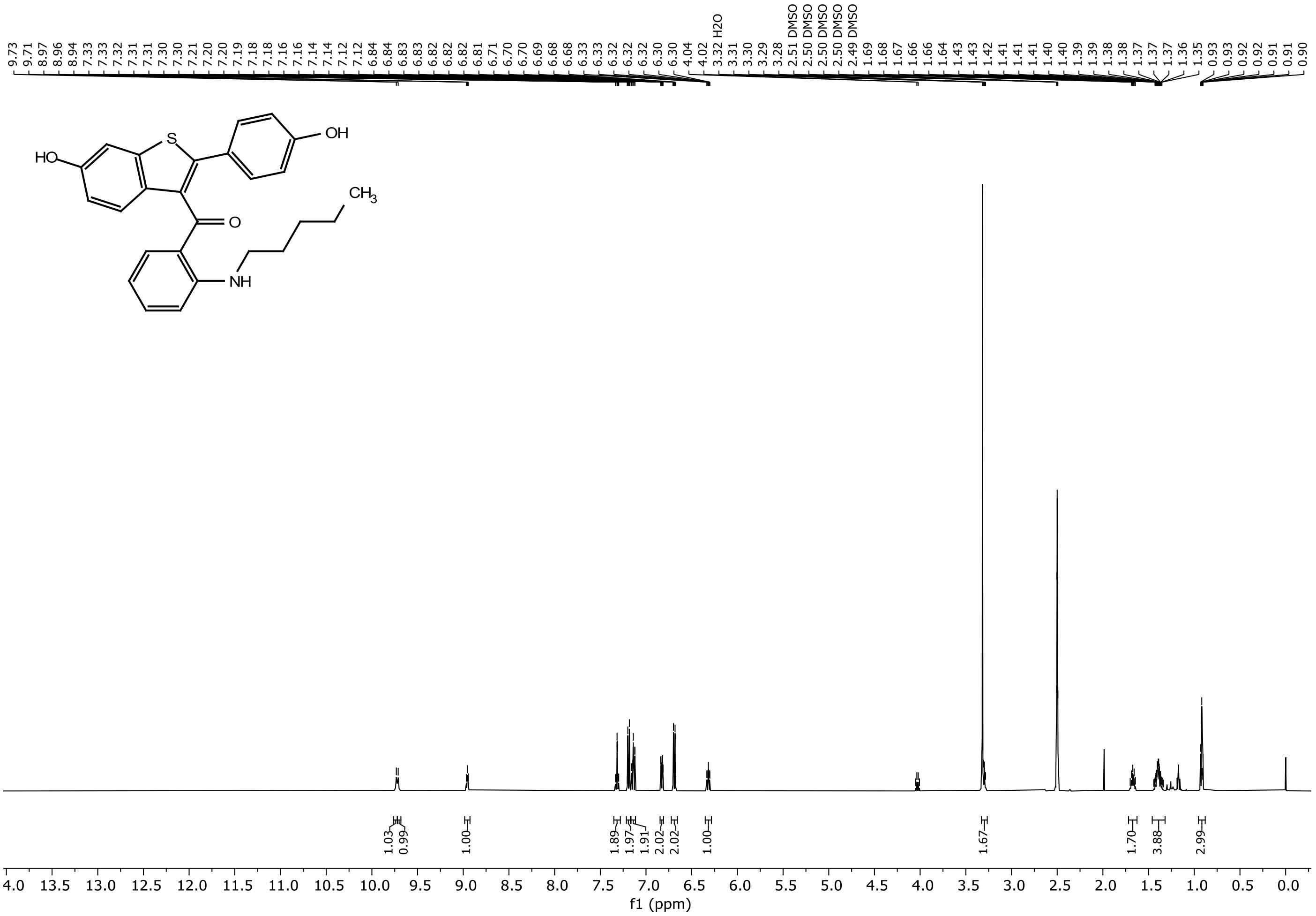

^{13}C NMR (126 MHz, C_2D_6OS) for compound **11f**

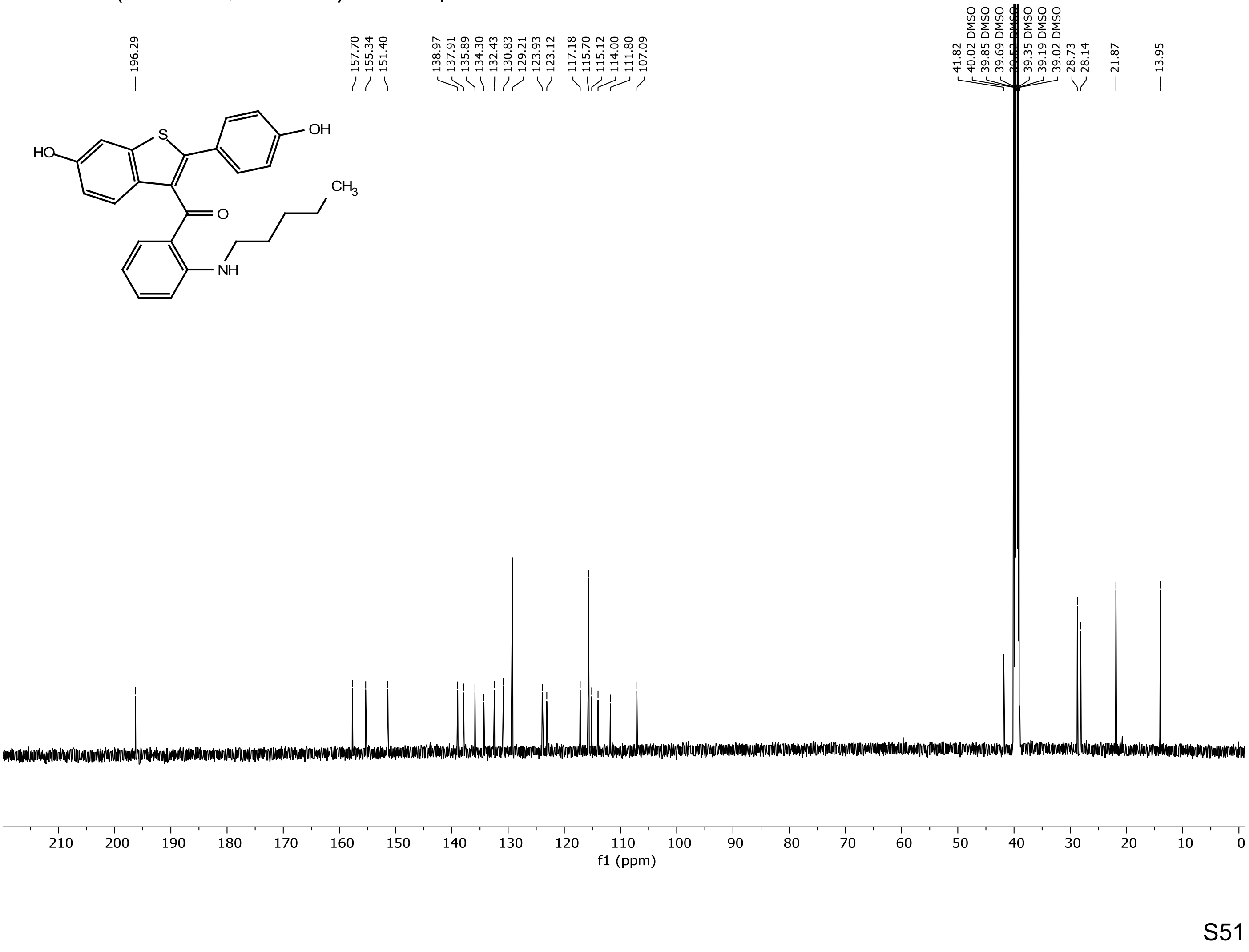

^{1}H NMR (500 MHz, C_2D_6OS) for compound **11g**

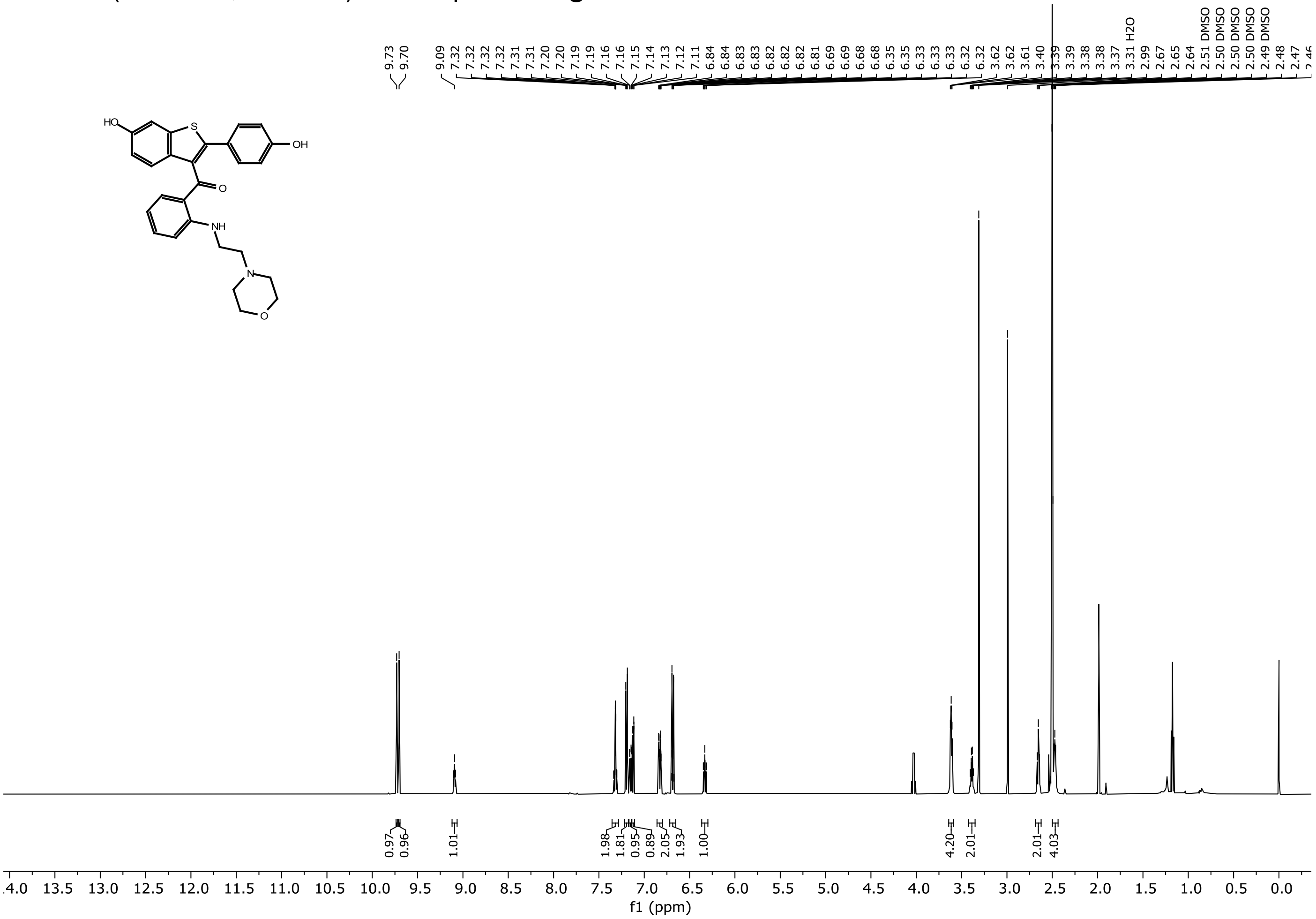

^{13}C NMR (126 MHz, C_2D_6OS) for compound **11g**

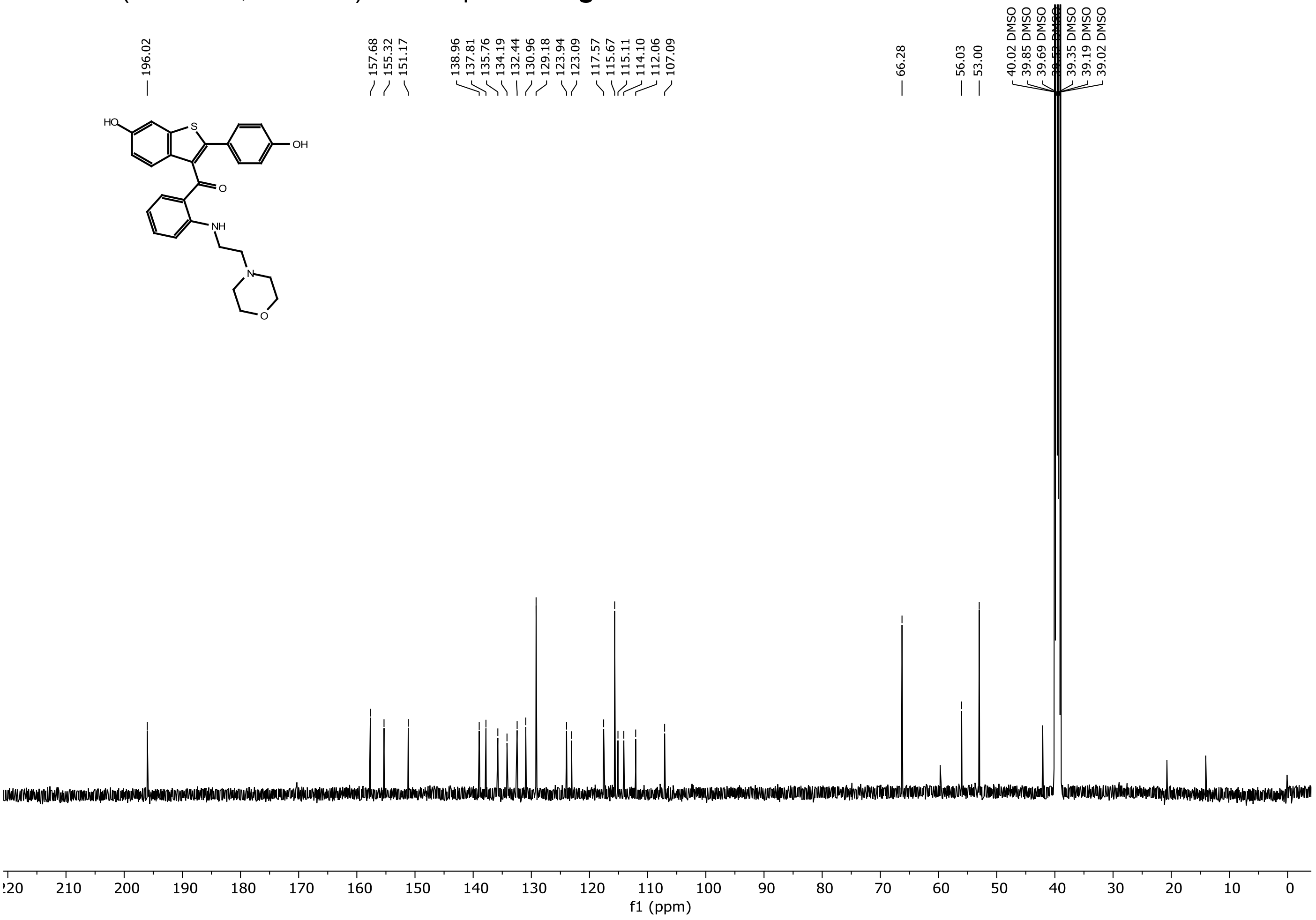

^{1}H NMR (500 MHz, C_2D_6OS) for compound **12a**

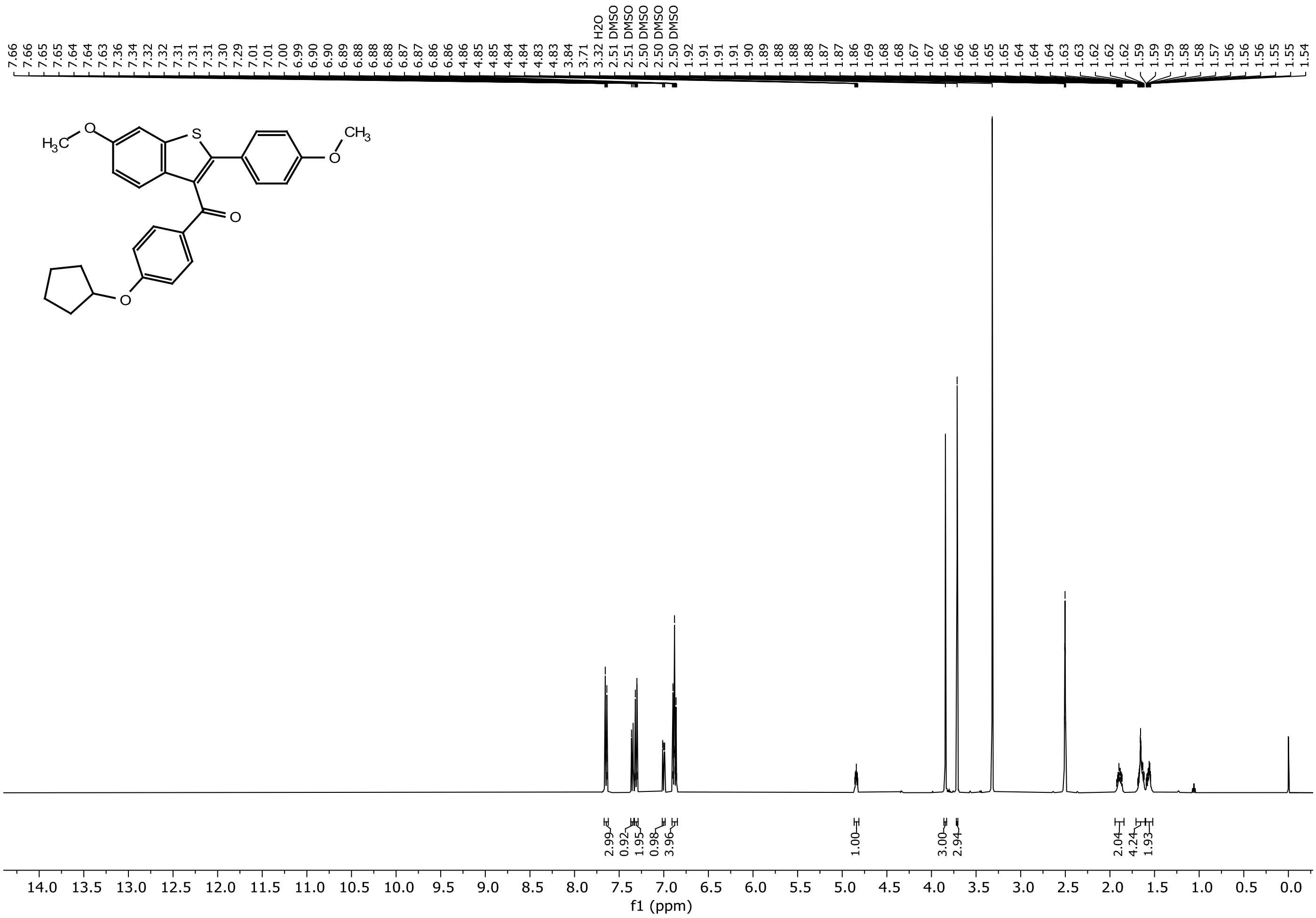

^{13}C NMR (126 MHz, C_2D_6OS) for compound **12a**

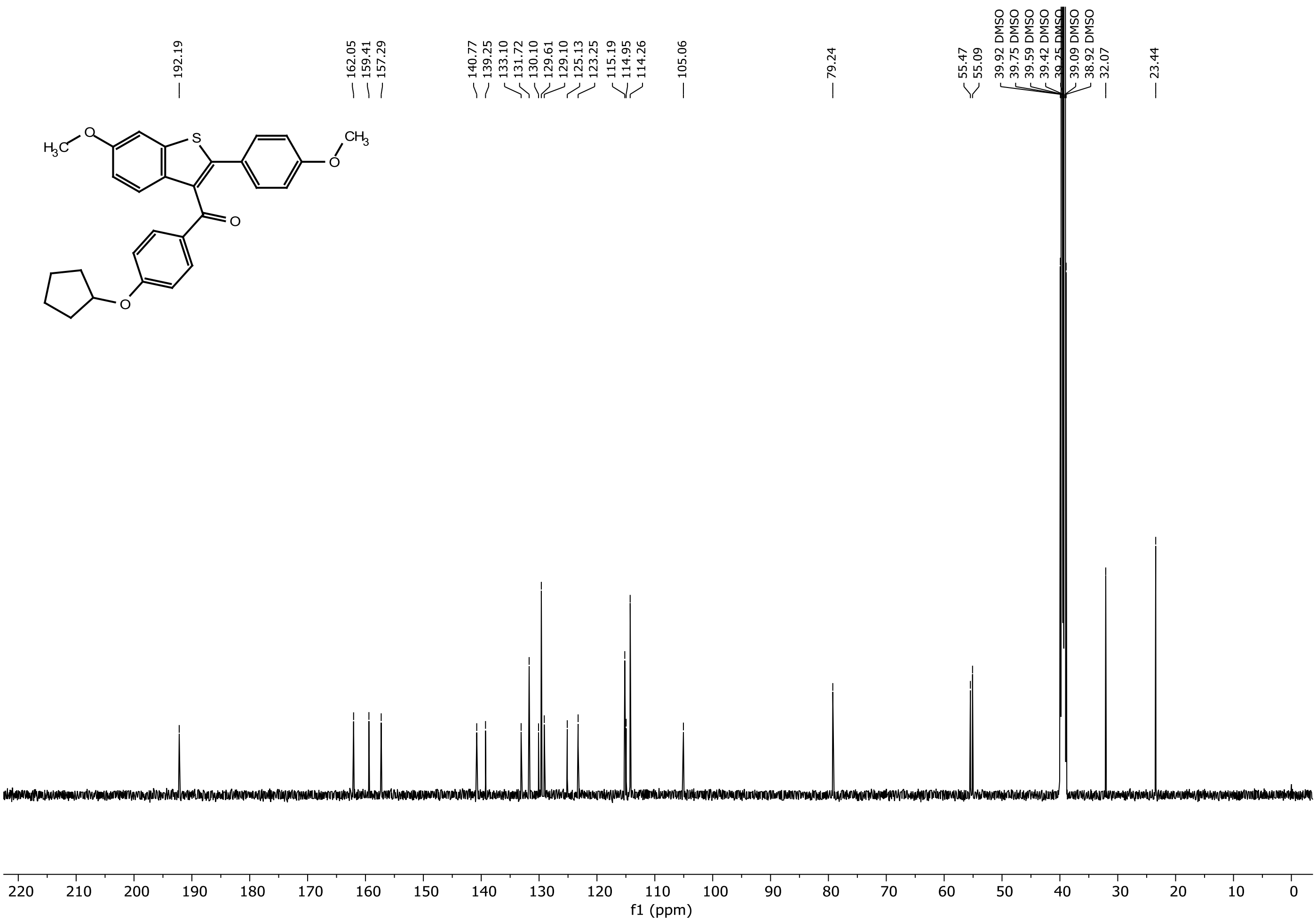

^{1}H NMR (600 MHz, C_2D_6OS) for compound **12b**

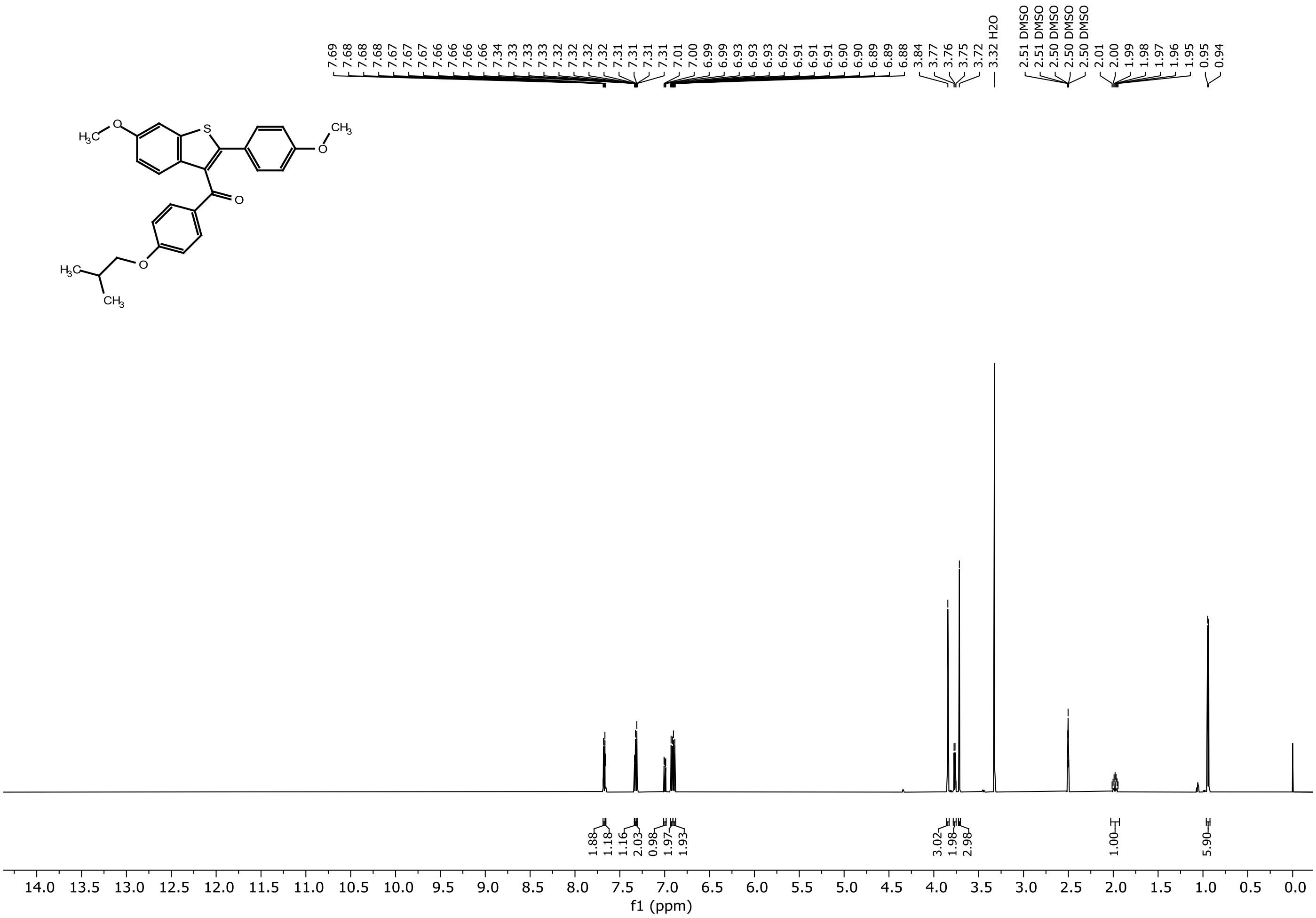

^{13}C NMR (151 MHz, C_2D_6OS) for compound **12b**

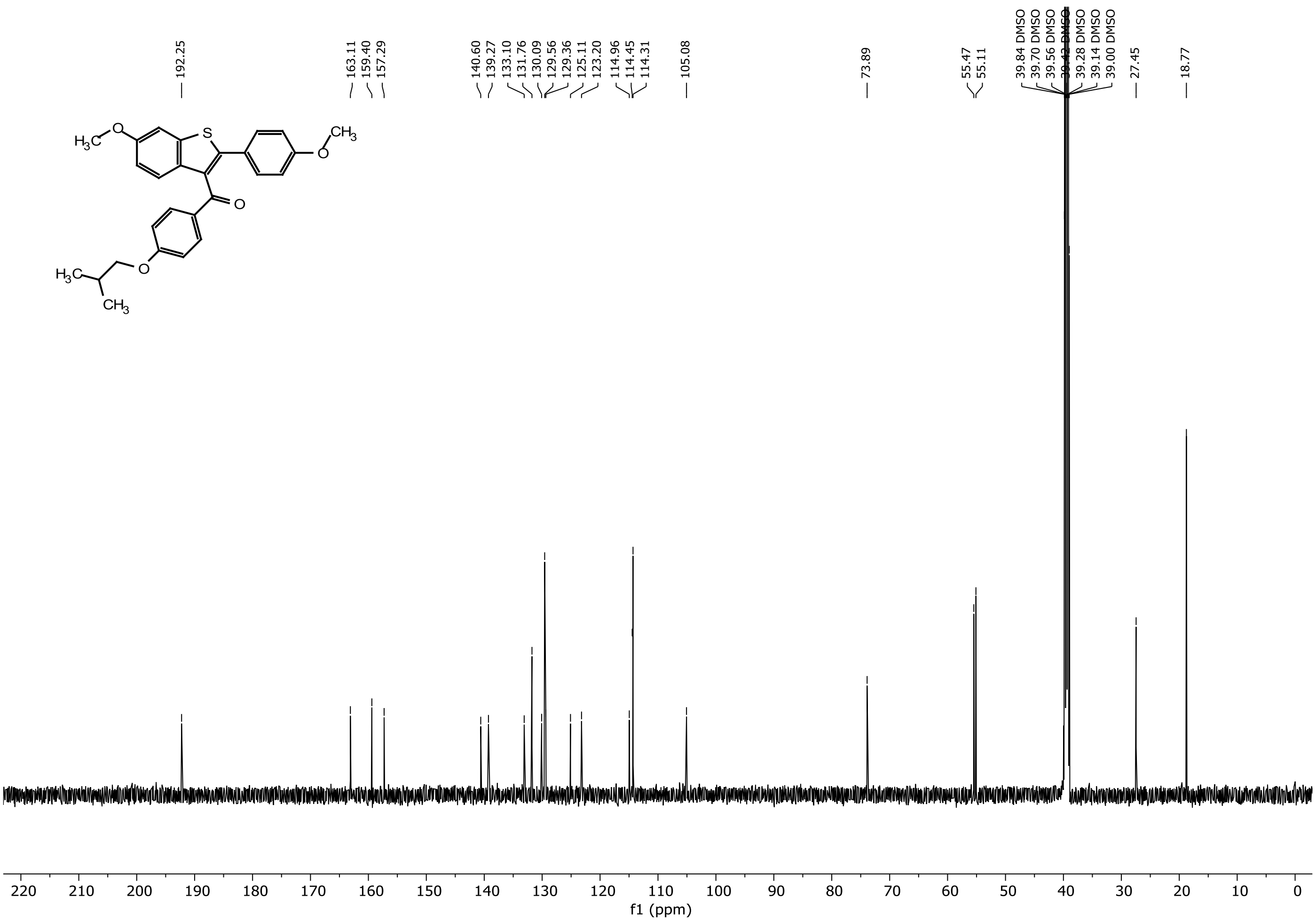

^{1}H NMR (500 MHz, C_2D_6OS) for compound **12c**

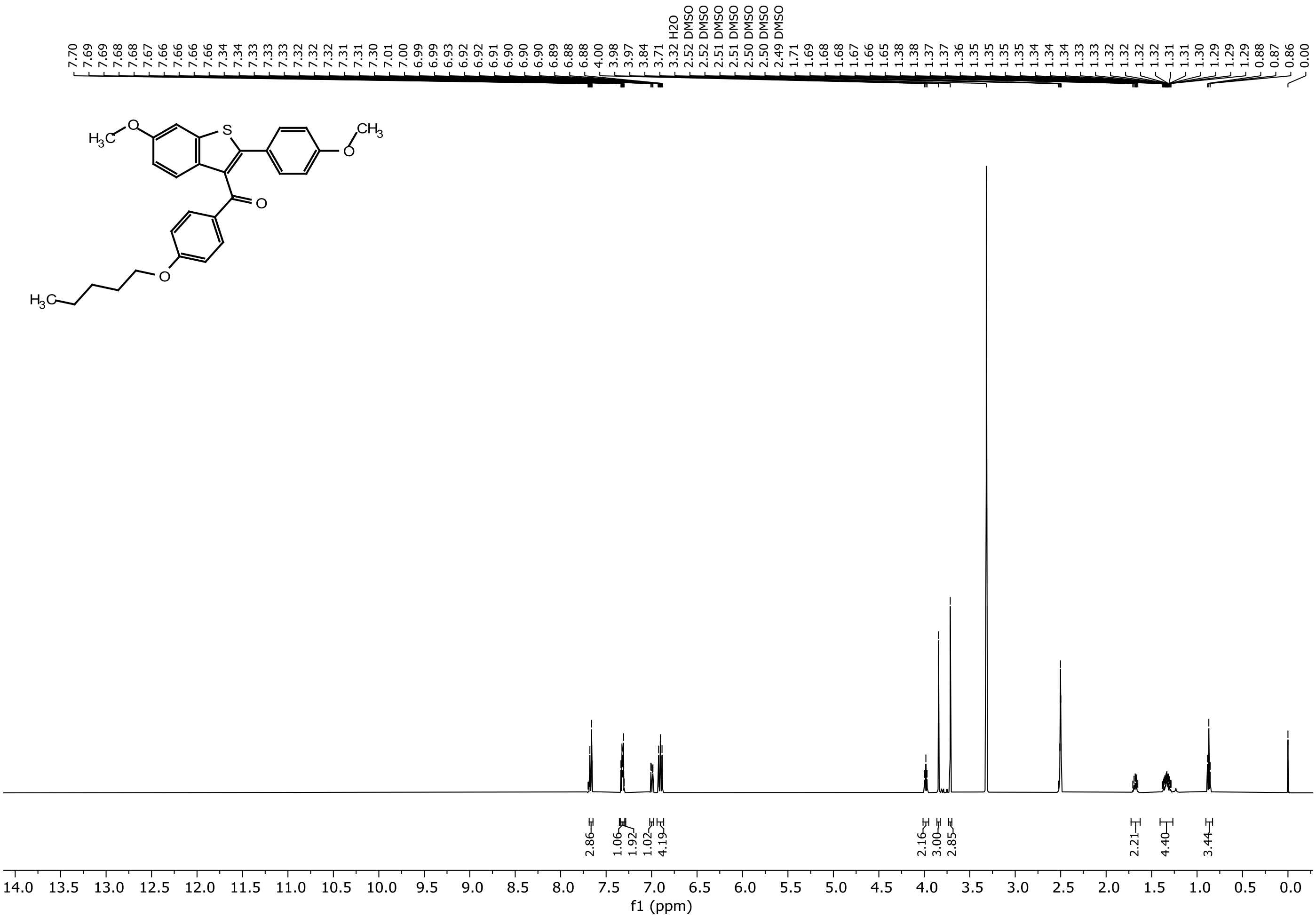

^{13}C NMR (126 MHz, C_2D_6OS) for compound **12c**

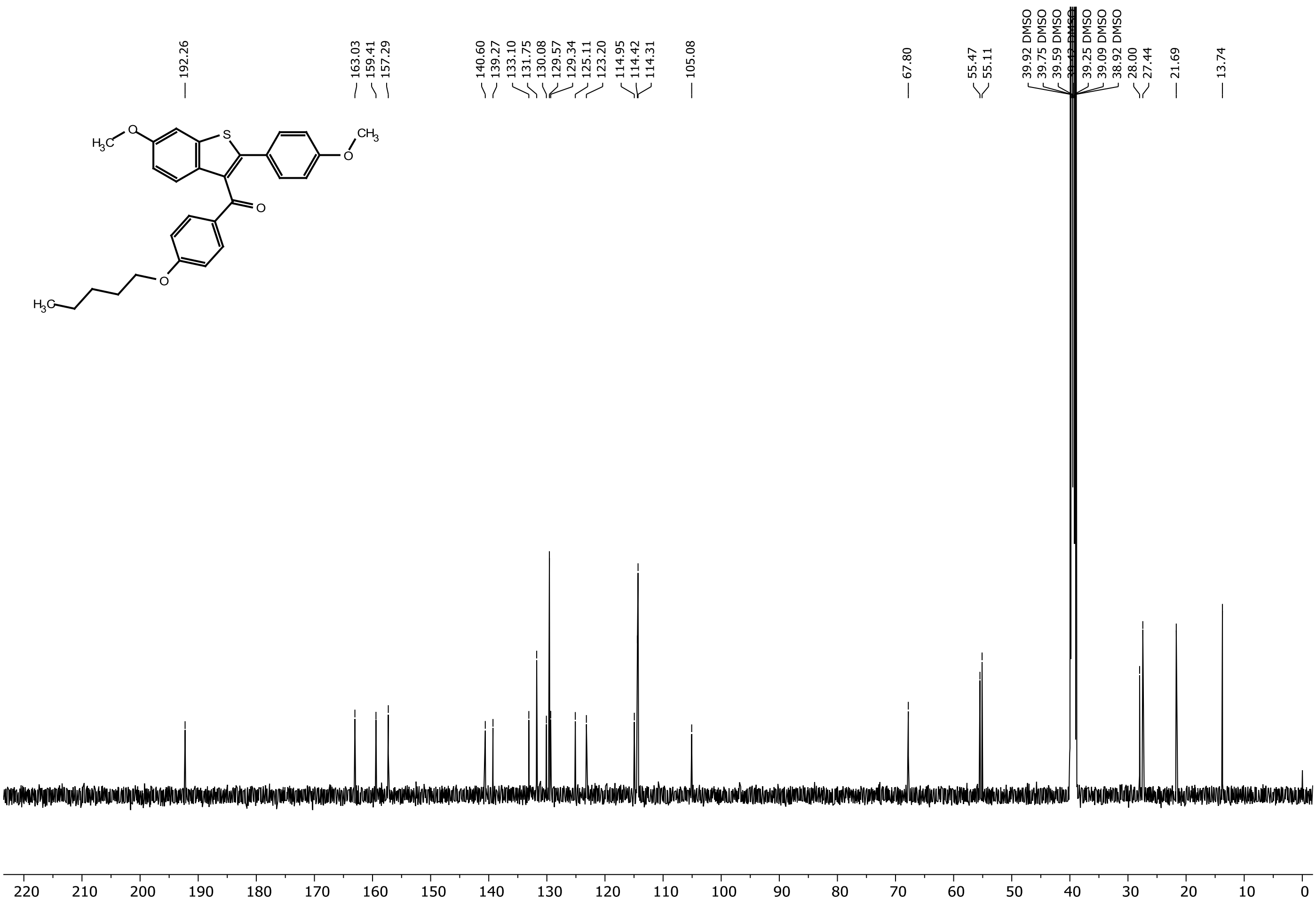

^{1}H NMR (500 MHz, C_2D_6OS) for compound **12d**

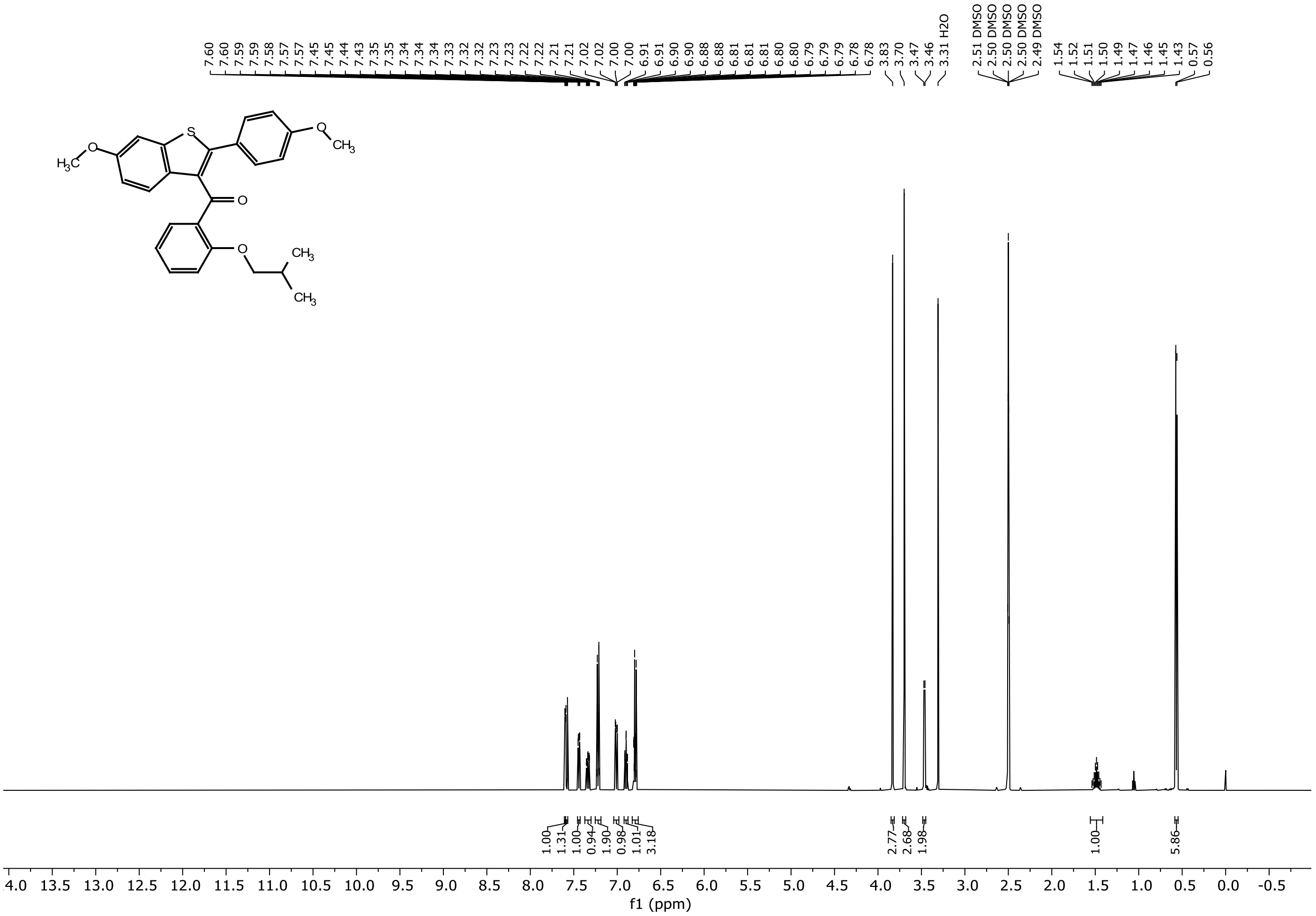

^{13}C NMR (126 MHz, C_2D_6OS) for compound **12d**

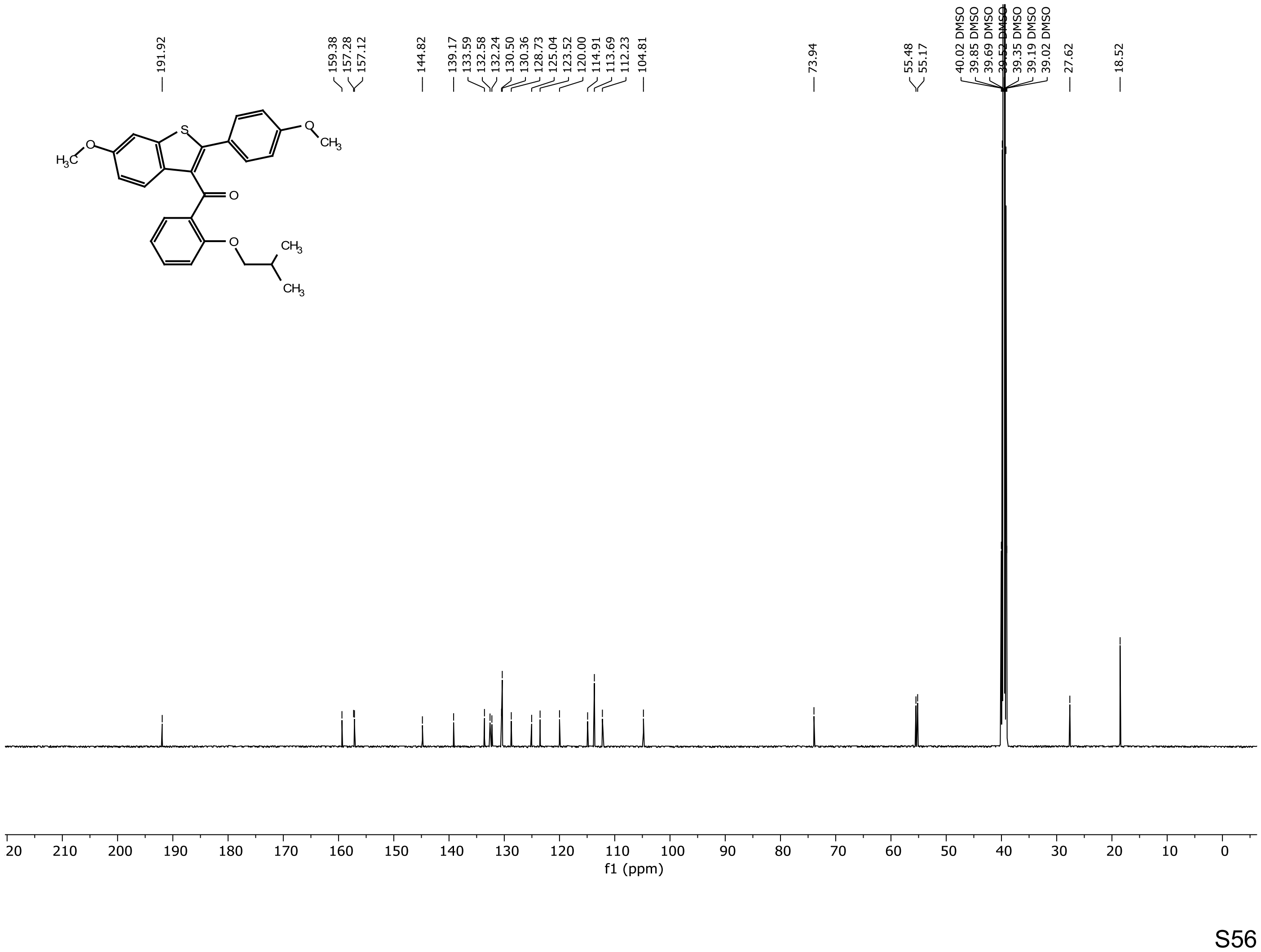

^{1}H NMR (500 MHz, C_2D_6OS) for compound **13a**

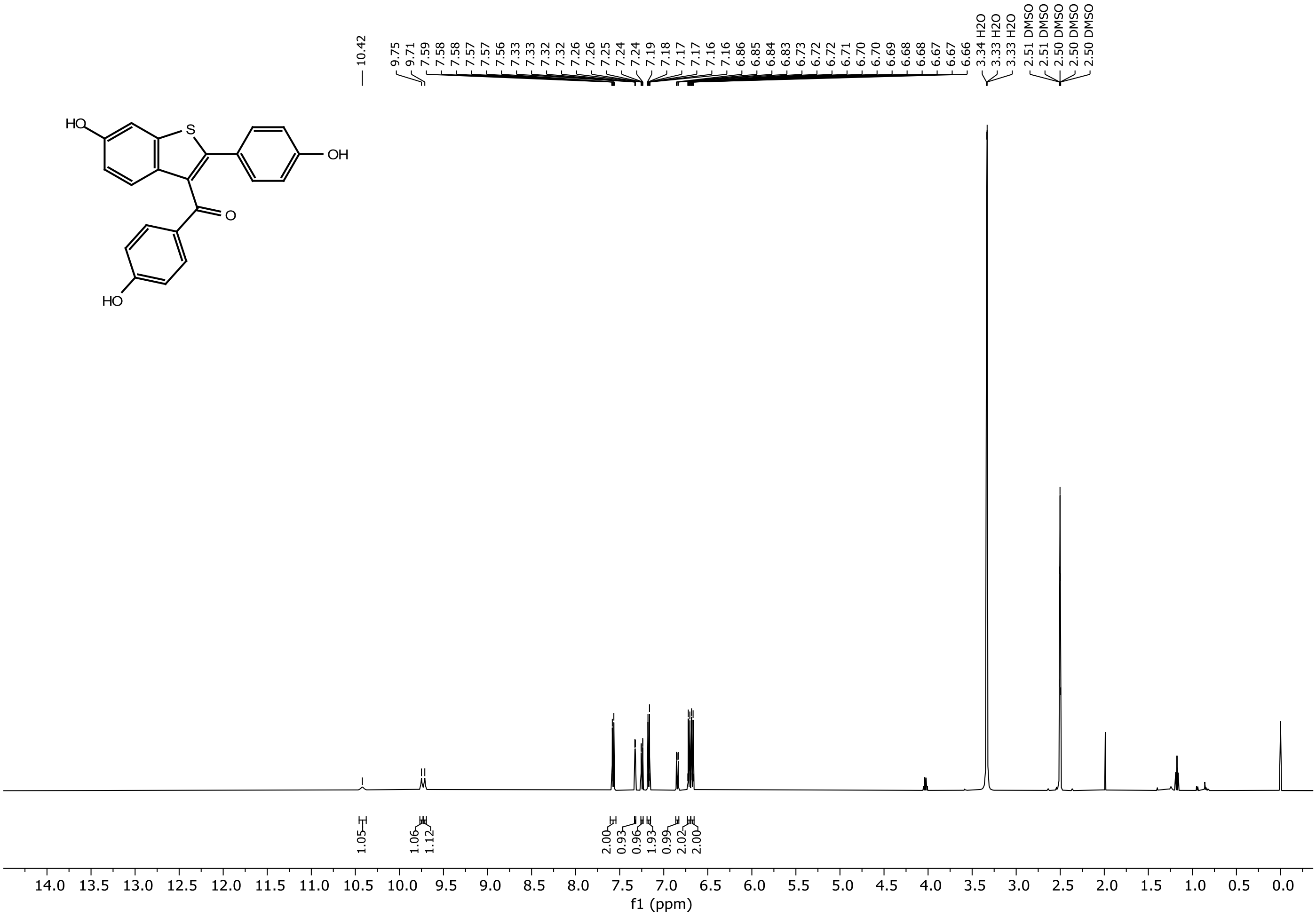

^{13}C NMR (126 MHz, C_2D_6OS) for compound **13a**

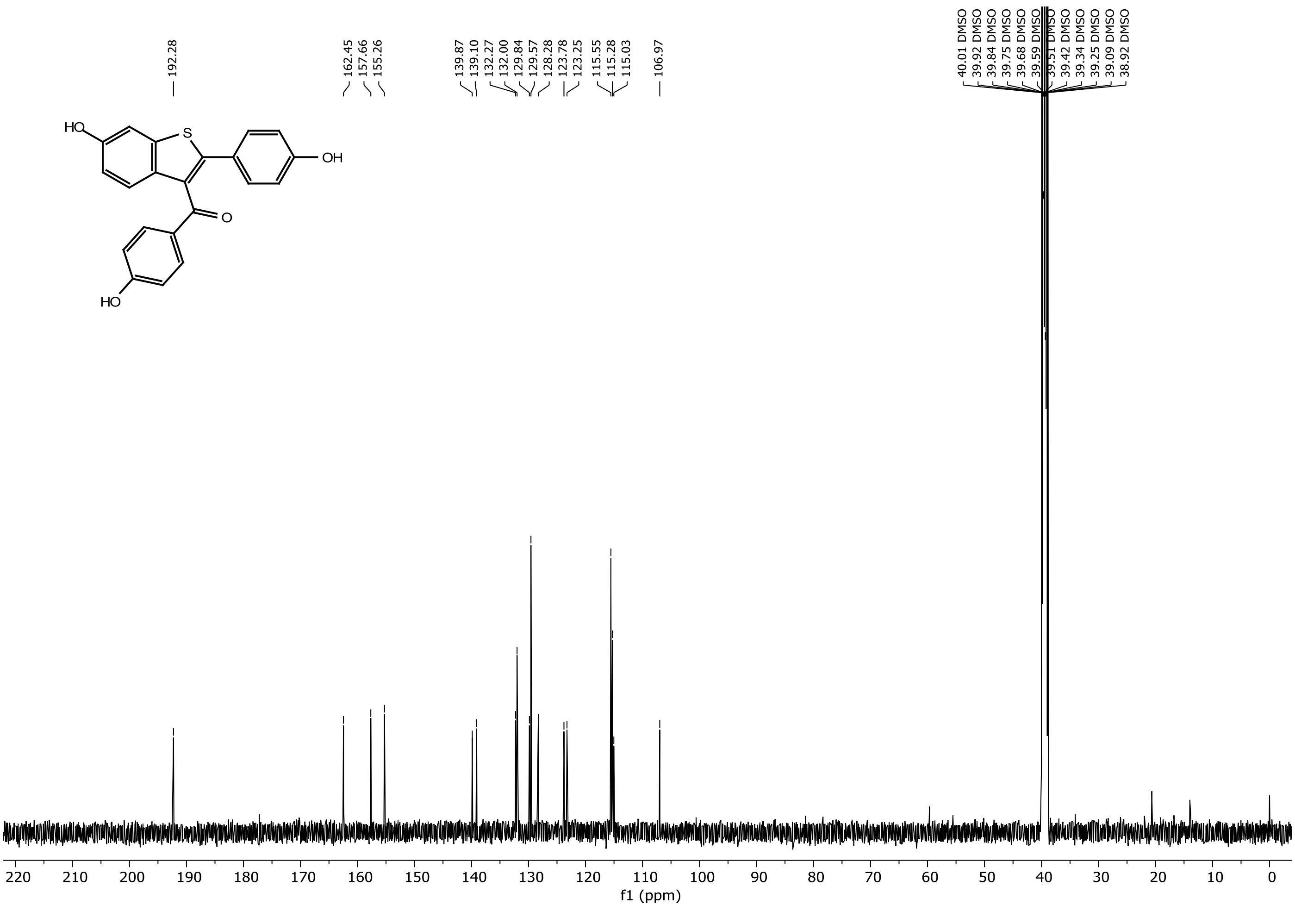

^{1}H NMR (500 MHz, C_2D_6OS) for compound **13b**

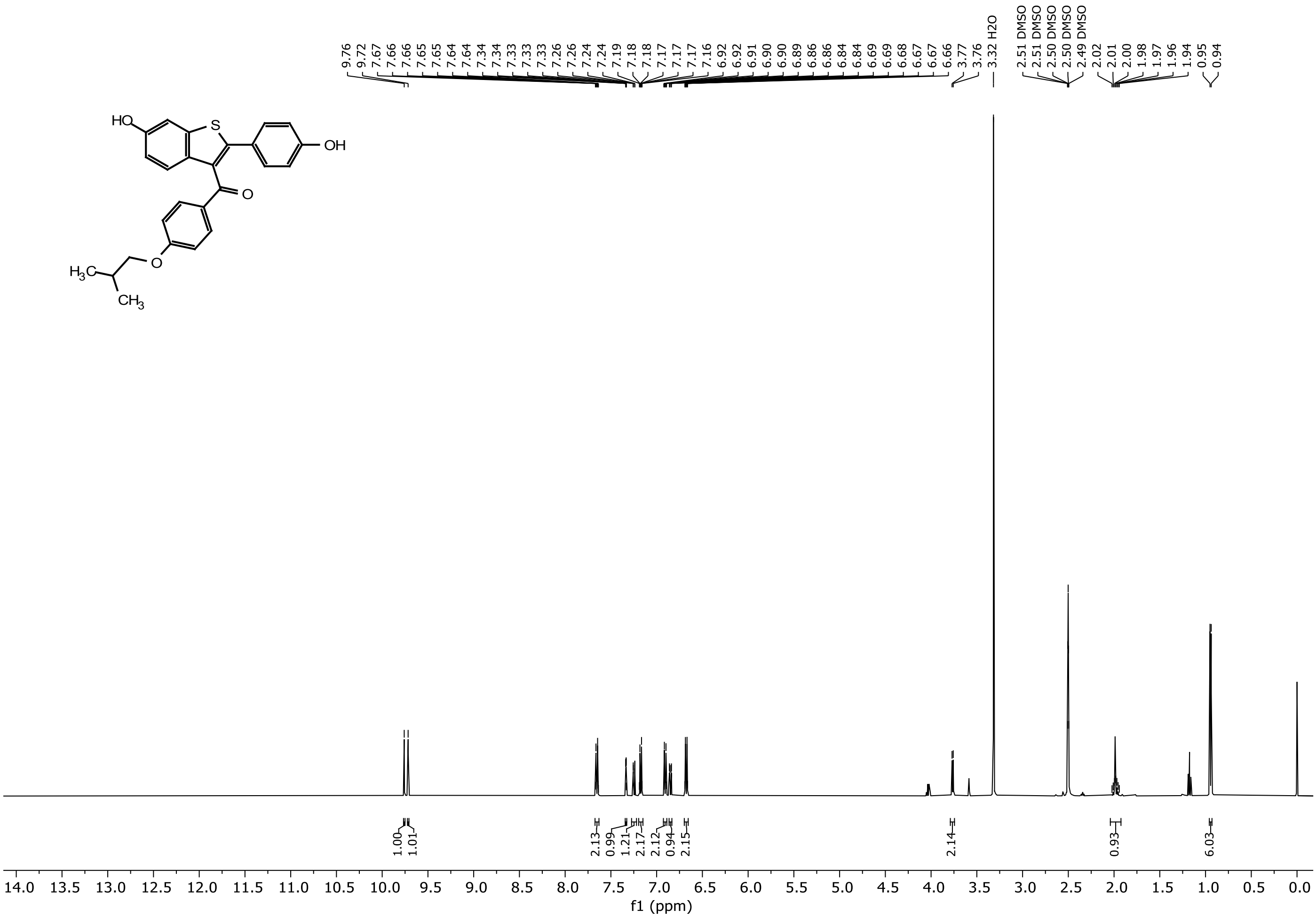

^{13}C NMR (126 MHz, C_2D_6OS) for compound **13b**

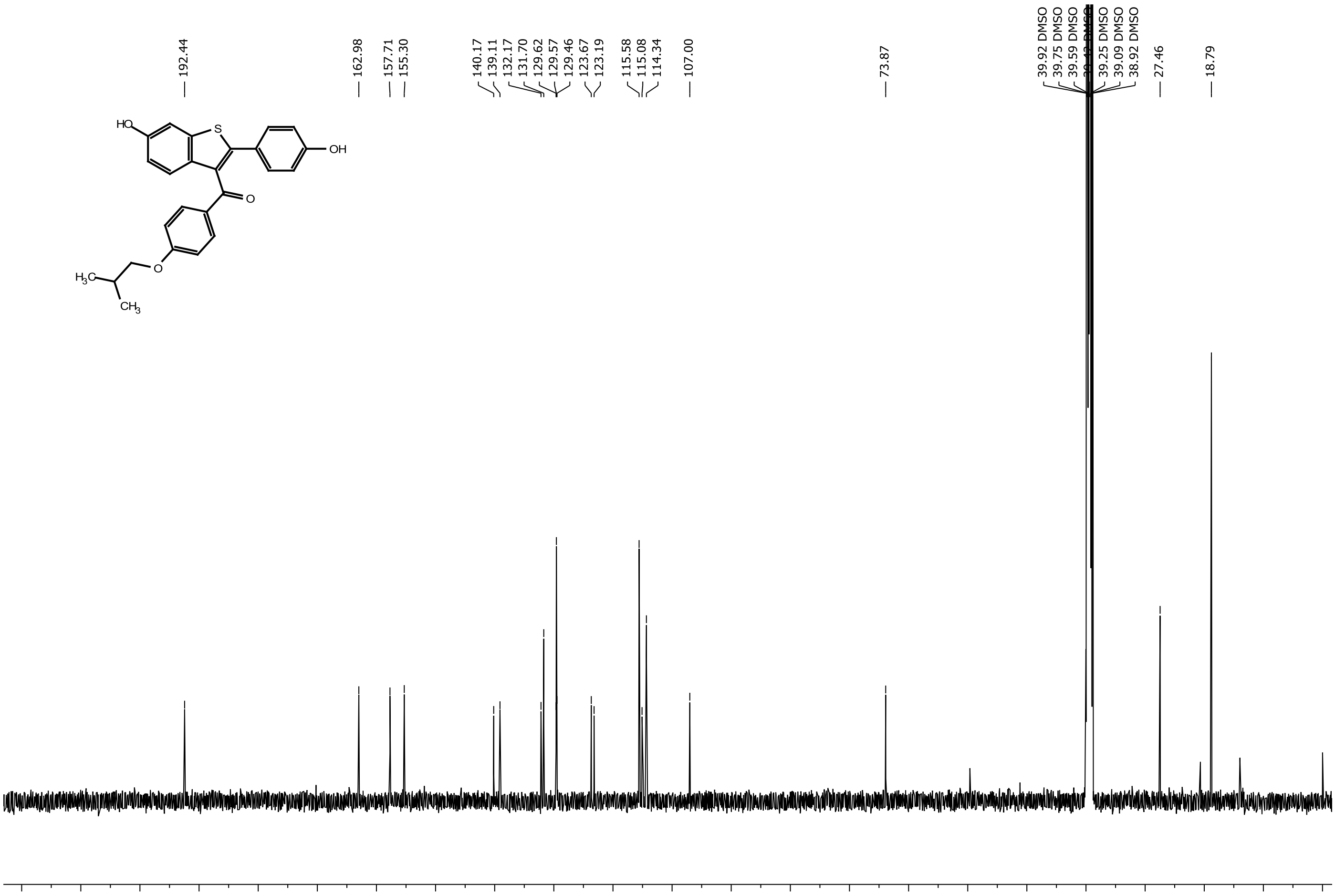

^{1}H NMR (600 MHz, C_2D_6OS) for compound **13c**

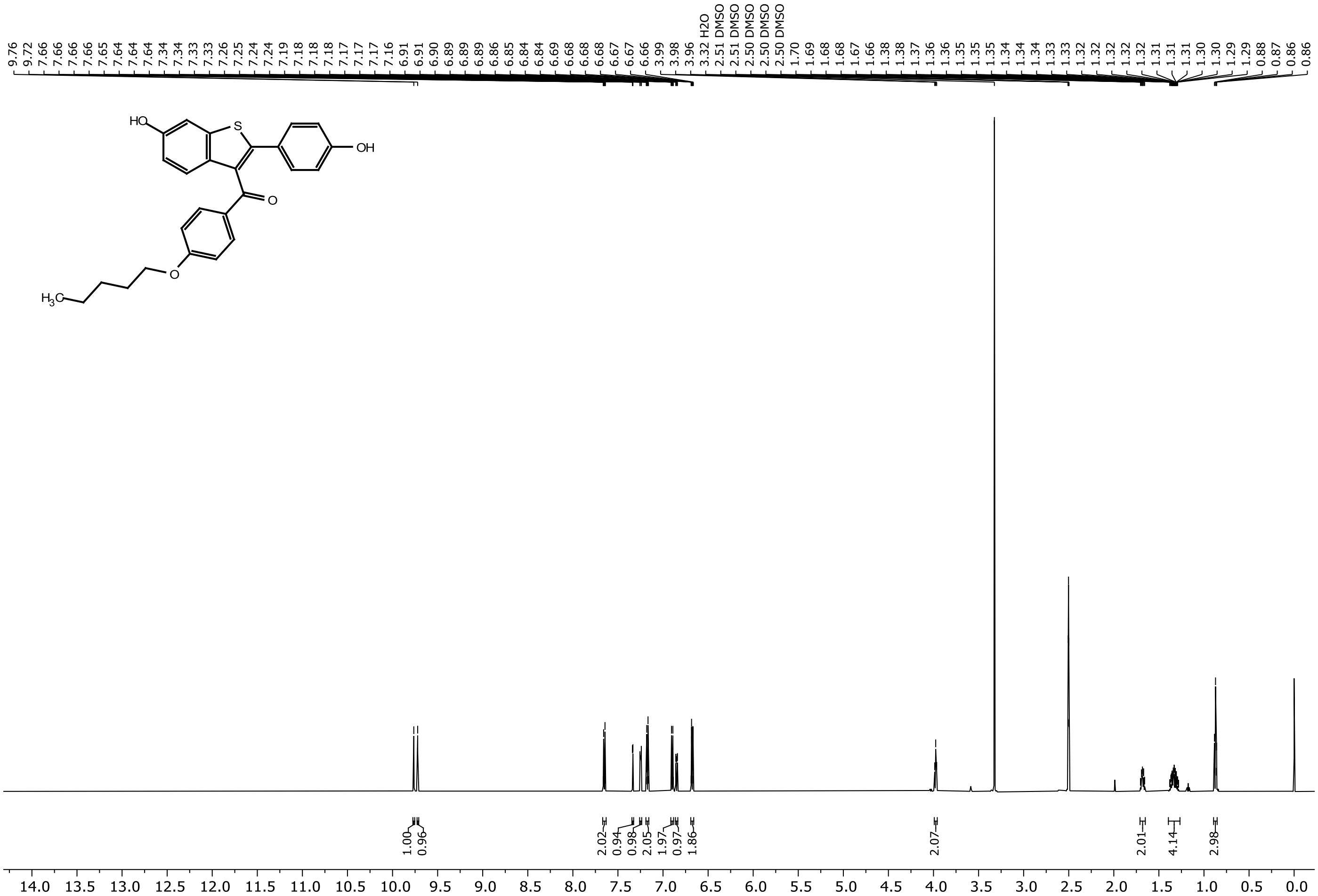

^{13}C NMR (126 MHz, C_2D_6OS) for compound **13c**

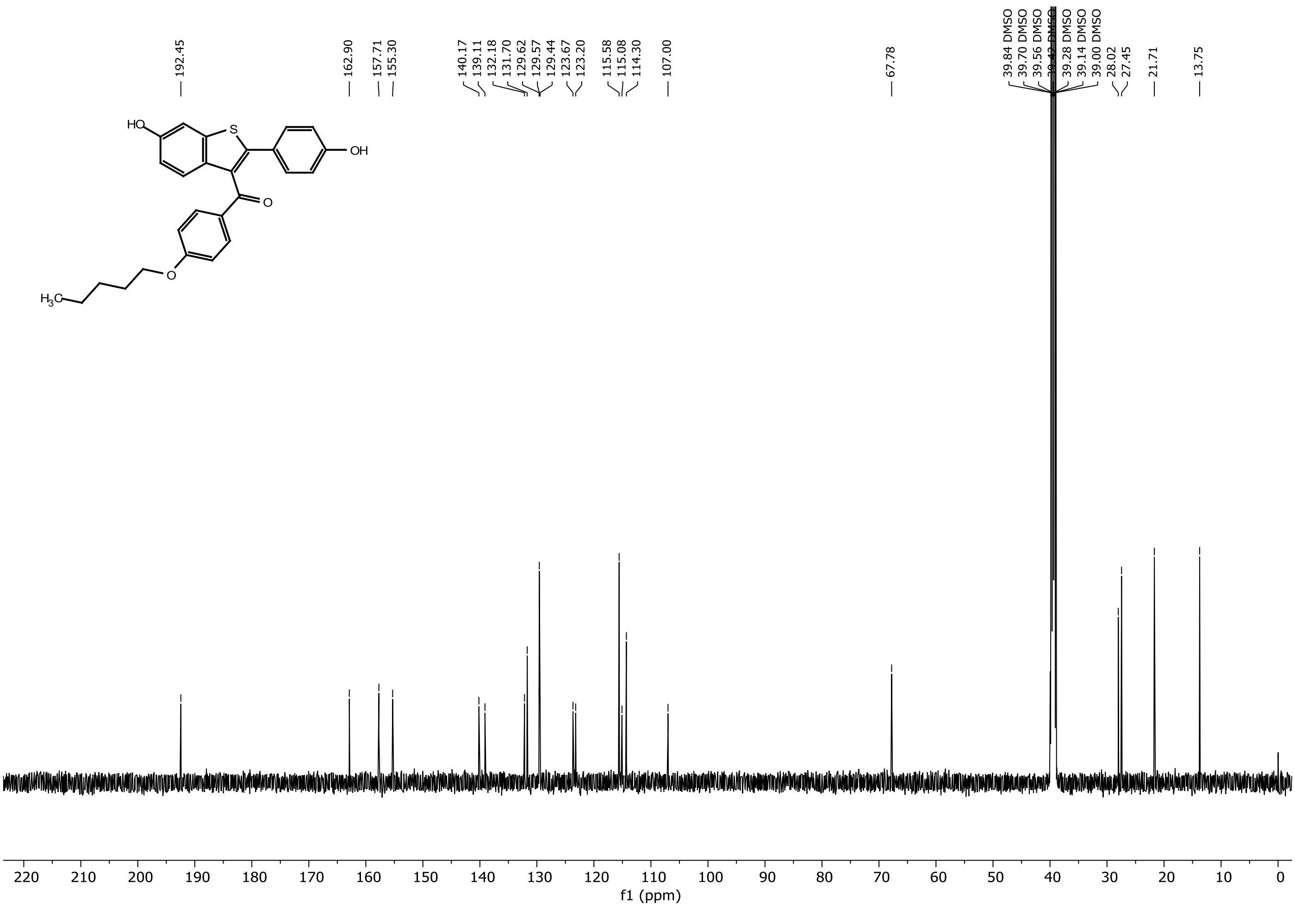

^{1}H NMR (500 MHz, C_2D_6OS) for compound **13d**

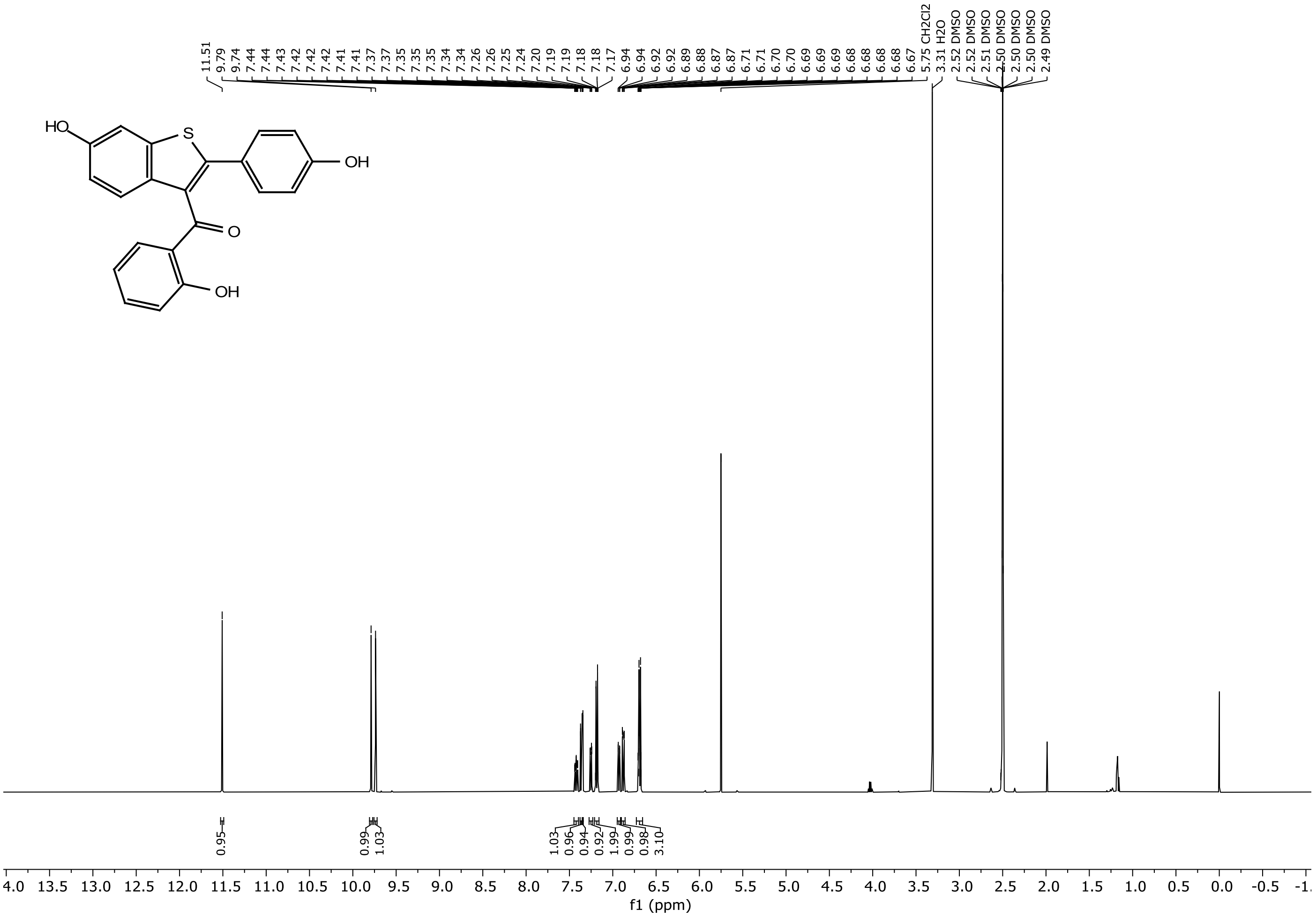

^{13}C NMR (126 MHz, C_2D_6OS) for compound **13d**

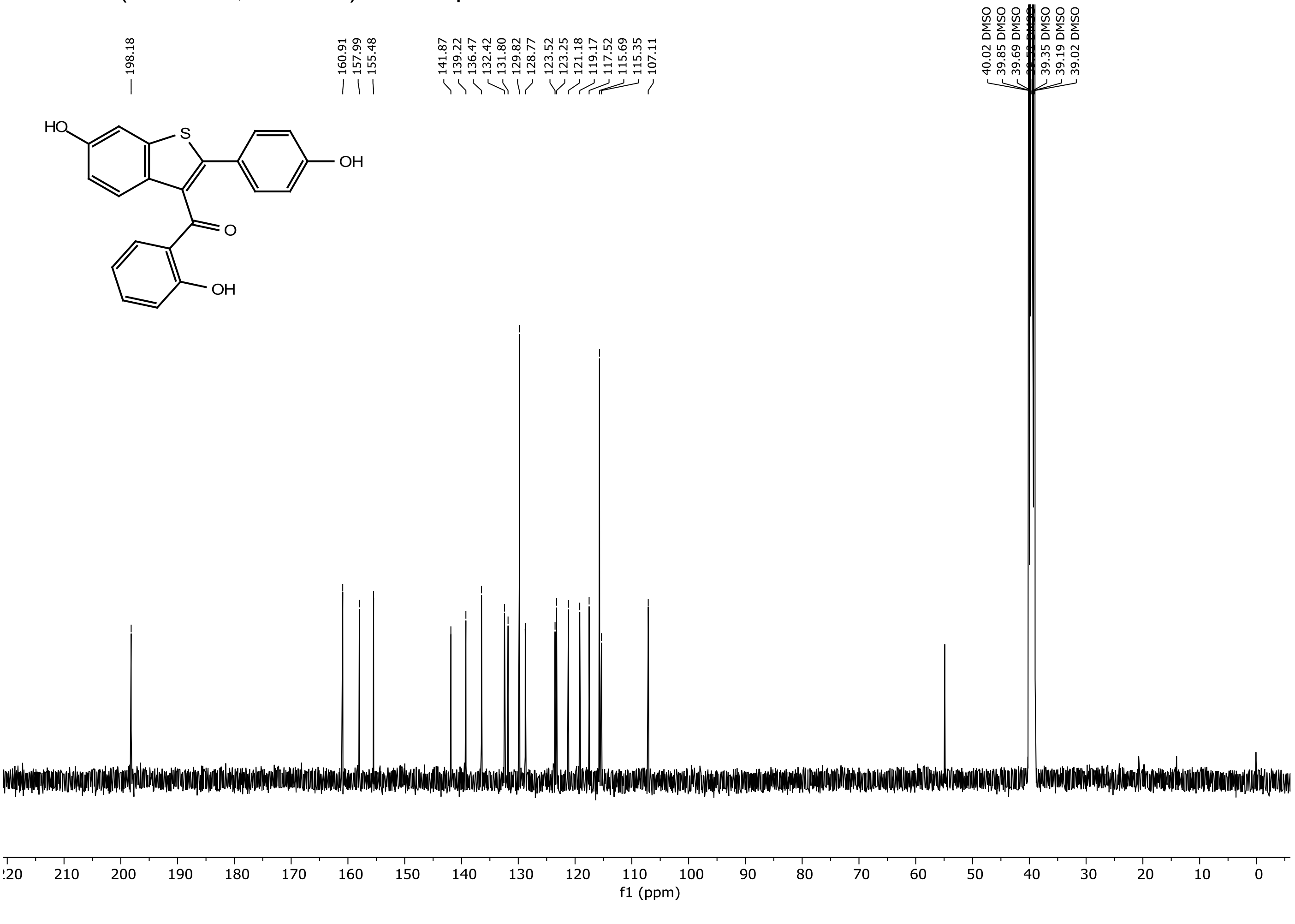

^{1}H NMR (500 MHz, C_2D_6OS) for compound **13e**

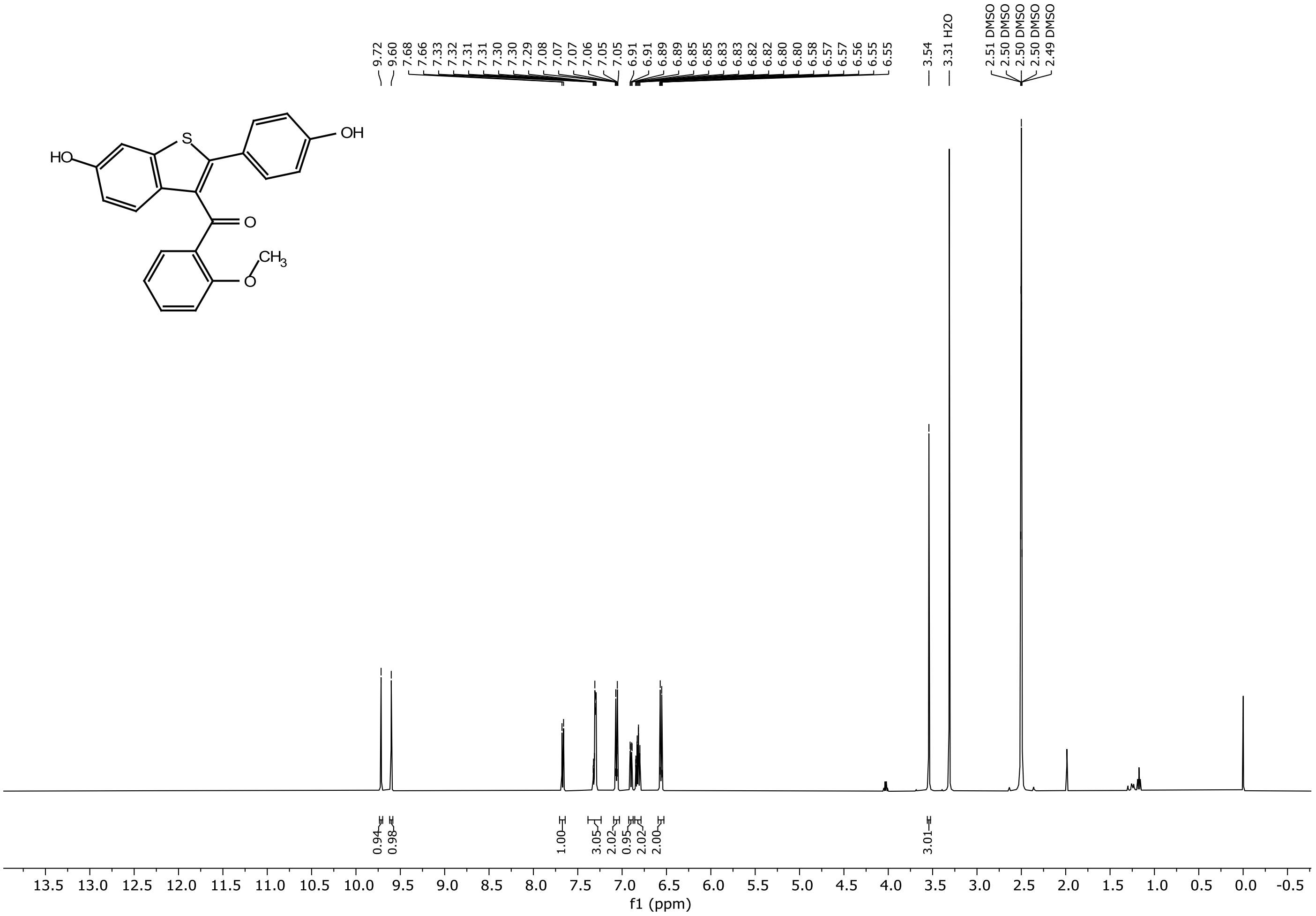

^{13}C NMR (126 MHz, C_2D_6OS) for compound **13e**

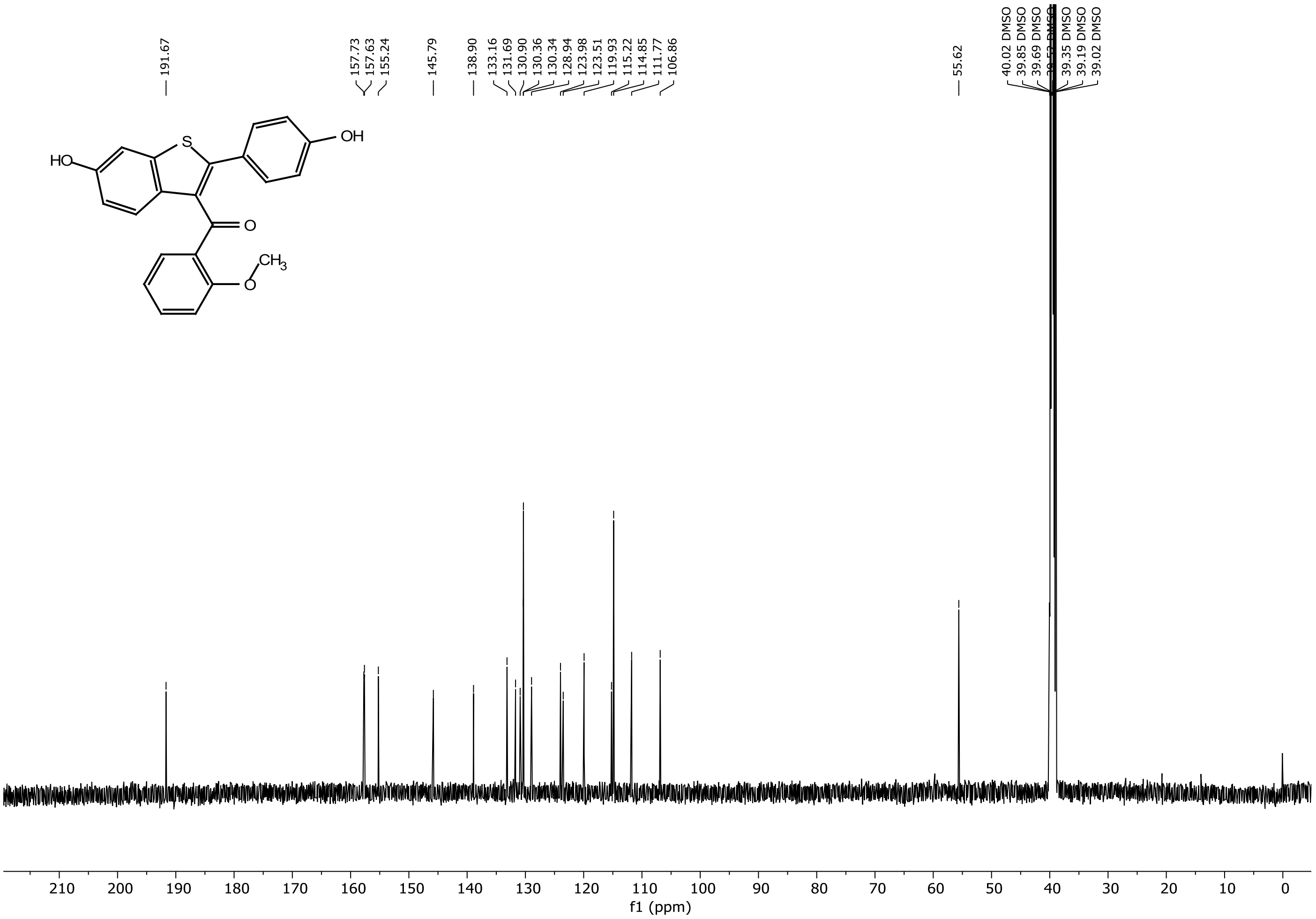

^{1}H NMR (500 MHz, C_2D_6OS) for compound **13f**

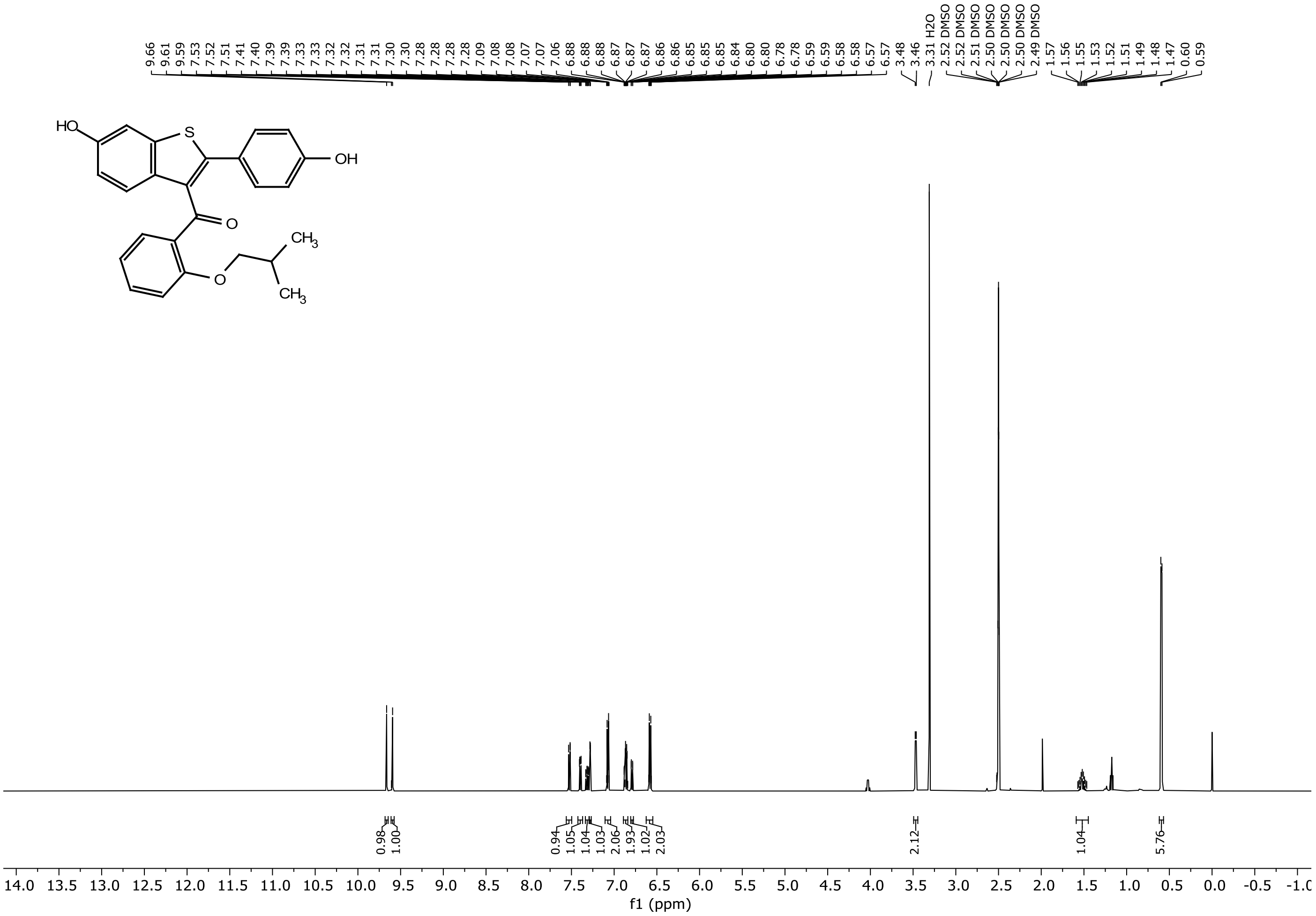

^{13}C NMR (126 MHz, C_2D_6OS) for compound **13f**

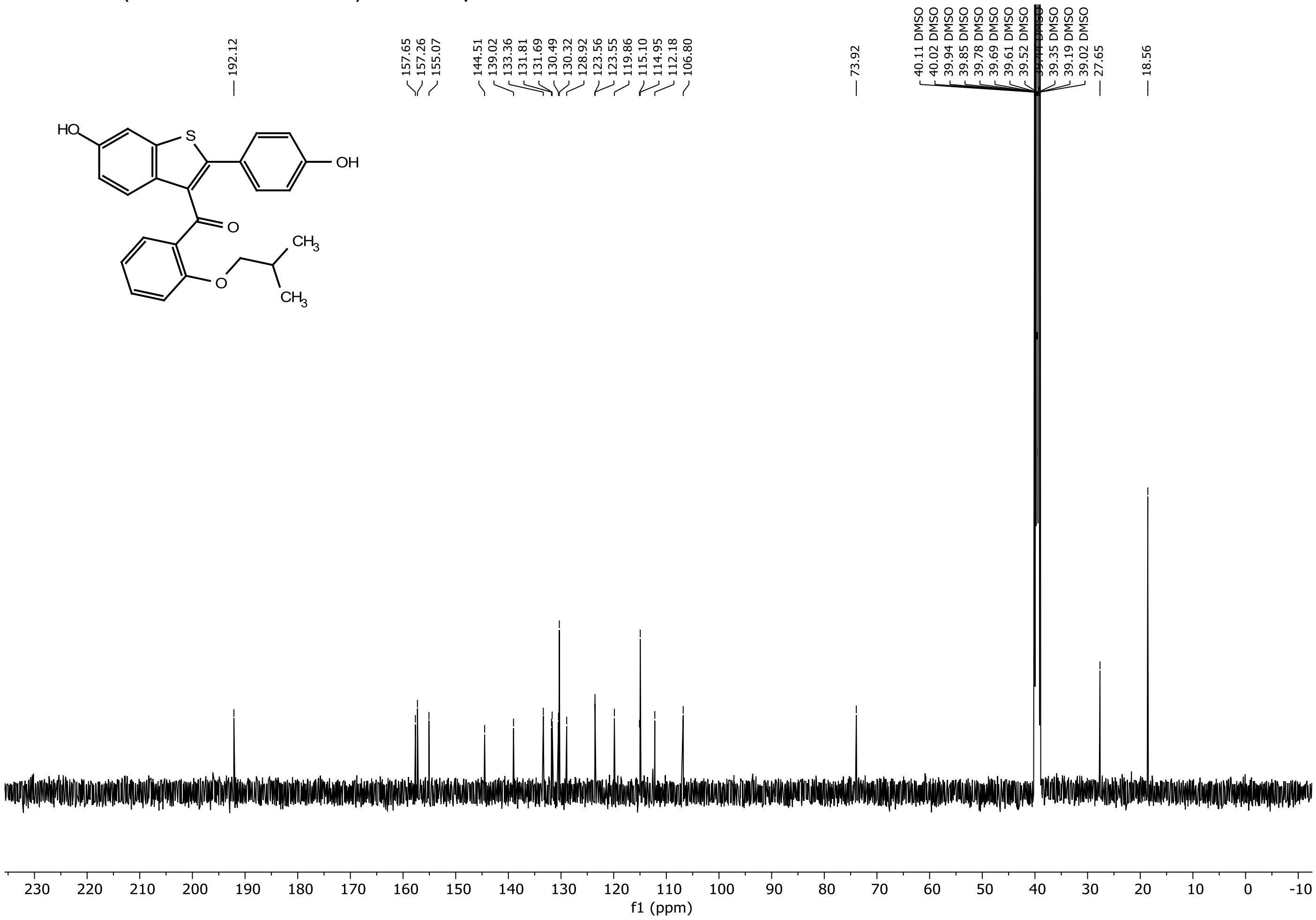

^{1}H NMR (500 MHz, C_2D_6OS) for compound **13g**

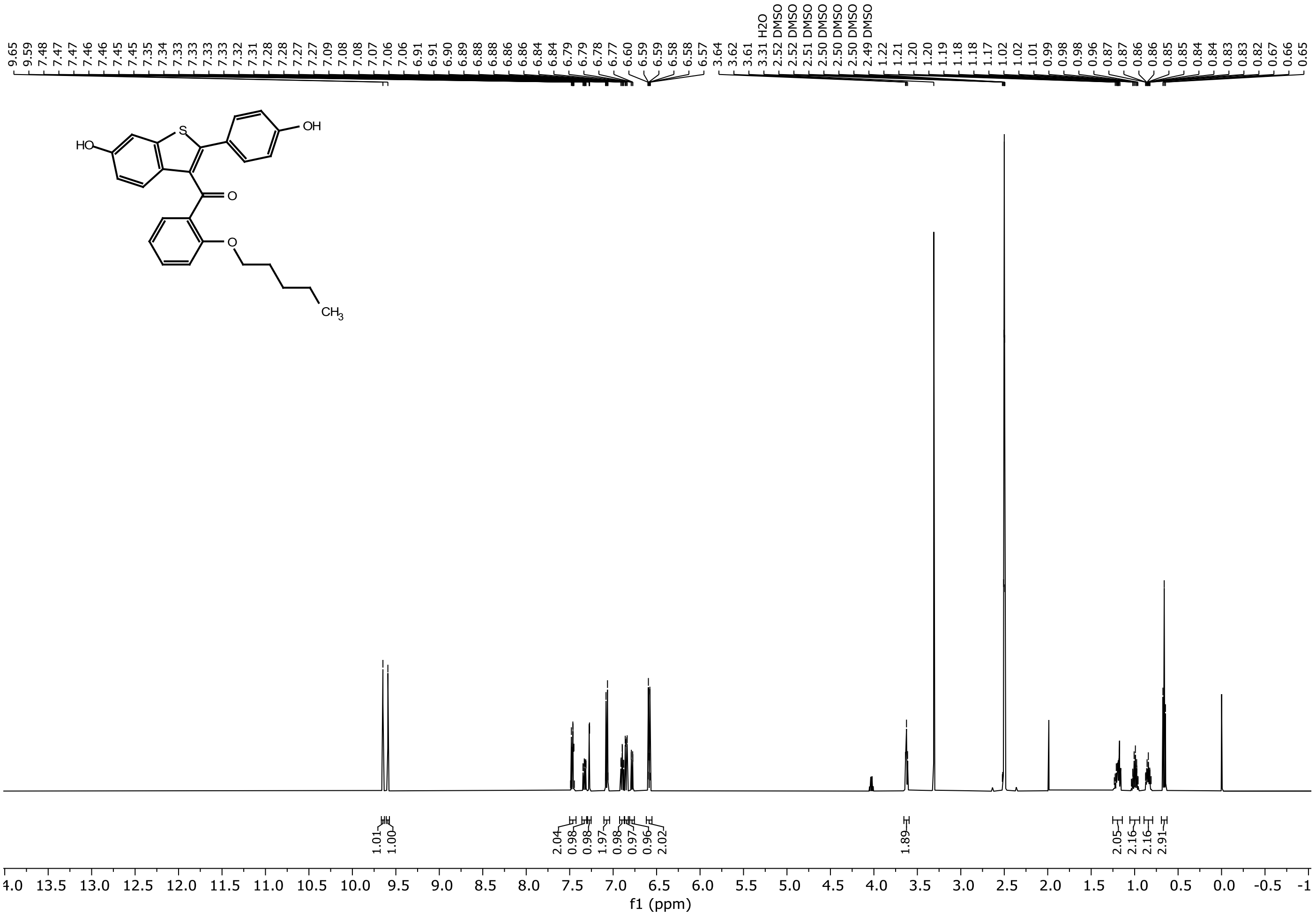

^{13}C NMR (126 MHz, C_2D_6OS) for compound **13g**

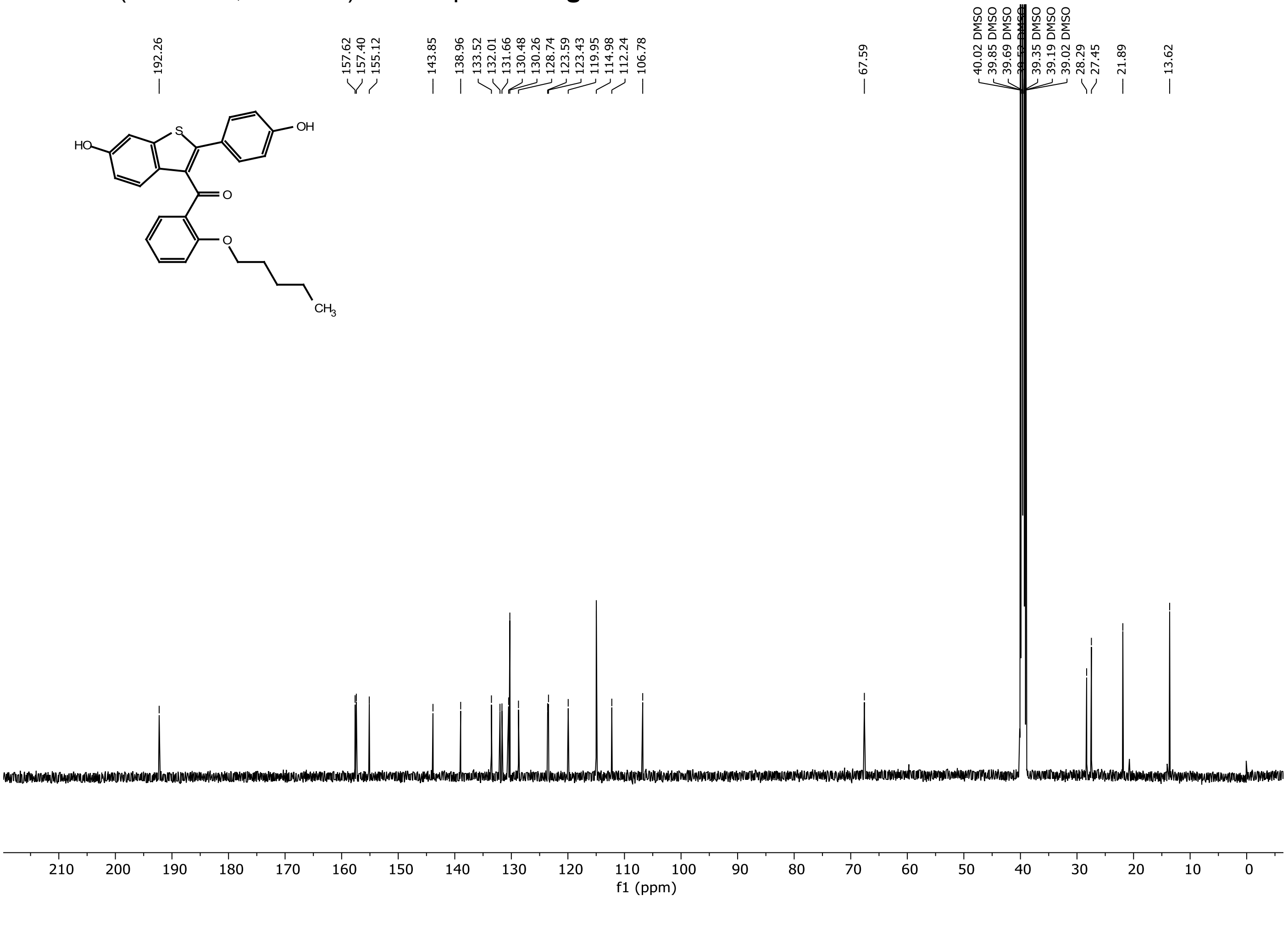

^{1}H NMR (500 MHz, C_2D_6OS) for compound **14a**

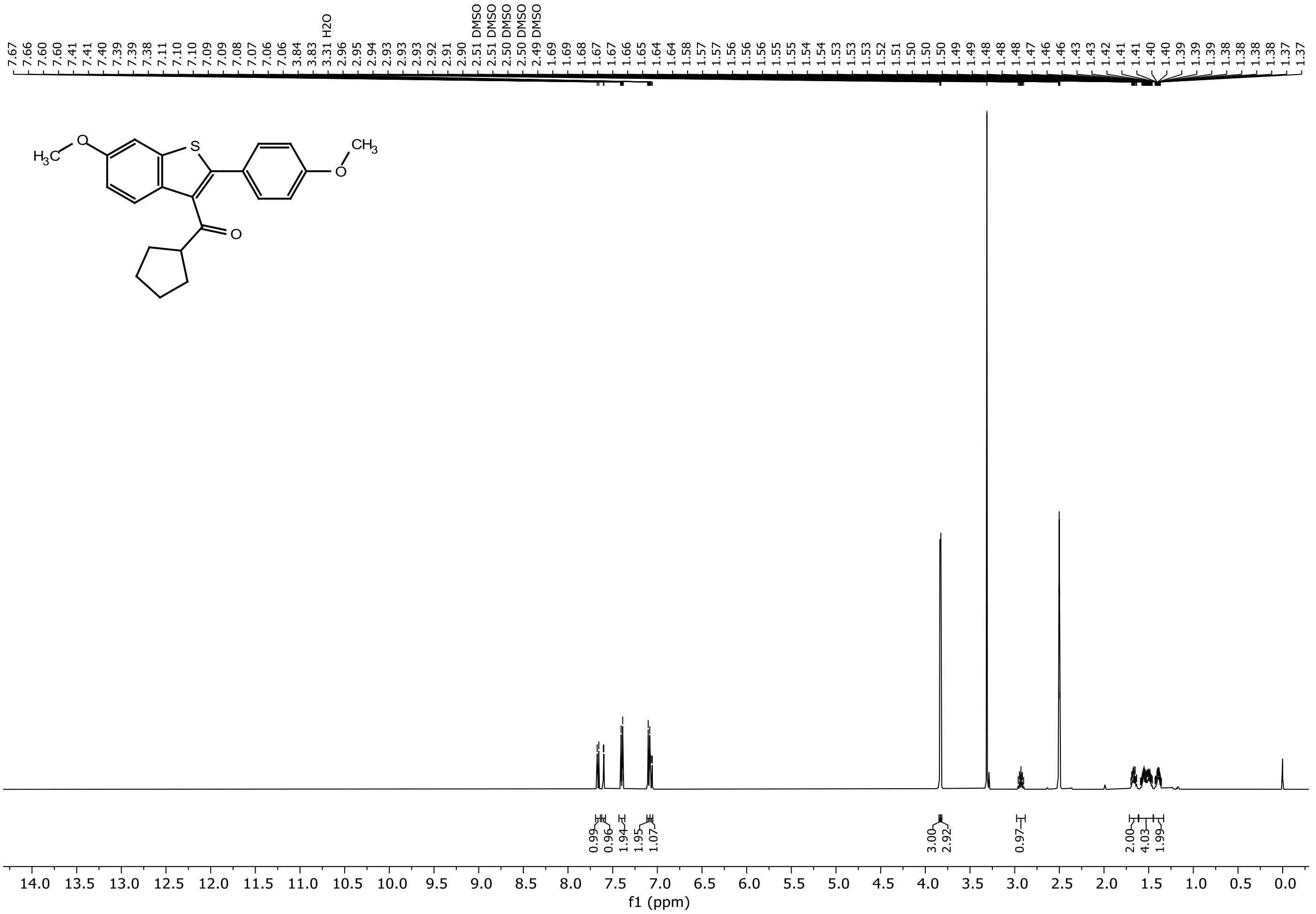

^{13}C NMR (126 MHz, C_2D_6OS) for compound **14a**

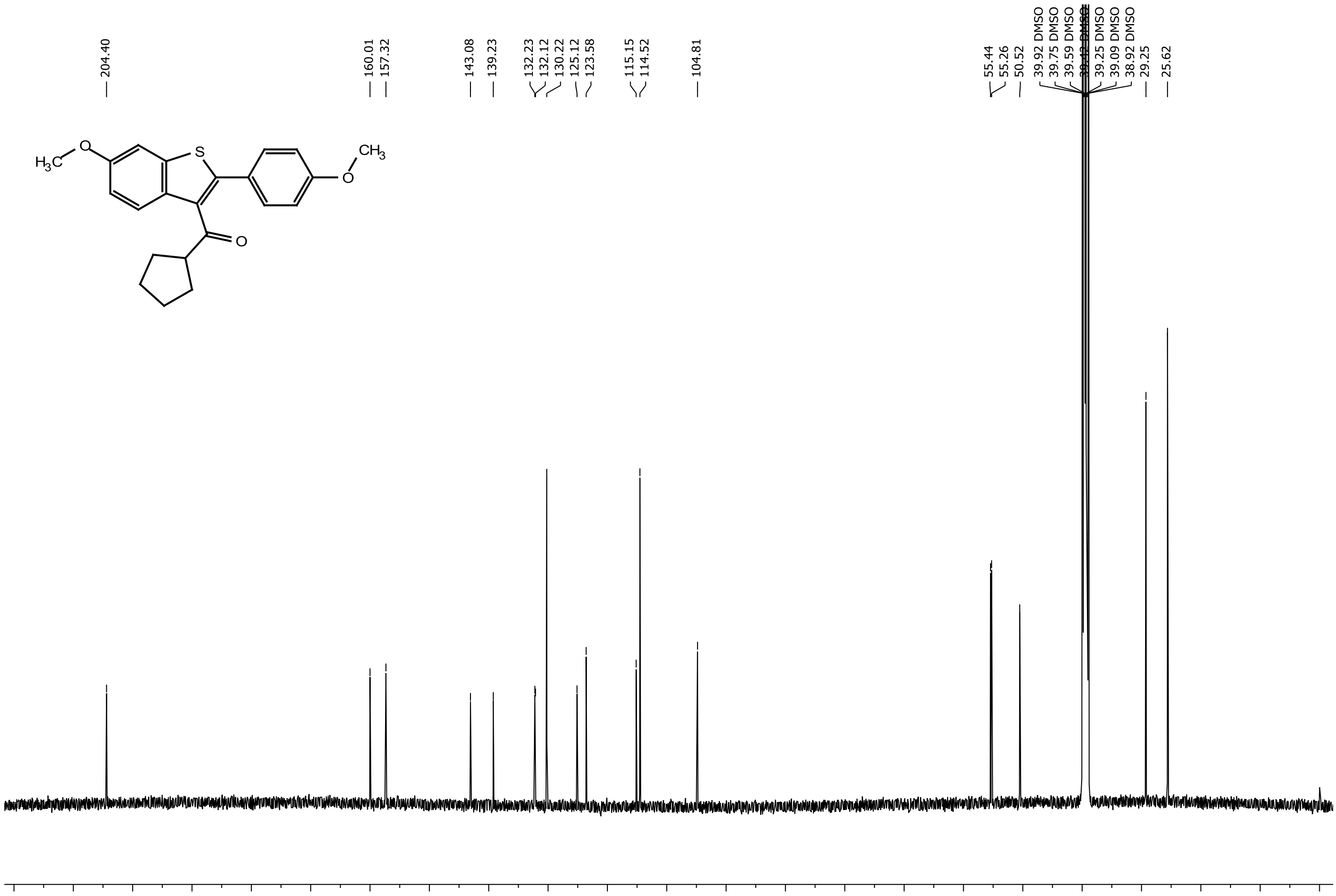

^{1}H NMR (400 MHz, C_2D_6OS) for compound **14b**

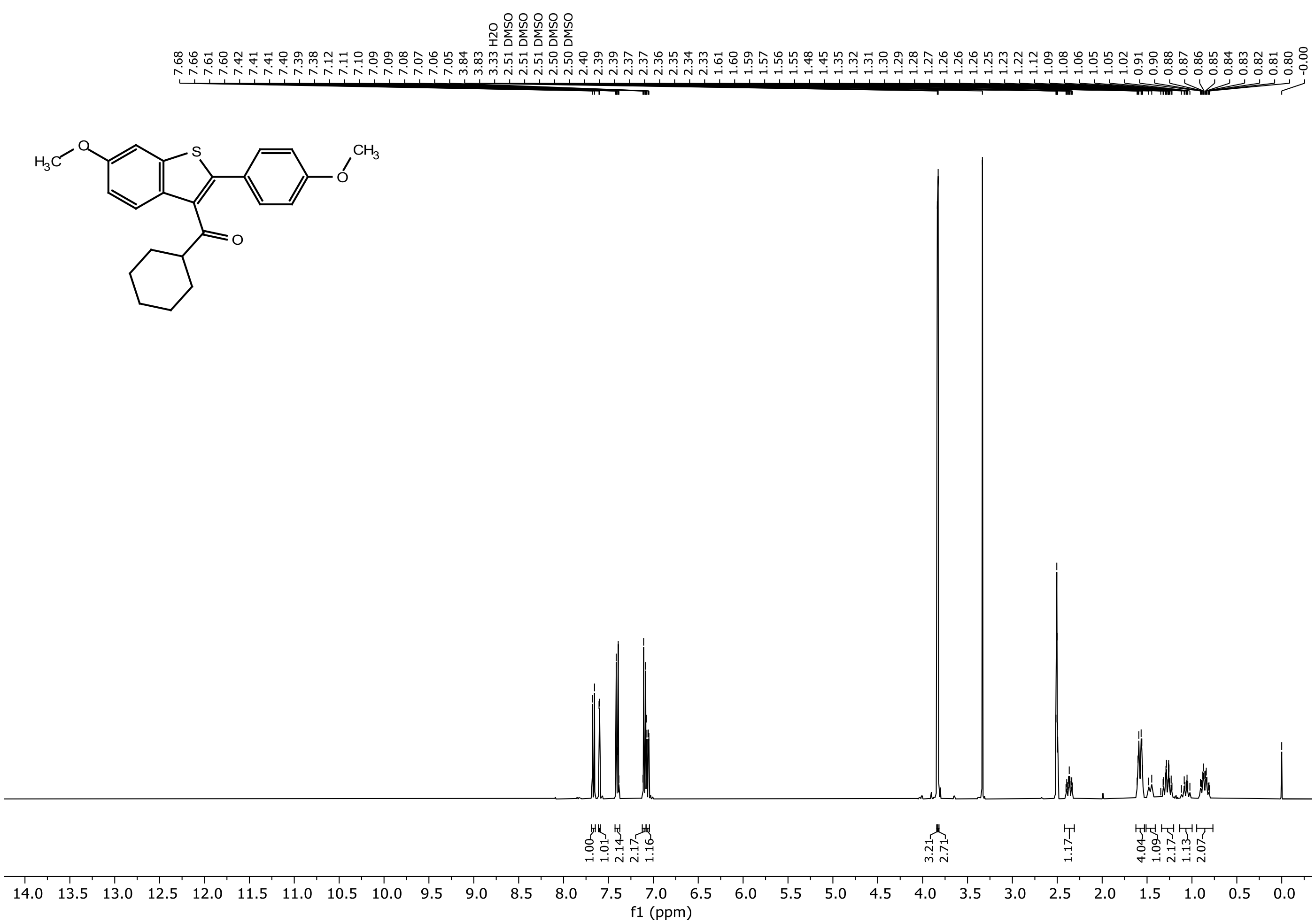

^{13}C NMR (101 MHz, C_2D_6OS) for compound **14b**

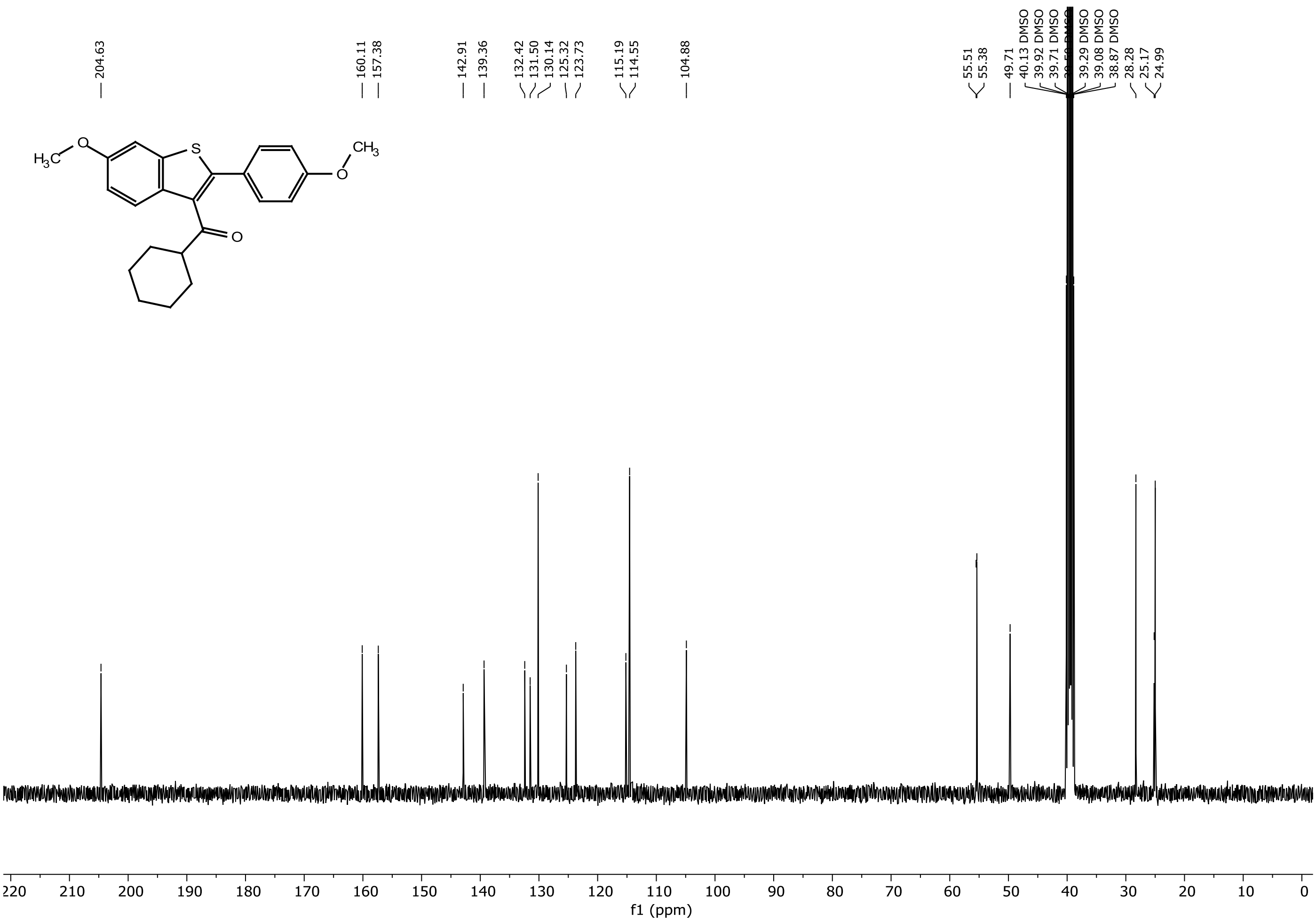

^{1}H NMR (600 MHz, C_2D_6OS) for compound **15a**

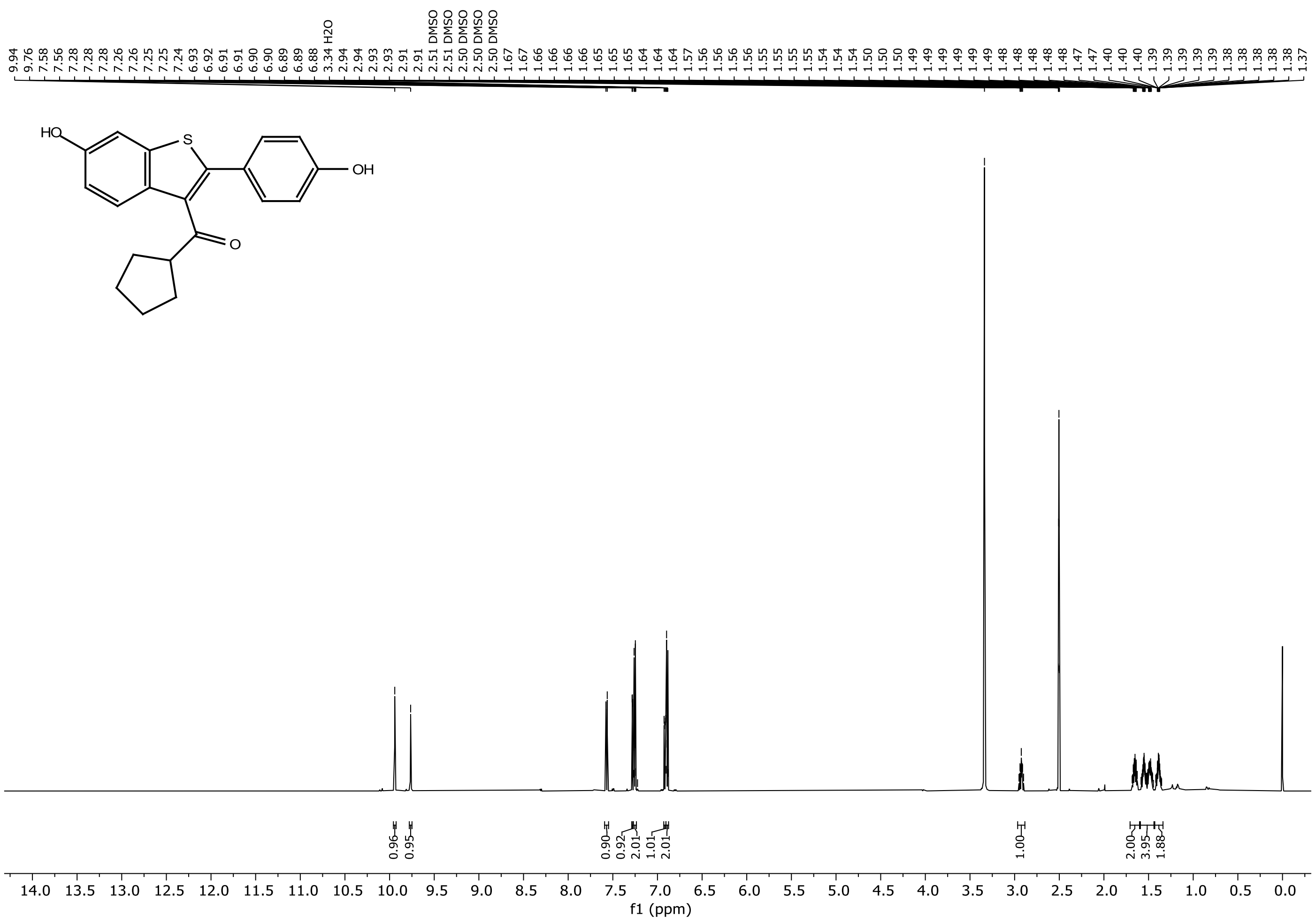

^{13}C NMR (151 MHz, C_2D_6OS) for compound **15a**

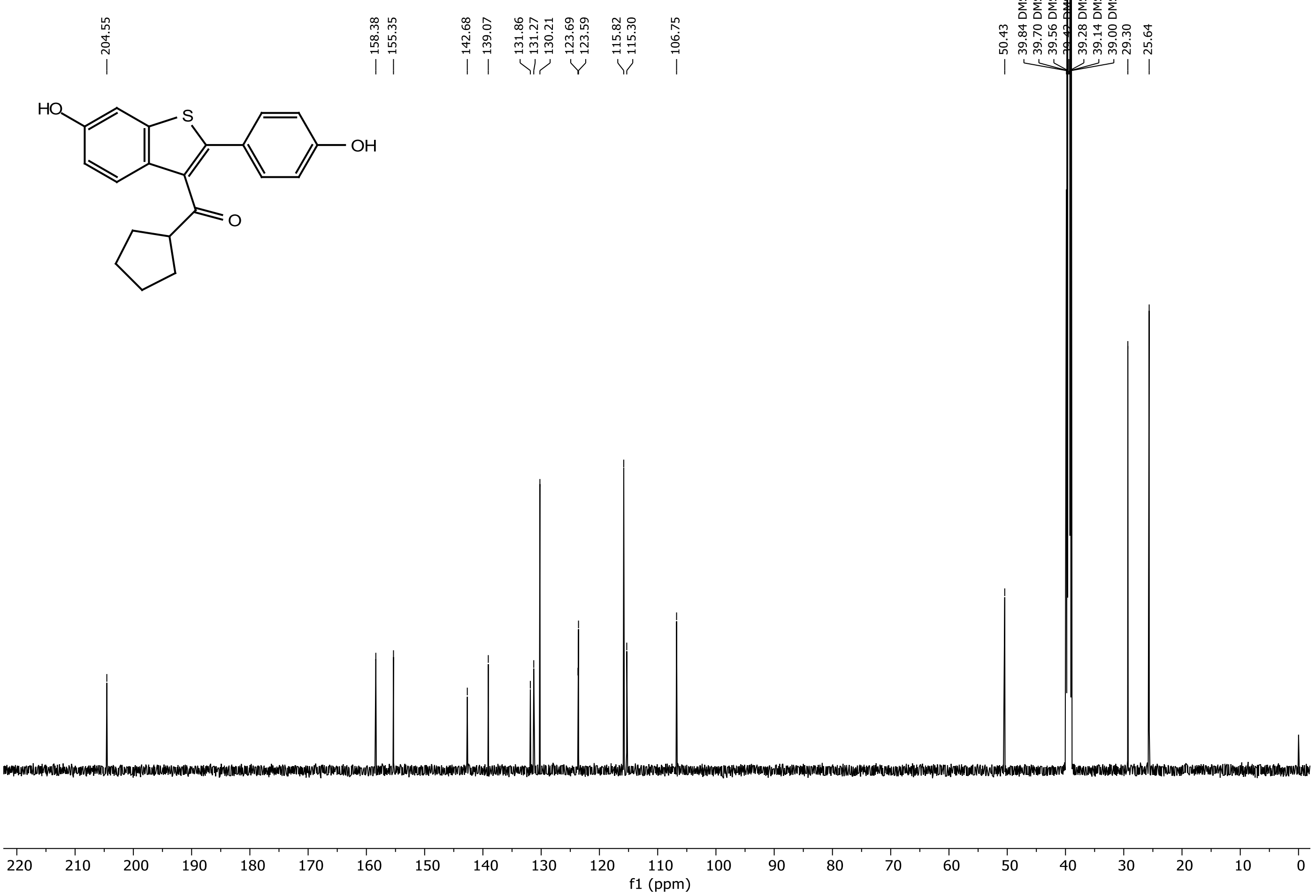

^{1}H NMR (600 MHz, C_2D_6OS) for compound **15b**

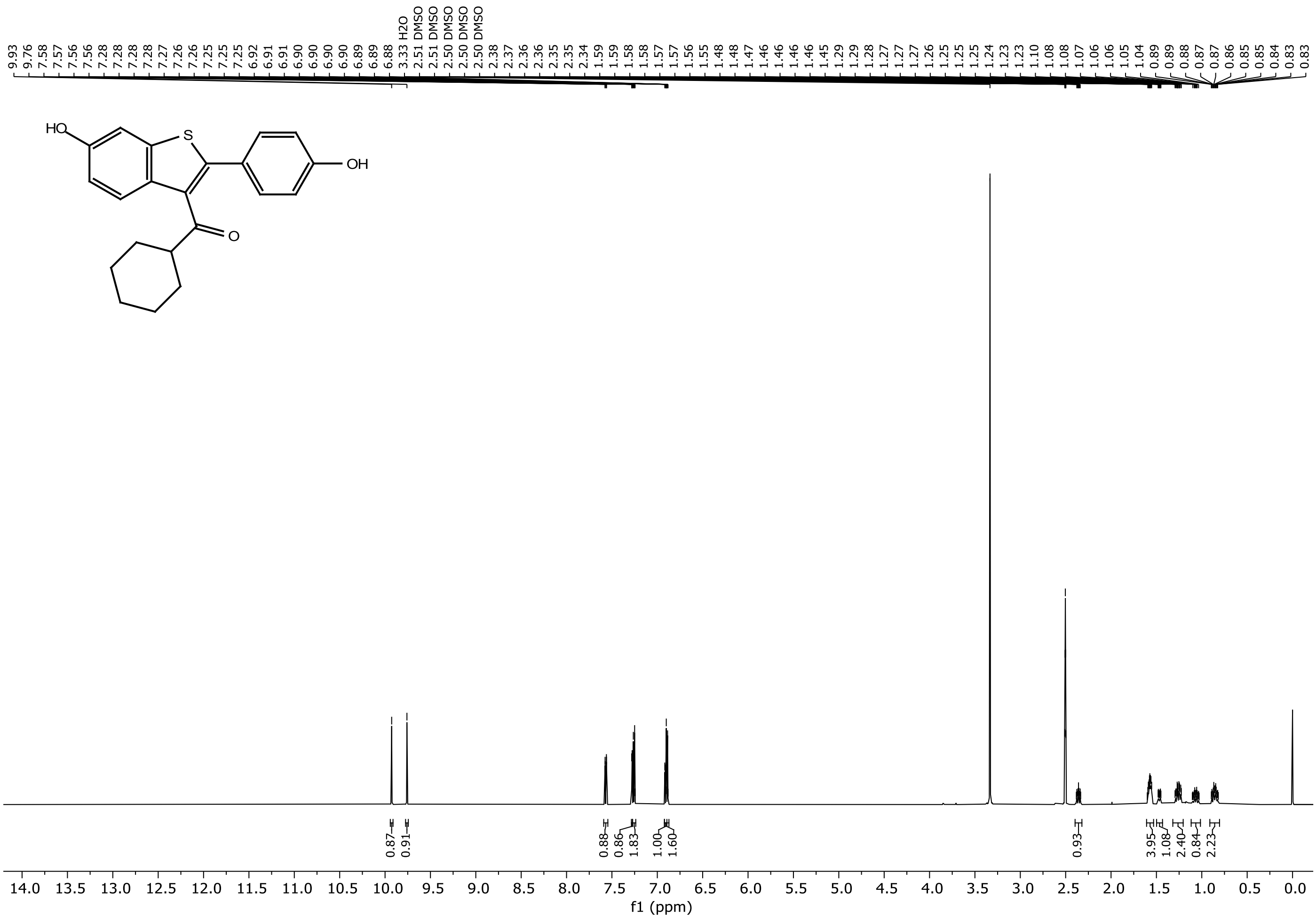

^{13}C NMR (151 MHz, C_2D_6OS) for compound **15b**

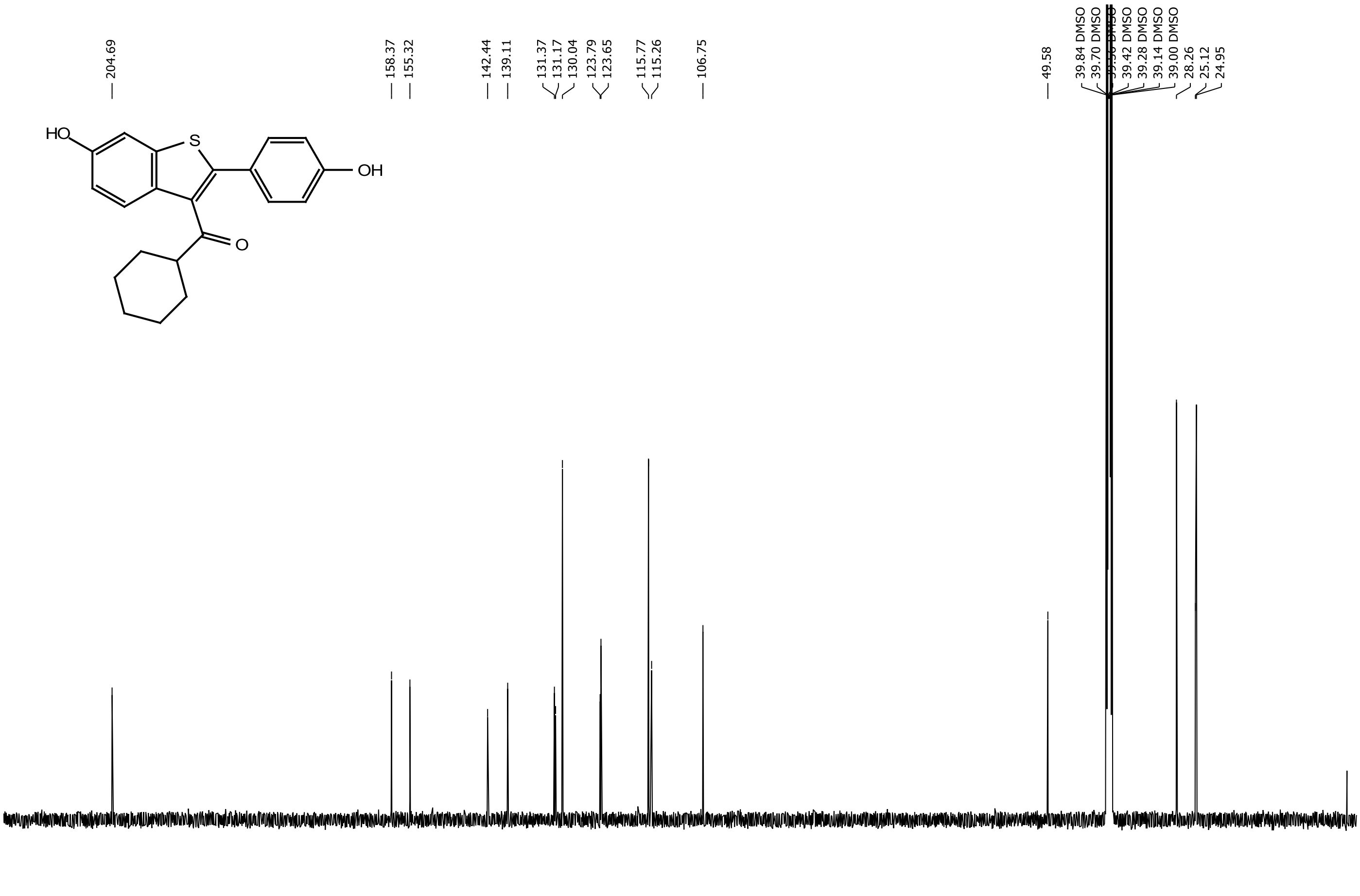

^{1}H NMR (500 MHz, C_2D_6OS) for compound **15c**

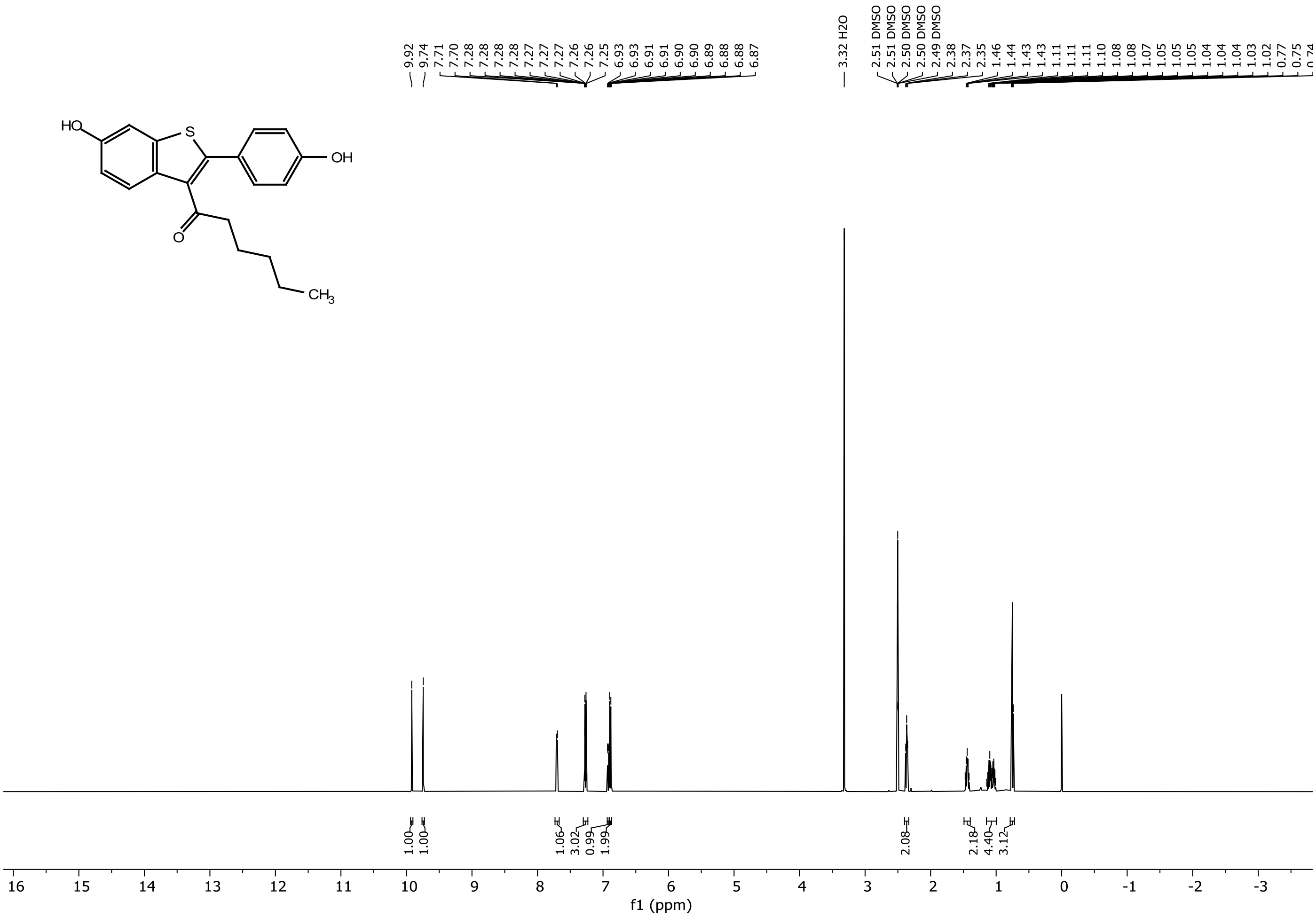

^{13}C NMR (126 MHz, C_2D_6OS) for compound **15c**

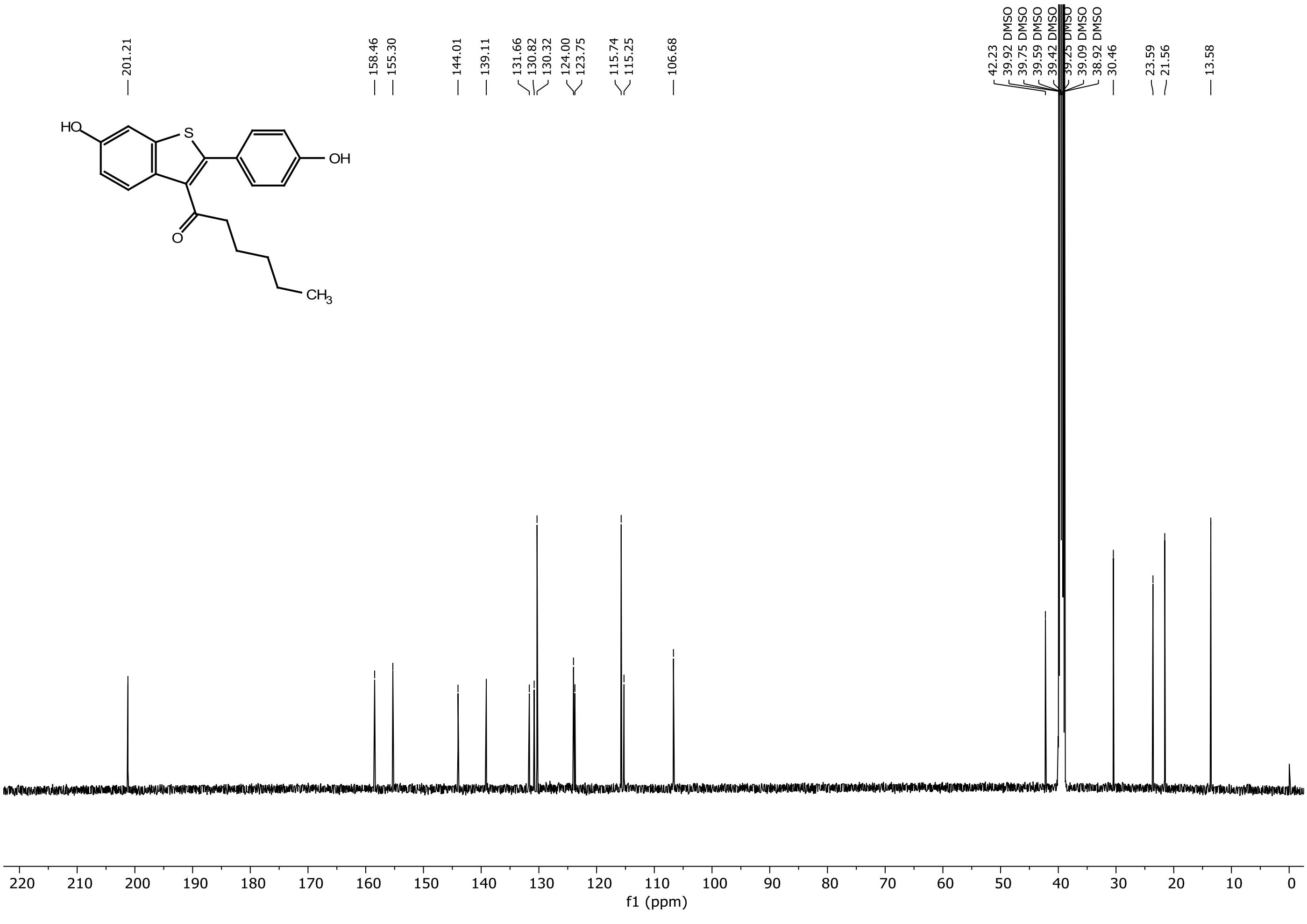

^{1}H NMR (500 MHz, C_2D_6OS) for compound **19a**

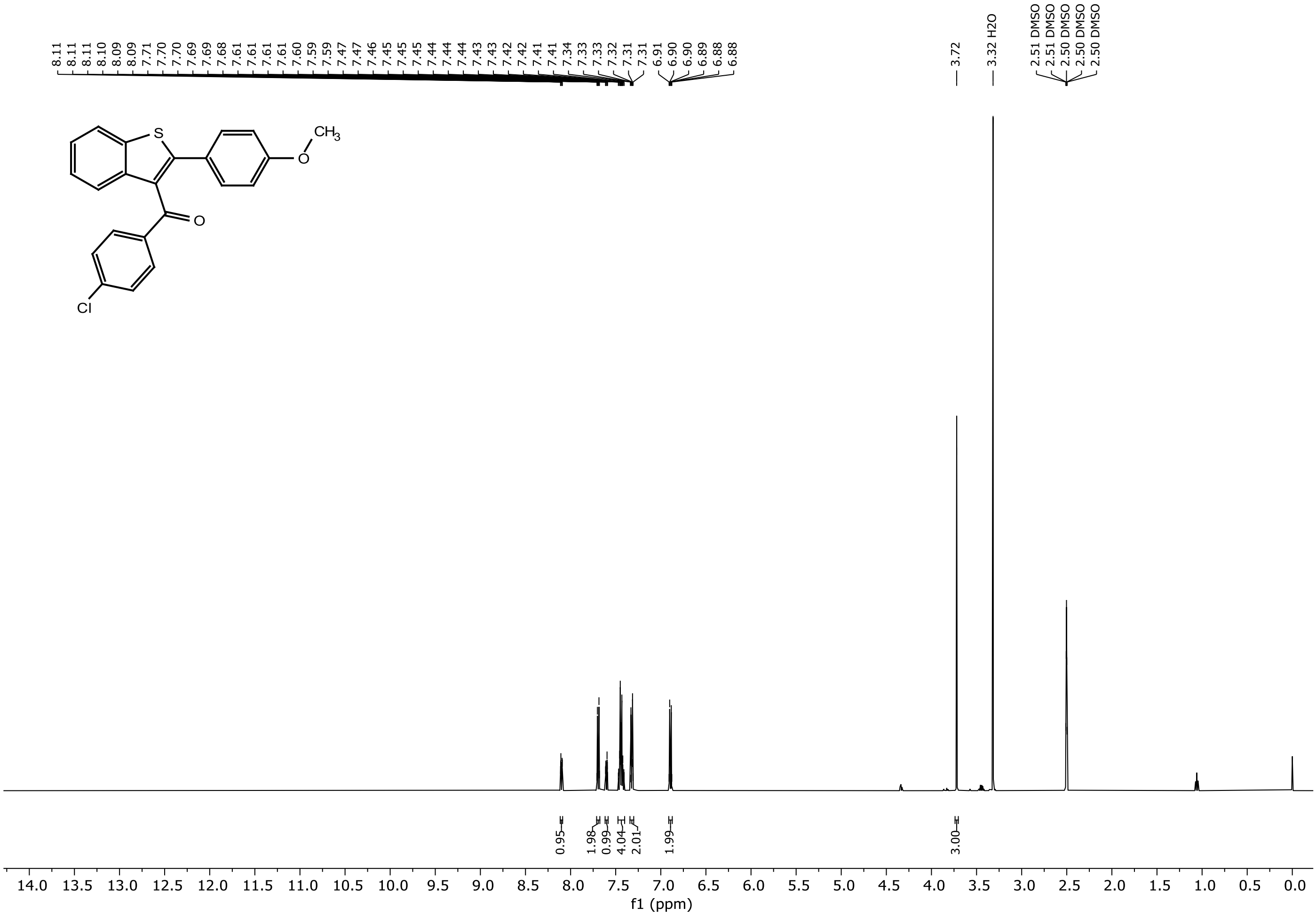

^{13}C NMR (126 MHz, C_2D_6OS) for compound **19a**

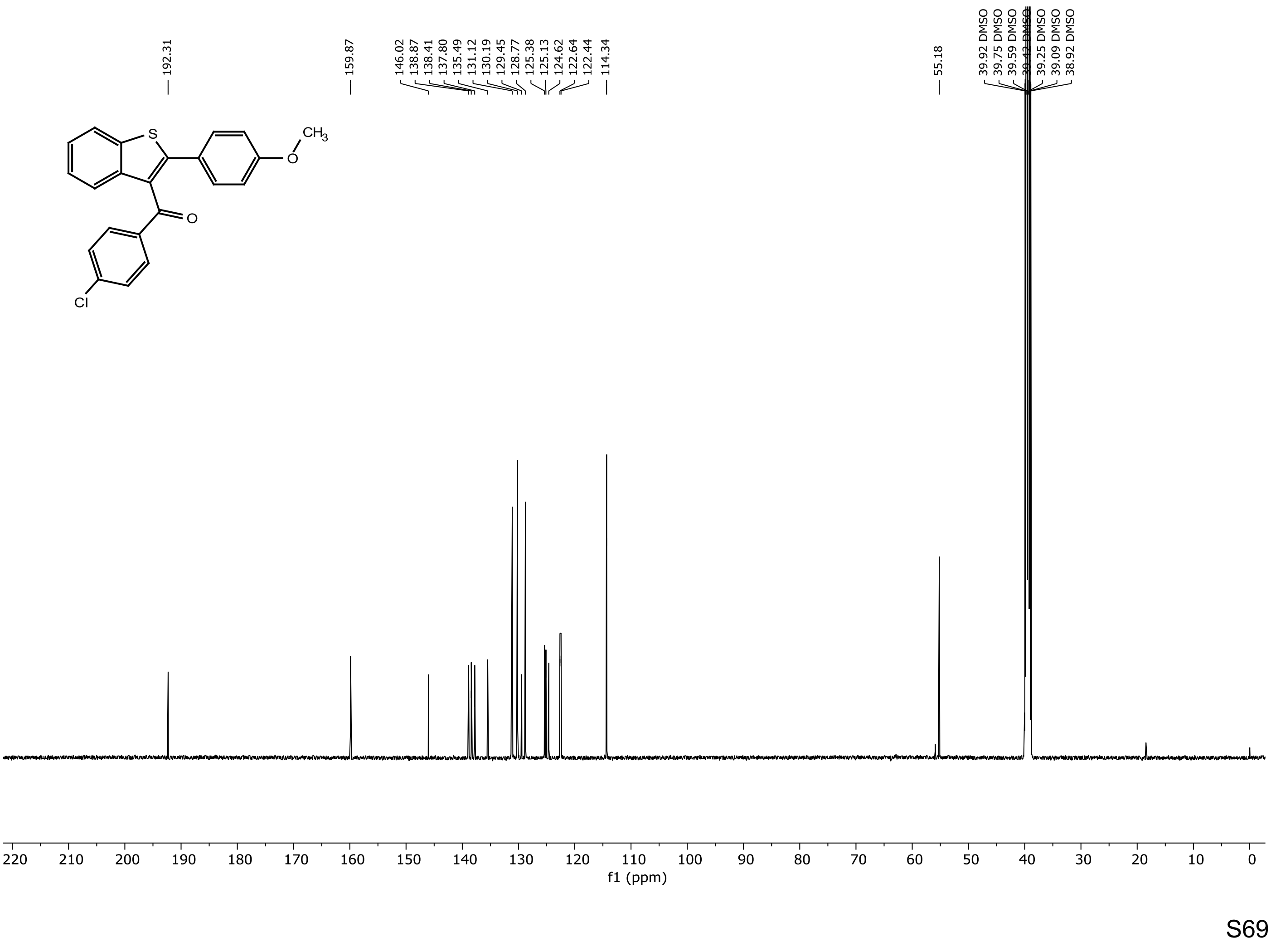

^{1}H NMR (400 MHz, C_2D_6OS) for compound **19b**

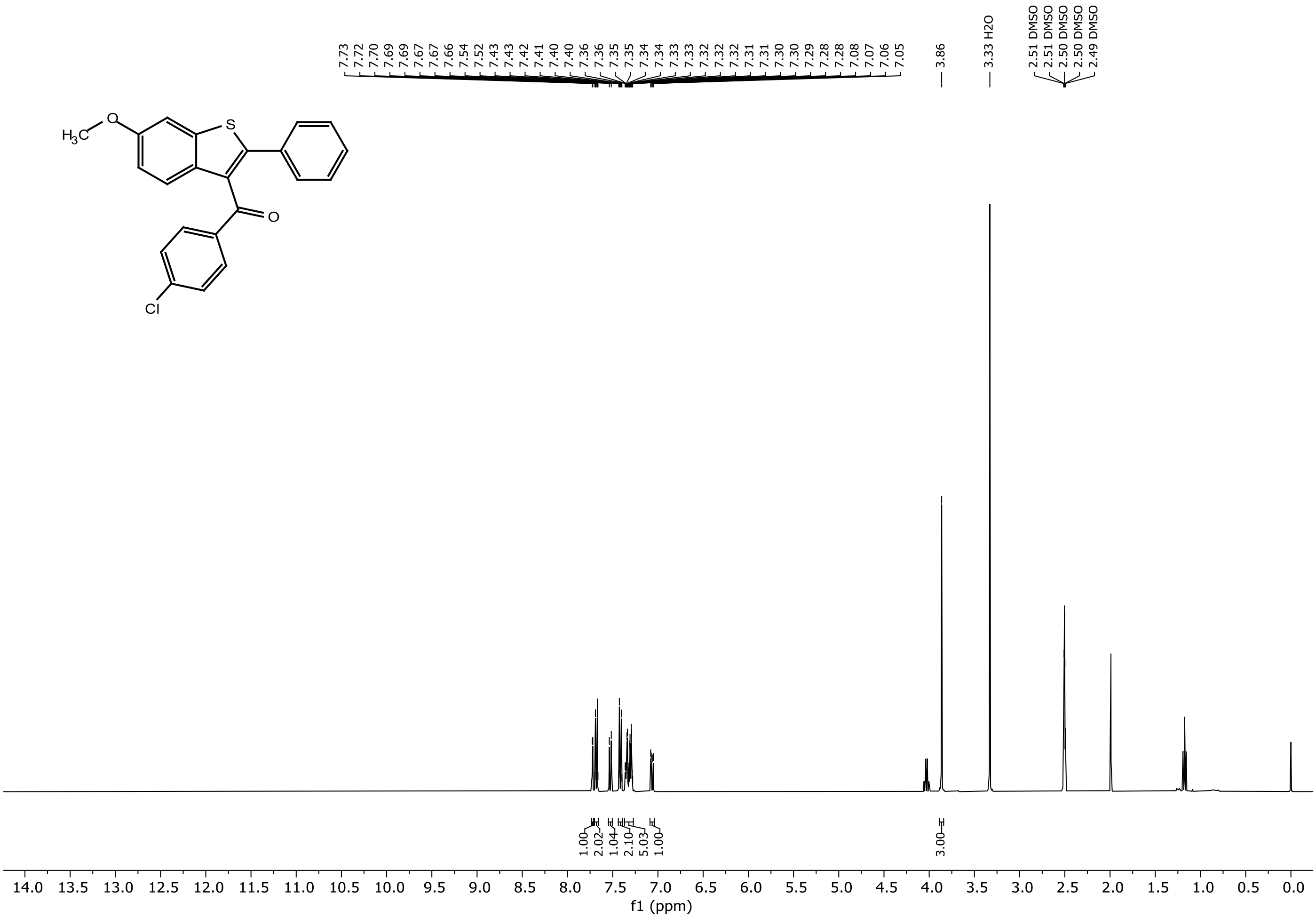

^{13}C NMR (101 MHz, C_2D_6OS) for compound **19b**

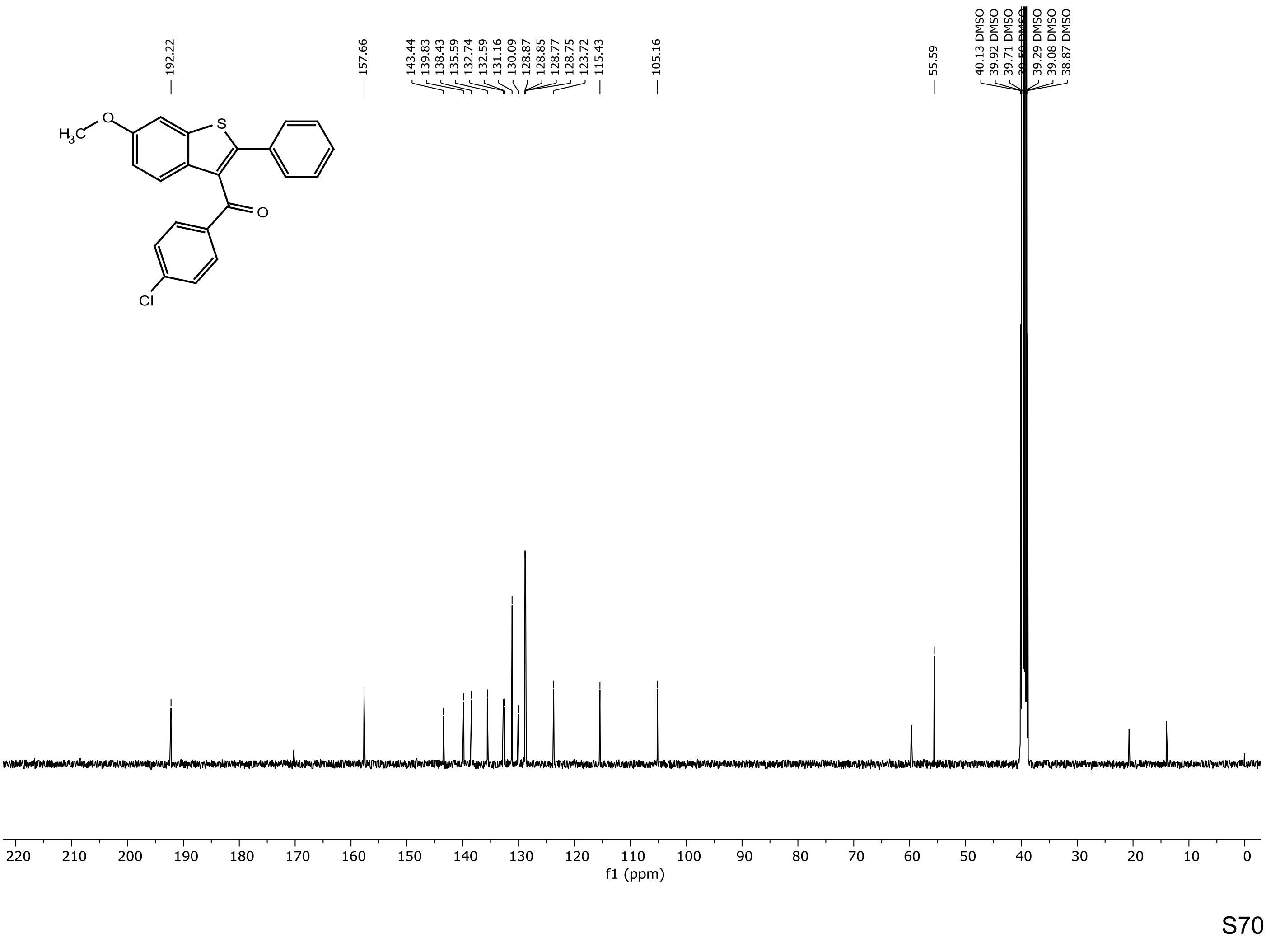

^{1}H NMR (500 MHz, C_2D_6OS) for compound **19c**

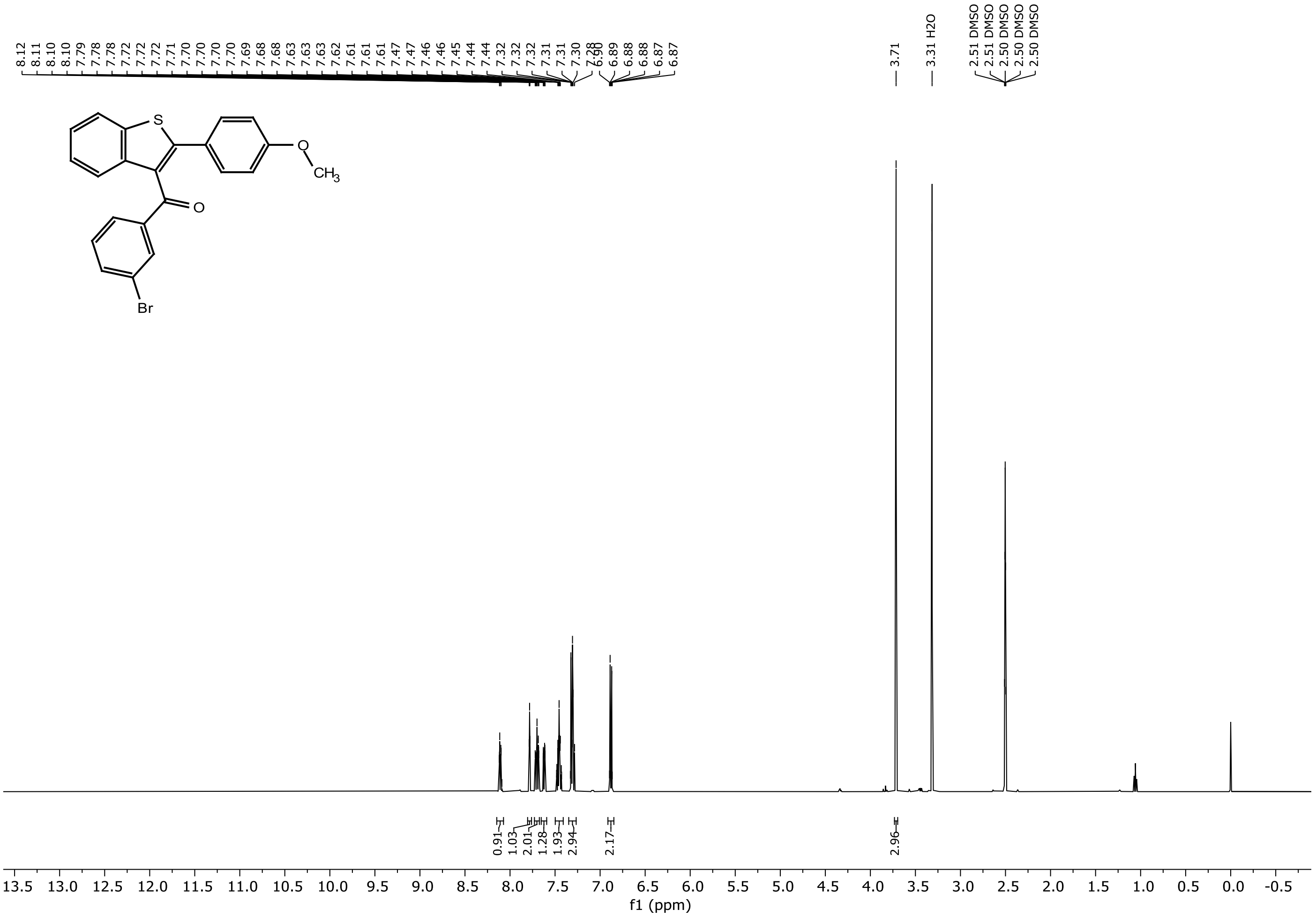

^{13}C NMR (126 MHz, C_2D_6OS) for compound **19c**

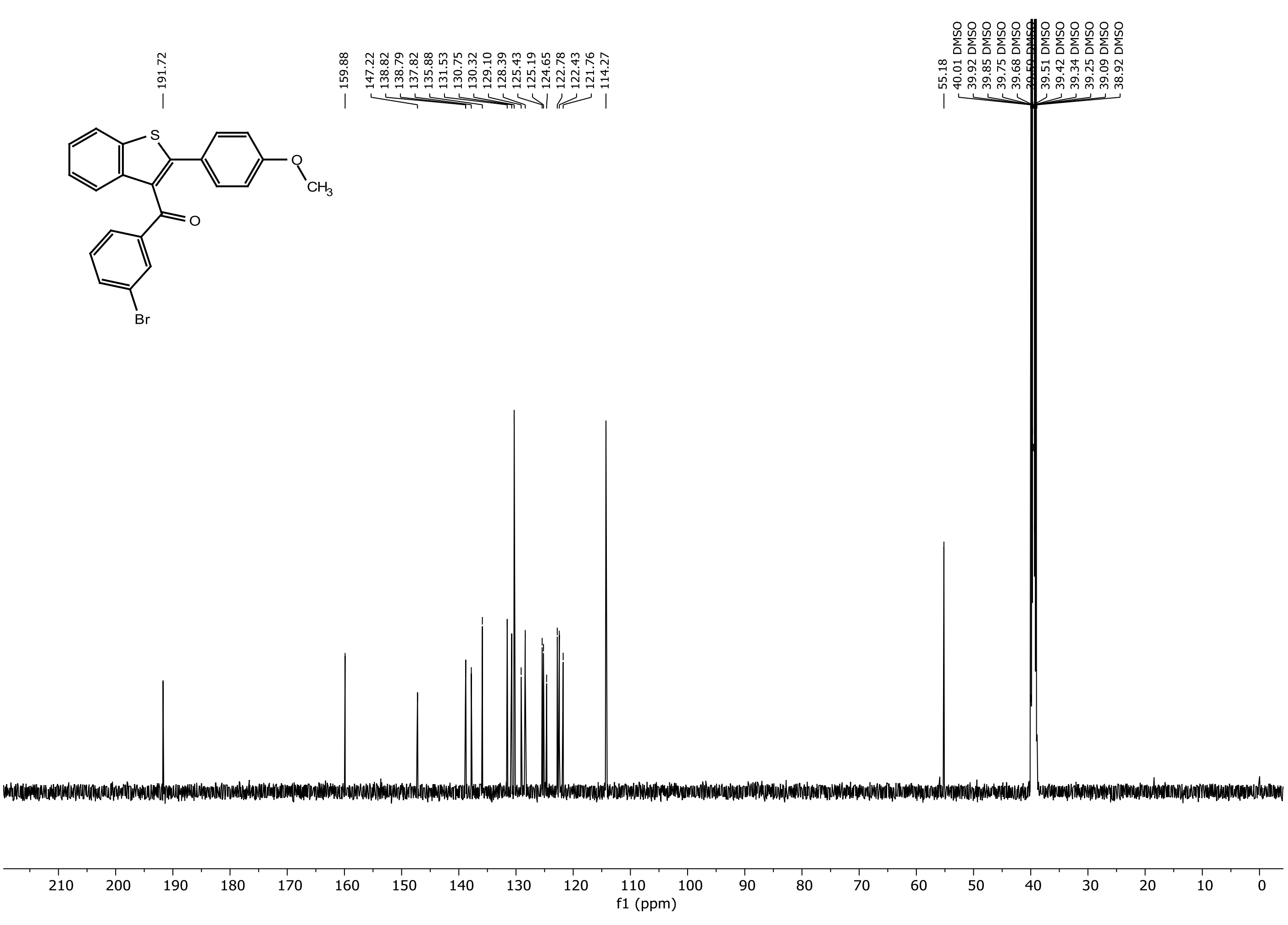

^{1}H NMR (500 MHz, C_2D_6OS) for compound **19d**

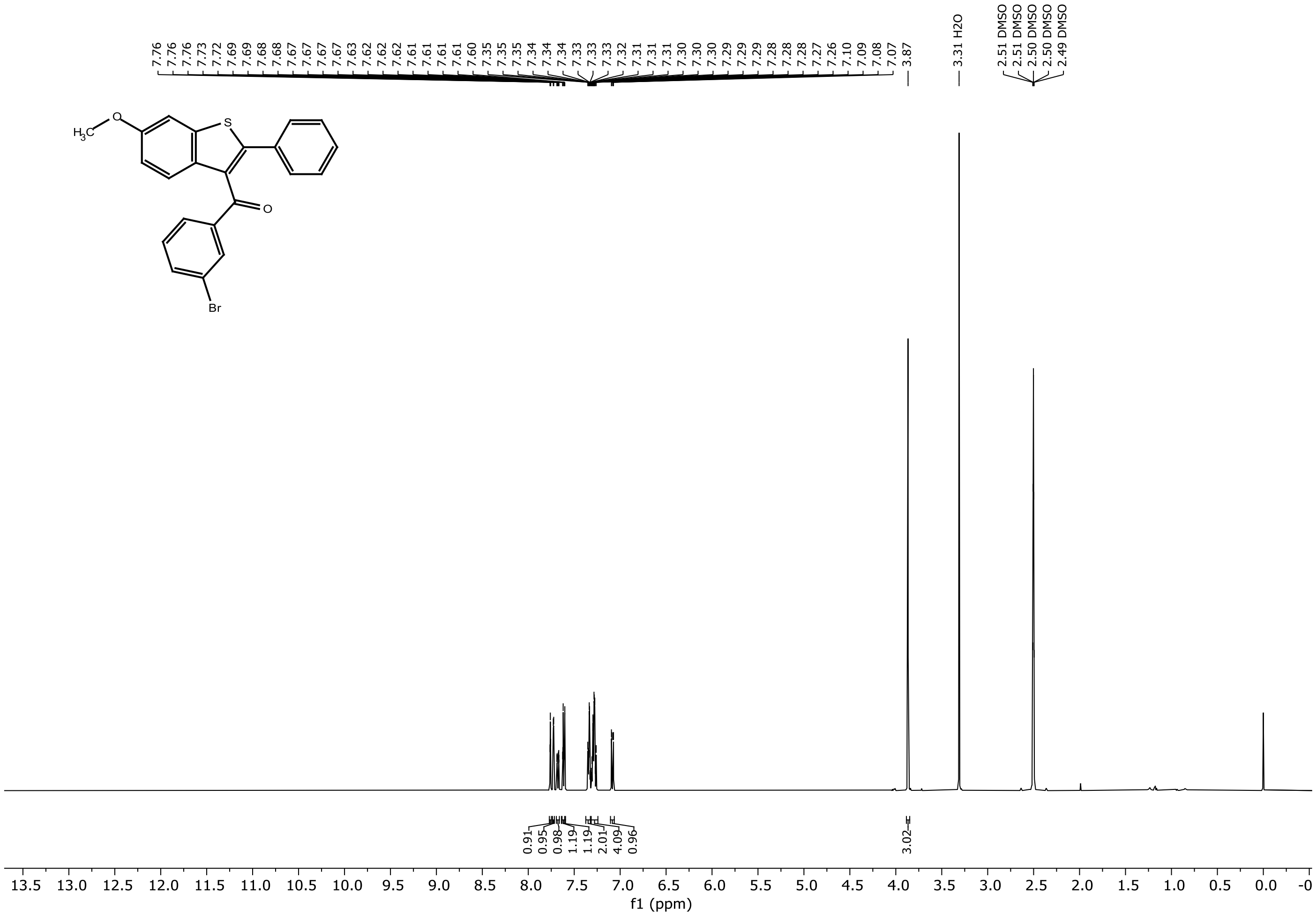

^{13}C NMR (126 MHz, C_2D_6OS) for compound **19d**

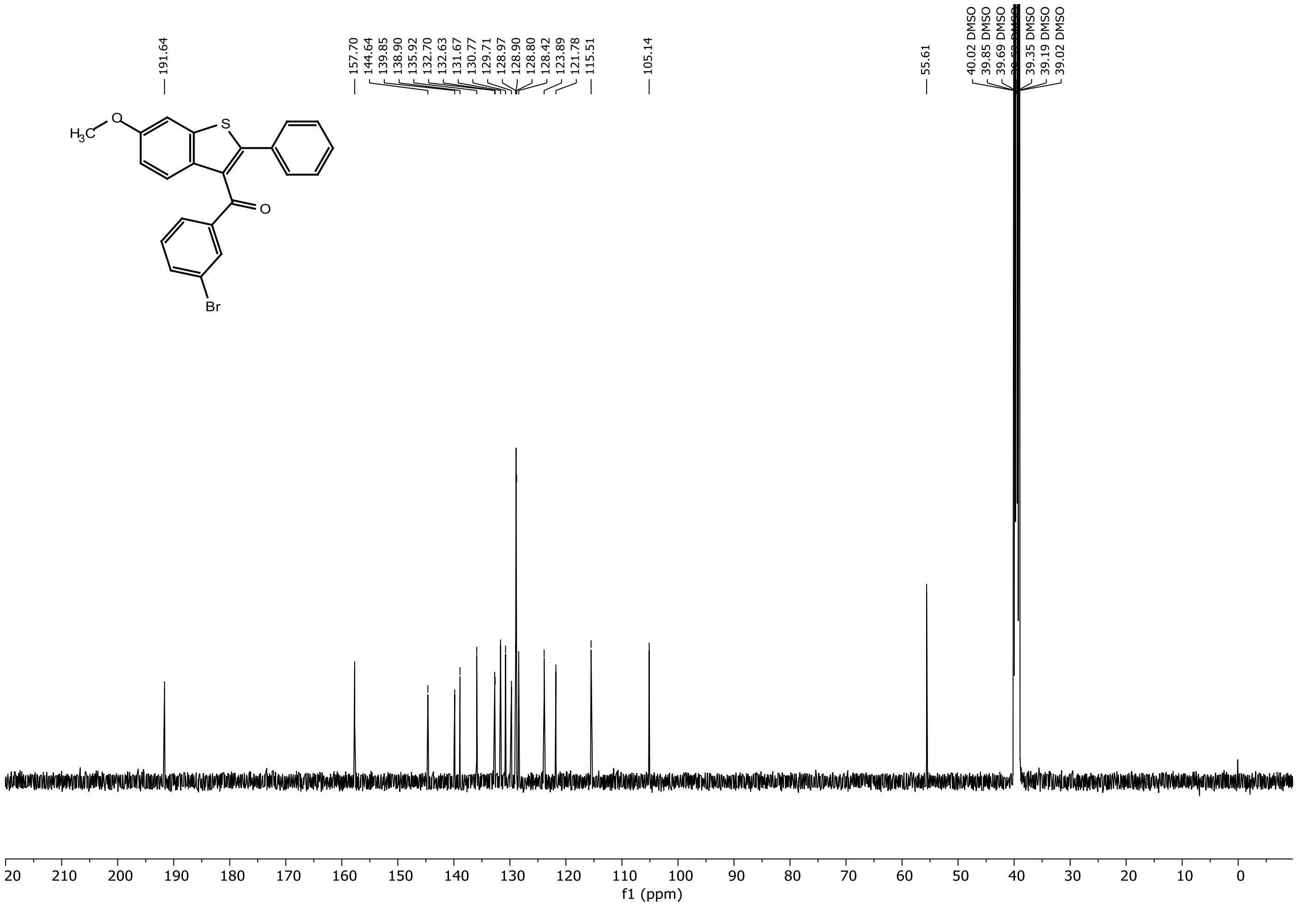

^1H NMR (500 MHz, C_2D_6OS) for compound **19f**

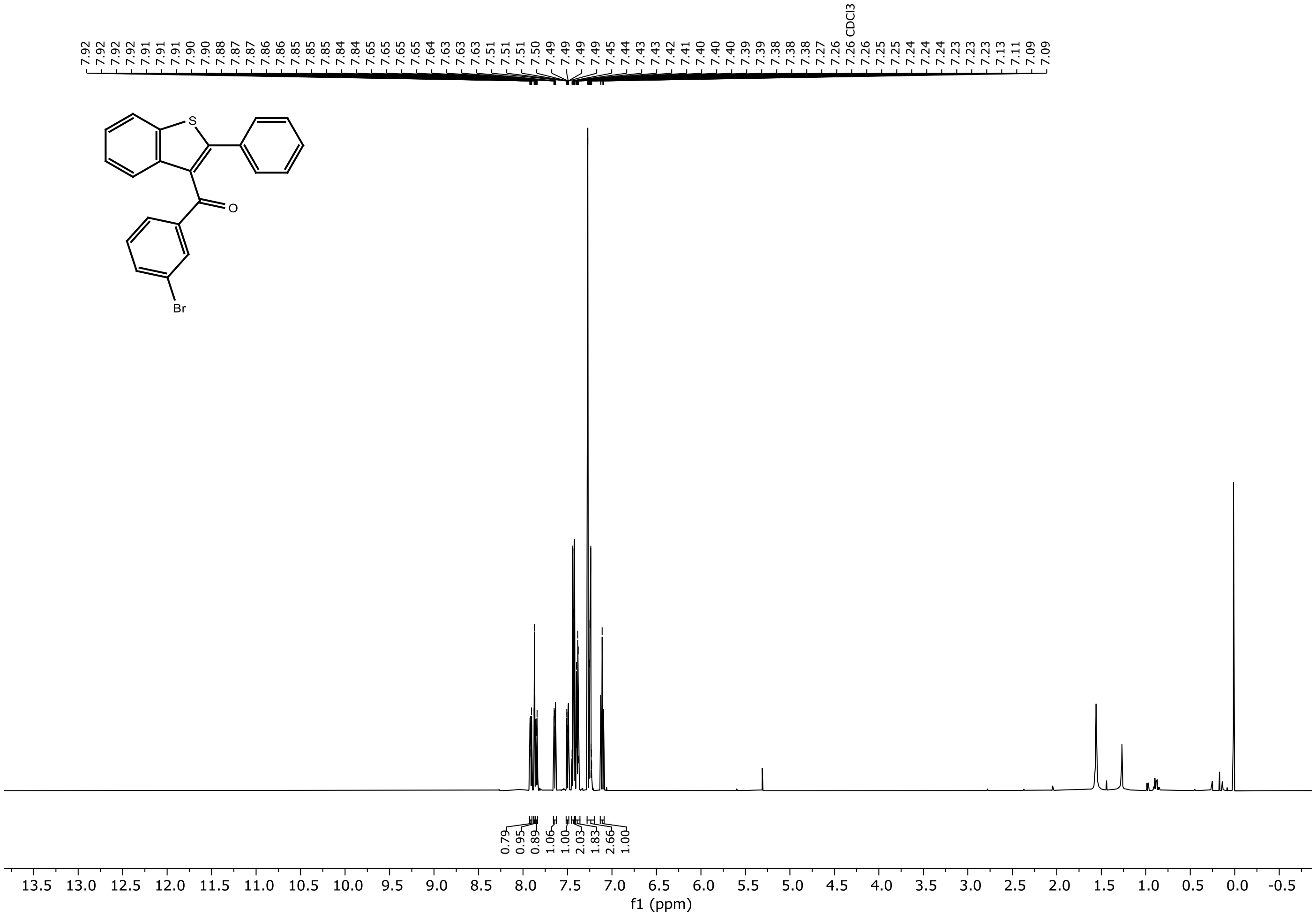

^{13}C NMR (126 MHz, C_2D_6OS) for compound **19f**

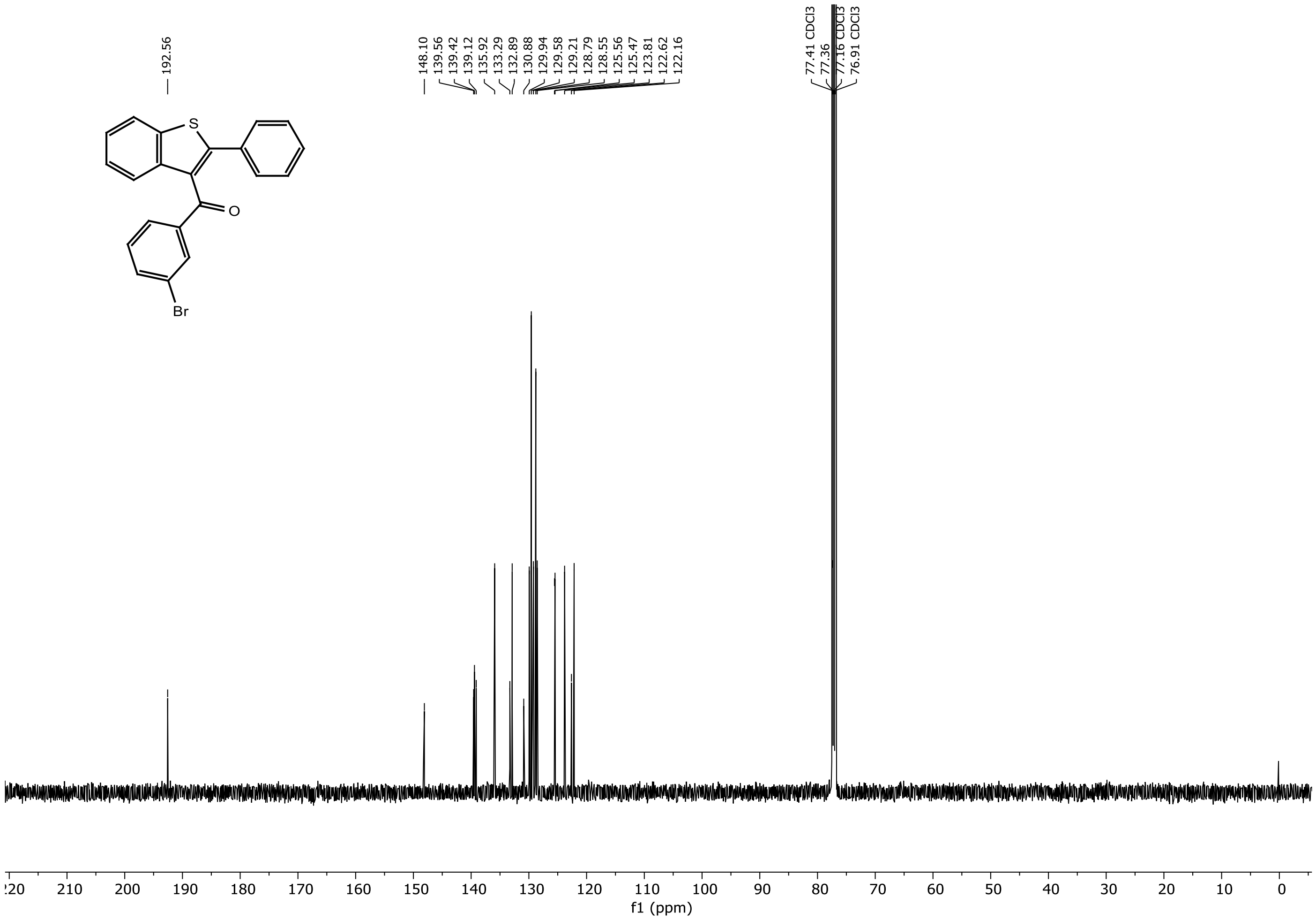

^1H NMR (500 MHz, C_2D_6OS) for compound **20a**

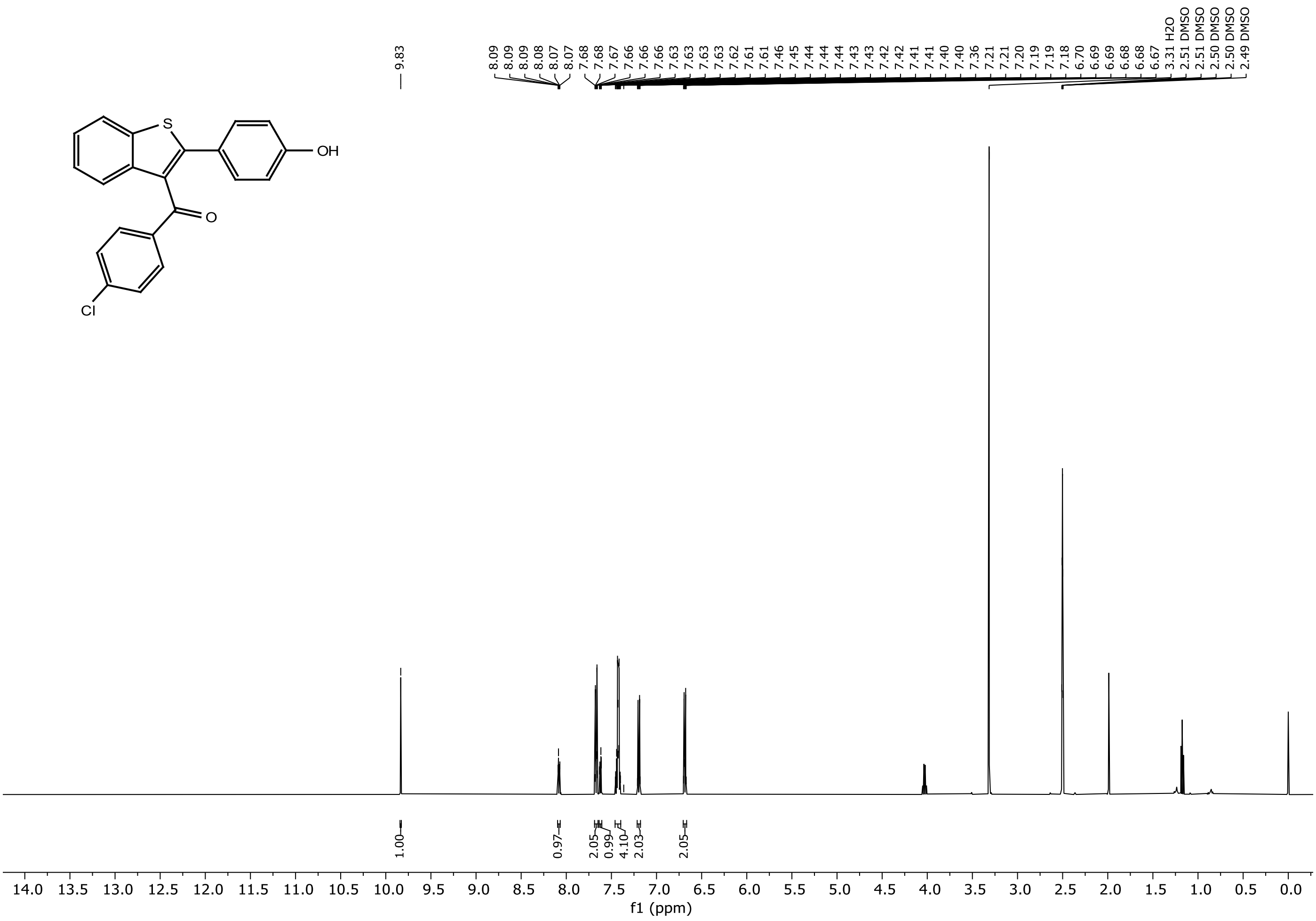

^{13}C NMR (126 MHz, C_2D_6OS) for compound **20a**

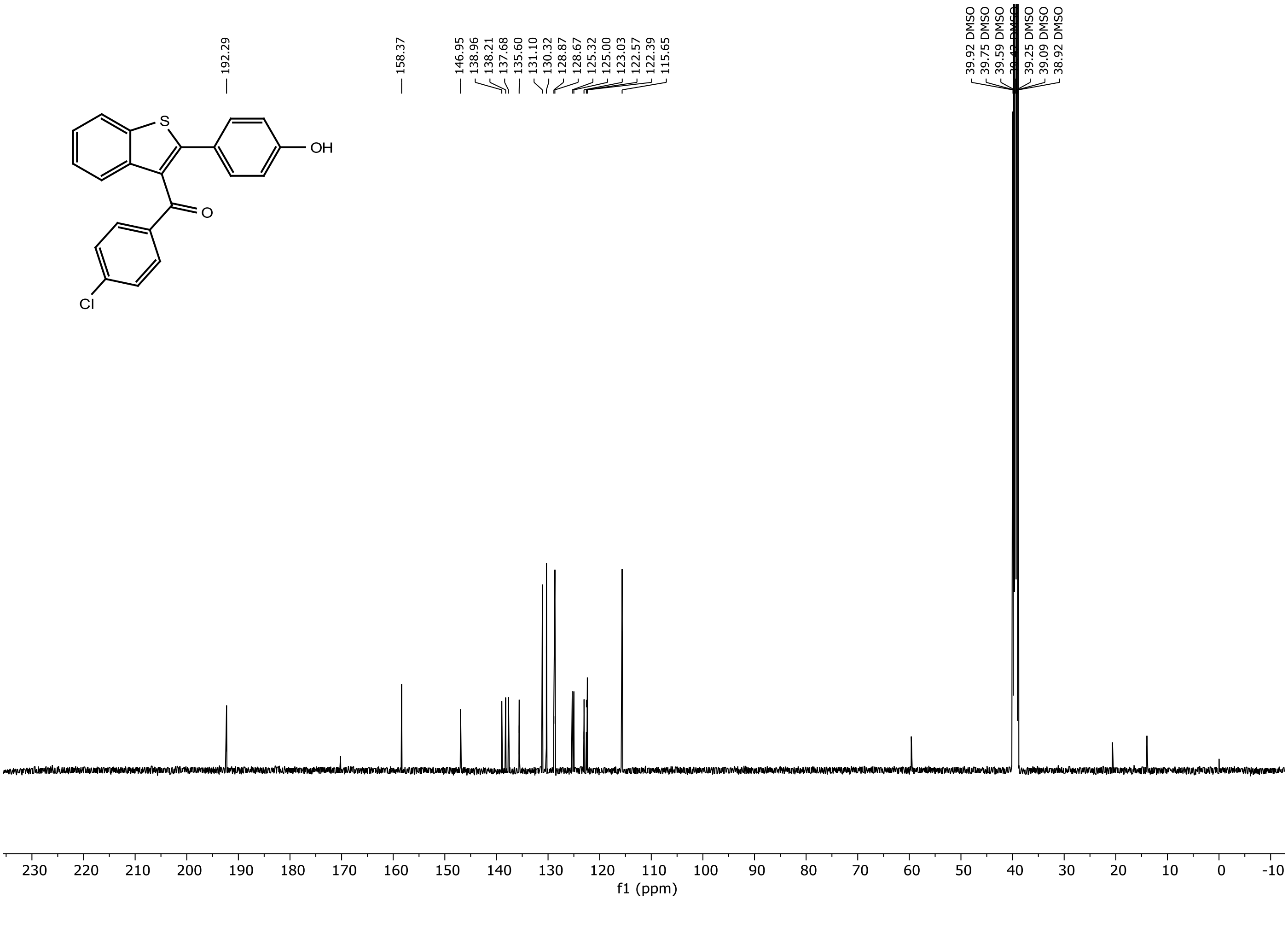

^1H NMR (600 MHz, C_2D_6OS) for compound **20b**

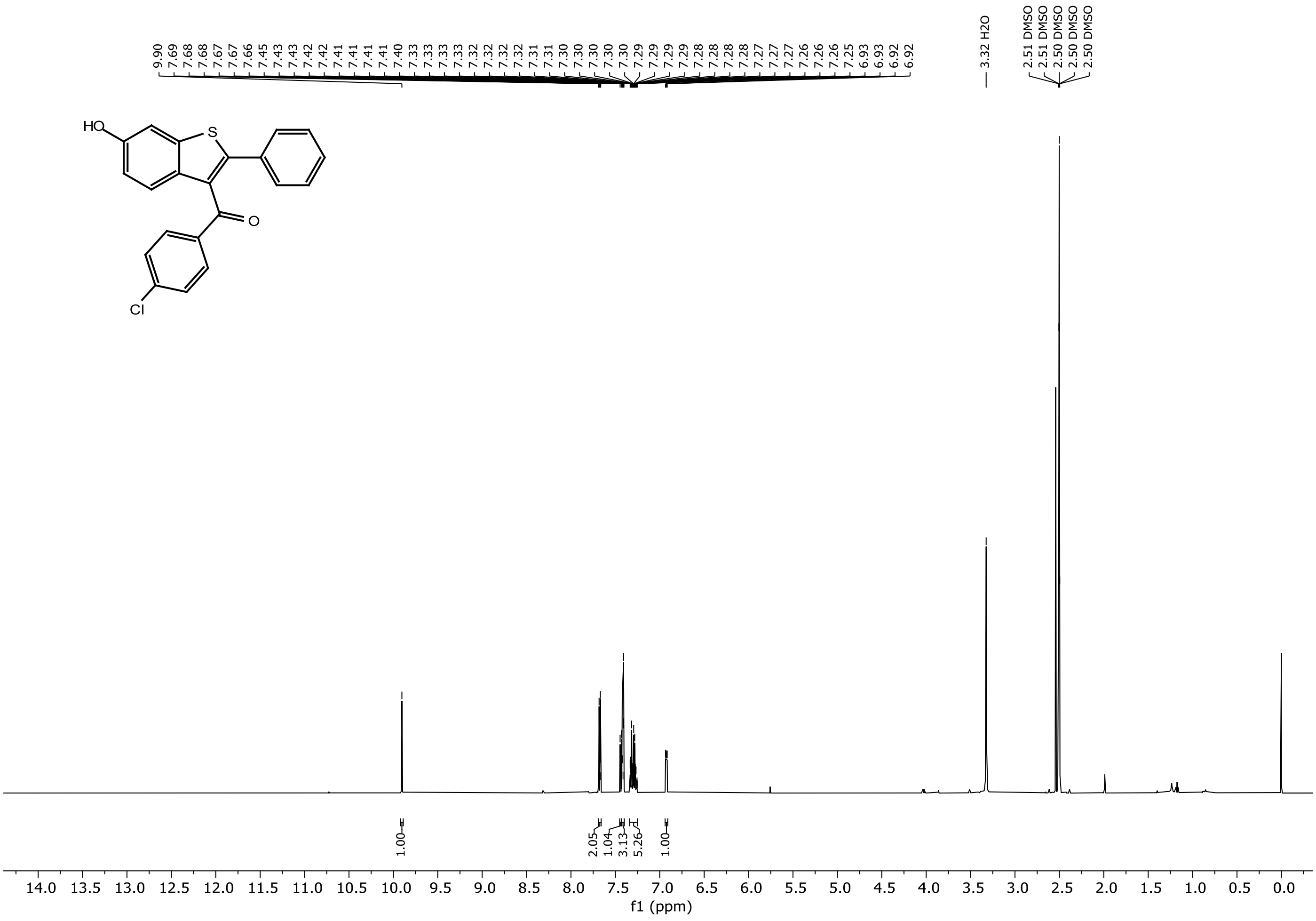

^{13}C NMR (151 MHz, C_2D_6OS) for compound **20b**

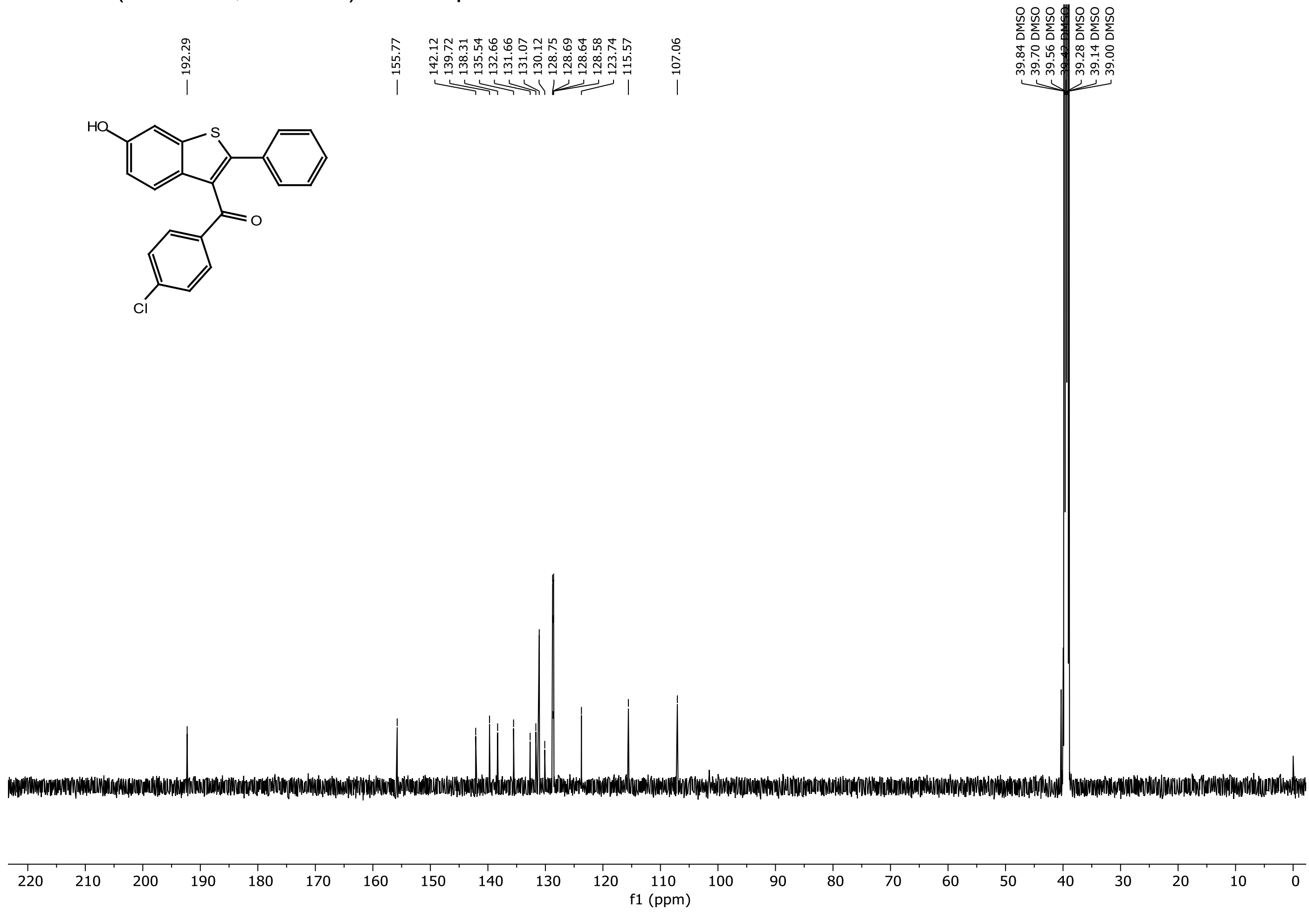

^{1}H NMR (600 MHz, $CDCl_3$) for compound **20c**

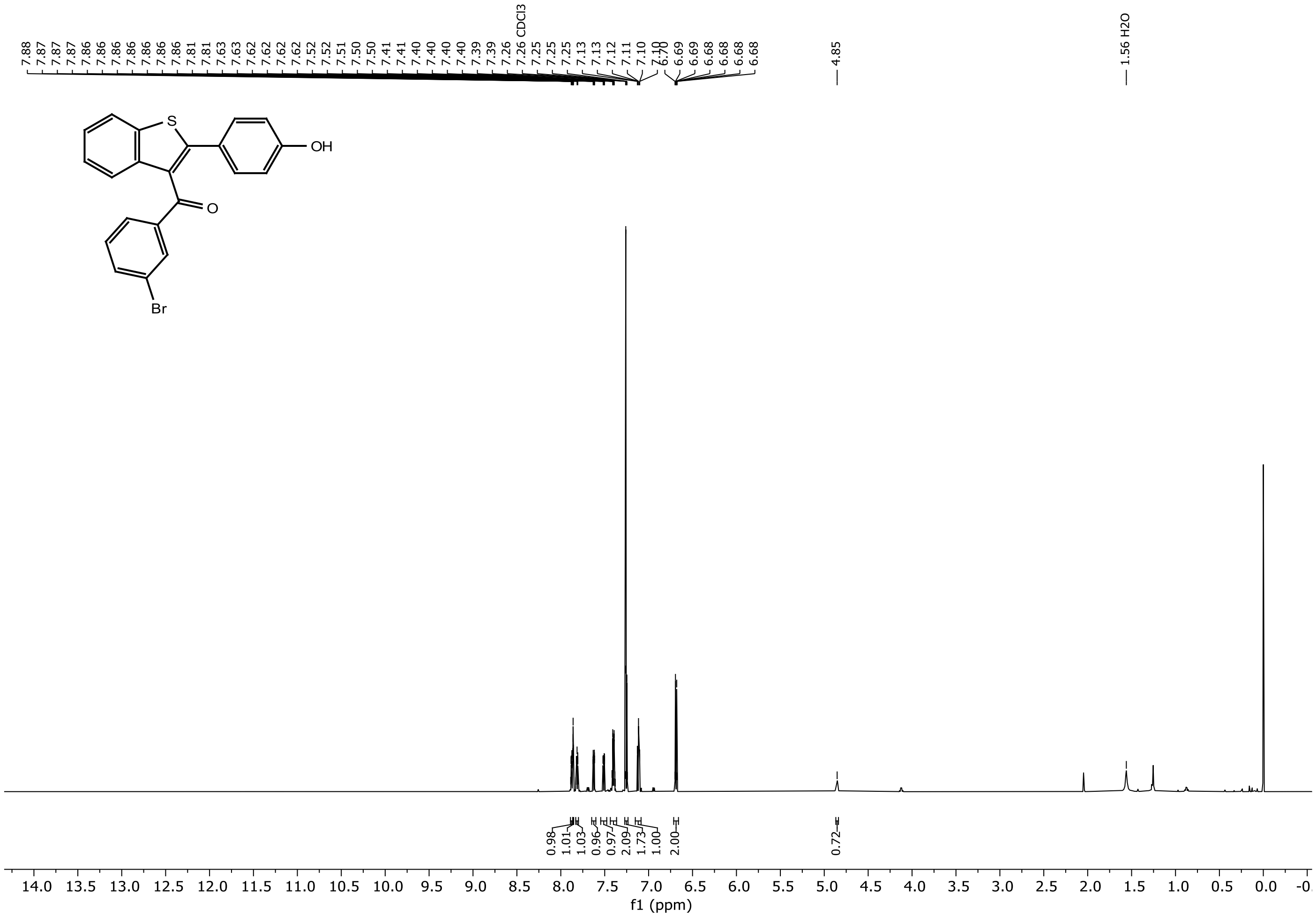

^{13}C NMR (151 MHz, $CDCl_3$) for compound **20c**

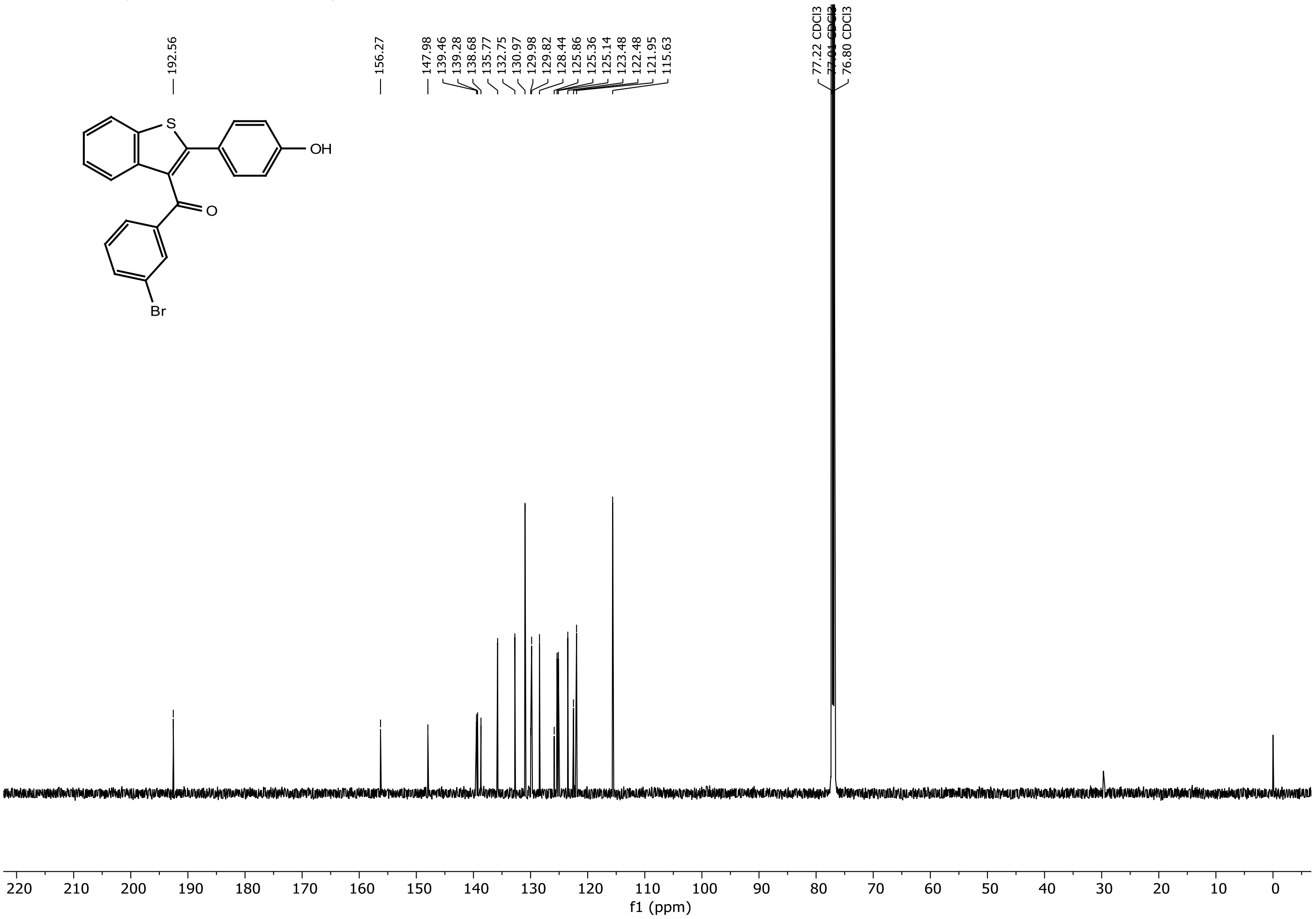

^{1}H NMR (600 MHz, C_2D_6OS) for compound **20d**

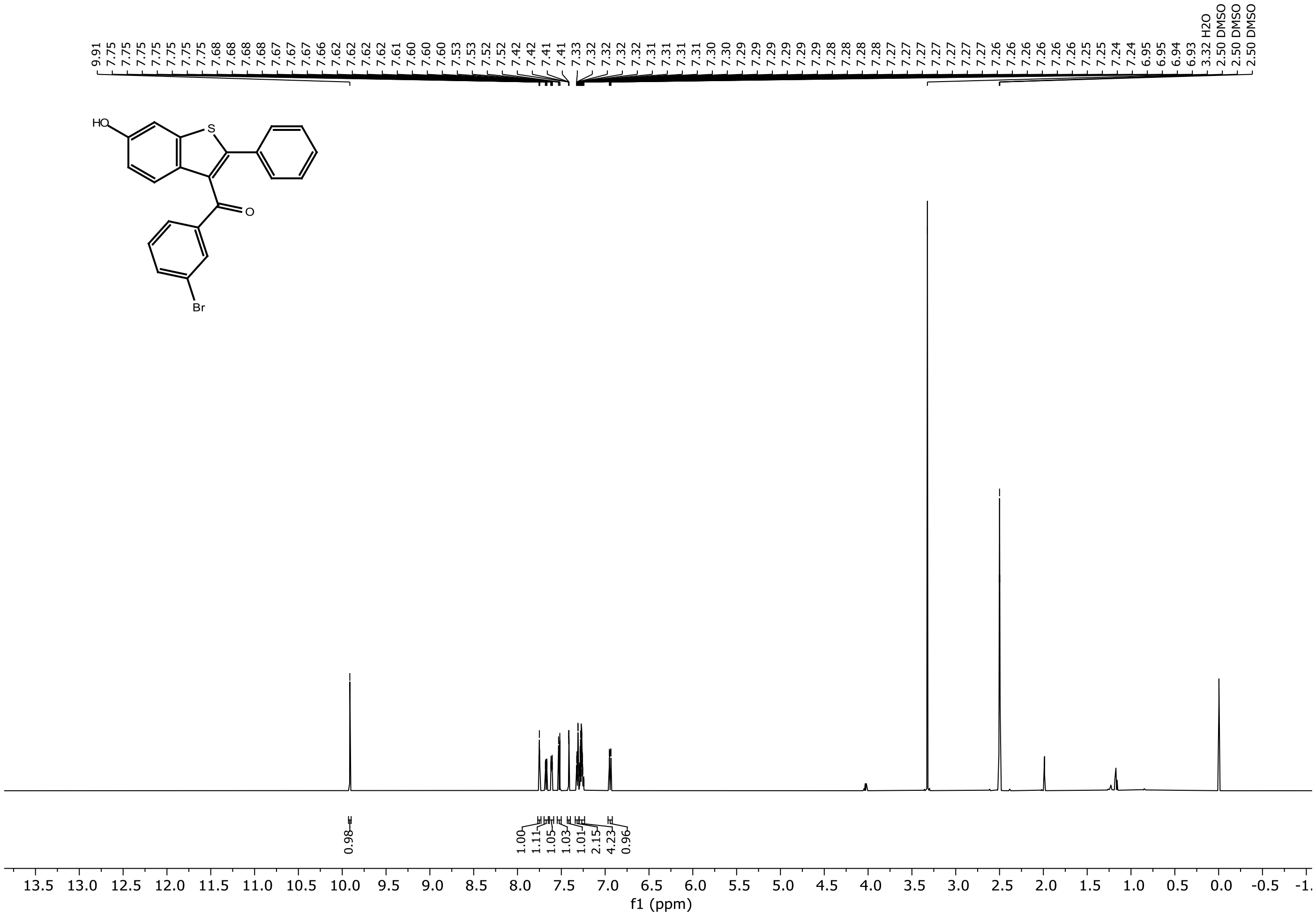

^{13}C NMR (151 MHz, C_2D_6OS) for compound **20d**

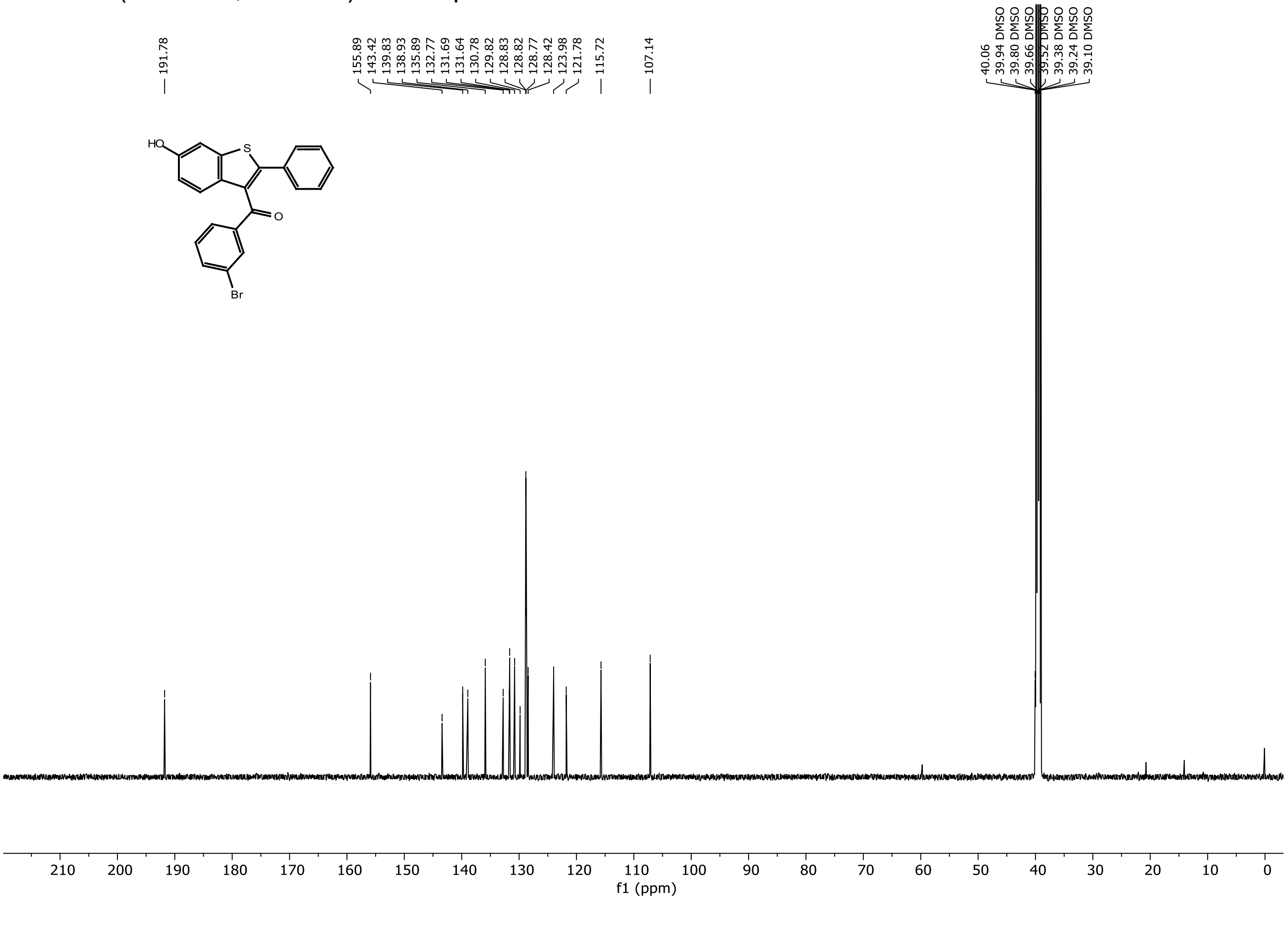

Supplementary HPLC Chromatograms

HPLC chromatogram of **9a** (KuThi035) (Method 2):

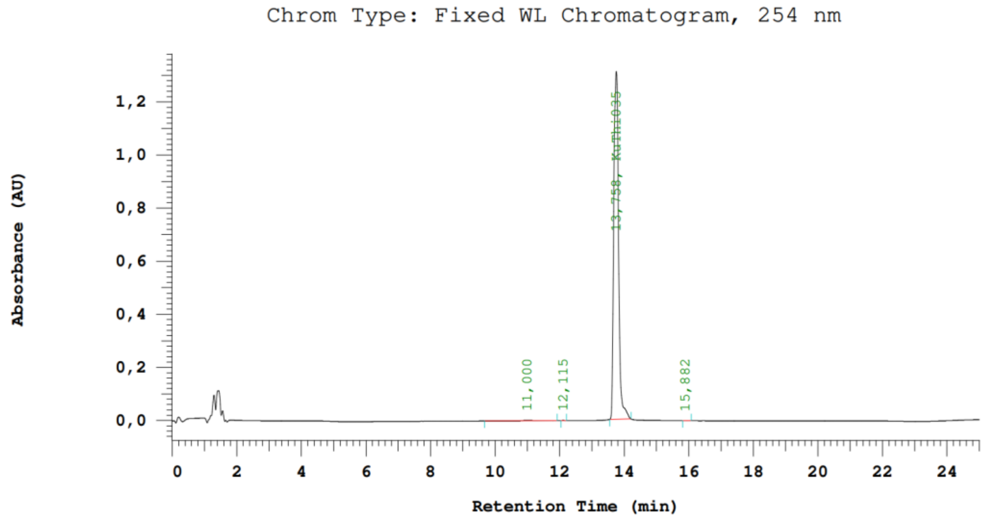

HPLC chromatogram of **9b** (KuZimm038) (Method 2):

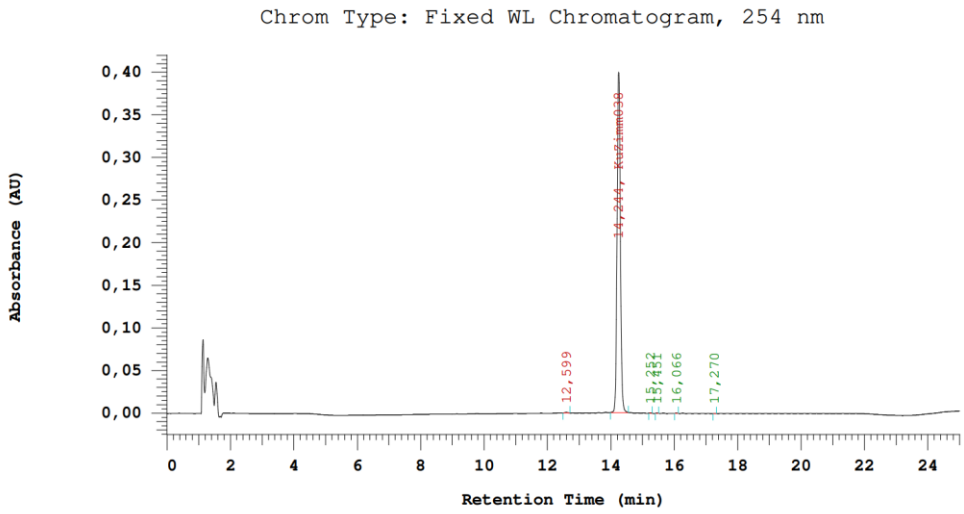

HPLC chromatogram of **9c** (KuZimm001) (Method 2):

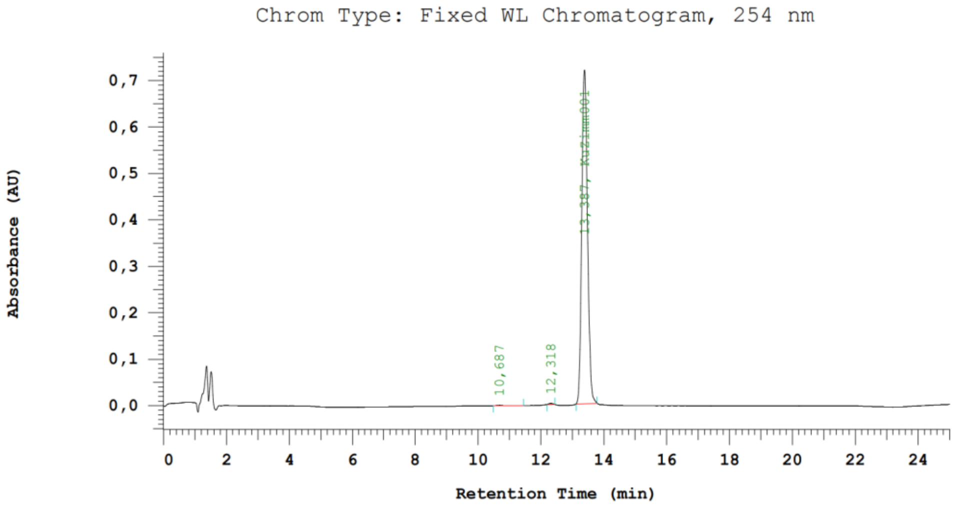

HPLC chromatogram of **9d** (KuZimm039) (Method 2):

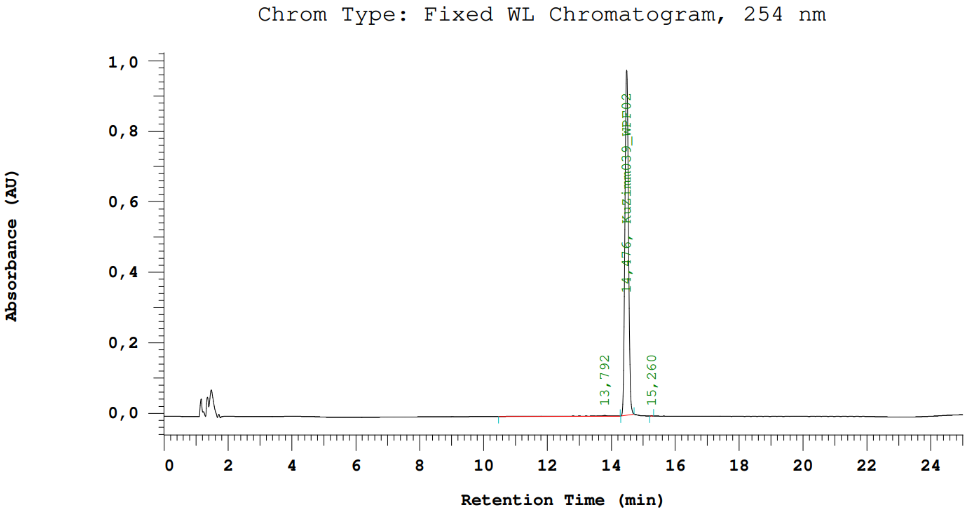

HPLC chromatogram of **9e** (KuZimm027) (Method 2):

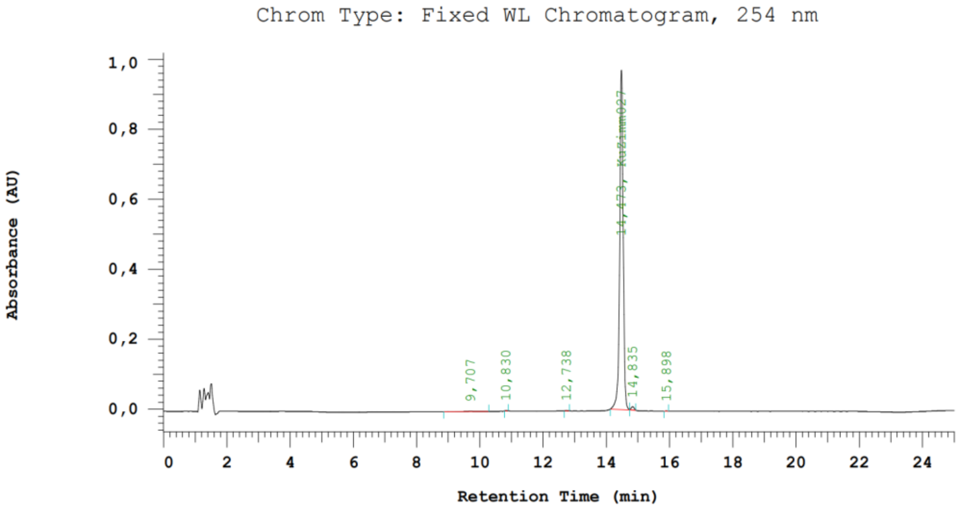

HPLC chromatogram of **9f** (KuThi018) (Method 2):

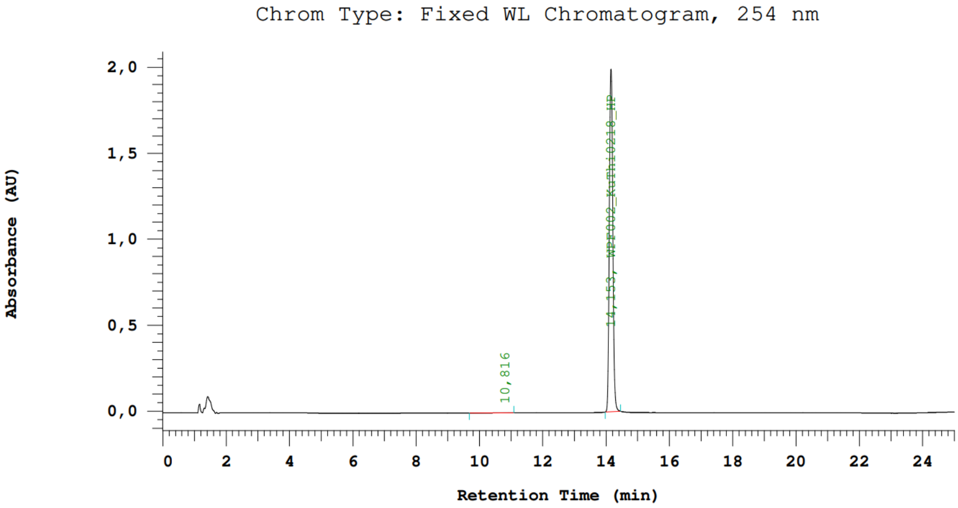

HPLC chromatogram of **9h** (KuThi021) (Method 2):

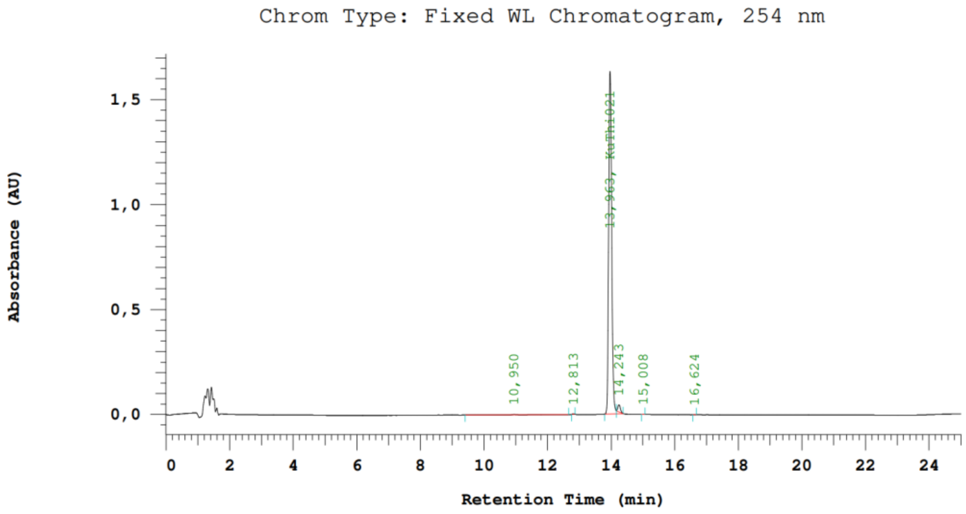

HPLC chromatogram of **9i** (KuThi025) (Method 2):

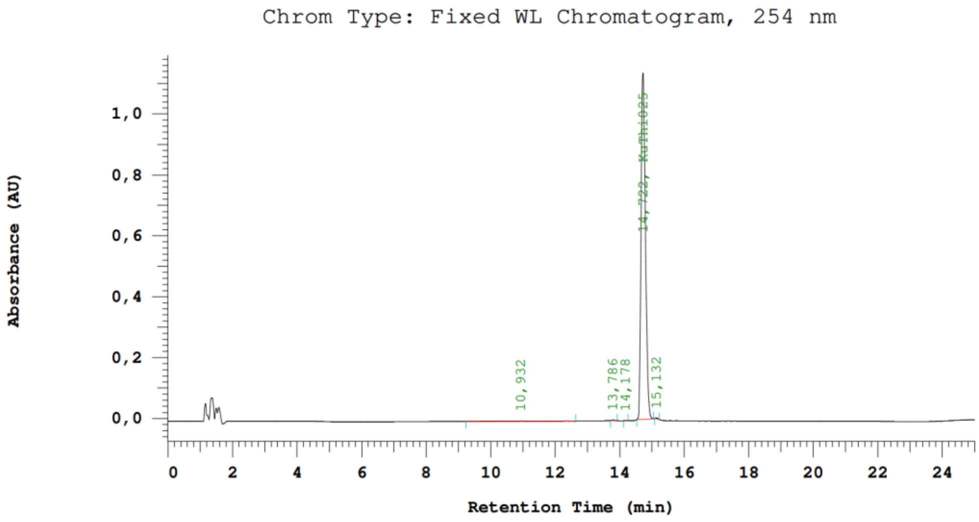

HPLC chromatogram of **9j** (KuThi024) (Method 2):

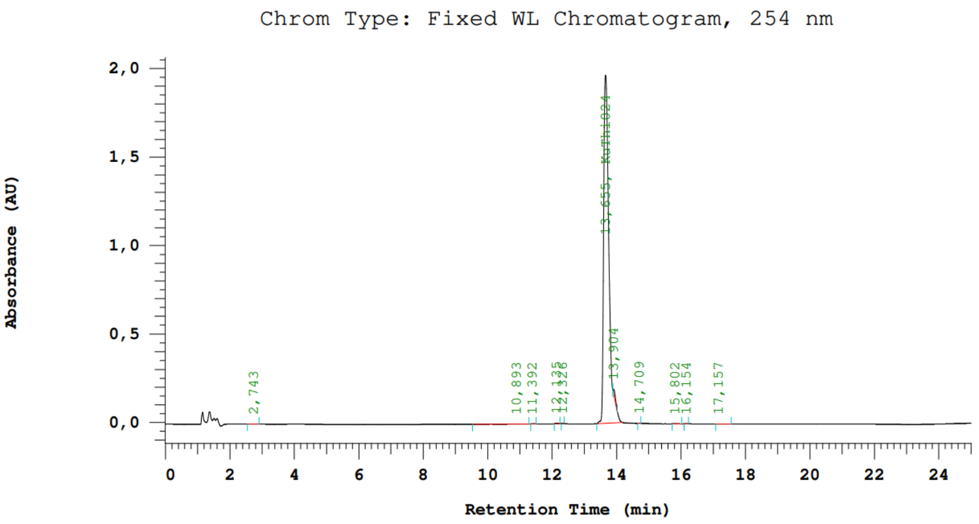

HPLC chromatogram of **9k** (KuThi017) (Method 2):

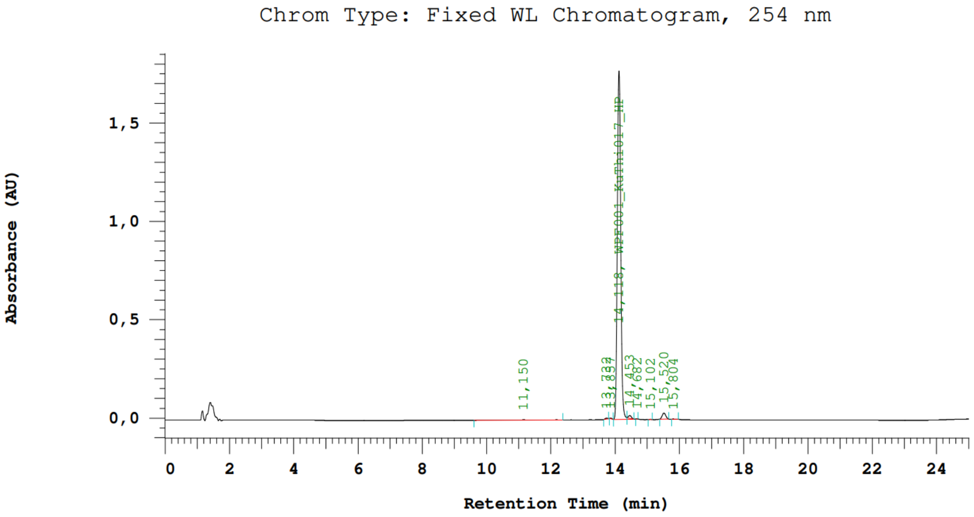

HPLC chromatogram of **9l** (KuThi001) (Method 2):

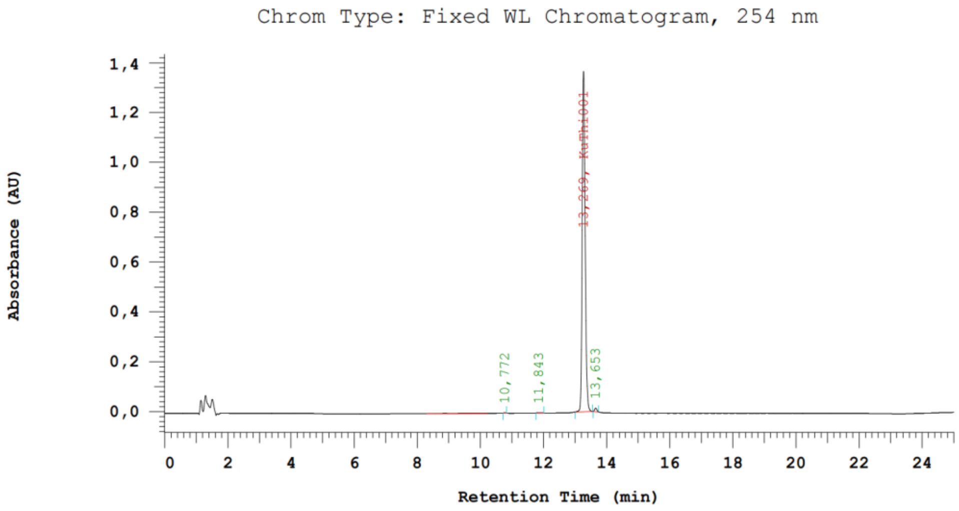

HPLC chromatogram of **10a** (KuThi036) (Method 2):

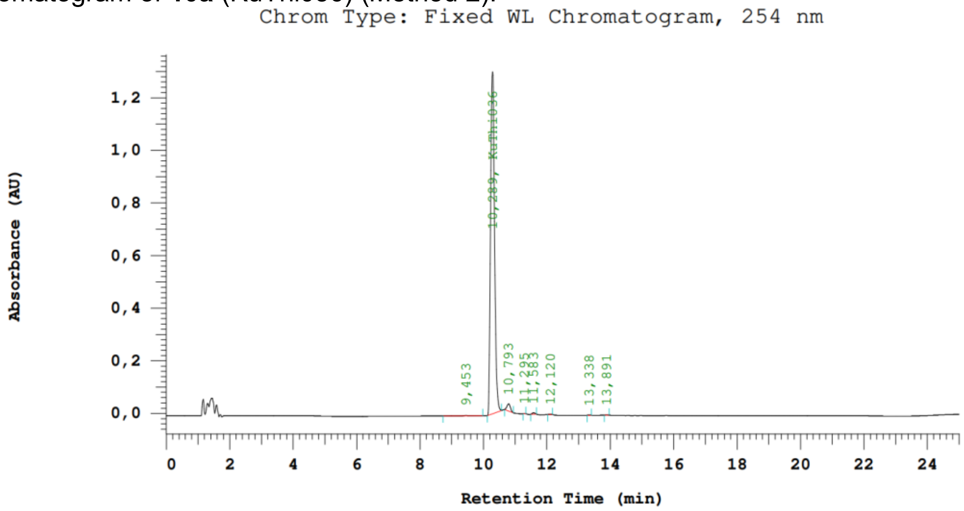

HPLC chromatogram of **10b** (KuZimm040) (Method 2):

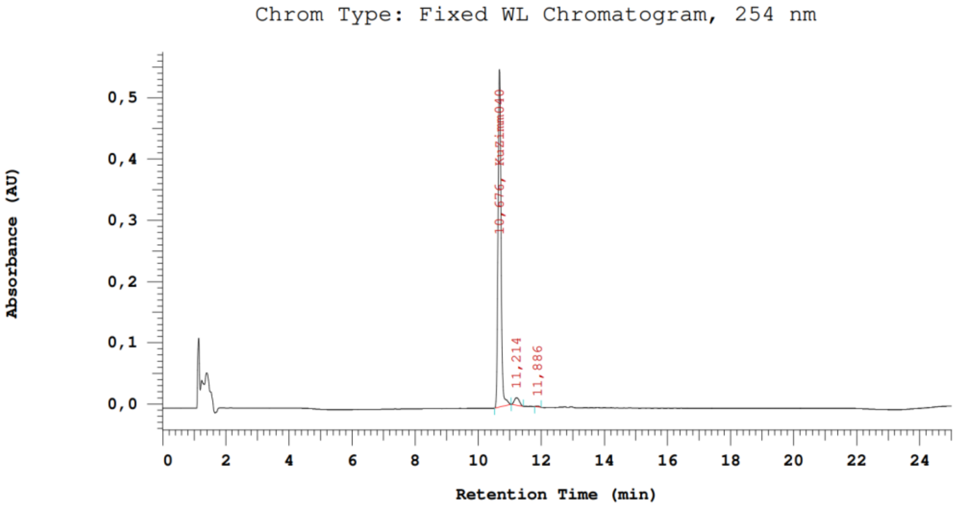

HPLC chromatogram of **10c** (KuZimm044) (Method 2):

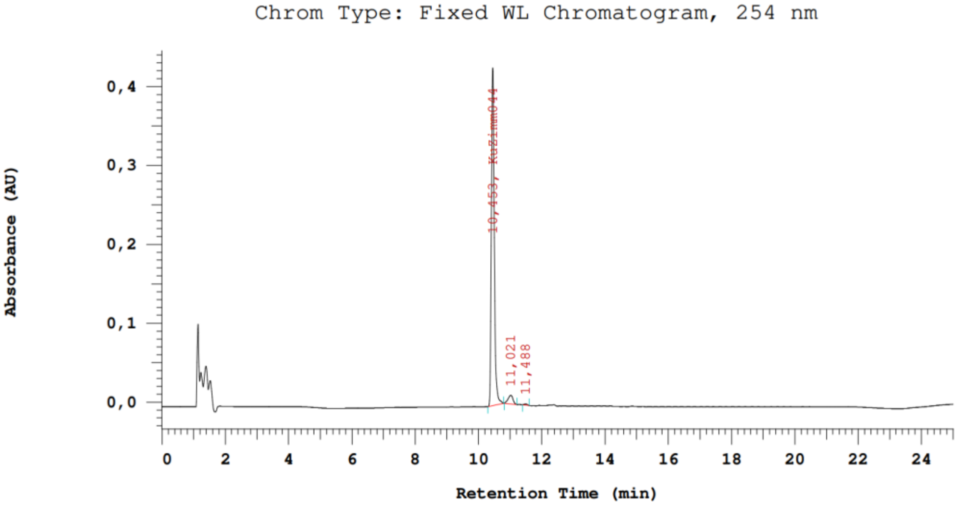

HPLC chromatogram of **10d** (KuZimm041) (Method 2):

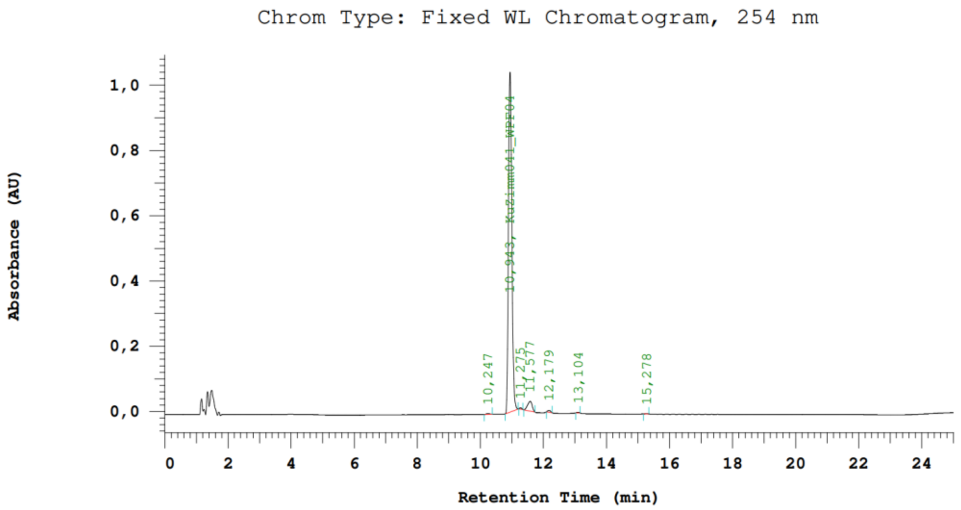

HPLC chromatogram of **10e** (KuZimm045) (Method 2):

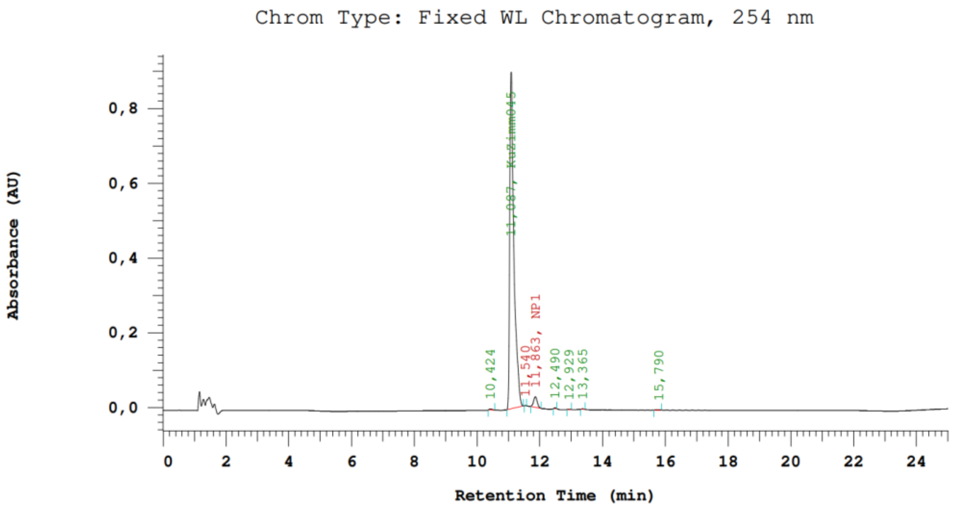

HPLC chromatogram of **10f** (KuThi030) (Method 2):

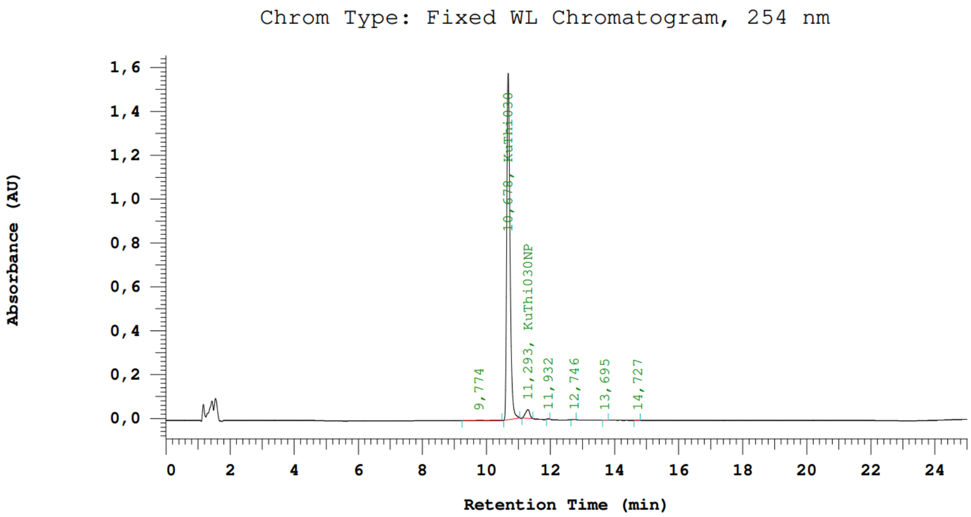

HPLC chromatogram of **10g** (KuThi033) (Method 2):

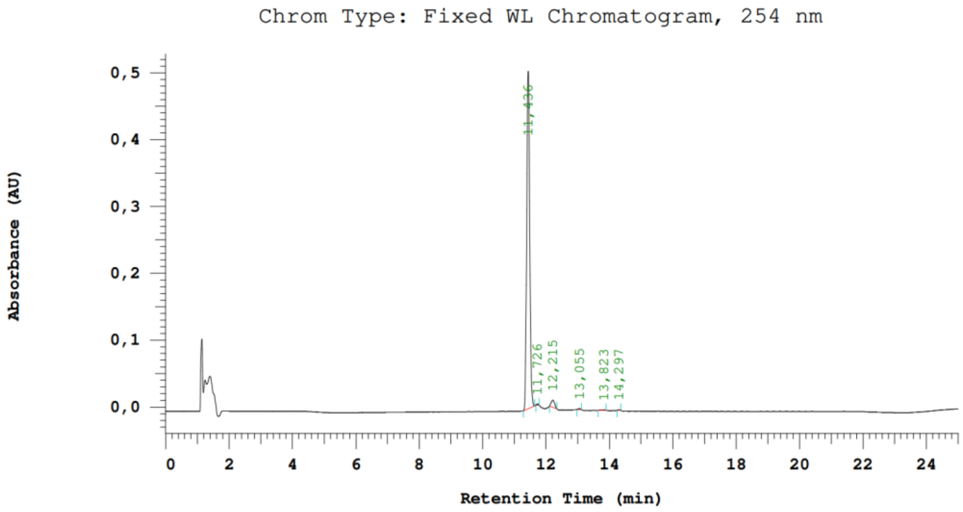

HPLC chromatogram of **10h** (KuThi022) (Method 2):

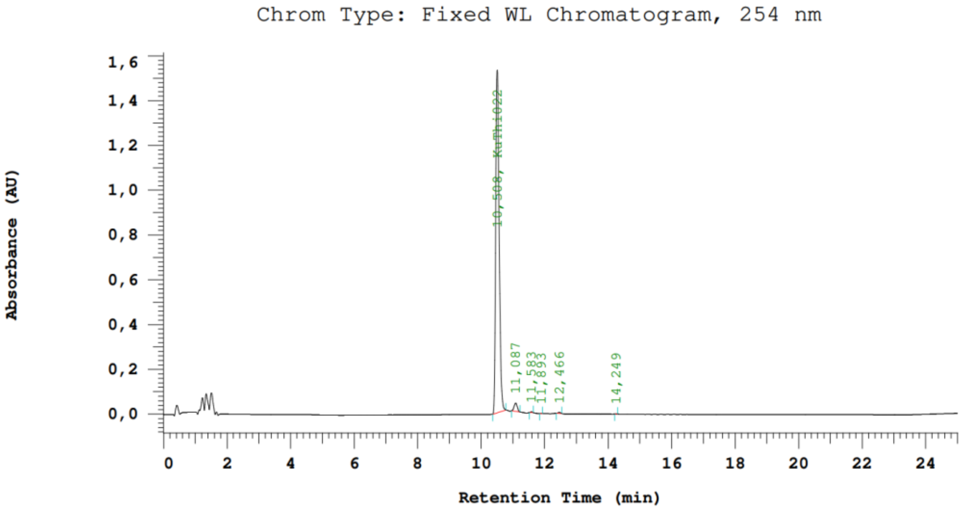

HPLC chromatogram of **10i** (KuThi029) (Method 2):

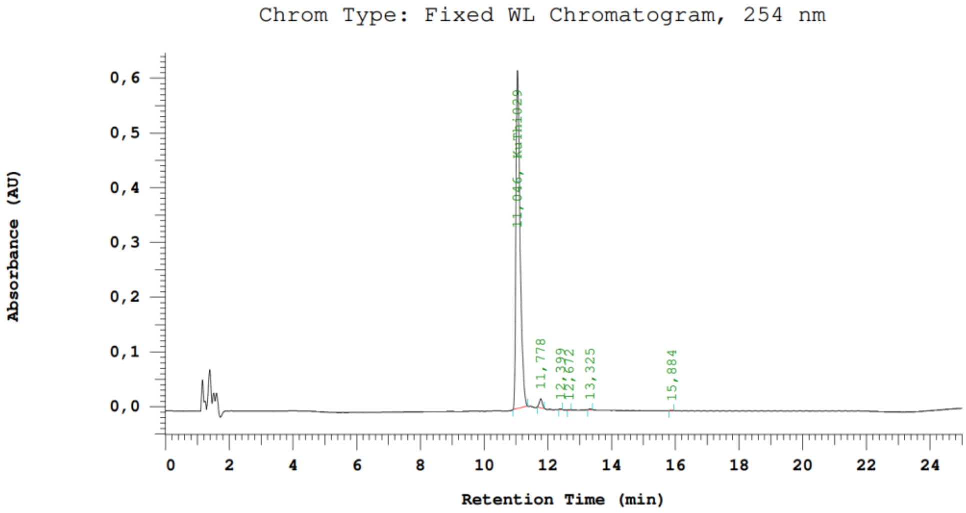

HPLC chromatogram of **10j** (KuThi028) (Method 2):

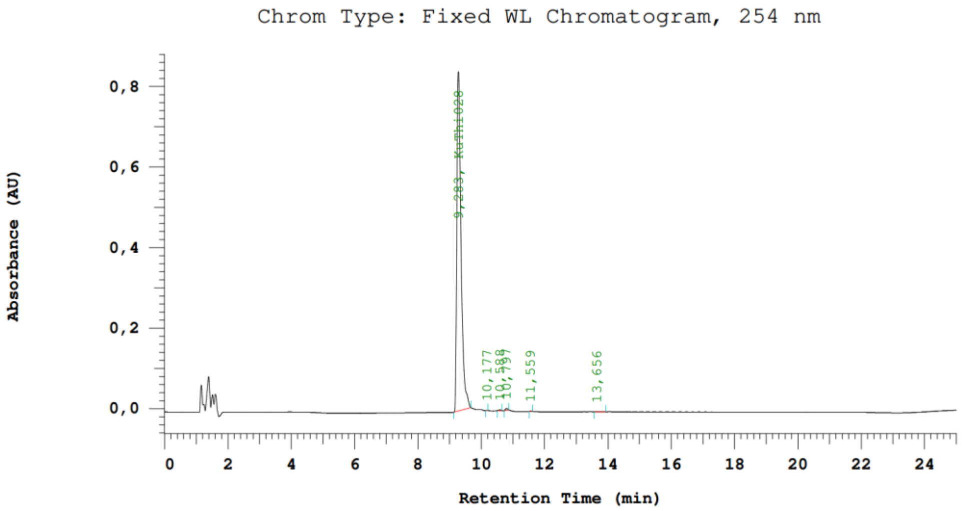

HPLC chromatogram of **10k** (KuThi019) (Method 2):

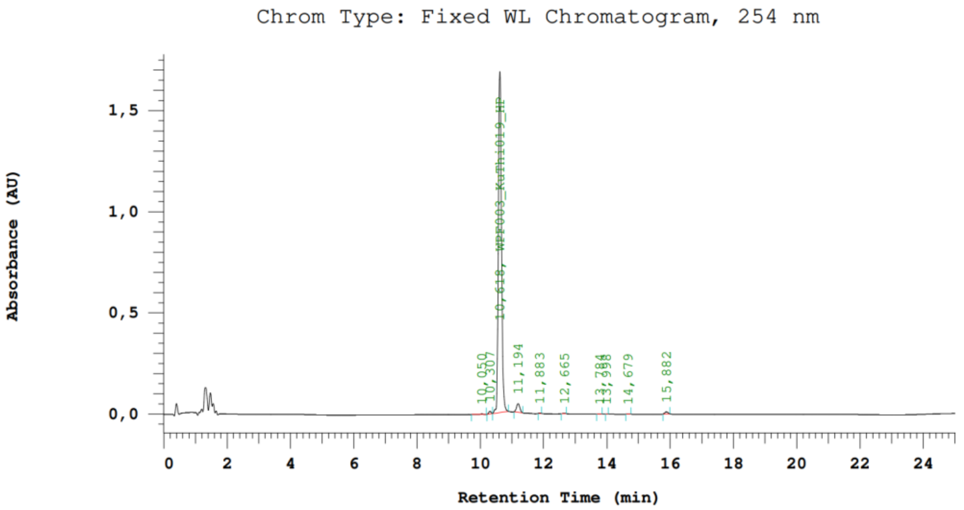

HPLC chromatogram of **10l** (KuThi002) (Method 2):

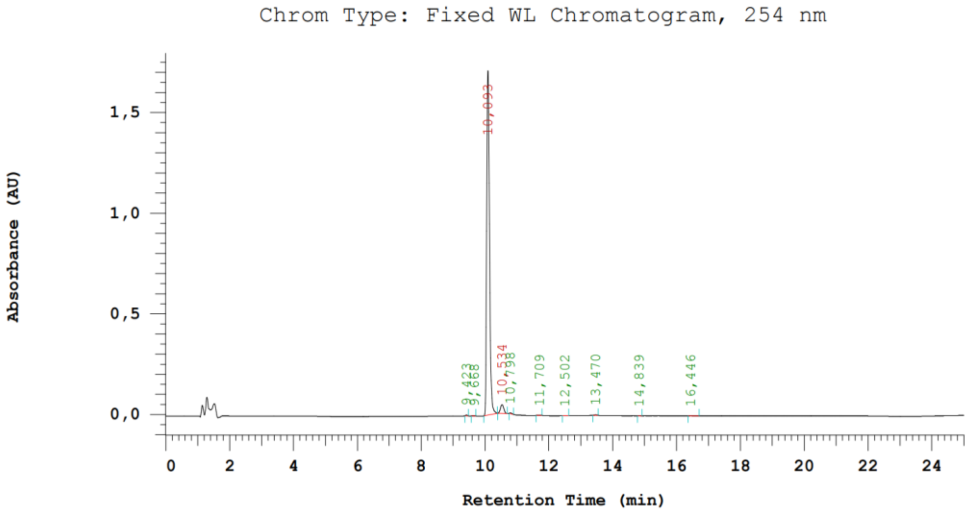

HPLC chromatogram of **11a** (KuZimm030) (Method 2):

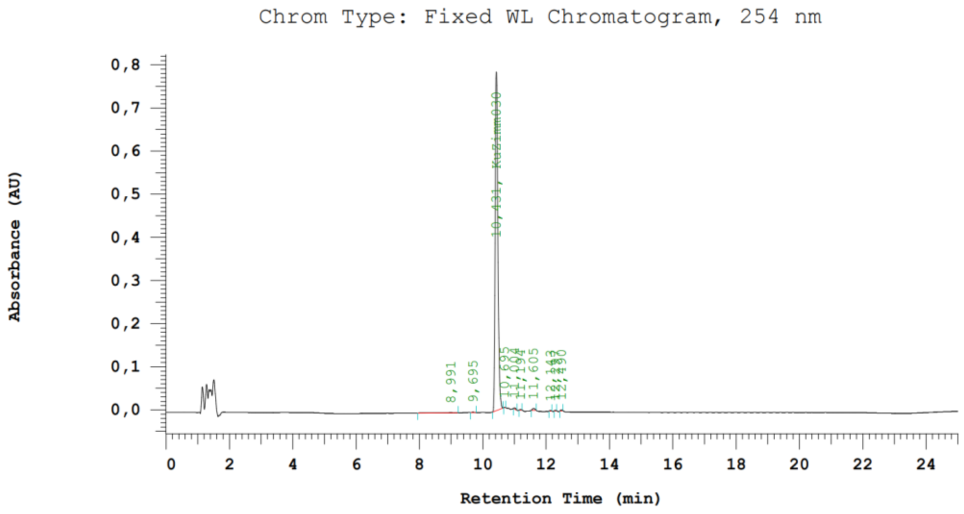

HPLC chromatogram of **11b** (KuZimm031) (Method 2):

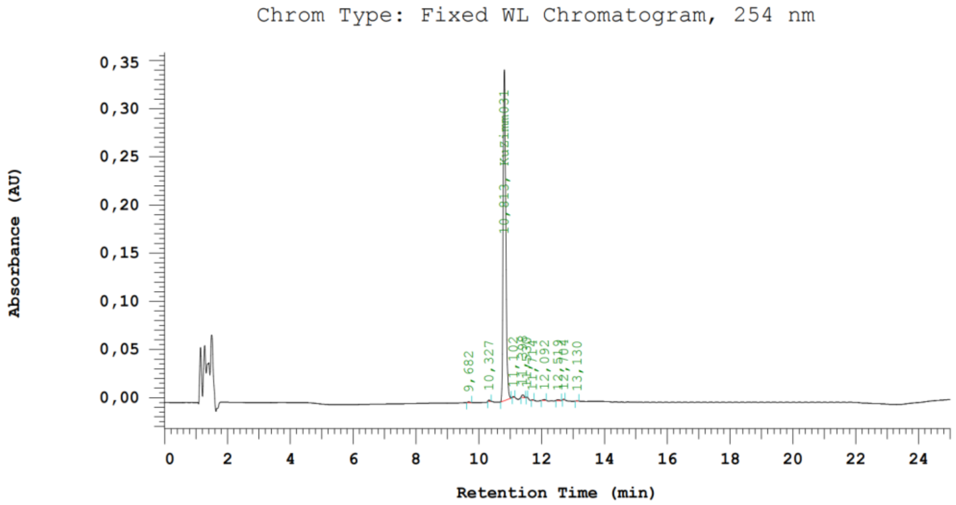

HPLC chromatogram of **11c** (KuZimm029) (Method 2):

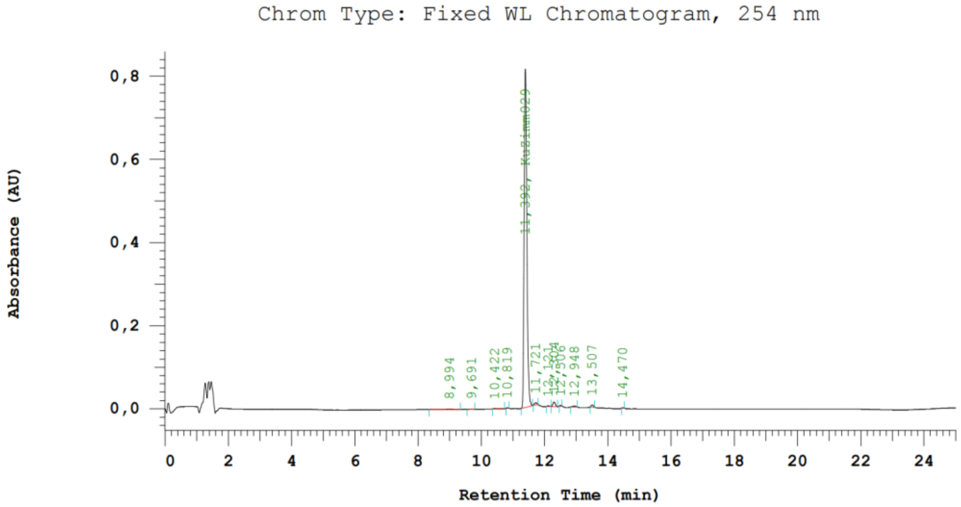

HPLC chromatogram of **11d** (KuThi032) (Method 2):

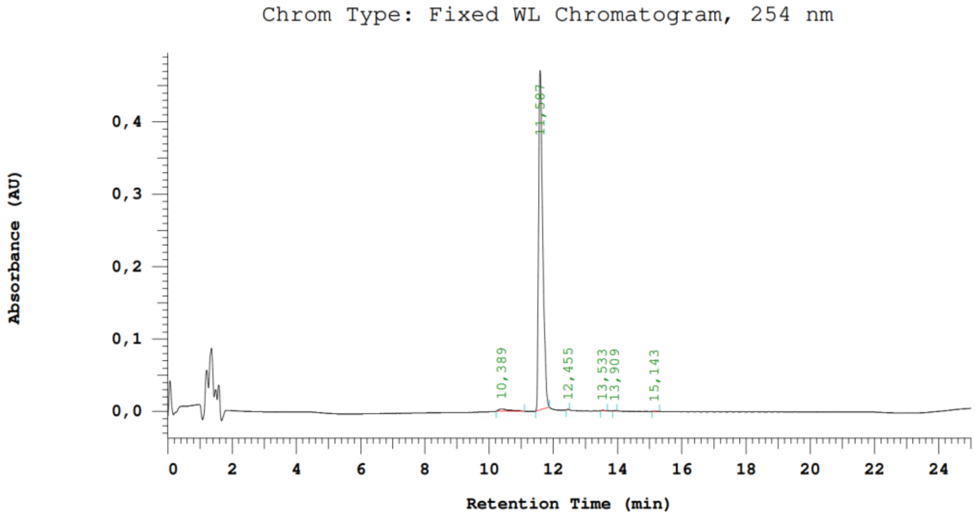

HPLC chromatogram of **11e** (KuThi009) (Method 2):

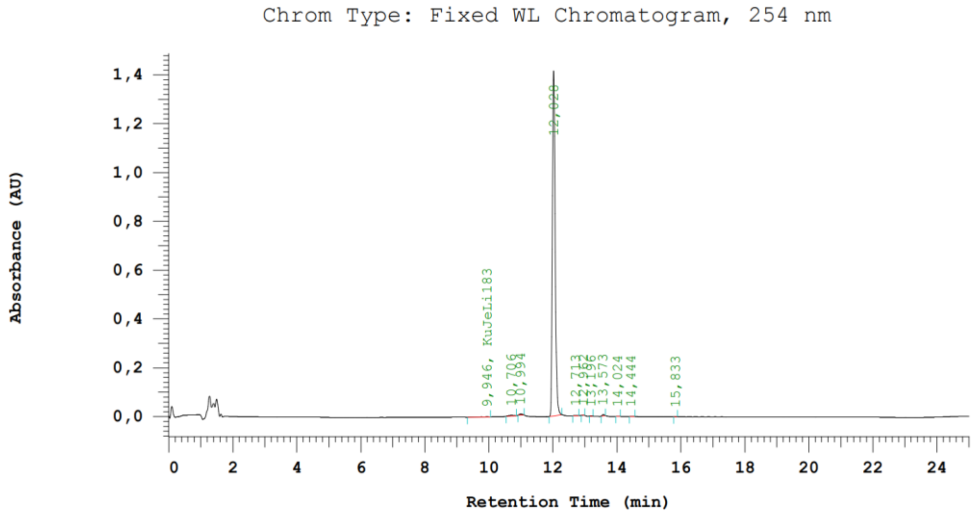

HPLC chromatogram of **11f** (KuThi005) (Method 2):

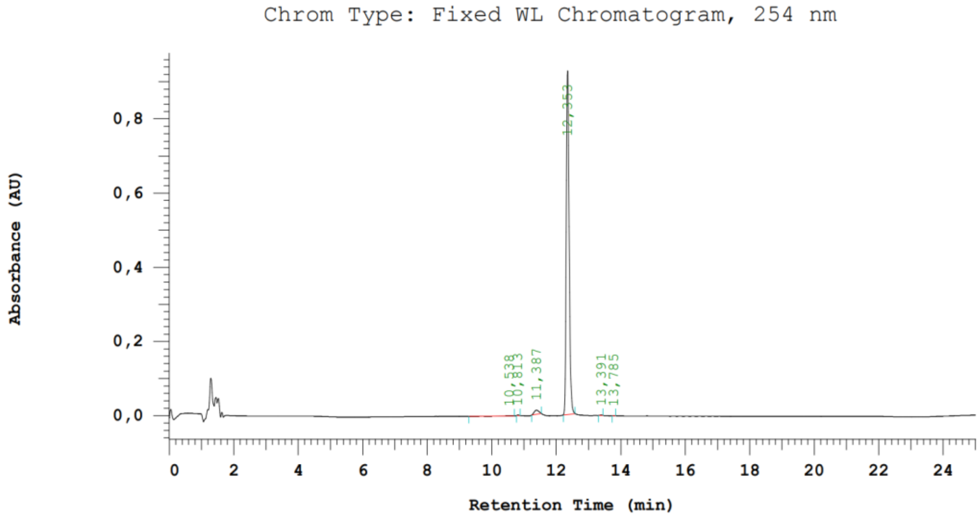

HPLC chromatogram of **11g** (KuThi010) (Method 1):

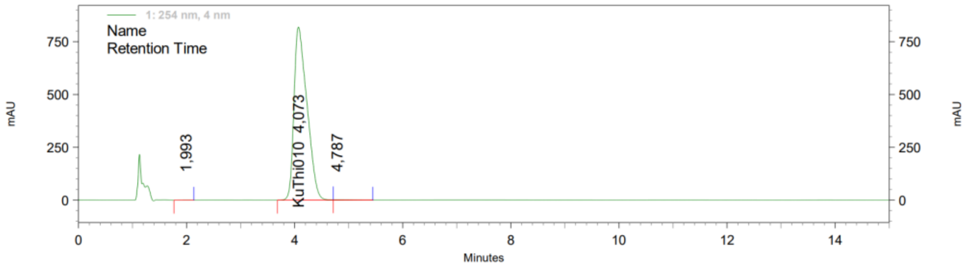

HPLC chromatogram of **12a** (KuZimm020) (Method 2):

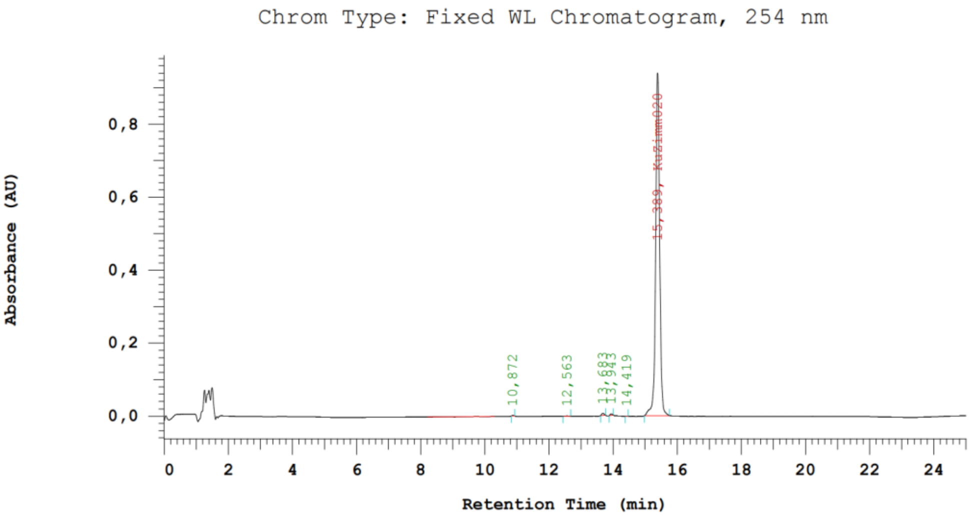

HPLC chromatogram of **12b** (KuZimm019) (Method 2):

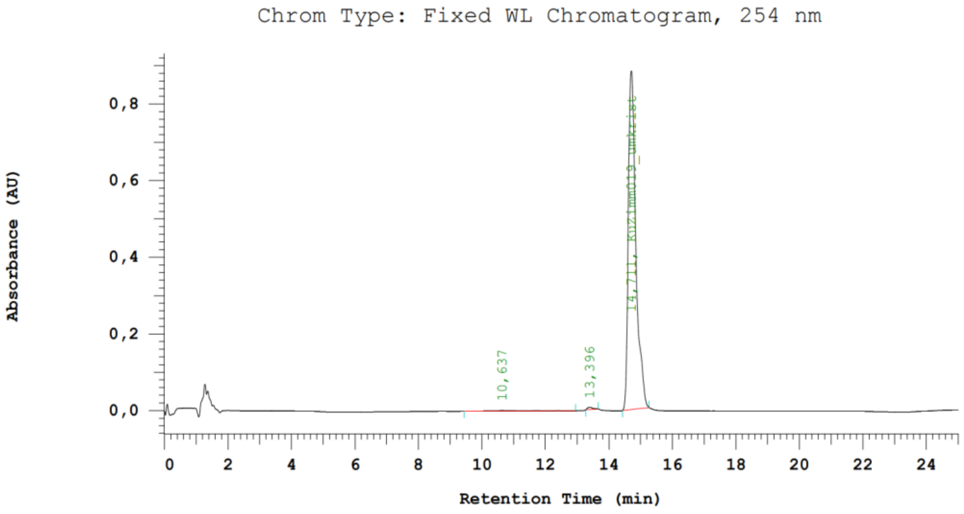

HPLC chromatogram of **12c** (KuZimm018) (Method 2):

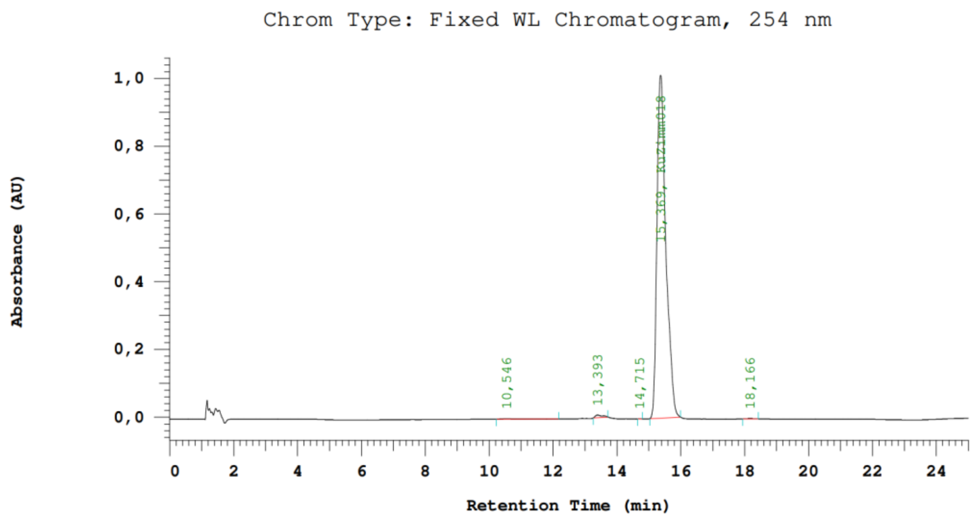

HPLC chromatogram of **12d** (KuThi012) (Method 2):

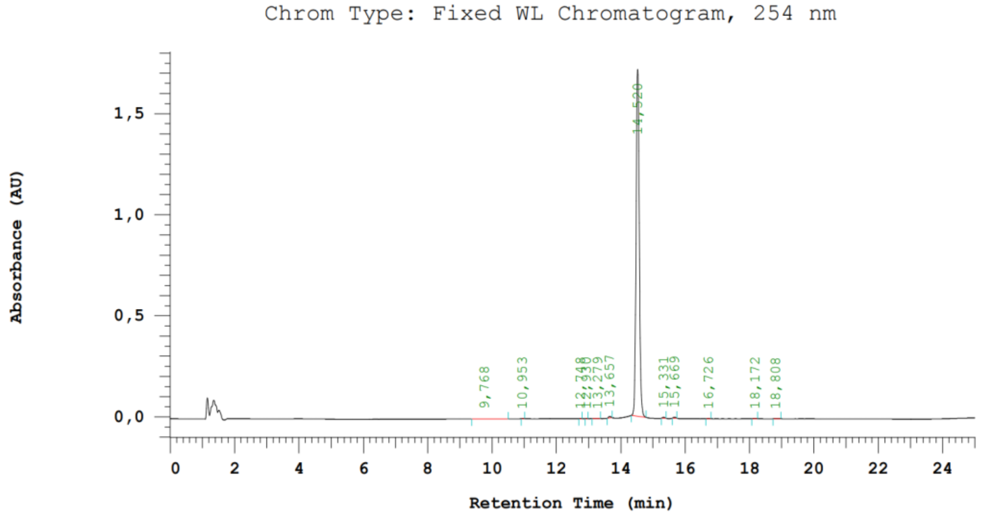

HPLC chromatogram of **13a** (KuZimm023) (Method 2):

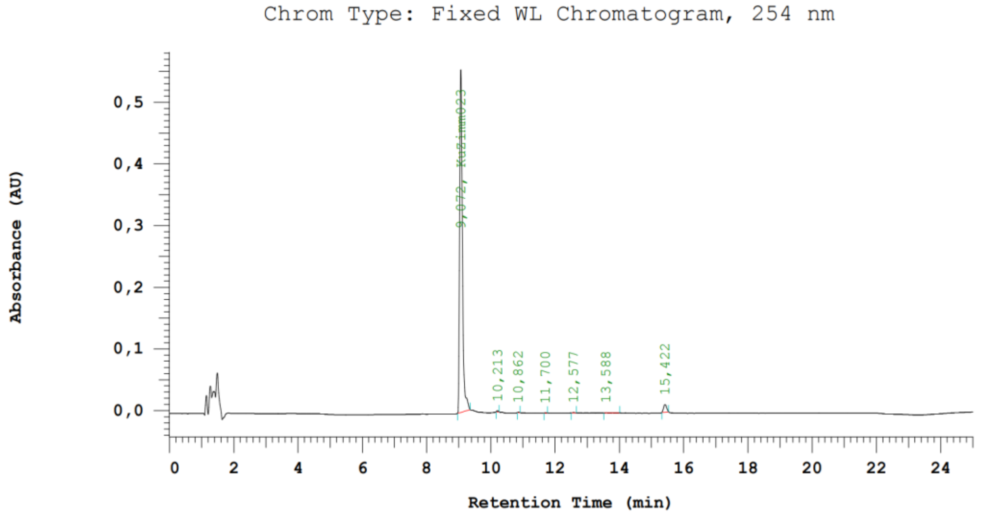

HPLC chromatogram of **13b** (KuZimm022) (Method 2):

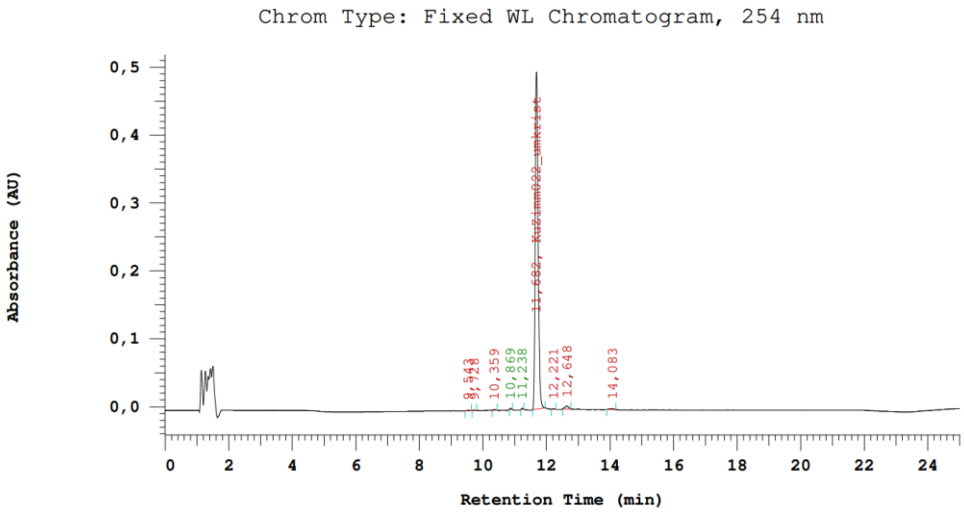

HPLC chromatogram of **13c** (KuZimm021) (Method 2):

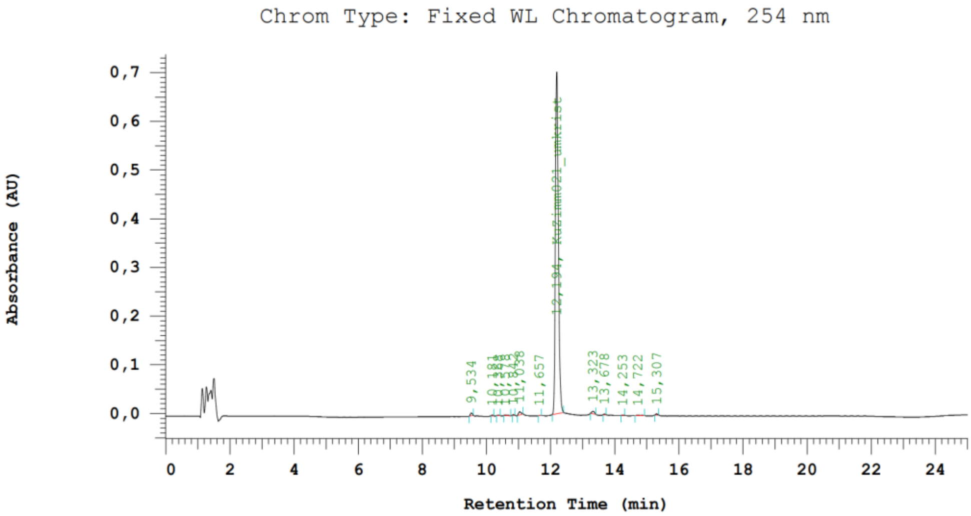

HPLC chromatogram of **13d** (KuThi013) (Method 2):

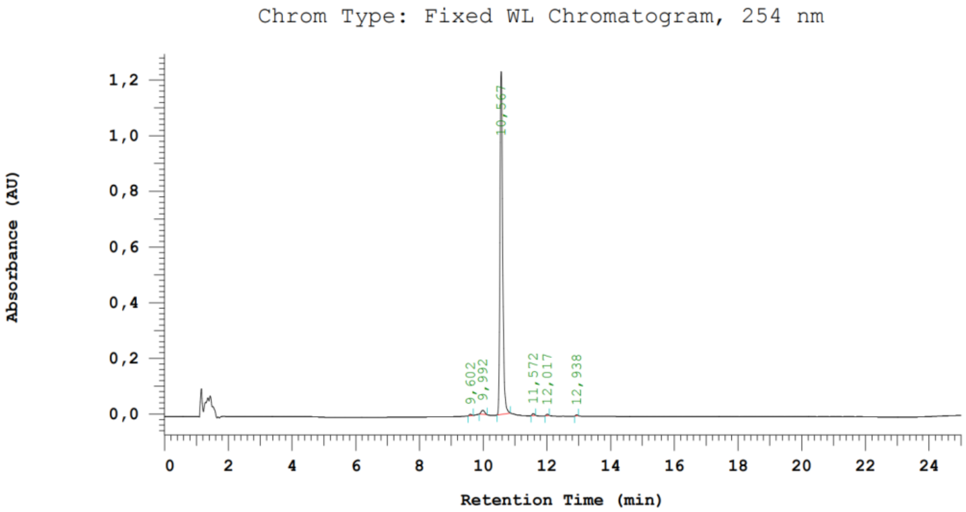

HPLC chromatogram of **13e** (KuThi016) (Method 2):

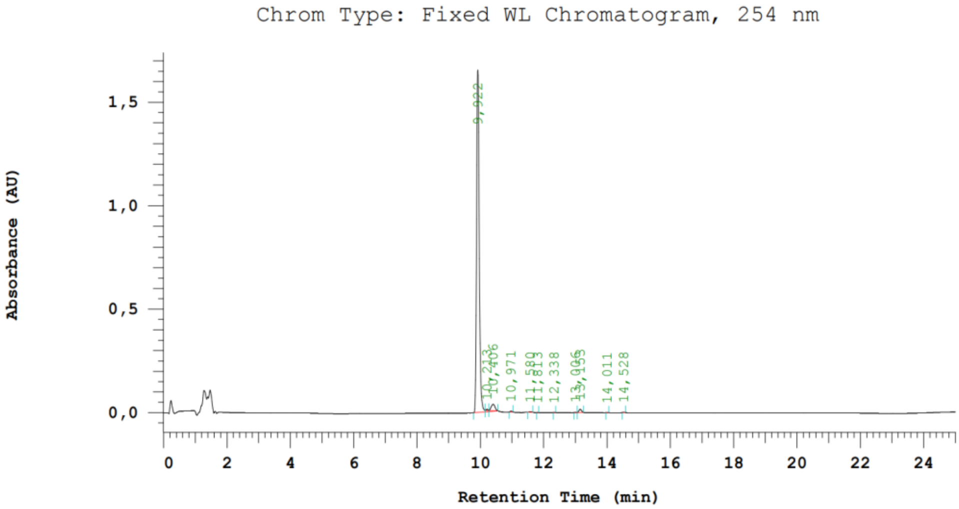

HPLC chromatogram of **13f** (KuThi015) (Method 2):

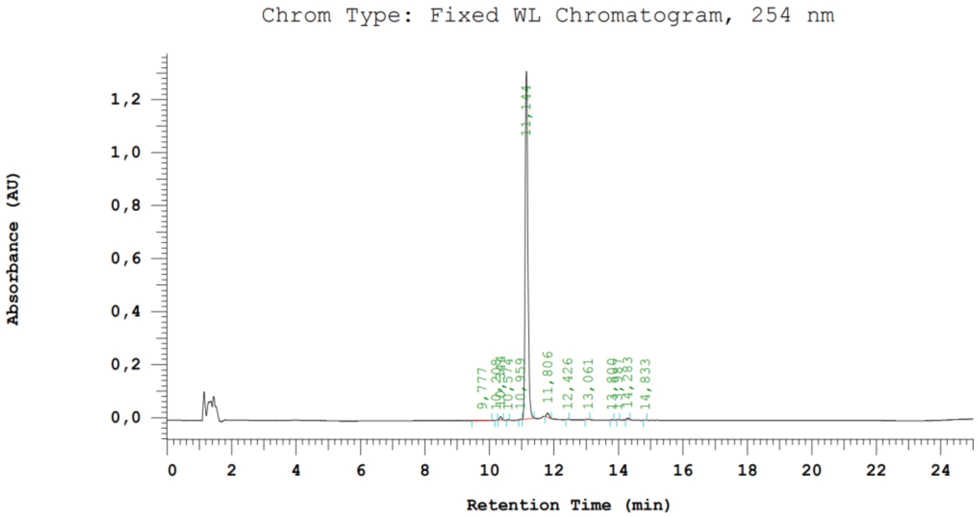

HPLC chromatogram of **13g** (KuThi014) (Method 2):

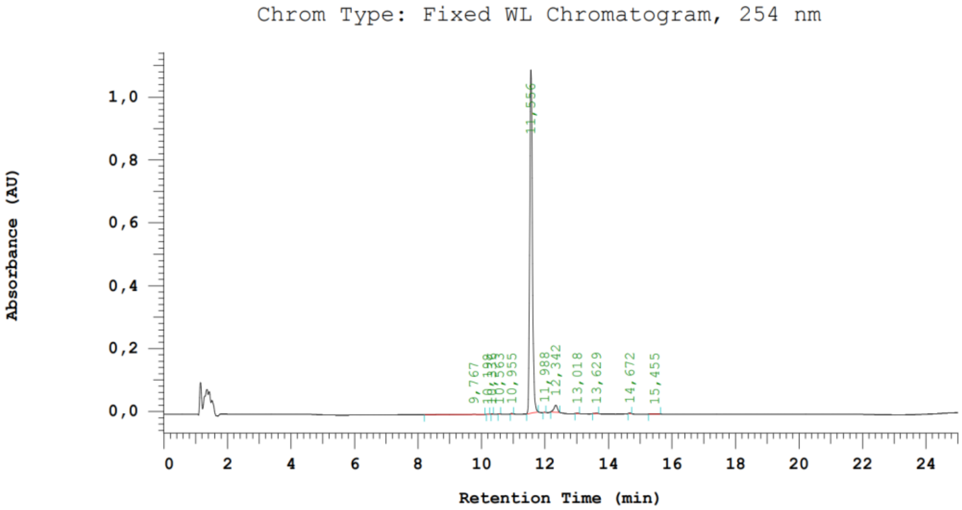

HPLC chromatogram of **15a** (KuZimm064) (Method 2):

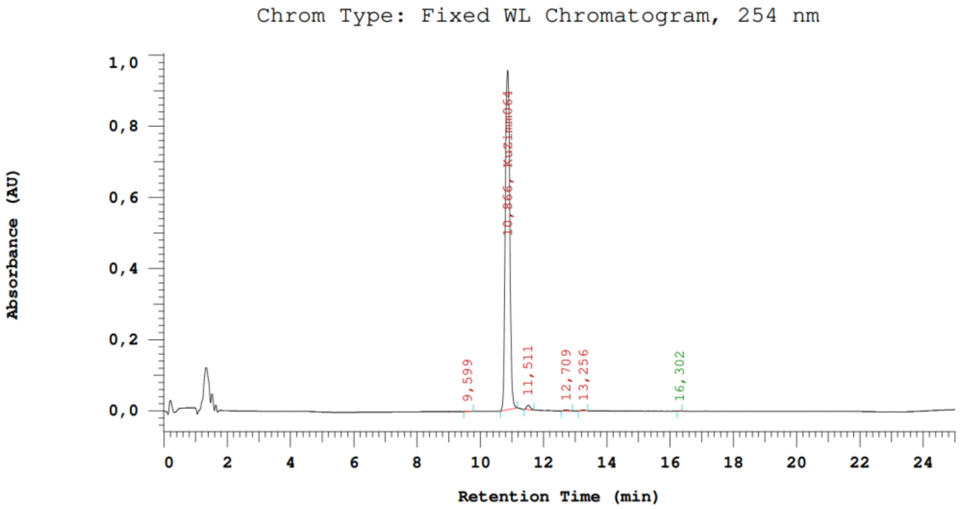

HPLC chromatogram of **15b** (KuZimm063) (Method 2):

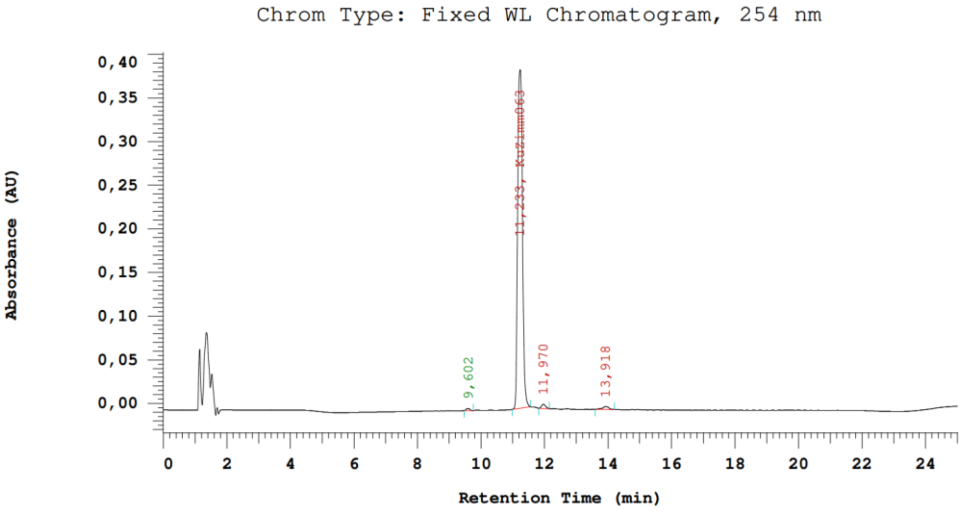

HPLC chromatogram of **15c** (KuZimm047) (Method 2):

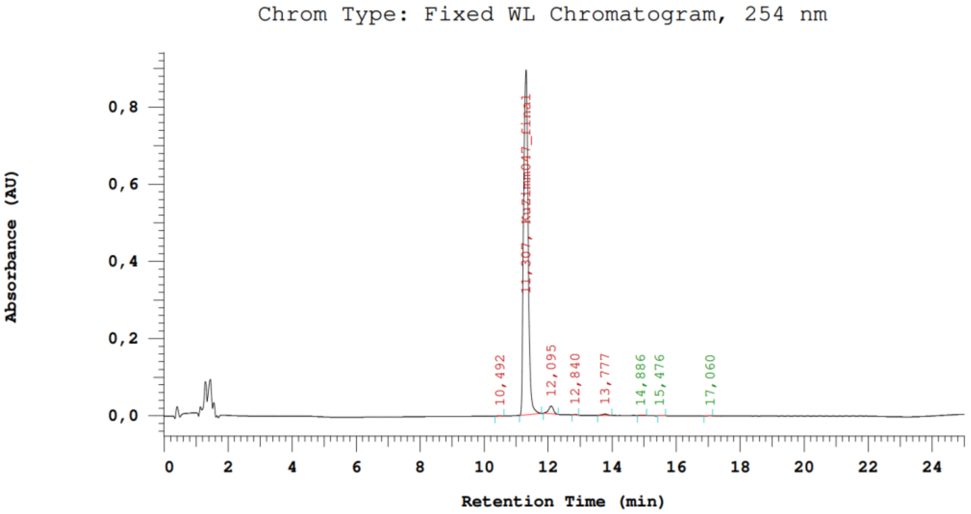

HPLC chromatogram of **19e** (KuZimm075) (Method 2):

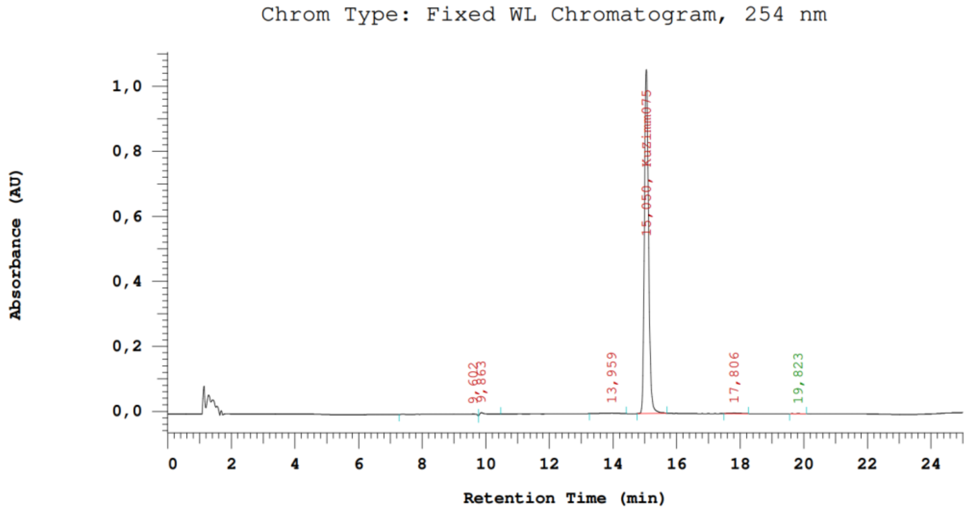

HPLC chromatogram of **19f** (KuThi067) (Method 2):

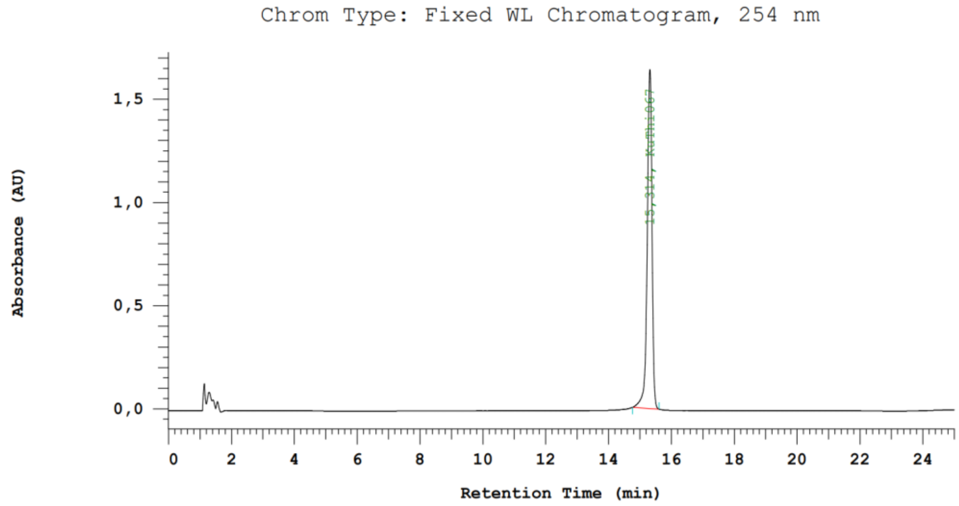

HPLC chromatogram of **20a** (KuZimm068) (Method 2):

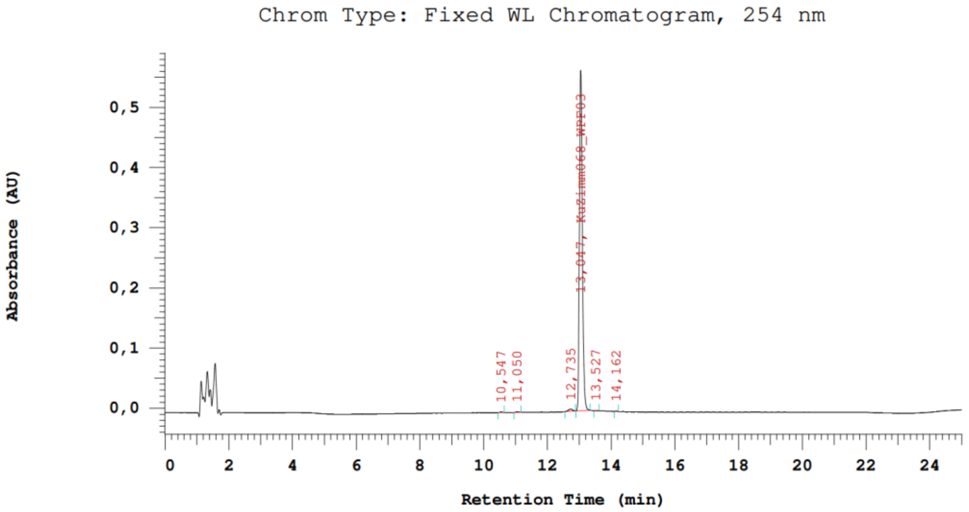

HPLC chromatogram of **20b** (KuZimm074) (Method 2):

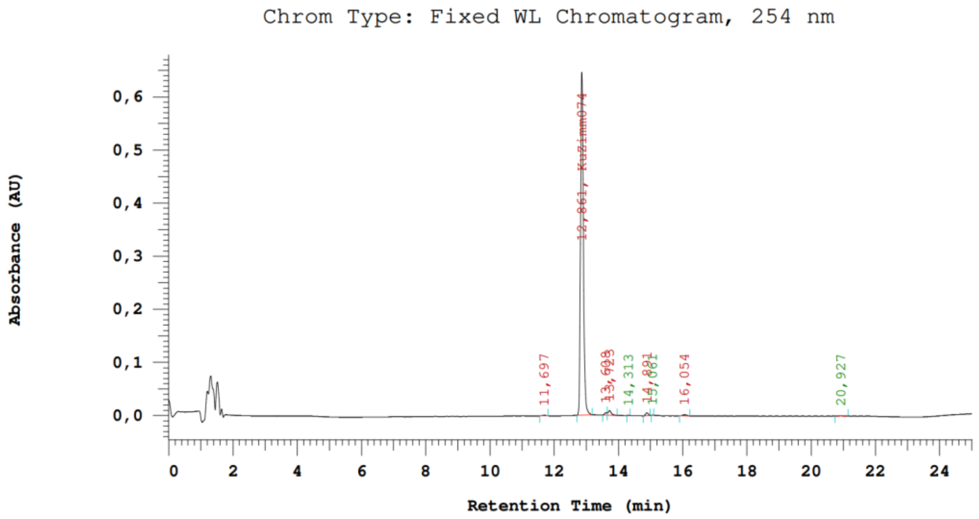

HPLC chromatogram of **20c** (KuThi070) (Method 2):

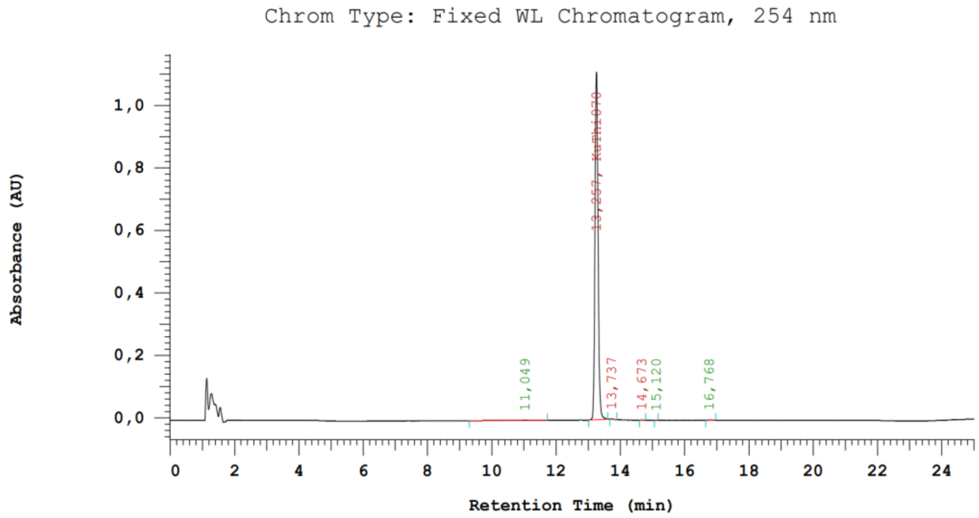

HPLC chromatogram of **20d** (KuThi071) (Method 2):

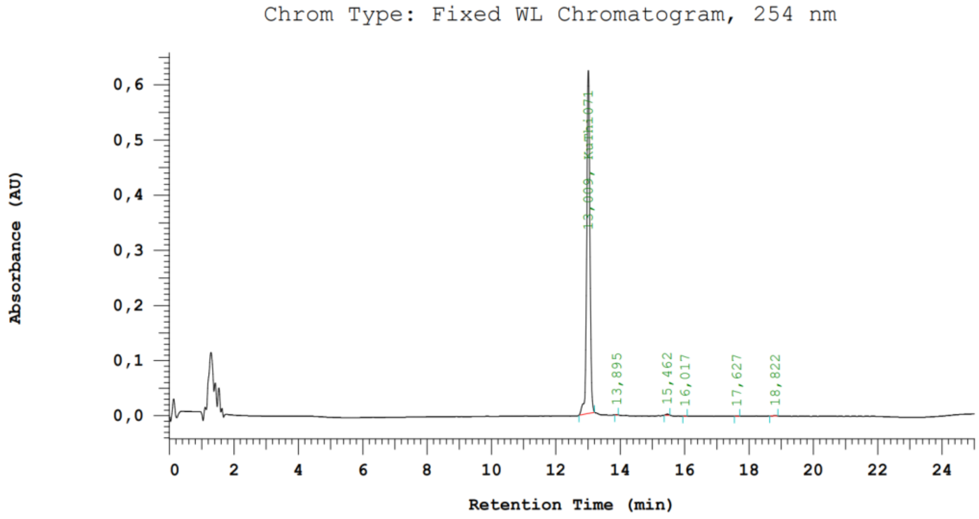

Supplementary References

1 Schütz, C.; Empting, M. Targeting the *Pseudomonas* quinolone signal quorum sensing system for the discovery of novel anti-infective pathoblockers. *Beilstein J. Org. Chem.* **2018**, *14*, 2627–2645, DOI: 10.3762/bjoc.14.241.

2 Starkey, M.; Lepine, F.; Maura, D.; Bandyopadhaya, A.; Lesic, B.; He, J.; Kitao, T.; Righi, V.; Milot, S.; Tzika, A.; Rahme, L. Identification of anti-virulence compounds that disrupt quorum-sensing regulated acute and persistent pathogenicity. *PLoS Pathog.* **2014**, *10*, e1004321, DOI: 10.1371/journal.ppat.1004321.

3 Hamed, M. M.; Abdelsamie, A. S.; Rox, K.; Schütz, C.; Kany, A. M.; Röhrig, T.; Schmelz, S.; Blankenfeldt, W.; Arce-Rodriguez, A.; Borrero-de Acuña, J. M.; Jahn, D.; Rademacher, J.; Ringshausen, F. C.; Cramer, N.; Tümmler, B.; Hirsch, A. K. H.; Hartmann, R. W.; Empting, M. Towards translation of PqsR inverse agonists: from *in vitro* efficacy optimization to *in vivo* proof-of-principle. *Adv. Sci.* **2023**, *10*, e2204443, DOI: 10.1002/advs.202204443.

4 Ahuja, E. G.; Janning, P.; Mentel, M.; Graebsch, A.; Breinbauer, R.; Hiller, W.; Costisella, B.; Thomashow, L. S.; Mavrodi, D. V.; Blankenfeldt, W. PhzA/B catalyzes the formation of the tricycle in phenazine biosynthesis. *J. Am. Chem. Soc.* **2008**, *130*, 17053–17061, DOI: 10.1021/ja806325k.

5 Pletz, J.; Berg, B.; Breinbauer, R. A general and direct reductive amination of aldehydes and ketones with electron-deficient anilines. *Synthesis* **2016**, *48*, 1301–1317, DOI: 10.1055/s-0035-1561384.

6 Mentel, M.; Breinbauer, R.; Blankenfeldt, W. Crystal structure of PhzA/B from Burkholderia cepacia R18194 in complex with 5-bromo-2-((1S,3R)-3-carboxycyclohexylamino)benzoic acid. *Protein Data Bank* **2009**, DOI: 10.2210/pdb3jum/pdb.

7 Mentel, M.; Blankenfeldt, W.; Breinbauer, R. The active site of an enzyme can host both enantiomers of a racemic ligand simultaneously. *Angew. Chem., Int. Ed. Engl.* **2009**, *48*, 9084–9087, DOI: 10.1002/anie.200902997.

8 Mentel, M.; Breinbauer, R.; Blankenfeldt, W. Crystal Structure of PhzA/B from Burkholderia cepacia R18194 in simultaneous complex with racemic 5-bromo-2-(piperidin-3-ylamino)benzoic acid. *Protein Data Bank* **2009**, DOI: 10.2210/pdb3JUN/pdb.

9 Mentel, M.; Breinbauer, R.; Blankenfeldt, W. Crystal Structure of PhzA/B from Burkholderia cepacia R18194 cocrystallized with 2 mM racemic 5-bromo-2-(piperidin-3-ylamino)benzoic acid. *Protein Data Bank* **2009**, DOI: 10.2210/pdb3JUQ/pdb.

10 Mentel, M.; Jain, I. H.; Breinbauer, R.; Blankenfeldt, W. Crystal Structure of PhzA/B from Burkholderia cepacia R18194 in complex with (R)-5-bromo-2-(piperidin-3-ylamino)benzoic acid. *Protein Data Bank* **2009**, DOI: 10.2210/pdb3JUO/pdb.

11 Mentel, M.; Jain, I. H.; Breinbauer, R.; Blankenfeldt, W. Crystal Structure of PhzA/B from Burkholderia cepacia R18194 in complex with (S)-5-bromo-2-(piperidin-3-ylamino)benzoic acid. *Protein Data Bank* **2009**, DOI: 10.2210/pdb3JUP/pdb.

12 Froes, T. Q.; Guido, R. V.; Metwally, K.; Castilho, M. S. A novel scaffold to fight *Pseudomonas aeruginosa* pyocyanin production: early steps to novel antivirulence drugs. *Future Med. Chem.* **2020**, *12*, 1489–1503, DOI: 10.4155/fmc-2019-0351.

13 Richter, M. F.; Drown, B. S.; Riley, A. P.; Garcia, A.; Shirai, T.; Svec, R. L.; Hergenrother, P. J. Predictive compound accumulation rules yield a broad-spectrum antibiotic. *Nature* **2017**, *545*, 299–304, DOI: 10.1038/nature22308.

14 Geddes, E. J.; Gugger, M. K.; Garcia, A.; Chavez, M. G.; Lee, M. R.; Perlmutter, S. J.; Bieniossek, C.; Guasch, L.; Hergenrother, P. J. Porin-independent accumulation in *Pseudomonas* enables antibiotic discovery. *Nature* **2023**, *624*, 145–153, DOI: 10.1038/s41586-023-06760-8.
